# Age-sex population adjusted analysis of disease severity in epidemics as a tool to devise public health policies for COVID-19

**DOI:** 10.1038/s41598-021-89615-4

**Published:** 2021-06-03

**Authors:** Carlo Vittorio Cannistraci, Maria Grazia Valsecchi, Ilaria Capua

**Affiliations:** 1 Center for Complex Network Intelligence (CCNI) at the Tsinghua Laboratory of Brain and Intelligence (THBI), Department of Biomedical Engineering, Tsinghua University, 160 Chengfu Rd., SanCaiTang Building, Haidian District, Beijing, 100084 China; 2 Biomedical Cybernetics Group, Biotechnology Center (BIOTEC), Center for Molecular and Cellular Bioengineering (CMCB), Center for Systems Biology Dresden (CSBD), Department of Physics, Technische Universität Dresden, Tatzberg 47/49, 01307 Dresden, Germany; 3 Bicocca Center of Bioinformatics, Biostatistics and Bioimaging, School of Medicine and Surgery, University of Milano-Bicocca, Monza, Italy; 4 One Health Center of Excellence, University of Florida, Gainesville, FL USA

**Keywords:** Data processing, Data mining, Epidemiology

## Abstract

Governments continue to update social intervention strategies to contain COVID-19 infections. However, investigation of COVID-19 severity indicators across the population might help to design more precise strategies, balancing the need to keep people safe and to reduce the socio-economic burden of generalized restriction precedures. Here, we propose a method for age-sex population-adjusted analysis of disease severity in epidemics that has the advantage to use simple and repeatable variables, which are daily or weekly available. This allows to monitor the effect of public health policies in short term, and to repeat these calculations over time to surveille epidemic dynamics and impact. Our method can help to define a risk-categorization of likeliness to develop a severe COVID-19 disease which requires intensive care or is indicative of a higher risk of dying. Indeed, analysis of suitable open-access COVID-19 data in three European countries indicates that individuals in the 0–40 age interval and females under 60 are significantly less likely to develop a severe condition and die, whereas males equal or above 60 are more likely at risk of severe disease and death. Hence, a combination of age-adaptive and sex-balanced guidelines for social interventions could represent key public health management tools for policymakers.

## Introduction

Many countries now find themselves managing a more mature series of actions as they seek to stabilize, and subsequently exit from, the post COVID-19 emergency phase. This is a real-life experiment which aims to re-establish a productive and socially acceptable environment after government-imposed lockdowns have mitigated viral spread^1^ with varying degrees of success. In 2020, in the absence of vaccines against Sars-CoV-2, mitigation of pandemic spread at a national level was achieved mostly by implementing blanket-like movement restrictions on the general population with the dramatic consequences of a generalized standstill on society. There are many unknowns which make this recovery phase and its strategy difficult to define. Indeed, the speed and extent of COVID-19 spread to attain pandemic status has challenged existing health management systems of many countries across the world. In Europe, for instance: Italy^2^, Spain^3^ and the UK^4^ have faced a true healthcare crisis which is still ongoing. Resolution is generally defined as the turning point when a healthcare system returns to routine operating standards of care. This includes not only emergency and intensive care unit (ICU)^2^ services but also the unwinding of surgery and diagnostic procedure backlogs. The process begins with a gradual re-introduction of a mobile population post lockdown which is based on selected criteria not including the risk of becoming severely ill or dying.

The rigorous data analysis presented in this study aims to support the rationale that decisions on managing the workforce should also be scientifically driven. Previous studies have investigated separately the effect of age^5–7^ or sex^8^ on COVID19, generally focusing on single country data. However, to the best of our knowledge, no study has yet considered comparison of the coupled effect of age-sex on COVID-19 severity in more than one country, and the investigation of appropriate data normalization by country’s age-sex population. Here, we address these points by analyzing existing data on COVID-19 severity in three European countries, and propose a risk-categorization of likeliness to develop a severe COVID-19 form which requires ICU or is indicative of a higher risk of dying. Our study aims to provide guidance to decision makers on elements which may support an evidence-based repopulation strategy and identify existing knowledge gaps which could be easily filled with more complete data, and that could be improved and accelerated by harmonized data collection and analysis. The method proposed by the present study has the advantage to use simple and repeatable variables which are available daily or weekly, thus allowing to monitor the effect of public health decisions in the short term, with the possibility to repeat these measures over time to evaluate promptly the epidemic dynamic and impact.

## Methods

### Data collection

Open access data are from: the Ministry of Health of Spain^9^; the Italian Superior Institute of Health^10^; the German Robert Koch Institute^11^. In particular, we considered these three countries because the structure of the open access data is to a certain extent similar and allows a comparison of variables stratified for age and sex, but with some limitations that we explain hereafter. Spain data includes age/sex stratified information for 4 variables: confirmed cases, hospitalizations, ICU and deaths. Italian data includes age/sex stratified information only 2 variables: confirmed cases and deaths. German data access was difficult, we were able to recover age/sex stratified information only for 1 variable: deaths. The age stratification for Spain and Italy is according to the following 10 age ranges: 0–9, 10–19, 20–29, 30–39, 40–49, 50–59, 60–69, 70–79, 80–89, 90 + . Whereas for Germany, the age stratification is coarser and includes the following age ranges: 59 −, 60–69, 70–79, 80–89, 90 +.

**Figure 1:**
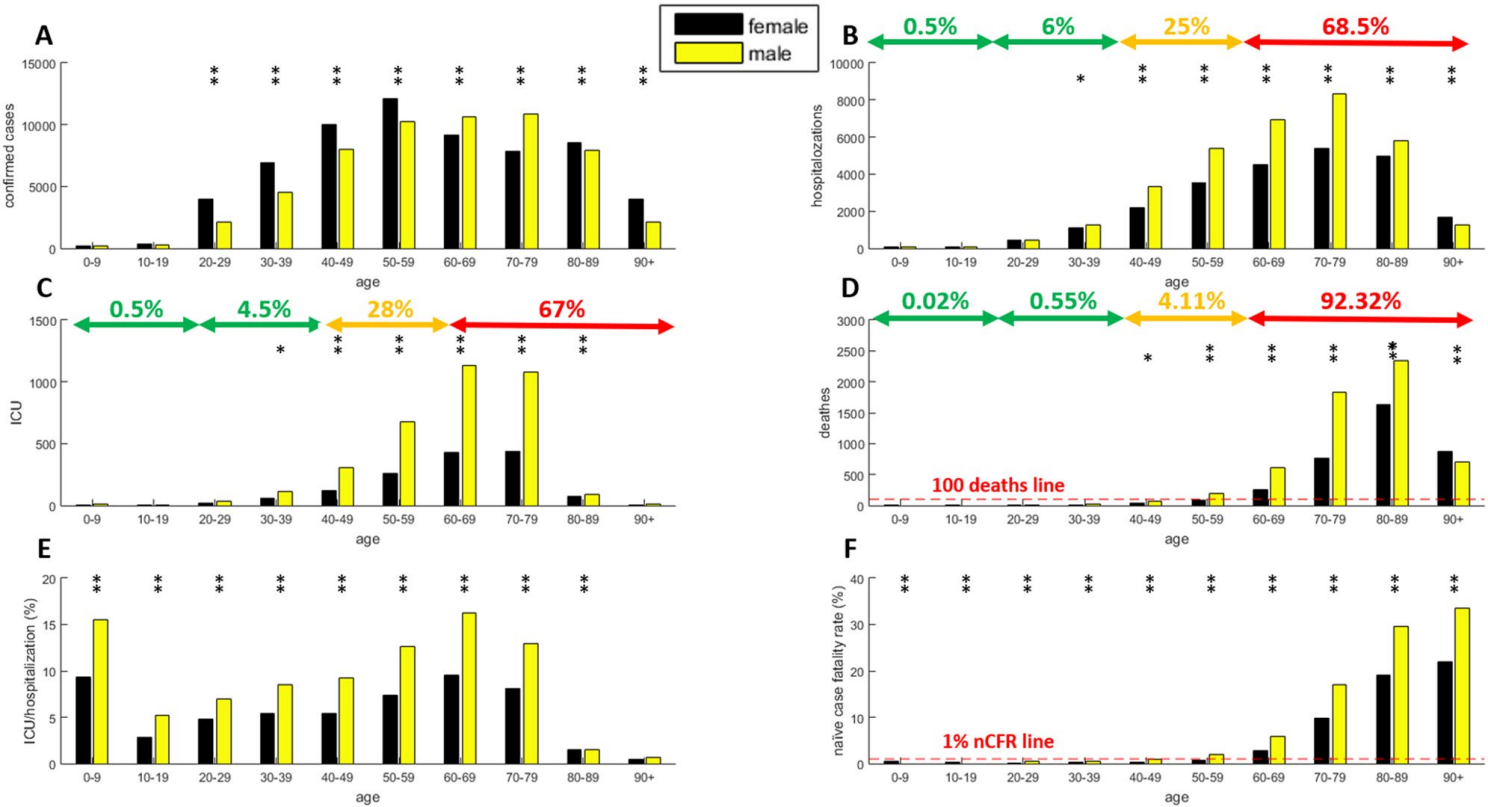
Data analysis of Spanish COVID-19 outbreak on April 10, 2020. **(A)** Confirmed cases, **(B)** hospitalizations, **(C)** ICU, **(D)** deaths, **(E)** ICU/hospitalization, **(F)** naïve case fatality rate. In each panel, the presence of at least one star (1 star indicates p-values below 0.05, 2 stars p-values below 0.001) on top of a specific age strata indicates that the sex inequality is statistically significant.

**Figure 2:**
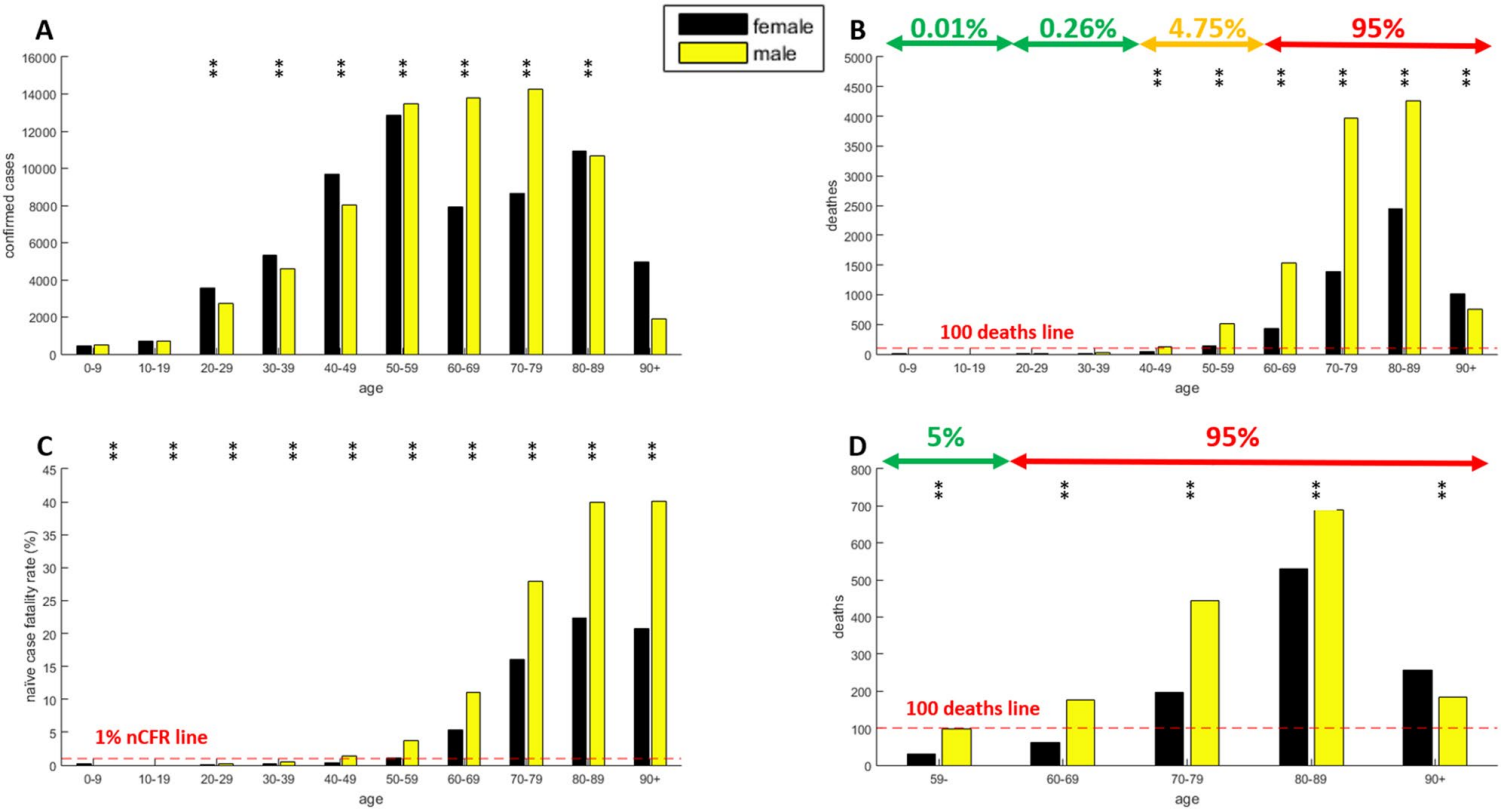
Data analysis of Italy and Germany COVID-19 outbreak on April 2020. **(A–C)** data of Italian outbreak on April 10, 2020. **(A)** Confirmed cases, **(B)** deaths, **(C)** naïve case fatality rate, **(D)** deaths data of German outbreak on April 12, 2020. The presence of at least one star (1 star indicates p-values below 0.05, 2 stars p-values below 0.001) on top of a specific age strata indicates that the sex inequality is statistically significant.

**Figure 3:**
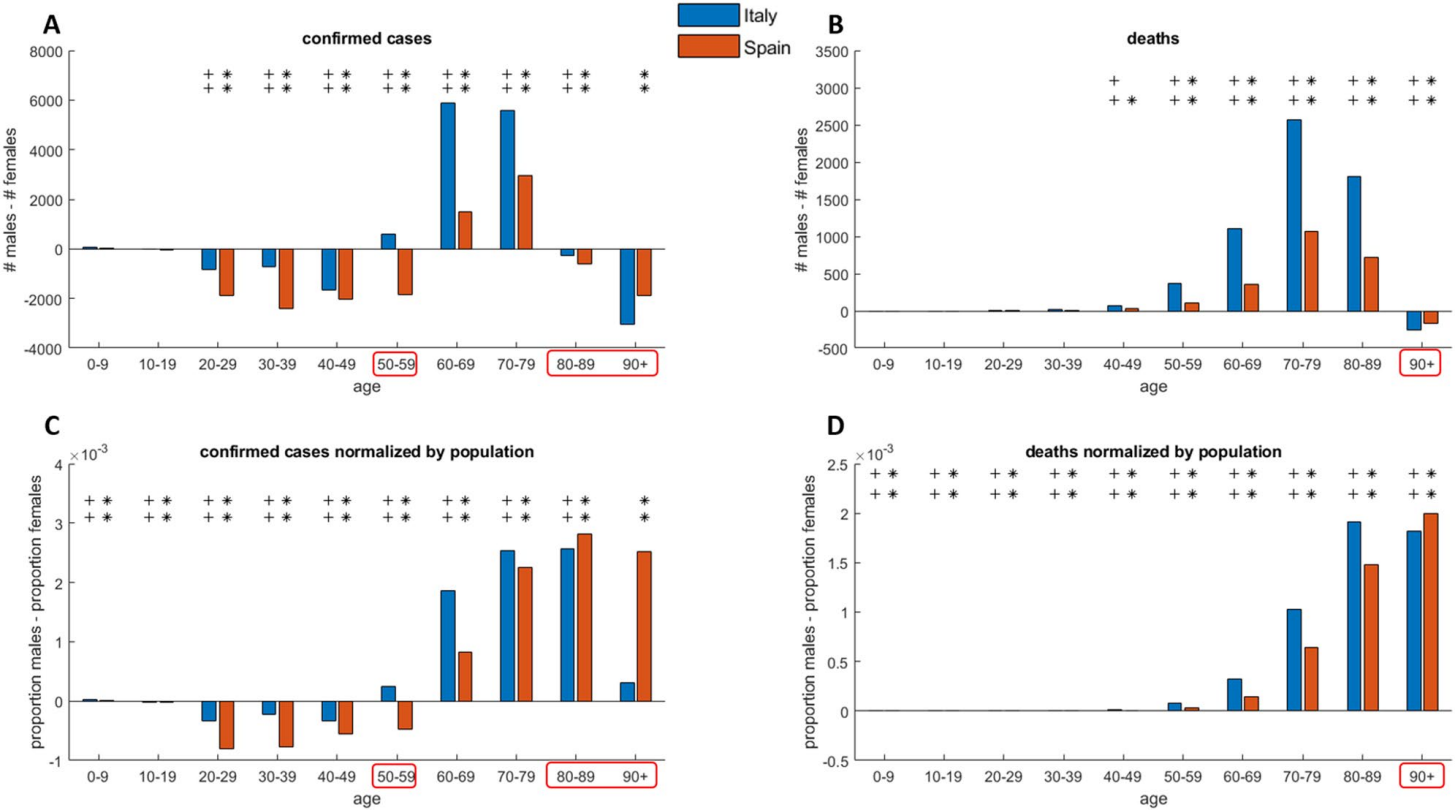
Data comparison of Spain and Italy COVID-19 outbreak for open access variables which are common to both national data sources. **(A)** Confirmed cases, **(B)** deaths, **(C)** confirmed cases normalized by national population, **(D)** deaths normalized by national population. The presence of at least one symbol (star for Spain and plus for Italy: 1 symbol indicates p-values below 0.05, 2 symbols p-values below 0.001) on top of a specific age strata indicates that the sex inequality is statistically significant. The red boxes on the age ranges on x-axis denote data that are not in agreement: either between the nations or between the variables before and after normalization adjustment. For all panels, the line on zero indicates the threshold over which, for the respective variable, the impact of COVID-19 on males is larger than on females.

For the first part of the study related with Figs. 1, 2 and 3, all data were considered in a similar time frame that is April 09–13, 2020. Specifically, as reported in Table 1: Spanish data refers to April 13, 2020 data report; Italian data refers to April 09, 2020 data report; German data refers to April 12, 2020 data report. The reasons to select this specific time frame are two. The first reason is to consider a chronological interval in which—independently from the precise initial point of the epidemics (that is unknown)—the data are fairly stabilized by the same constraint which is the ‘lockdown’ (a period in which great part of the population is confined at home to significantly reduce the epidemic spread). In other words, the data are considered in a ‘resting-state’ time point where unknown factors that influence the epidemics are minimized and mitigated in all countries by the same social intervention. The second reason is to emulate a real scenario in which different states in a continent (such as Europe) find themselves in the critical need to concurrently coordinate (on the basis of data analysis) a common guidance on effective outbreak-personalized social interventions for the second and subsequent phases following the easing of a lockdown.

**Table 1:** Data availability in the different time points considered in this study.

Country	First point (after lockdown stabilization) April 2020	Second point (end of first wave) May–June 2020	Third point (beginning of vaccination campaign) December 2020	Fourth point (one year from first time point) April 2021
Spain	April 13, 2020	May 29, 2020	–	–
Italy	April 09, 2020	June 3, 2020	December 29, 2020	April 14, 2020
Germany	April 12, 2020	–	–	–

Data for all time points were available only for Italy.

**Figure 4:**
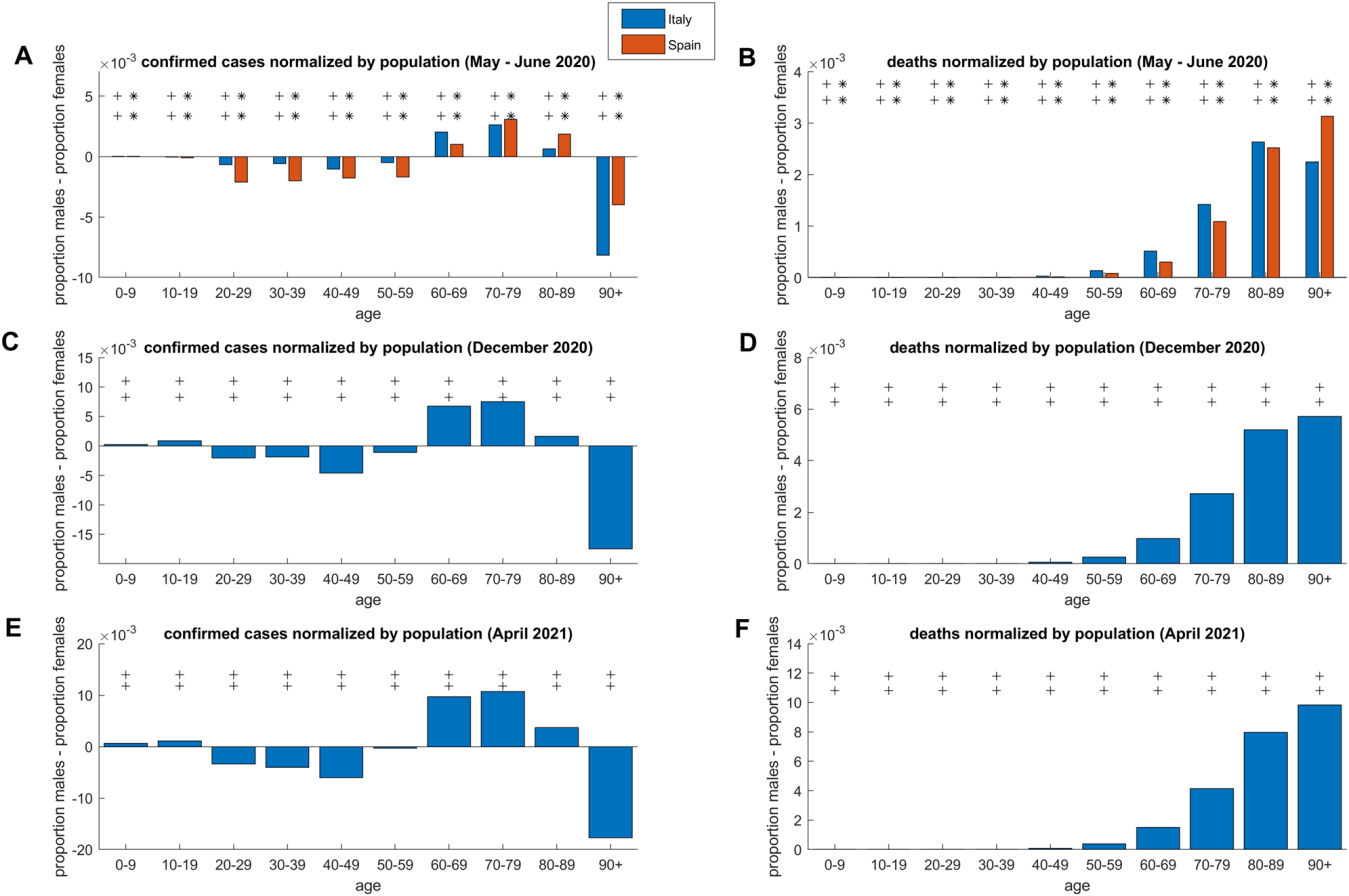
Data comparison of Spain and/or Italy COVID-19 outbreak for population normalized open access variables which are common to both national data sources in different time points from May 2020 to April 2021. **(A)** Confirmed cases normalized by national population in Spain (May 29, 2020) and Italy (June 3, 2020). **(B)** Deaths normalized by national population in Spain (May 29, 2020) and Italy (June 3, 2020). **(C)** Confirmed cases normalized by national population in Italy (December 29, 2020). **(D)** Deaths normalized by national population in Italy (December 29, 2020). **(E)** Confirmed cases normalized by national population in Italy (April 14, 2021). **(F)** Deaths normalized by national population in Italy (April 14, 2021). The presence of at least one symbol (star for Spain and plus for Italy: 1 symbol indicates p-values below 0.05, 2 symbols p-values below 0.001) on top of a specific age strata indicates that the sex inequality is statistically significant. For all panels, the line on zero indicates the threshold over which, for the respective variable, the impact of COVID-19 on males is larger than on females.

For the second part of the study, which aims to confirm the results of Fig. 3, and whose results are provided in Fig. 4, the three following time points were considered as reported in Table 1. The first time point is associated with the end of the first wave, and the data were available both for Spain (May 29, 2020) and Italy (June 3, 2020). The second time point is at the beginning of the vaccination in Italy (data from Spain and Germany were not anymore available in this time point). Officially in Italy the vaccination started on December 27, 2020, but the first available COVID-19 bulletin with the appropriate data was available on December 29, 2020. This does not represent a problem because the effect of vaccination campaign is not able to impact the data at this early stage. The third and final time point is on April 14, 2021 which is after around 1 year from the first time point (April, 2020) considered in this study, and the data were available only for Italy.

### Data availability

Table 1 reports the data availability in the different time points considered in this study. Data for all time points were available only for Italy. For each of the reported dates in Table 1, the data were extracted from the original COVID-19 bulletins of the respective countries, which are provided in the Supplementary Information.

### Data analysis

A Binomial test is applied to integer variables (confirmed cases, hospitalizations, ICUs, deaths), in order to assess whether the difference between female and male occurrence is significant at each age strata. To apply the test at each age strata, we consider the following setting: the sample size is N = # females + # males, in the selected age strata; the number of male extractions is k = # males, in the selected age strata; the probability of extracting a male is p = national level male proportion in the selected age strata.

A Pearson Chi-Square test for the inequality of two proportions is applied to measures derived as a ratio of integer variables (ICU/hospitalization, naïve case fatality rate, confirmed cases/national population 2019, deaths/national population 2019), in order to assess whether the difference between the female and male proportions is significant at each age strata.

For a certain variable (for instance, deaths or deaths/national population), a p-values is computed comparing the female and male values at each age strata by applying one of the statistical tests above. The p-value allows to assess whether the difference between female and male is significant at each age strata. However, in order to adjust for multiple hypothesis testing of the same variable at different age-strata, we apply a Bonferroni-correction, which is preferred because it is one the most conservative corrections.

Finally, in order to mark the strength of the finding, the visual representation of the level of p-value significance in the figures of this study is as follow: 1 star indicates p-values below 0.05 and 2 stars indicates p-values below 0.001.

## Results

Figure 1 reports the age and sex data for the Spanish COVID-19 outbreak as of April 13, 2020. The absolute number of confirmed cases in Fig. 1A shows that there are more female detected infections than male detected infections in the age intervals 20–59 and 80 + . However, this variable needs to be properly standardized compared to the sex distribution in the population by age class, as we discuss in Fig. 3. Further, this information is known to be incomplete given the unknown number of undetected, and the large portion of, asymptomatic cases^12^. The clinical peculiarities of COVID-19, the lack of harmonized testing protocols and total absence of serological surveillance^13^ are drivers that make currently available data on the number of confirmed infections difficult to use and to interpret.

Notwithstanding these hurdles, the goal of this study is to highlight strong evidences associated with COVID-19 severity changes for age-sex effect, with the aim of supporting guidance on useful outbreak-personalized social interventions for the subsequent phases following the easing of a lockdown. In this regard, the information that emerges from the number of hospitalizations (Fig. 1B) and number of ICU occupants (Fig. 1C) is the most relevant. Remuzzi et al.^2^ stressed the importance of finding immediate solutions for countries with reduced health system capacity (such as Italy or Spain) to effectively respond to the needs of patients who are infected and require intensive care. From our analysis, it emerges that in Spain approximatively 68% of hospitalizations (Fig. 1B) and 67% of ICU occupants (Fig. 1C) are associated with the age range 60 + and that, regardless of age, the healthcare demand in terms of capacity is higher for males than for females. Males seem to be at significantly (p < 0.001) higher risk of developing more severe COVID-19 infection after age 40 and at least up to age 80 given the unbalance in the number of hospitalizations and ICU admissions compared to the male distribution in the national population by age. It is notable that the number of deaths in the range 0–39 (Fig. 1D) is exceptionally low (0.57%) and without significant sex imbalance. In the range 40–59, deaths increase to approximately 4% of the overall total whilst in the 60 + age category, the death total climbs to around 92% of all deaths recorded. This trend is also confirmed by the naïve case fatality rate (Fig. 1F) that exceeds the 1% threshold in the 40 + age range category, although the sex inequality is significant in each age strata. Altogether, these results suggest that the 60 + age range is at higher risk than the rest of the population and hence may generate a greater healthcare demand, due mostly to the healthcare requirements of males, which are significantly higher than females. The age range 40–59 is moderately at risk of death but a significant (p < 0.05 for 40–49 and p < 0.001 for 50–59) sex discrepancy is confirmed also in this case. Interestingly, from the ICU/hospitalization rate (Fig. 1E), it emerges that although very few cases required hospitalization in the 0–9 age range category, those that did were more likely to require ICU compared to juvenile and middle age strata. In addition, the analysis of ICU/hospitalization rate (Fig. 1E) suggests that males require significantly (p < 0.001) higher ICU treatment regardless of age.

Figure 2 reports the age and sex analysis of all openly available data for the Italian COVID-19 outbreaks as of April 9, 2020 and the German outbreak as of April 12, 2020. The absolute number of confirmed cases in Italy (Fig. 2A) shows that apparently there are more female detected infection than male detected infection in age ranges 20–49 and 80–89, which agrees with the Spanish population. However, also here (as for Fig. 1A related to Spain data) this variable needs to be properly standardized compared to the sex distribution in the population by age class, as we discuss later in Fig. 3. The number of deaths in Italy (Fig. 2B) and Germany (Fig. 2D, the available open access data for Germany report only the age strata 59- as a unique range) confirms the age/sex trend and the percentages obtained for the Spanish population. The naïve case fatality rate for Italy (Fig. 2C) displays age-growing trend and sex imbalance similarly to the results in Spain (Fig. 1F).

In Fig. 3 we offer a sex imbalance (stratified by age) comparative analysis across countries for confirmed cases and deaths, which are the only two absolute variables available (open access) and equally stratified both for Spanish and Italian populations. German data are not harmonised and age-stratified in the same way of Italy and Spain, hence they cannot be included in this comparison. Figure 3A and Fig. 3C report the sex difference in confirmed cases before and after age strata national population adjustment (which is computed as the number of cases divided by the respective sex population number in that specific age strata). With reference to the difference without adjustment (absolute confirmed cases are reported in Fig. 3A), we could conclude that in the range 20–59 and 80 + the female detected infection is significantly higher (p < 0.001) than the male detected infections. However, part of this result is misleading. If the same difference is computed on the adjusted variables (Fig. 3C), it emerges that in the range 80–89, in both Spain and Italy, the male detected infection is significantly higher (p < 0.001) than the female equivalent, however in the range 90 + the same finding is not significant in the Italian data, and this suggests that more evidences should be provided in order to clarify the sex difference in the range 90 + . Figure 3B,D reports the sex difference in deaths respectively before and after age strata national population adjustment. In contrast to the findings in Fig. 3B (original values are used), when we evaluate the numbers adjusted in relation to the national population (Fig. 3D), it emerges that in reality the male deaths in the age range 90 + are significantly larger (p < 0.001) than the female ones (compare the range 90 + in Fig. 3A,C). These results in Fig. 3 emphasize the importance of performing statistical analyses between variables adjusted by national age-sex population and support that, both in Spain and Italy, 60 + is confirmed as a critical zone where confirmed infections (Fig. 3C) and deaths (Fig. 3D) in males are significantly higher than in females. Finally, for confirmation, we repeated the same analysis of Fig. 3C,D in other three time points which are reported in Table1, and the results of this confirmation analysis are provided in Fig. 4. We conclude that the results in Fig. 3C,D that are related with the first time point on April 2020 (after lockdown stabilization period), are generally confirmed: in Fig. 4A,B at the end of the first wave (May–June 2020) for Spain and Italy; in Fig. 4C,D at the beginning of vaccination campaign in Italy; in Fig. 4E,F after one year from the first time point considered in this study. Furthermore, the results in Fig. 4A,C,E offer evidences according to which the sex difference in the range 90 + (which was uncertain in Fig. 3C) for confirmed cases normalized by national population is significantly higher (p < 0.001) in females than in males.

## Discussion

Previous studies have addressed the effect of age on hospitalization and mortality. In the current study, we seek to develop these arguments further: we have investigated the synergic effect of age and sex on COVID-19 severity and, on this basis, we propose considerations that support age-adaptive and sex-balanced social interventions to ‘navigate’ successfully towards the next phases of the COVID-19 pandemic.

At the beginning of the COVID-19 outbreak in China, specific knowledge in relation to the nature of the pathogen, the rate of spread through populations and the clinical disease progression was lacking. Therefore, a  generalized stay-home (lockdown suppression) strategy was the only applicable solution available to authorities. This method was applied by several other countries and has shown to be determinant in mitigating the viral spread. Although we still lack data^12^ on many aspects of COVID-19, two clear facts have emerged from the first year of the pandemic data.

The first fact is that data from Spain, Italy and Germany underpin that COVID-19 severity has a remarkable growth with the age. Thus, we propose that social interventions should adapt and differentiate with age in order to optimize their efficiency. This means that, for instance, if indicators of epidemic severity (such as the naïve case fatality ratio) increase with age, then age categories at higher risk should be protected with ad-hoc social interventions that might be different from the ones of other age categories at lower risk. This implies that strategies based on recurring generalized lockdown social interventions are not only badly conceptualized but also inefficient (inappropriate from a cost-benefit point of view), because they constrain all individuals regardless of their risk of becoming affected by a severe form of COVID-19. Generalized lockdown social interventions primarily aim to contain the contagion process, neglecting other important factors that keep a society healthy. Instead, we suggest to apply precision-based social interventions that aim to contain the* impact* of a contagion process by focusing on the individuals at risk of a severe form of disease.

The second fact is that COVID-19 severity is significantly higher in males than females in the analyzed data. This difference is particularly large in the age range 40 + , and a sex-balanced repopulation after lockdown should be encouraged.

The design of new COVID-19-personalized social intervention strategies that utilize the learnings from these and other data, such as relevant comorbidities, may contribute to saving human lives, reducing healthcare costs and facilitating a more rapid return to economic sustainability. This age-adaptive and sex-balanced strategy could be better tuned by comparison of data from existing and ongoing studies in other countries. Unfortunately, the open access data currently available from most countries are inadequate for this type of study. Data on hospitalizations and ICU admissions (which are crucial in data analysis for COVID-19 decision making^2^) are not easily accessible. In order to gain the most robust evidence and develop accurate guidelines to support policy makers, it is imperative that the scientific community is able to rely on harmonized and interoperable open access data. Nevertheless, in emergency scenarios with reduced data availability, the medical and scientific communities should use all data potential to rapidly move from generic social intervention strategies towards outbreak-tailored approaches. In our study we find that a common sex-age COVID-19 trend is shared between Spain and Italy, and 60 + males show significantly higher detected infection and death risk than females. Hence, males in the age range 60 + should be informed of this risk and empowered to protect their health through tailored public intervention strategies. Ideally, in the era of personalized healthcare, future outbreak precision strategies should act in a coordinated fashion and on different levels, by focusing on the risk factors which are associated with the host (for instance, age and sex in COVID-19), by integrating different large-scale social interventions with medium-scale active surveillance^14^ and small-scale contact tracing^15^.

Yet, there are some limitations in our investigation that can be considered to improve next studies. The analysis of only three countries may constitute a weakness considering the pandemic nature of COVID-19, which of course includes the emergence of variants that may have unknown characteristics. Hopefully, this void will be solved in the future, when more open access and harmonized data will be available. To this regard we stress the importance to collect data and design studies that take into consideration—and eventually correct for—additional driving factors than age and sex. For instance, the impact of the number of elderly people hospitalized into retirement homes in different countries might be an interesting investigation to pursue. The molecular mechanisms associated to age and sex in COVID-19 severity should be investigated further, as early scientific findings are provided in biomedical studies of network medicine from Guzzi et al. ^16^ and Belyaeva et al. ^17^. We know also that the human microbiome is significantly modified by age and sex ^18^, and the investigation of bacteria-metabolite networks ^19^ in COVID-19 age/sex-associated studies could represent an interesting direction to follow.

In this study we conclude that both age and sex are variables that influence the clinical outcomes of COVID-19. Individuals in the 0–40 age ranges and females under 60 are significantly less likely to develop a severe condition and die, whereas males equal or above 60 are more likely at risk of severe disease and death. This is reflected in ICU admissions and therefore in greater healthcare costs. Thus, implementing an age-adaptive and sex-balanced repopulation protocol would seem a unique public health empowerment opportunity. This should be coupled with a graded shielding protocol for other categories at risk, including children with underlying conditions. We introduce a method that is useful to describe, monitor and compare COVID-19 disease main characteristics and the impact of preventive and care interventions. Although the method is less useful for providing guidance to repopulation and identifying knowledge gaps, it can be considered a starting point: an analytical approach that could be used as foundation for the development of methods that can compute the repopulation values. Indeed, this conceptual framework developed comparing data from three European countries could then be tailored to each country on the basis of its demographics, economic needs and general health condition to establish general guidelines for stakeholders to follow, as they face the challenges of responding to evolving pandemic scenarios.

## Supplementary Information

Supplementary Information.
