# Supplementary material for: Age-sex population adjusted analysis of disease severity in epidemics as a tool to devise public health policies for COVID-19
Source: Sci Rep. 2021 Jun 3;11:11787. doi: 10.1038/s41598-021-89615-4 (PMC8175671; doi:10.1038/s41598-021-89615-4)
Supplement: Supplementary file 1 — Supplementary Information. [file 41598_2021_89615_MOESM1_ESM.pdf]

2020

Epidemia COVID-19

Aggiornamento nazionale

9 aprile 2020 – ore 16:00

DATA PUBBLICAZIONE: 10 APRILE 2020

Prodotto dall'Istituto Superiore di Sanità (ISS), Roma

A cura di: Flavia Riccardo, Xanthi Andrianou, Antonino Bella, Martina Del Manso, Alberto Mateo Urdiales, Massimo Fabiani, Stefania Bellino, Stefano Boros, Fortunato (Paolo) D'Ancona, Maria Cristina Rota, Antonietta Filia, Ornella Punzo, Andrea Siddu, Corrado Di Benedetto, Marco Tallon, Alessandra Ciervo, Maria Rita Castrucci, Patrizio Pezzotti, Paola Stefanelli, Giovanni Rezza, per ISS,

e di: Manuela Di Giacomo (Abruzzo); Michele La Bianca (Basilicata); Anna Domenica Mignuoli (Calabria); Angelo D'Argenzio (Campania); Erika Massimiliani (Emilia-Romagna); Tolinda Gallo (Friuli Venezia Giulia); Paola Scognamiglio (Lazio); Camilla Sticchi (Liguria); Danilo Cereda (Lombardia); Daniel Fiacchini (Marche); Francesco Sforza (Molise); Maria Grazia Zuccaro (P.A. Bolzano); Pier Paolo Benetollo (P.A. Trento); Daniela Tiberti (Piemonte); Maria Chironna (Puglia); Maria Antonietta Palmas (Sardegna); Salvatore Scondotto (Sicilia); Emanuela Balocchini (Toscana); Anna Tosti (Umbria); Mauro Ruffier (Valle D'Aosta); Filippo Da Re (Veneto).

Citare il documento come segue: Task force COVID-19 del Dipartimento Malattie Infettive e Servizio di Informatica, Istituto Superiore di Sanità. Epidemia COVID-19, Aggiornamento nazionale: 9 aprile 2020

EPIDEMIA COVID-19

Aggiornamento nazionale

9 aprile 2020 – ore 16:00

Nota di lettura: *Questo bollettino è prodotto dall'Istituto Superiore di Sanità (ISS) ed integra dati microbiologici ed epidemiologici forniti dalle Regioni e dal Laboratorio Nazionale di Riferimento per SARS-CoV-2 dell'ISS. I dati vengono raccolti attraverso una piattaforma web dedicata ed include tutti i casi di COVID-19 diagnosticati dai laboratori di riferimento regionali. I dati vengono aggiornati giornalmente da ciascuna Regione anche se alcune informazioni possono richiedere qualche giorno per il loro inserimento. Per questo motivo, potrebbe non esserci una completa concordanza con quanto riportato attraverso il flusso informativo della Protezione Civile e del Ministero della Salute che riportano dati aggregati.*

I dati raccolti sono in continua fase di consolidamento e, come prevedibile in una situazione emergenziale, alcune informazioni sono incomplete. In particolare, si segnala, soprattutto nelle Regioni in cui si sta verificando una trasmissione locale sostenuta del virus, la possibilità di un ritardo di alcuni giorni tra il momento della esecuzione del tampone per la diagnosi e la segnalazione sulla piattaforma dedicata. Pertanto, la diminuzione dei casi che si osserva negli ultimi due giorni (Figura 1), deve essere al momento interpretata come un ritardo di notifica e non come descrittiva dell'andamento dell'epidemia.

Il bollettino descrive, con grafici, mappe e tabelle la diffusione, nel tempo e nello spazio, dell'epidemia di COVID-19 in Italia. Fornisce, inoltre, una descrizione delle caratteristiche delle persone affette.

In evidenza

	9 aprile 2020	2 aprile 2020	Evoluzione settimanale	
**Casi**	136.110	106.399	+29.771	↑ 27,9%
**Deceduti**	16.654	12.550	+4.104	↑ 32,7%
**Operatori sanitari**	14.066	10.657	+3.409	↑ 32,0%
**In terapia intensiva**	3.609	2.779	+830	↑ 29,9%

Regioni/PA con maggiore o minore incremento rispetto al bollettino del 2 aprile 2020			
**Maggior incremento**	Campania	Basilicata	Valle d'Aosta
**Minor incremento**	Marche	Emilia-Romagna	Lombardia

La situazione nazionale

- Dall'inizio dell'epidemia alle ore 16 del 9 aprile 2020, sono stati riportati al sistema di sorveglianza 136.110 casi di COVID-19 diagnosticati dai laboratori di riferimento regionale come positivi per SARS-CoV–2 (29.711 casi in più rispetto al precedente bollettino riferito al 2 aprile 2020). È stata confermata la diagnosi di infezione da SARS-CoV-2 nel 99% dei campioni inviati dai laboratori di riferimento regionale e processati dal laboratorio nazionale di riferimento (ISS). Sono stati notificati 16.654 decessi (4.104 decessi in più rispetto al bollettino del 2 aprile 2020).

- La Figura 1 mostra l'andamento del numero di casi di COVID 19 segnalati per data di prelievo/diagnosi (disponibile per 131.995/136.110 casi). La curva epidemica mostra un andamento in crescita delle nuove diagnosi fino al 20 marzo 2020 e successivamente a tale data un decremento lieve ma costante che dovrà essere confermato nei prossimi giorni, quando i dati saranno consolidati.

- La data di inizio sintomi è al momento disponibile solo in 77.927 dei 136.110 casi segnalati. Questo può essere dovuto al fatto che una parte dei casi diagnosticati non ha ancora sviluppato sintomi e/o dal mancato consolidamento del dato stesso. La Figura 2 mostra la distribuzione dei casi per data inizio dei sintomi, che evidenzia come i primi casi sintomatici risalgano alla fine di gennaio, con un andamento in crescita del numero di casi fino al 13 marzo 2020. Anche in questo caso il picco osservato non tiene conto sia del ritardo della segnalazione che dei casi che potrebbero aver sviluppato i sintomi dopo tale data.

- Il tempo mediano trascorso tra la data di insorgenza dei primi sintomi e la data di diagnosi è di 3 giorni per il periodo 20-29 febbraio (calcolato su 1.614 casi), di 5 giorni per il periodo 1-20 marzo (37.722 casi), di 6 giorni dal 21 marzo al 9 aprile (34.379 casi).

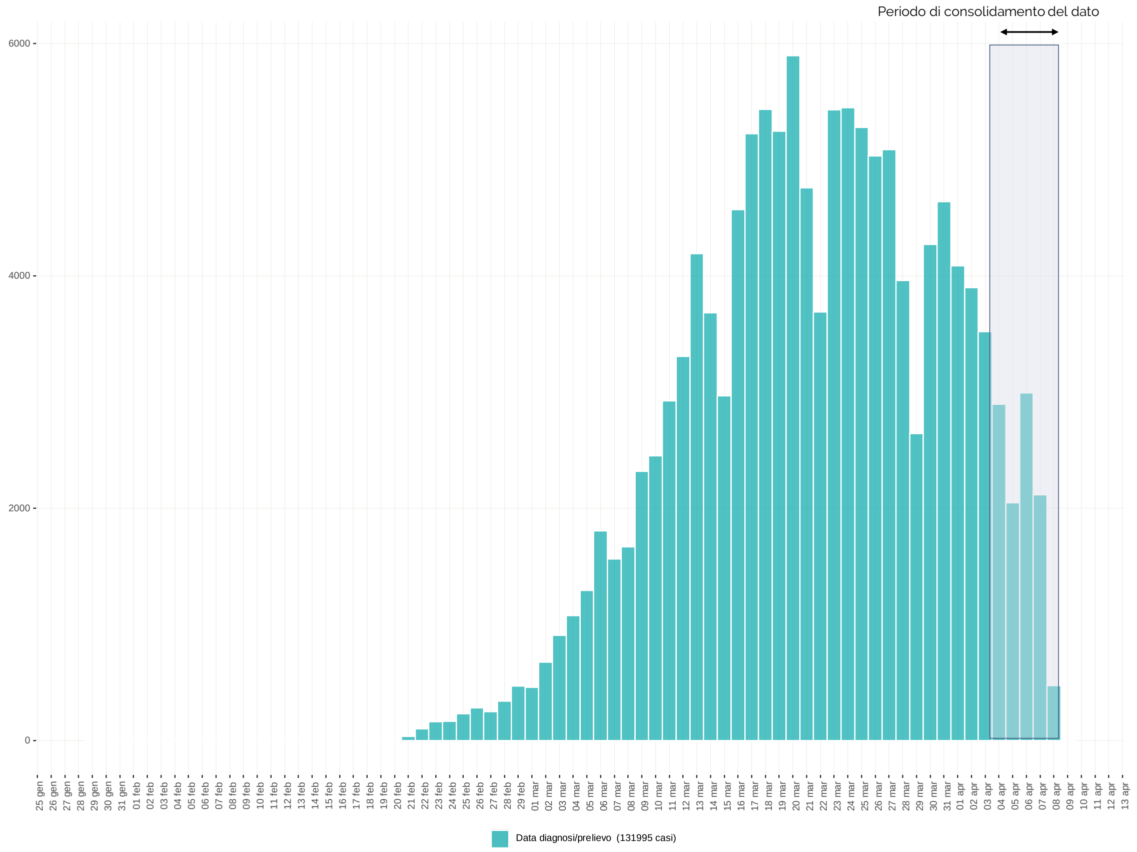

FIGURA 1 – CASI DI COVID-19 DIAGNOSTICATI DAI LABORATORI DI RIFERIMENTO REGIONALE, PER DATA PRELIEVO/DIAGNOSI (N=131.995).

Nota I dati più recenti devono essere considerati provvisori (vedere soprattutto riquadro grigio)

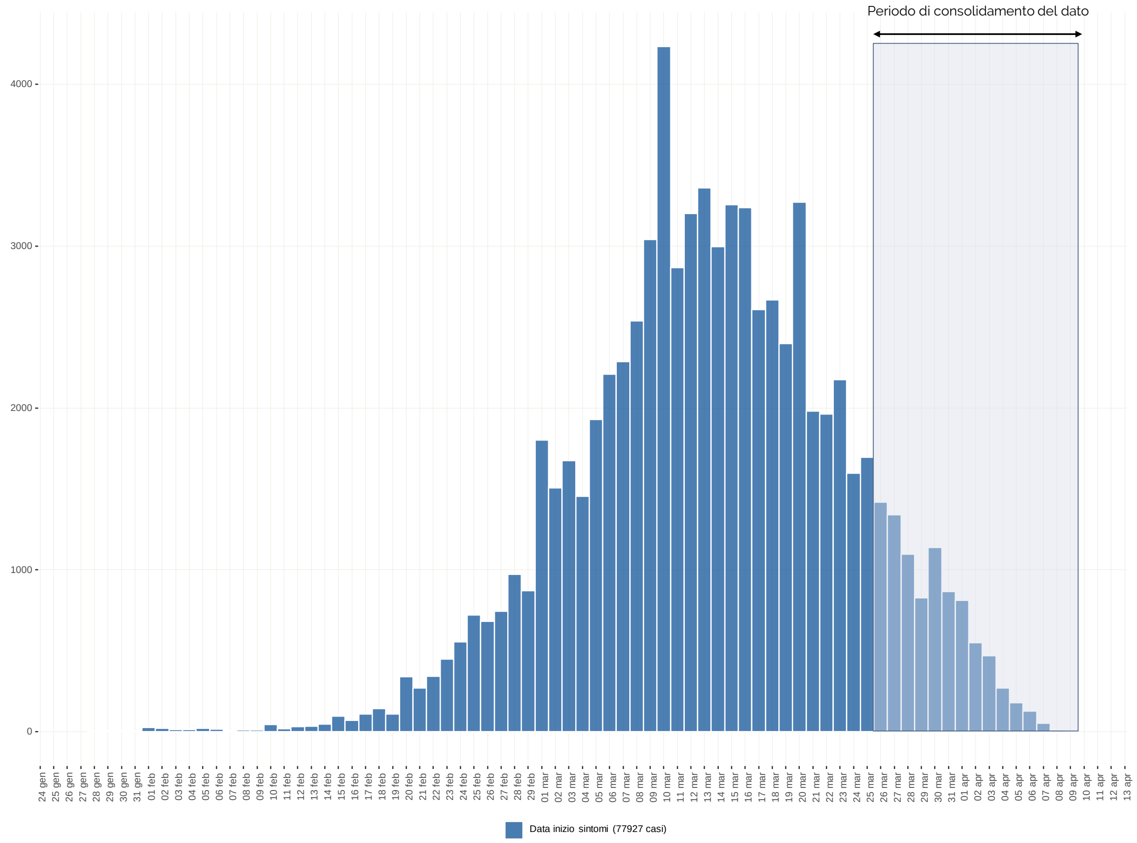

FIGURA 2 – CASI DI COVID-19 DIAGNOSTICATI DAI LABORATORI DI RIFERIMENTO REGIONALE, PER DATA INIZIO SINTOMI (N=77.927).

Nota: i dati più recenti devono essere considerati provvisori sia per il ritardo di notifica dei casi più recenti sia perché casi non ancora diagnosticati riporteranno in parte la data di inizio sintomi nei giorni del riquadro grigio.

- 70.726 casi sono di sesso maschile (52,1%). L'età mediana è di 62 anni (Range 0-100).

- La Tabella 1 mostra la distribuzione dei casi e dei decessi segnalati per sesso e fasce di età decennali. L'informazione sul sesso è nota per 135.813/136.110 casi. Nelle fasce di età 20-29, 30-39, 40-49 anni, e >90 anni si osserva un numero maggiore di casi di sesso femminile rispetto al numero di casi di sesso maschile. Nella fascia di età >90 anni, il numero di soggetti di sesso femminile è addirittura oltre il doppio quello di soggetti di sesso maschile probabilmente per la struttura demografica della popolazione. In tutte le altre fasce d'età esaminate, si osserva un maggior numero di casi di sesso maschile rispetto a casi di sesso femminile.

- Nella Tabella 1 si osserva un aumento della letalità con l'aumentare dell'età dei casi. La letalità è più elevata in soggetti di sesso maschile in tutte le fasce di età, tranne che per la fascia di età >90 anni.

- Nel 31,4% dei casi segnalati viene riportata almeno una co-morbidità (patologie cardiovascolari, patologie respiratorie, diabete, deficit immunitari, patologie

metaboliche, patologie oncologiche, obesità, patologie renali o altre patologie croniche).

- Le indagini epidemiologiche effettuate suggeriscono che nella quasi totalità dei casi l'infezione è stata acquisita in Italia (tutti i casi ad eccezione dei primi tre casi segnalati dalla regione Lazio che si sono verosimilmente infettati in Cina).

- La gravità clinica dei pazienti non è riportata in tutte le Regioni/PPAA in modo standardizzato secondo le modalità previste dalla sorveglianza COVID-19, ma si sta procedendo alla raccolta di tale informazione. Attualmente la gravità clinica è disponibile per 50.754 casi, di cui 4.666 (9,2%) asintomatici, 8.463 (16,7%) pauci-sintomatici, 7.534 (14,8%) con sintomi per cui non è specificato il livello di gravità, 19.285 (38,0%) con sintomi lievi, 9.467 (18,7%) con sintomi severi tali da richiedere ospedalizzazione, 1.339 (2,6%) con quadro clinico di gravità critica che richiede ricovero in Terapia Intensiva.

TABELLA 1 - DISTRIBUZIONE DEI CASI DIAGNOSTICATI DAI LABORATORI DI RIFERIMENTO REGIONALE (N=136.110) E DEI DECESSI SEGNALATI (N=16.654) PER FASCIA DI ETÀ E SESSO

Classe di Età	Soggetti di sesso maschile					Soggetti di sesso femminile					Casi totali				
	N. Casi	% Casi per sesso	N. Deceduti	% Deceduti per sesso	% Letalità	N. Casi	% Casi per sesso	N. Deceduti	% Deceduti per sesso	% Letalità	N. Casi	% Casi per classe di età	N. Deceduti	% Deceduti per classe di età	% Letalità
0-9	509	54,5	0	0,0	0,0	425	45,5	1	100,0	0,2	938	0,7	1	0,0	0,1
10-19	717	50,2	0	0,0	0,0	712	49,8	0	0,0	0,0	1.432	1,1	0	0,0	0,0
20-29	2.752	43,5	5	71,4	0,2	3.576	56,5	2	28,6	0,1	6.360	4,7	7	0,0	0,1
30-39	4.612	46,5	25	69,4	0,5	5.316	53,5	11	30,6	0,2	9.956	7,3	36	0,2	0,4
40-49	8.021	45,3	114	74,5	1,4	9.676	54,7	39	25,5	0,4	17.745	13,0	153	0,9	0,9
50-59	13.469	51,1	504	79,1	3,7	12.864	48,9	133	20,9	1,0	26.391	19,4	638	3,8	2,4
60-69	13.784	63,5	1.532	78,4	11,1	7.908	36,5	423	21,6	5,3	21.734	16,0	1.957	11,8	9,0
70-79	14.234	62,2	3.968	74,0	27,9	8.666	37,8	1.393	26,0	16,1	22.934	16,8	5.366	32,2	23,4
80-89	10.661	49,4	4.255	63,5	39,9	10.935	50,6	2.450	36,5	22,4	21.636	15,9	6.711	40,3	31,0
>90	1.894	27,7	760	42,6	40,1	4.941	72,3	1.022	57,4	20,7	6.842	5,0	1.784	10,7	26,1
Non noto	73	51,8	1	100,0	1,4	68	48,2	0	0,0	0,0	142	0,1	1	0,0	0,7
**Totale**	70.726		11.164		15,8	65.087		5.474		8,4	136.110		16.654		12,2

NOTA: LA TABELLA NON INCLUDE I CASI CON SESSO NON NOTO

- L'informazione sul luogo di trattamento è disponibile per 95.250 casi (il 70% dei casi totali), di cui risultano ospedalizzati 19.375 casi (20,3%). Di questi ultimi, 3.609 (22,8%) sono in terapia intensiva. Attualmente, questo dato non è classificato in tutte le Regioni/PPAA in modo standardizzato, secondo le modalità previste dalla sorveglianza COVID-19, ma si sta procedendo alla raccolta di tale informazione. Pertanto, i dati sullo stato clinico e sul reparto di degenza saranno soggetti a modifiche dovute al loro progressivo consolidamento.

- La Figura 3 mostra i dati cumulativi, riportati dal Ministero della Salute e dalla Protezione Civile al 6 aprile 2020, sulla condizione di ricovero e isolamento e sugli esiti dei casi di COVID-19 diagnosticati dai laboratori di riferimento regionale. Si sottolinea che il numero di casi riportato in Figura 3 è un numero aggregato riportato giornalmente dalle regioni attraverso il flusso informativo della Protezione Civile e del Ministero della Salute e, pertanto, differisce dal dato individuale (soggetto a un maggior ritardo di notifica) riportato nella piattaforma della sorveglianza Covid-19.

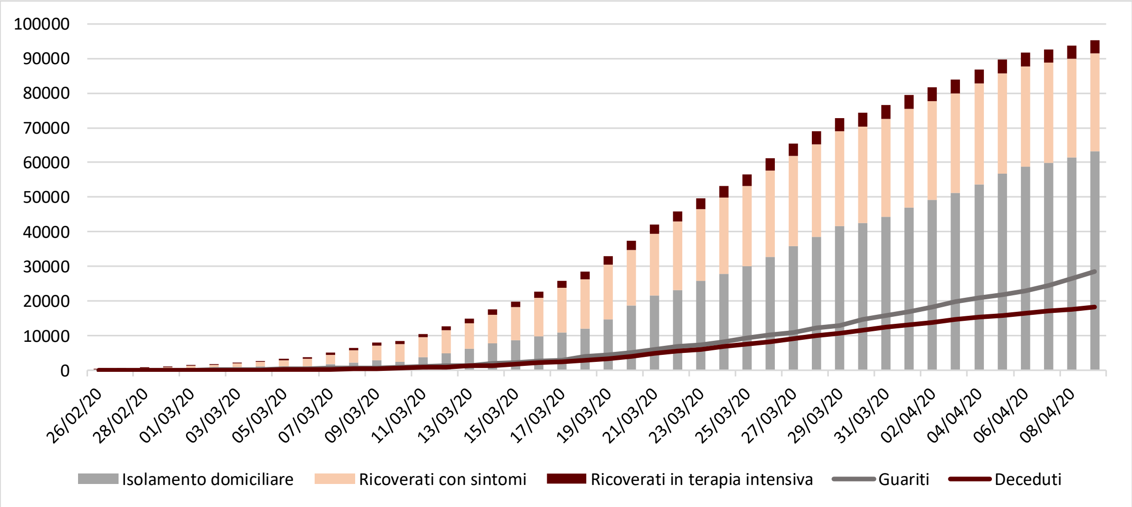

Figura 3 – Numero cumulativo di casi di COVID-19 diagnosticati dai laboratori di riferimento regionale, per stato di ricovero/isolamento ed esito (N=143.626) al 09/04/2020

FONTE DATI MINISTERO DELLA SALUTE E PROTEZIONE CIVILE

- La Figura 4 e la Tabella 2 mostrano l'incidenza e la distribuzione dei casi segnalati per Regione/PA. Al 9 aprile 2020, tutte le province italiane hanno segnalato almeno un caso di COVID-19. I casi si concentrano soprattutto nel nord Italia, in particolare in Lombardia, Emilia-Romagna, Veneto e Piemonte. Molti casi sono riportati anche da Toscana, Marche, Lazio, P.A. Trento e Campania; in ognuna di queste regioni il numero totale di casi di COVID-19 segnalati al sistema di sorveglianza supera i 3.000. Altre 7 Regioni/PPAA hanno riportato ognuna oltre 1.000 casi di infezione. Le rimanenti cinque regioni (Basilicata, Calabria, Molise, Sardegna, Valle d'Aosta) hanno riportato ognuna un numero di casi inferiore a

1.000, indicando una trasmissione più limitata.

- La Figura 4 mostra i dati di incidenza (per 100.000 abitanti) dei casi di COVID-19 diagnosticati dai laboratori di riferimento regionale (n=136.110) e il numero di casi diagnosticati negli ultimi 7 giorni (n=14.047), per regione/P.A. di diagnosi. Si sottolinea che in alcune regioni/PPAA che riportano un numero meno consistente di casi rispetto ad altre, l'incidenza cumulativa (numero di casi totali segnalati/popolazione residente) è tuttavia particolarmente elevata (PA Trento, PA Bolzano, Valle d'Aosta), con valori simili a quelli riportati dalle Regioni Emilia-Romagna, Lombardia e Marche.

- La Figura 5 confronta i dati di incidenza cumulativa per provincia di domicilio/residenza, raccolti dall'ISS con quelli raccolti dal Ministero della Salute/Protezione Civile (dati aggregati). Si può osservare che, sebbene l'incidenza stimata con i dati raccolti dal Ministero della Salute/Protezione civile sia più elevata perché o meno soggetta a ritardo di notifica, le due mappe mostrano quadri simili relativamente alle aree di diffusione dell'infezione.

- La Tabella 3 riporta la distribuzione per fascia di età e sesso dei casi con un'età <18 anni. Complessivamente i casi diagnosticati in questa fascia di età sono circa l'1% del totale. Tra essi il 19,7% ha un'età inferiore ai 2 anni; il 63,7% ha una età >6 anni. La Tabella 4 riporta, per i casi per cui l'informazione è disponibile (1.466/1.966=74,5%), il dato sul luogo di trattamento. Complessivamente sono ospedalizzati circa il 6,9% dei casi <18 anni. Come atteso, la percentuale è maggiore tra i casi con età <2 anni.

FIGURA 4 – INCIDENZA (PER 100.000 ABITANTI) DEI CASI DI COVID-19 DIAGNOSTICATI DAI LABORATORI DI RIFERIMENTO REGIONALE (N=136.110) E NUMERO DI CASI DIAGNOSTICATI NEGLI ULTIMI 7 GIORNI (N=14.047), PER REGIONE/PA DI DIAGNOSI

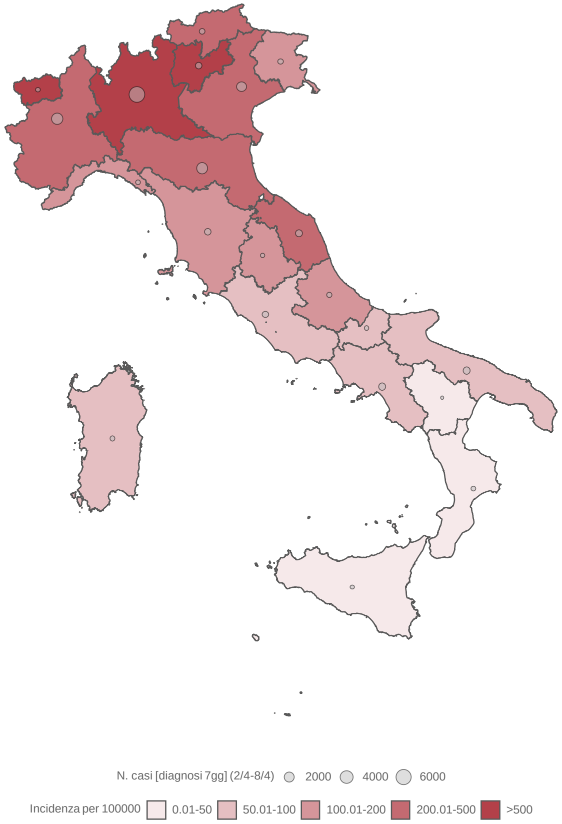

TABELLA 2- DISTRIBUZIONE DEI CASI DIAGNOSTICATI DAI LABORATORI DI RIFERIMENTO REGIONALE, PER REGIONE/PA DI DIAGNOSI (N=136.110)

Regione/PPAA	Casi	% su totale	Incidenza cumulativa per 100.000
Lombardia	54.801	40,3	544,71
Emilia-Romagna	18.188	13,4	407,85
Veneto	12.940	9,5	263,77
Piemonte	12.392	9,1	284,45
Toscana	5.219	3,8	139,93
Marche	4.755	3,5	311,75
Lazio	4.238	3,1	72,09
P.A. Trento	3.326	2,4	614,68
Campania	3.002	2,2	51,74
Liguria	2.899	2,1	186,96
Puglia	2.714	2,0	67,36
Friuli-Venezia Giulia	1.987	1,5	163,51
P.A. Bolzano	1.868	1,4	351,67
Abruzzo	1.862	1,4	141,97
Sicilia	1.782	1,3	35,64
Umbria	1.194	0,9	135,37
Sardegna	903	0,7	55,07
Valle d'Aosta	874	0,6	695,49
Calabria	785	0,6	40,32
Molise	232	0,2	75,91
Basilicata	149	0,1	26,47

FIGURA 5 – INCIDENZA CUMULATIVA (PER 100.000 ABITANTI) DI COVID-19 PER PROVINCIA; CONFRONTO FONTE DATI ISTITUTO SUPERIORE DI SANITÀ (ISS) E MINISTERO DELLA SALUTE (MDS) E PROTEZIONE CIVILE

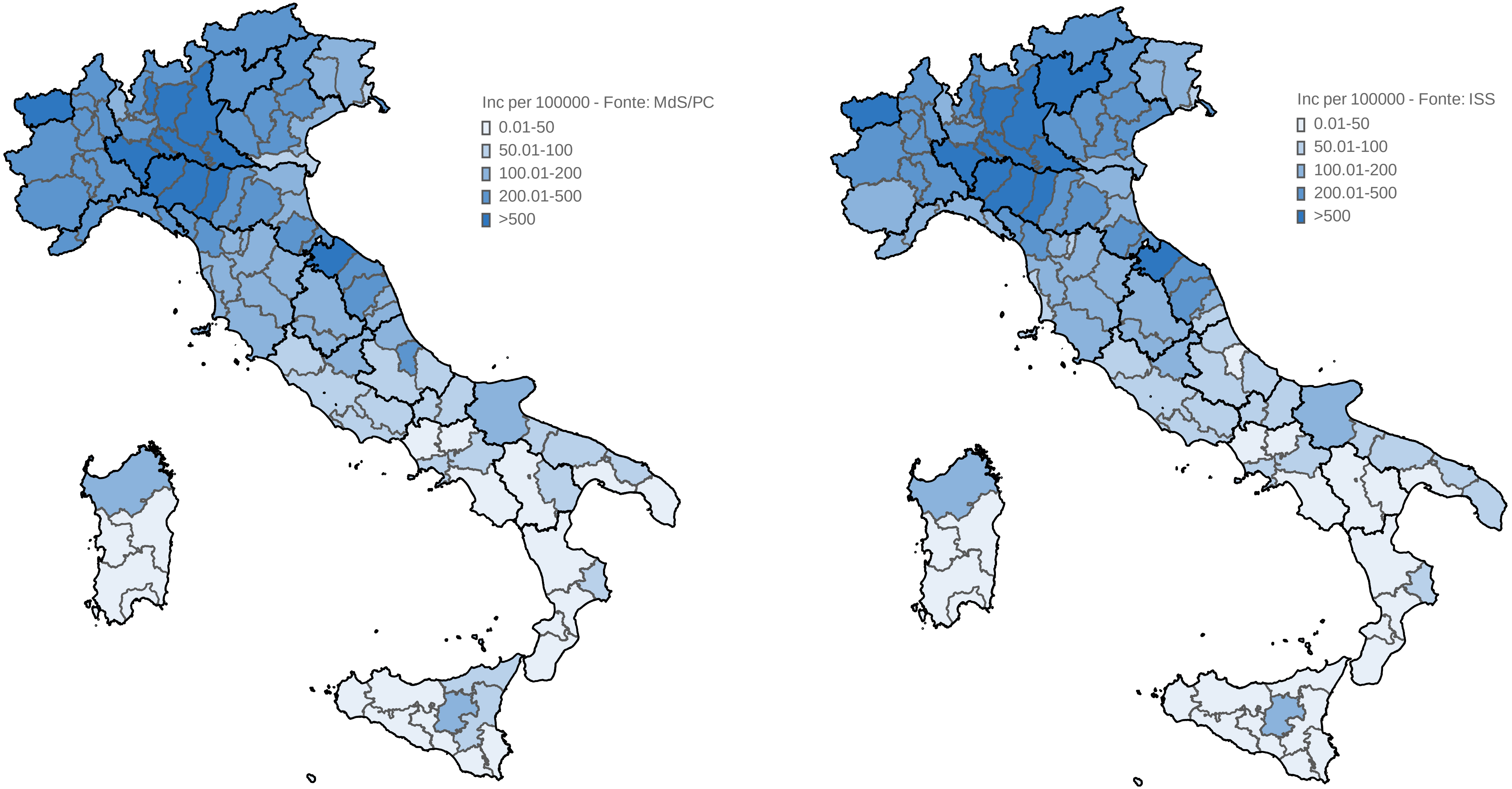

FIGURA 5 – INCIDENZA CUMULATIVA (PER 100.000 ABITANTI) DI COVID-19 PER PROVINCIA; CONFRONTO FONTE DATI ISTITUTO SUPERIORE DI SANITÀ (ISS) E MINISTERO DELLA SALUTE (MDS) E PROTEZIONE CIVILE

TABELLA 3- DISTRIBUZIONE DEI CASI CON ETÀ <18 ANNI, PER CLASSE DI ETÀ (N=1.966)

Classe di età (anni)	N. casi	%	Femmine	Maschi	% Femmine	% Maschi
0-1	387	19,7	170	213	44,4	55,6
2-6	326	16,6	141	185	43,3	56,7
7-17	1.253	63,7	633	618	50,6	49,4
**<18 anni**	1.966		944	1.016	48,2	51,8

NOTA: LA TABELLA NON INCLUDE I CASI CON SESSO NON NOTO

TABELLA 4- DISTRIBUZIONE DEI CASI CON ETÀ <18 ANNI PER CLASSE DI ETÀ (N=1.966) E LUOGO DI TRATTAMENTO

Classe di età (anni)	N. casi a domicilio	N. casi ospedalizzati	% ospedalizzati per classe di età	% ospedalizzati (su totale casi ospedalizzati <18 anni)
0-1	267	42	10,9	41,2
2-6	227	20	6,1	19,6
7-17	870	40	3,2	39,2
**<18 anni**	1.364	102	5,2	100,0

Fattori di rischio

- Ad eccezione dei primi tre casi di COVID-19 segnalati, provenienti dalla Cina, nessun'altro caso notificato in Italia ha riportato una storia di viaggio in paesi con trasmissione sostenuta da SARS-CoV-2 durante il periodo di incubazione di 14 gg.

- Sono stati diagnosticati 14.066 casi tra operatori sanitari (età mediana 48 anni, 33% di sesso maschile), circa il 10% dei casi totali segnalati. È evidente l'elevato potenziale di trasmissione in ambito assistenziale di questo patogeno. La tabella 5 riporta la distribuzione dei casi segnalati per classe di età e la letalità osservata in questo gruppo di popolazione.

- I dati indicano che la letalità tra gli operatori sanitari è inferiore rispetto alla letalità totale (vedi tabella 1), verosimilmente dovuto al fatto che gli operatori sanitari asintomatici e pauci-sintomatici, sono stati maggiormente testati rispetto alla popolazione generale. Tuttavia, va anche sottolineato che l'informazione sull'esito della malattia non è nota per un numero elevato di casi.

- La Figura 6 riporta la percentuale di operatori sanitari tra i casi positivi segnalati in totale in Italia, per periodo di diagnosi (ogni 4 giorni). Si osserva che, a 3 giorni

dalla diagnosi dei primi casi di COVID-19 si è verificato un picco nella percentuale di operatori sanitari tra i casi totali diagnosticati nel periodo. Questo verosimilmente riflette l'effettuazione di un numero elevato di test tra gli operatori sanitari in quella fase, che ha fatto emergere le persone positive anche prima che manifestassero la sintomatologia. Il picco è stato seguito da un andamento pressoché costante; solo nell'ultimo periodo di osservazione si è notata una diminuzione della percentuale di casi tra gli operatori sanitari rispetto al totale dei casi. Tuttavia tale informazione potrebbe risentire di un ritardo nell'indagine epidemiologica effettuata da ciascuna regione e quindi richiederà di essere verificata nelle prossime settimane.

TABELLA 5. DISTRIBUZIONE DEI CASI, DECEDUTI E LETALITÀ IN OPERATORI SANITARI

Classe d'età (anni)	Casi [n (%)]	Deceduti [n (%)]	Letalità (%)
**18-29**	1284 (9,2%)	0 (0%)	0,0%
**30-39**	2.346 (16,7%)	2 (4,2%)	0,1%
**40-49**	3.938 (28,1%)	3 (6,3%)	0,1%
**50-59**	4.809 (34,3%)	14 (29,2%)	0,3%
**60-69**	1.568 (11,2%)	20 (41,7%)	1,3%
**70-79**	87 (0,6%)	9 (18,8%)	10,3%
**Totale**	14.032	48	0,3%

NOTA: LA TABELLA NON INCLUDE I CASI CON ETÀ NON NOTA

Per effettuare una valutazione più approfondita delle infezioni da SARS-COV -2 negli operatori sanitari è stata lanciata una breve survey tra le Regioni e P.A. L'obiettivo è quello di valutare retrospettivamente il numero di operatori sanitari che hanno contratto il COVID-19, e descrivere le categorie professionali maggiormente coinvolte, i setting assistenziali, il tipo di servizio e di attività svolta. I risultati di tale indagine, attualmente ancora non completata, saranno presentati in un prossimo bollettino.

Inoltre, sempre relativamente agli operatori sanitari, verrà avviata una nuova indagine epidemiologica retrospettiva in collaborazione tra INAIL, ISS e Regioni/PPAA; inoltre, con una modalità prospettica sarà effettuato uno studio in collaborazione tra le stesse istituzioni per analizzare in dettaglio i principali fattori di rischio per COVID-19 presenti nelle diverse categorie di operatori sanitari. Tali risultati, quando disponibili, saranno riportati in uno specifico documento.

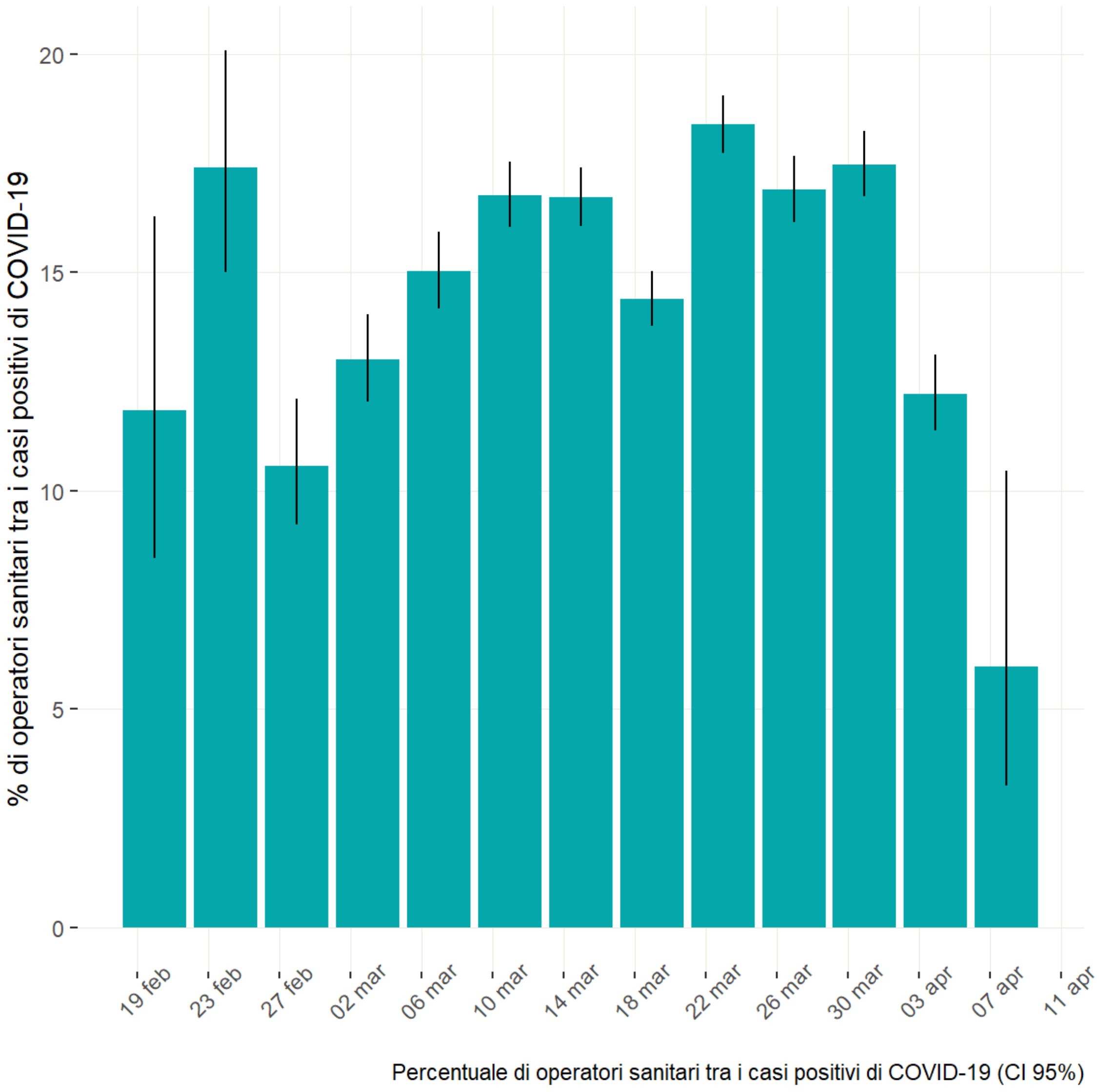

FIGURA 6 – PERCENTUALE DI OPERATORI SANITARI RIPORTATI SUL TOTALE DEI CASI PER PERIODO DI DIAGNOSI

NOTA: OGNI BARRA FA RIFERIMENTO ALL'INTERVALLO DI TEMPO TRA LA DATA INDICATA SOTTO LA BARRA E QUELLA SUCCESSIVA (ESEMPIO: 19 FEB SI RIFERISCE AL PERIODO DAL 19-22 FEB, 23 FEB SI RIFERISCE AL PERIODO DAL 23-26 FEB, ETC.)

Coronavirus Disease 2019 (COVID-19) Daily Situation Report of the Robert Koch Institute

12/04/2020 - UPDATED STATUS FOR GERMANY

Confirmed cases	Deaths	Deaths (%)	Recovered
120.479 (+ 2.821*)	2.673 (+ 129*)	2,2%	ca. 60.300**

– Changes since the last report are marked blue in the text – **Change from previous day; **Estimate*

Summary (as of 12/04/2020, 12:00 AM)

- In total, 120,479 COVID-19 cases and 2,673 deaths due to COVID-19 have been electronically reported to the Robert Koch Institute in Germany.
- All 16 federal states are affected. The incidence (cases per 100,000) of COVID-19 is highest in Bavaria (247), Baden-Wuerttemberg (218), Saarland (208) and Hamburg (203).
- Most cases (69%) are between 15 and 59 years old; men (49%) and women (51%) are almost equally affected.
- 86% of deaths, but only 17% of all cases, occurred in persons aged 70 years or older.
- Reports on COVID-19 related outbreaks in nursing homes and hospitals are increasing. In some of these outbreaks, the number of deaths is relatively high.
- As of 10/04/2020 countries and regions are not longer classified as international risk areas.

Epidemiological Situation in Germany

Geographical distribution of cases

Epidemiological analyses are based on validated cases notified electronically to RKI according to the Protection Against Infection Law (Data closure: 12:00 AM daily).

Since January 2020, a total of 120,479 (+2,821) laboratory-confirmed cases of coronavirus disease 2019 (COVID-19) have been electronically reported to and validated at the RKI, including 2,673 deaths (see Table 1 and Figure 1). Delays in cases reporting and data transmission may occur over the Easter Holiday. In addition, diagnosis is ongoing for suspected cases in several federal states. Information on confirmed cases are also available on the RKI website at https://www.rki.de/DE/Content/InfAZ/N/Neuartiges_Coronavirus/Fallzahlen.html and https://corona.rki.de

Federal State	Total Number of cases	Number of new cases	Cases/100,000 pop.	Number of deaths
**Baden-Wuerttemberg**	24,078	461	218	649
**Bavaria**	32,282	829	247	786
**Berlin**	4,567	109	122	50
**Brandenburg**	1,857	93	74	38
**Bremen**	480	11	70	15
**Hamburg**	3,742	79	203	56
**Hesse**	5,859	51	94	123
**Mecklenburg-Western Pomerania**	605	4	38	11
**Lower Saxony**	7,602	259	95	189
**North Rhine-Westphalia**	24,267	623	135	495
**Rhineland-Palatinate**	4,734	112	116	64
**Saarland**	2,058	42	208	43
**Saxony**	3,600	35	88	67
**Saxony-Anhalt**	1,166	31	53	21
**Schleswig-Holstein**	2,118	53	73	41
**Thuringia**	1,464	29	68	25
**Total**	**120,479**	**2,821**	**145**	**2,673**

Table 1: Number and cumulative incidence (per 100,000 population) of notified laboratory-confirmed COVID-19 cases and deaths per federal state, Germany (12/04/2020, 12:00 AM).

Note: The report is a snapshot and is continuously updated.

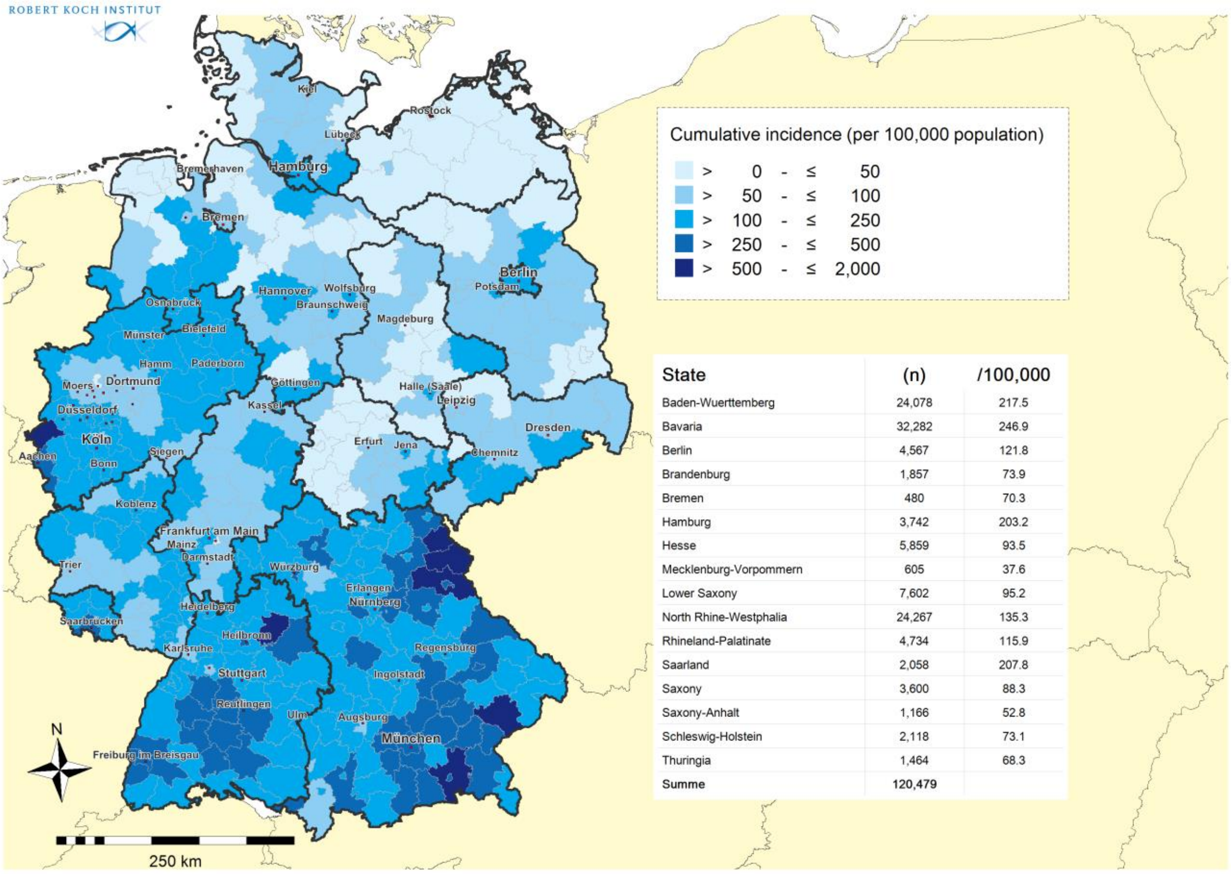

State	(n)	/100,000
Baden-Wuerttemberg	24,078	217.5
Bavaria	32,282	246.9
Berlin	4,567	121.8
Brandenburg	1,857	73.9
Bremen	480	70.3
Hamburg	3,742	203.2
Hesse	5,859	93.5
Mecklenburg-Vorpommern	605	37.6
Lower Saxony	7,602	95.2
North Rhine-Westphalia	24,267	135.3
Rhineland-Palatinate	4,734	115.9
Saarland	2,058	207.8
Saxony	3,600	88.3
Saxony-Anhalt	1,166	52.8
Schleswig-Holstein	2,118	73.1
Thuringia	1,464	68.3
Summe	120,479	

Figure 1: Number and cumulative incidence (per 100,000 population) of the 120,479 electronically reported COVID-19 cases in Germany by county and federal state (12/04/2020, 12:00 AM).

Please see the COVID-19 dashboard (https://corona.rki.de/) for information on number of COVID-19 cases by county (local health authority).

Distribution of cases over time

COVID-19 cases were first notified in Germany in January 2020. In 45,288 cases, onset of symptoms is unknown and therefore date of reporting is shown (see Figure 2).

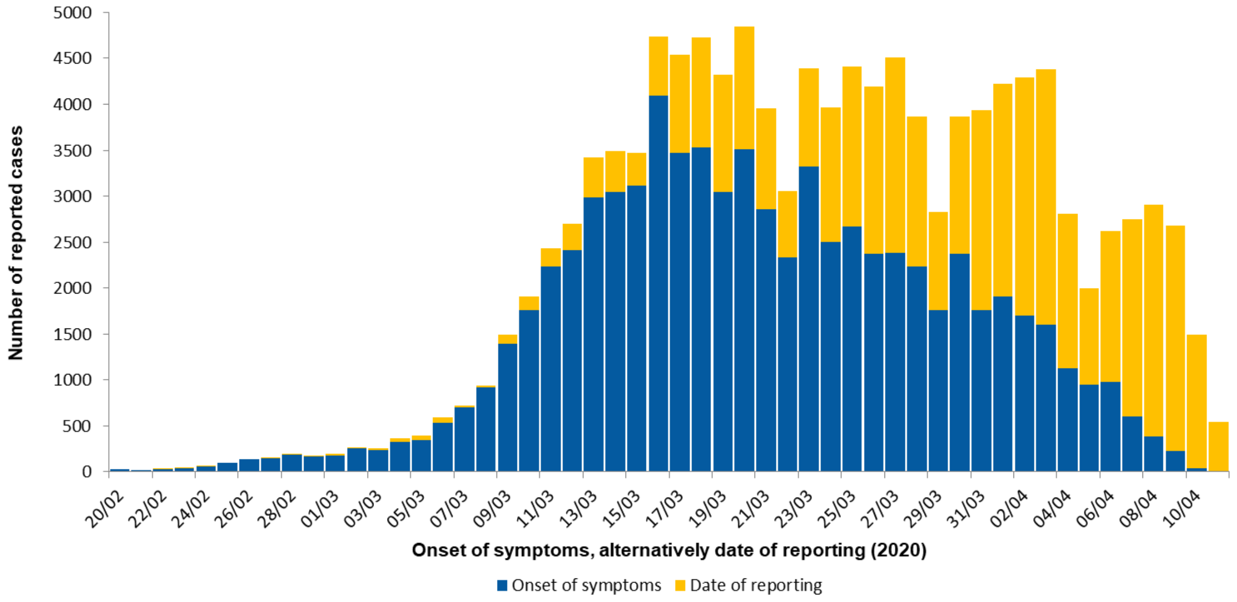

Figure 2: Number of electronically reported COVID-19 cases in Germany by date of symptom onset and alternatively by date of reporting from 20/02/2020 (12/04/2020, 12:00 AM)

Note: The report is a snapshot and is continuously updated.

Demographic distribution of cases

Of reported cases, 49% are male and 51% are female. Among notified cases, 947 were children under 5 years of age, 2,362 children aged 5 to 14 years, 82,461 persons aged 15 to 59 years, 23,340 persons aged 60 to 79 years and 11,165 persons aged 80 years and older (see Figure 3). The age of 204 notified cases is unknown. The median age of cases is 50 years.

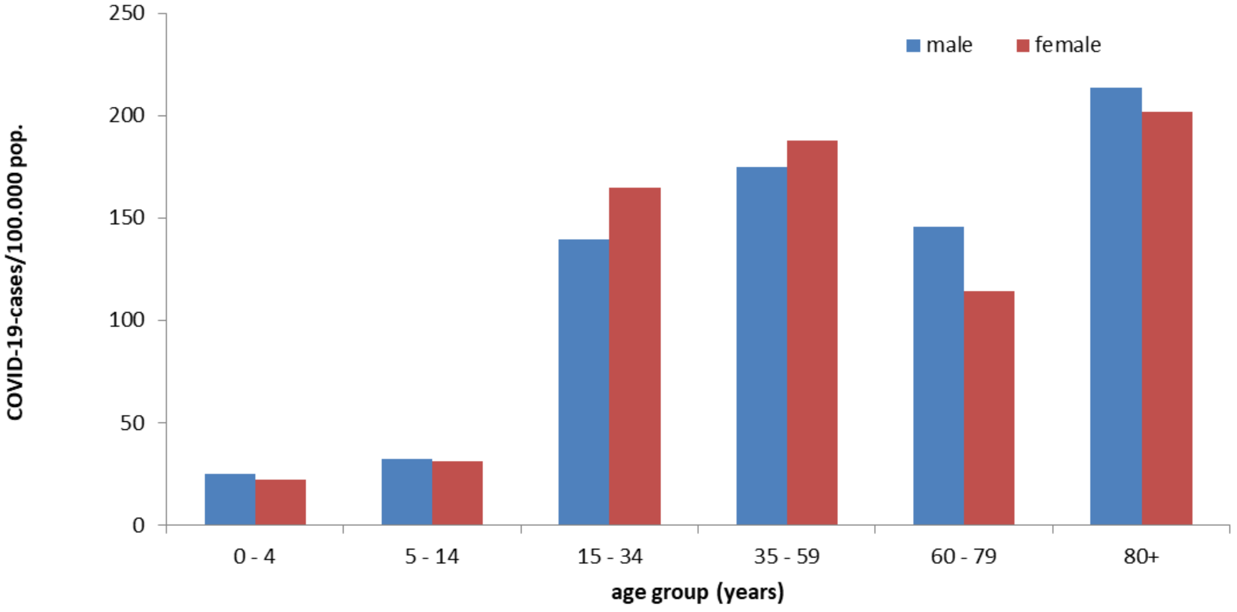

Figure 3: Electronically reported COVID-19 cases/100,000 population in Germany by age group and sex (n=119,889) for cases with information available (12/04/2020, 12:00 AM)

Clinical aspects

Information on symptoms is available for 92,361 of the notified cases. The most common symptoms are cough (51%), fever (42%) and rhinorrhoea (22%). Pneumonia was reported in 2,180 cases (2%). Hospitalisation was reported for 14,129 (15%) of 92,098 COVID-19 cases with information on hospitalisation available.

Approximately 60,300 persons are estimated to have recovered from their COVID-19 infection. As the exact date of recovery is unknown in most cases, an algorithm was developed to estimate the number of recovered cases.

The 2,673 COVID-19 related deaths reported in Germany concerned 1,592 (60%) men and 1,076 (40%) women (sex was unknown in 5 cases, age was unknown in 2 cases). The median age was 82 years. Of all deaths, 2,304 (86%) were in persons 70 years or older, but only 17% of all cases were in this age group. Reports on COVID-19 related outbreaks in nursing homes are increasing. In some of these outbreaks, the number of deaths is relatively high.

	Age groups (years)				
**Sex**	**<60**	**60 - 69**	**70 - 79**	**80 - 89**	**≥90**
**male**	97	176	445	689	185
**female**	31	62	196	530	257

Table 2: Number of COVID-19 cases notified as having died by age group and sex (available for 2,668 of the 2,673 deaths; 12/04/2020, 12:00 AM)

Note: The report is a snapshot and is continuously updated.

SARS-CoV-2 infections among staff working in medical facilities

Of notified cases with a SARS-CoV-2 infection, at least 5,500 were reported among staff working in medical facilities as defined by §23 of the German Protection Against Infection law (IfSG), such as hospitals, outpatient clinics and practices, dialysis clinics or outpatient nursing services. Among these 5,500 persons 72% were female and 28% male. The median age was 42 years.

Estimation of the reproduction number (R)

The reproduction number, R_0, is the mean number of persons infected by a case. R_0 can only be estimated and not directly extracted from the notification system. The current estimate is R= 1.3 (95% confidence interval: 1.0-1.6) and is based on electronically notified cases (10/04/2020 12:00 AM) and an assumed mean generation time of 4 days. Cases with disease onset on the preceding 3 days were excluded from the estimation as their low number due to incomplete reporting would lead to an unstable estimate. For more details on the methodology see Epid. Bull. 17 | 2020 Online vorab: 9. April 2020 https://www.rki.de/DE/Content/Infekt/EpidBull/Archiv/2020/Ausgaben/17_20_SARS-CoV2_vorab.pdf?__blob=publicationFile

DIVI intensive care register[1]

A registry of the German Interdisciplinary Association for Intensive and Emergency Medicine (DIVI), the RKI and the German Hospital Federation (DKG) was set up to document the capacities for intensive care as well as the number of COVID-19 cases treated in participating hospitals: https://www.intensivregister.de/#/intensivregister .
As of 12/04/2020, 752 hospitals or departments reported to DIV I (-2 since 10/04/2020) to the registry. Migration of the registry to a new server has led to reporting delays, thus comparisons with data prior to 03/04/2020 are not meaningful. Currently, likely fewer than half of all ICU beds in Germany are captured by the registry. A total of 19,701 intensive care beds were registered, of which 11,376 (58%) are occupied and 8,325 beds are currently available (Table 3).

	Number of patients	Percentage	Changes to previous day*
Currently in ICU	2,405		
- of these: mechanically ventilated	1,859	77%	
Discharged from ICU	2,978		
- of these: deaths	864	29%	

Table 3: COVID-19 patients requiring intensive care (ICU) recorded in the DIVI register (12/04/2020, 8:15 am)
*Due to the changing number of reporting hospitals from day to day this comparison is currently not meaningful

[1] The situation report as of 11/04/2020 reported on data with state of date 10/04/2020, as there were no new data at 11/04/2020 available from the DIVI intensive care register.

Assessment by the RKI

At the global and the national level, the situation is very dynamic and must be taken seriously. Severe and fatal courses occur in some cases. The number of cases, hospitalisations and fatalities in Germany continues to increase. The RKI currently assesses the risk to the health of the German population overall as **high** and as **very high** for risk groups. The probability of serious disease progression increases with increasing age and underlying illnesses. The risk of disease varies from region to region. The burden on the health care system depends on the geographical and age distribution of cases, health care capacity and initiation of containment measures (isolation, quarantine, social distancing etc.), and may be very high in some geographical regions. This assessment may change on short notice as a result of new findings.

Measures taken by Germany

International risk areas

As of 10/04/2020 countries and regions are not longer classified as international risk areas. Due to pandemic spread, there is a global risk of acquiring COVID-19. In a considerable number of countries there are outbreaks with sometimes large numbers of cases; the exact numbers of cases in other countries are not known. Therefore there is a risk of transmission in Germany as well as in many other, not always well-defined regions worldwide.

All arriving travellers in Germany are to remain in quarantine for 14 days: https://www.bundesregierung.de/breg-de/themen/coronavirus/neue-einreiseregeln-1739634 *(in German)*

Other measures

- Outbreaks are ongoing in many districts, some with high case numbers. Data on current disease activity can be found on the dashboard https://corona.rki.de/ and in the daily situation reports.
- RKI teams are currently supporting outbreak containment measures with a focus on outbreaks in retirement and health care homes as well as hospitals in several federal states.
- A new federal law was implemented on 28/03/2020 for the protection of the public in the event of epidemic situations, granting the federal government additional competencies for the control of epidemics. https://www.bundesgesundheitsministerium.de/presse/pressemitteilungen/2020/1-quartal/corona-gesetzespaket-im-bundesrat.html *(in German)*
- As of 23/03/2020, gatherings of more than 2 persons (with the exception of families and household members) are banned in all federal states. Restaurants and businesses concerned with body care were closed. In public spaces, all persons must maintain a distance of 1.5 metres to other indivduals https://www.bundesregierung.de/breg-de/themen/coronavirus/besprechung-der-bundeskanzlerin-mit-den-regierungschefinnen-und-regierungschefs-der-laender-1733248 *(in German)*

GOBIERNO DE ESPAÑA
MINISTERIO DE SANIDAD
SECRETARIA GENERAL DE SANIDAD
DIRECCIÓN GENERAL DE SALUD PÚBLICA, CALIDAD E INNOVACIÓN
Centro de Coordinación de Alertas y Emergencias Sanitarias

Actualización nº 74. Enfermedad por el coronavirus (COVID-19). 13.04.2020 *(datos consolidados a las 21:00 horas del 12.04.2020)*

1. SITUACIÓN ACTUAL

Situación en España:

En España, hasta el momento se han registrado 169.496 casos, 17.489 fallecidos y 64.727 curados (Tabla 1 y Figura 1). Las Comunidades Autónomas con una mayor incidencia acumulada en los últimos 14 días son La Rioja, Castilla La Mancha, Madrid, y Navarra (Figura 2 y Figura 3). La distribución por grupos de edad de casos hospitalizados, ingresados en UCI y fallecidos se recoge en la Tabla 2.

Tabla 1. Casos COVID-19, incidencia acumulada (IA) en los últimos 14 días, ingreso en UCI y fallecidos por Comunidades Autónomas en España, 13.04.2020 (datos consolidados a las 21:00 horas del 12.04.2020).

CCAA	TOTAL confirmados*	IA (14 d.)	Casos que han precisado hospitalización¥	Casos que han ingresado en UCI	Fallecidos	Curados	*Nuevos*
**Andalucía**	**10.187**	56,83	4.993	621	815	1.967	*181*
**Aragón**	**4.187**	159,86	1.938	274	464	946	*117*
**Asturias**	**1.958**	78,22	1.265	110	149	434	*66*
**Baleares**	**1.550**	47,85	811	143	117	862	*16*
**Canarias**	**1.944**	34,36	763	145	96	458	*26*
**Cantabria**	**1.777**	116,51	827	75	117	317	*25*
**Castilla La Mancha**	**14.054**	403,18	7.782	475	1.626	2.532	*356*
**Castilla y León**	**12.628**	284,51	6.077	312¥	1.263	4.154	*510*
**Cataluña**	**34.726**	241,93	22.780	2.713	3.538	15.602	*699*
**Ceuta**	**95**	82,57	9	4	4	20	*2*
**C. Valenciana**	**9.060**	78,94	4.269	576	876	2.803	*219*
**Extremadura**	**2.658**	102,84	1.096	108	321	511	*78*
**Galicia**	**7.494**	139,69	2.344	136¥	284	1.186	*158*
**Madrid**	**47.146**	346,01	11.233¥	1.327¥	6.423	25.385	*559*
**Melilla**	**101**	57,81	43	3	2	18	*3*
**Murcia**	**1.463**	35,08	543	94	101	353	*14*
**Navarra**	**4.092**	297,46	1.693	124	239	695	*123*
**País Vasco**	**11.018**	224,71	5.622	464	831	5.026	*246*
**La Rioja**	**3.358**	512,95	1.141	73	223	1.458	*79*
**ESPAÑA**	**169.496**	179,26			**17.489**	**64.727**	*3.477*

IA (14 d.): Incidencia acumulada (casos acumulados por 100.000 habitantes) en los últimos 14 días.
Los datos de las CCAA están en continua revisión y ciertas oscilaciones diarias pueden deberse a procesos de depuración de datos y no a variaciones reales acontecidas de un día a otro.
** Los casos confirmados no provienen de la suma de pacientes hospitalizados, curados y fallecidos, ya que no son excluyentes. Pacientes fallecidos y curados pueden haber precisado hospitalización y por tanto computar en ambos grupos. Los pacientes que han precisado UCI también computan en los pacientes que han requerido hospitalización.*
¥ Los datos de estas comunidades son datos de prevalencia (personas ingresadas a fecha de hoy). No reflejan el total de personas que han sido hospitalizadas o ingresadas en UCI a lo largo del periodo de notificación por lo que no se puede realizar el sumatorio de todas las personas que han requerido hospitalización o ingreso en UCI en España.
Fuente elaboración propia

Tabla 2. Distribución de casos hospitalizados, ingresados en UCI y fallecidos por grupos de edad con datos notificados incluyendo edad y sexo*. (datos consolidados a las 21:00 horas del 12.04.2020).

Grupo de edad (años)	Total							
	Confirmados	Hospitalizados totales		UCI		Fallecidos		
	n	n	%	n	%	n	%	Letalidad(%)
0-9	396	154	0,3	20	0,4	1	0,0	0,3
10-19	647	129	0,2	5	0,1	1	0,0	0,2
20-29	6.137	914	1,6	53	1,1	17	0,2	0,3
30-39	11.484	2.409	4,2	170	3,5	35	0,4	0,3
40-49	17.962	5.500	9,7	424	8,7	109	1,1	0,6
50-59	22.336	8.933	15,7	938	19,3	281	3,0	1,3
60-69	19.747	11.469	20,1	1.563	32,1	873	9,2	4,4
70-79	18.716	13.726	24,1	1.519	31,2	2.607	27,5	13,9
80-89	16.468	10.762	18,9	160	3,3	3.969	41,9	24,1
90 y +	6.101	2.976	5,2	16	0,3	1.589	16,8	26,0
Total	119.994	56.972	100%	4.868	100%	9.482	100%	

Grupo de edad (años)	Mujeres							
	Confirmados	Hospitalizados totales		UCI		Fallecidos		
	n	n	%	n	%	n	%	Letalidad(%)
0-9	184	64	0,3	6	0,4	1	0,0	0,5
10-19	341	71	0,3	2	0,1	1	0,0	0,3
20-29	4.000	462	1,9	22	1,5	5	0,1	0,1
30-39	6.948	1.132	4,7	61	4,3	15	0,4	0,2
40-49	9.989	2.197	9,1	118	8,3	39	1,1	0,4
50-59	12.095	3.549	14,8	260	18,3	89	2,4	0,7
60-69	9.134	4.511	18,8	432	30,4	259	7,0	2,8
70-79	7.884	5.410	22,5	440	31,0	770	20,9	9,8
80-89	8.537	4.952	20,6	73	5,1	1.624	44,1	19,0
90 y +	3.989	1.696	7,1	7	0,5	880	23,9	22,1
Total	63.101	24.044	100%	1.421	100%	3.683	100%	

Grupo de edad (años)	Hombres							
	Confirmados	Hospitalizados totales		UCI		Fallecidos		
	n	n	%	n	%	n	%	Letalidad(%)
0-9	212	90	0,3	14	0,4	0	0,0	0,0
10-19	306	58	0,2	3	0,1	0	0,0	0,0
20-29	2.131	447	1,4	31	0,9	12	0,2	0,6
30-39	4.535	1.276	3,9	109	3,2	20	0,3	0,4
40-49	7.971	3.303	10,0	306	8,9	70	1,2	0,9
50-59	10.237	5.382	16,4	677	19,7	192	3,3	1,9
60-69	10.610	6.956	21,1	1.131	32,8	614	10,6	5,8
70-79	10.829	8.313	25,3	1.077	31,3	1.837	31,7	17,0
80-89	7.930	5.809	17,6	87	2,5	2.345	40,4	29,6
90 y +	2.112	1.280	3,9	9	0,3	709	12,2	33,6
Total	56.873	32.914	100%	3.444	100%	5.799	100%	

**Datos obtenidos del análisis sobre casos con edad y sexo 119.974 casos notificados con información de edad y sexo. Estos datos se actualizan permanentemente, pudiendo existir una disminución del nº de casos en algún grupo de edad y sexo.*

Fuente elaboración propia

Figura 1. Casos diarios confirmados de COVID-19 por fecha de notificación, diagnóstico e inicio de síntomas.

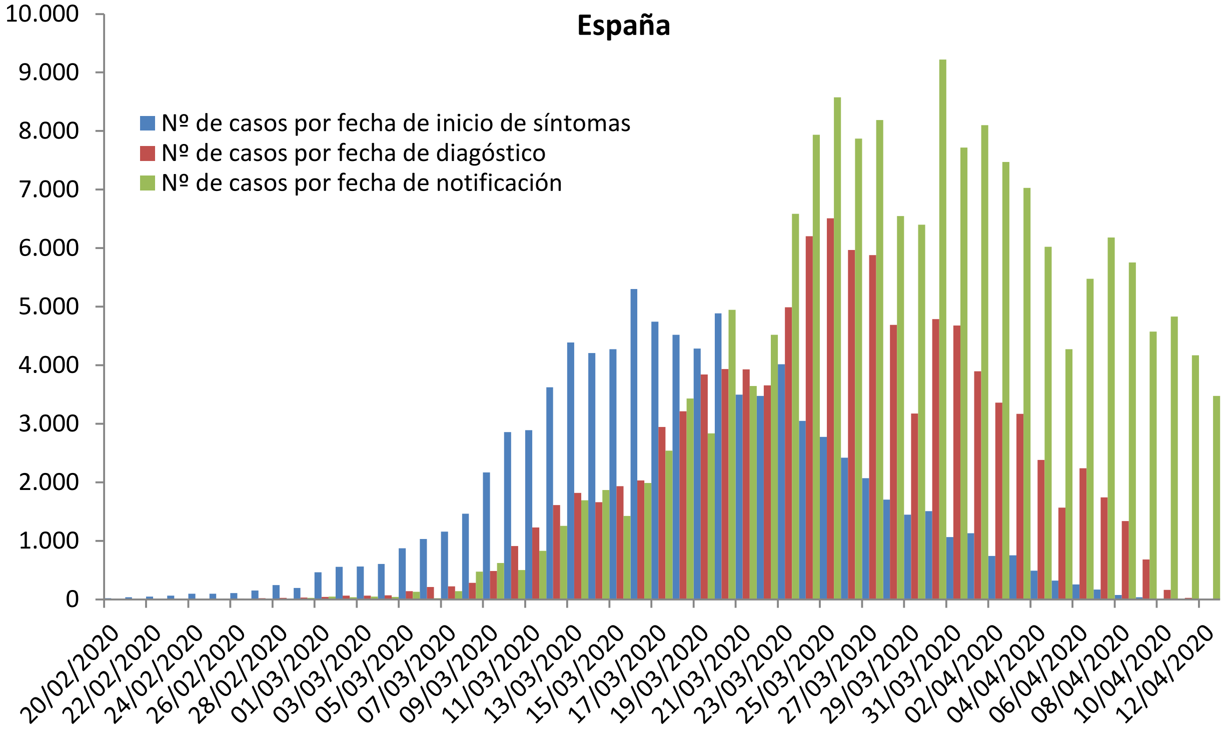

*Se dispone de fecha de inicio de síntomas en en 87.109 casos y de diagnóstico en 101.815 casos

Figura 2. Casos diarios confirmados de COVID-19 por fecha de notificación en las Comunidades Autónomas con mayor incidencia en los últimos 14 días, 13.04.2020 (datos consolidados a las 21:00 horas del 12.04.2020).

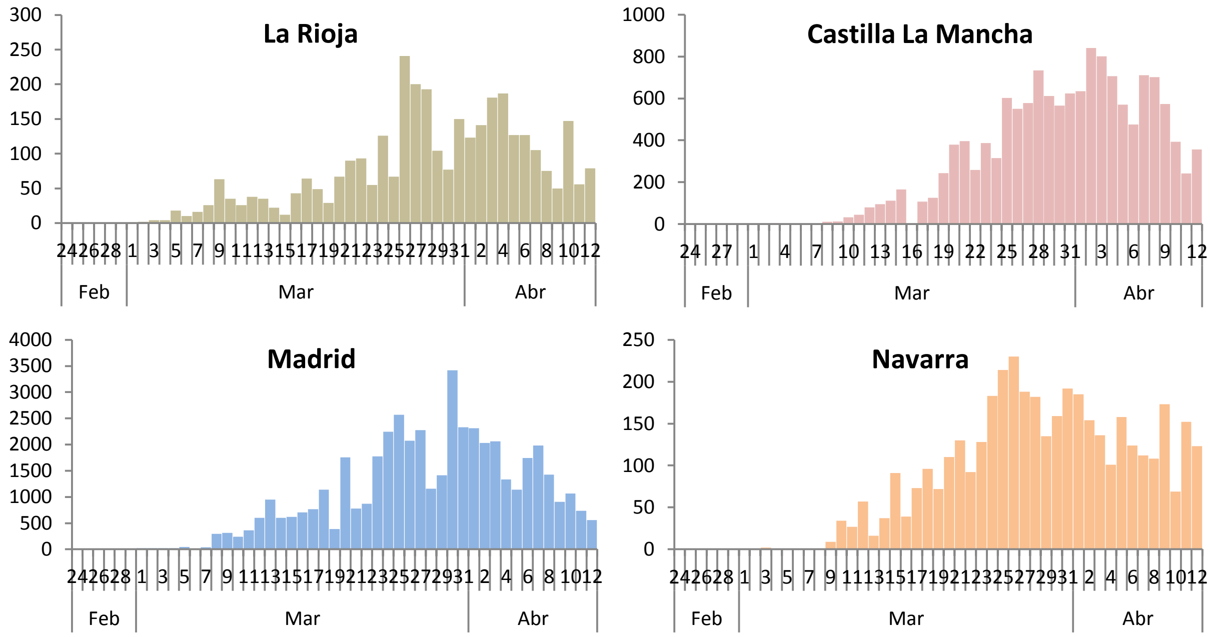

Figura 3. Casos diarios confirmados de COVID-19 por fecha de notificación por Comunidades Autónomas, 13.04.2020 (datos consolidados a las 21:00 horas del 12.04.2020).

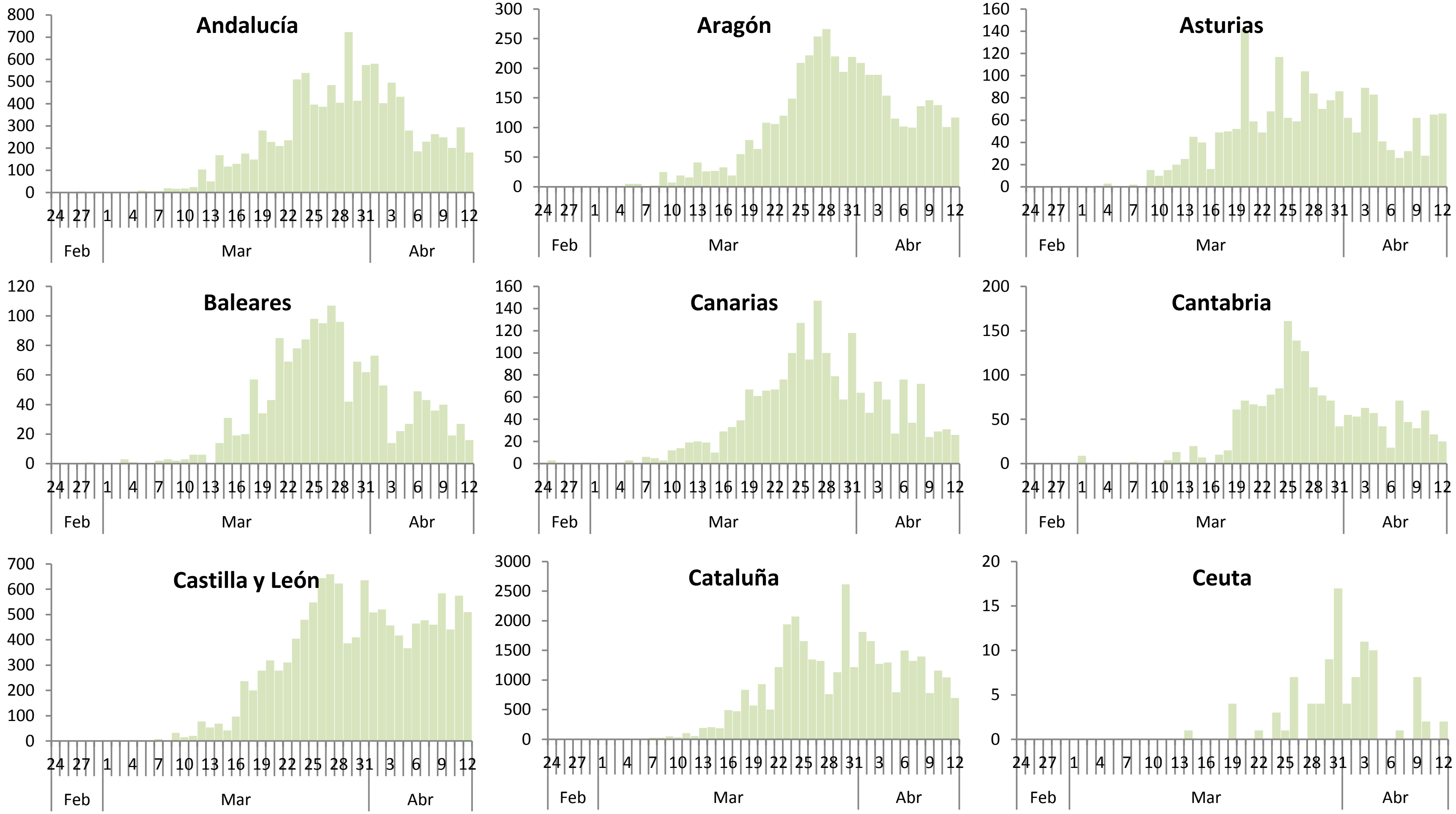

SECRETARIA GENERAL DE SANIDAD

DIRECCIÓN GENERAL DE SALUD PÚBLICA, CALIDAD E INNOVACIÓN

Centro de Coordinación de Alertas y Emergencias Sanitarias

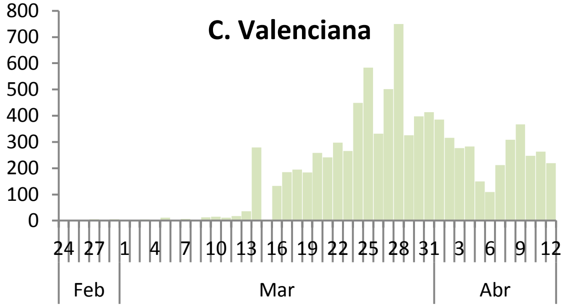

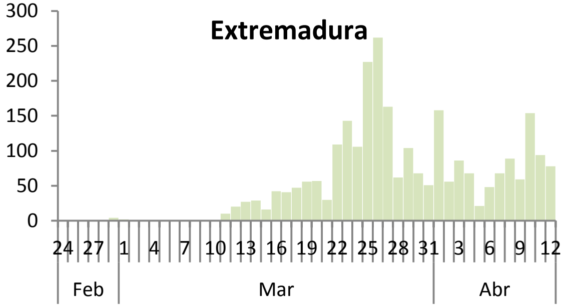

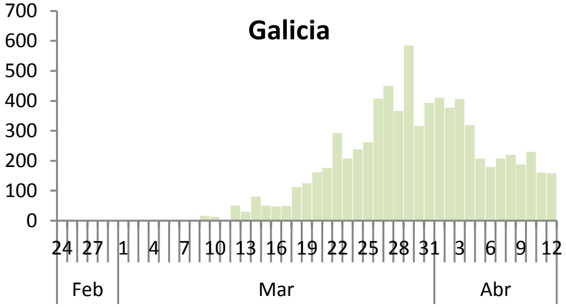

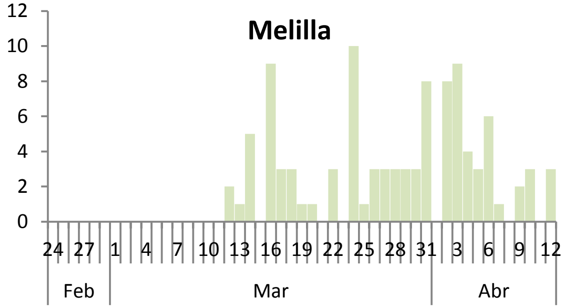

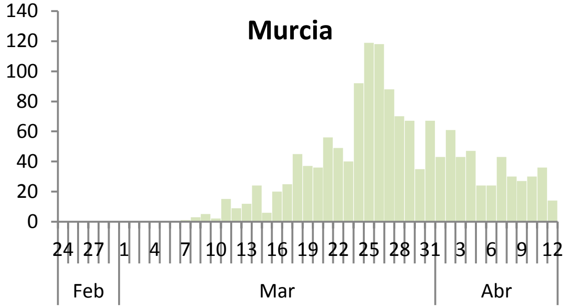

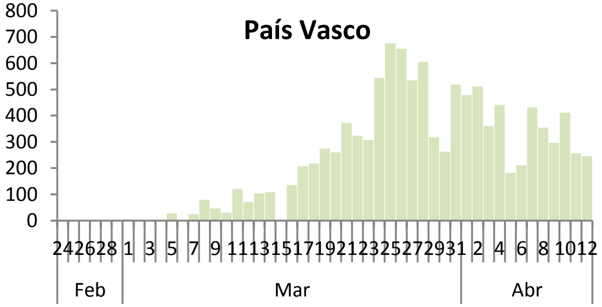

SITUACIÓN INTERNACIONAL (*datos consultados a las 7:00 horas del 13.04.2020)*

Situación en Europa:

En Europa, hasta el día de hoy se han notificado al menos 904.518 casos confirmados. (Tabla 3) Los países con más casos notificados son España (169.496), Italia (156.363), Alemania (120.479), Francia (95.403) y Reino Unido (84.279). El país con mayor número de fallecidos es Italia (19.899) seguido de España (17.489), Francia (14.393) y Reino Unido (10.612) (Tabla 4 y Figura 4). En Italia, las regiones más afectadas son Lombardía (59.052), seguida de Emilia Romagna (20.098), Piemonte (16.660) y Veneto (14.077). En Alemania, las regiones más afectadas son Bayern (32.282), Nordrhein-Westfalen (24.267) y Baden-Württemberg (24.078). En Francia, las regiones más afectadas son Île-de-France y Gran Este. Inglaterra (66.330) y Escocia (5.912) son las partes más afectadas de Reino Unido.

Tabla 3. Casos confirmados de COVID-19 en Europa. (Datos consultados a las 7:00 horas del 13.04.2020[1])

	Casos		Casos		Casos		Casos
España	169.496	Suecia	10.151	Islandia	1.689	Andorra	622
Italia	156.363	Irlanda	8.928	República de Moldavia	1.560	Chipre	616
Alemania	120.479	Polonia	6.356	Croacia	1.534	Albania	446
Francia	95.403	Noruega	6.320	Hungría	1.410	Malta	370
Reino Unido	84.279	Dinamarca	5.996	Estonia	1.304	San Marino	356
Turquía	56.956	Rumanía	5.990	Eslovenia	1.188	Montenegro	262
Bélgica	29.647	Chequia	5.902	Lituania	1.053	Georgia	252
Países Bajos	25.587	Serbia	3.380	Armenia	1.013	Islas Faroe	184
Suiza	25.300	Luxemburgo	3.270	Bosnia y Herzegovina	948	Gibraltar	113
Portugal	16.585	Finlandia	2.905	Macedonia del Norte	828	Liechtenstein	80
Rusia	15.770	Ucrania	2.777	Eslovaquia	728	Mónaco	54
Austria	13.945	Bielorrusia	2.226	Bulgaria	661		
Israel	10.525	Grecia	2.081	Letonia	630		
	**Total**						**904.518**

[1] *Datos consultados en las páginas web de las autoridades sanitarias de los propios países (los doce países más afectados), en la página web de la Oficina Regional para Europa de la Organización Mundial de la Salud (OMS) y del Centro Europeo de Prevención y Control de Enfermedades (ECDC).*

Tabla 4. Casos confirmados, IA de los últimos 14 días, nº de fallecidos y letalidad en los doce países más afectados de Europa (Datos consultados a las 7:00 horas del 13.04.2020[2])

	Casos confirmados	Casos nuevos desde ayer	IA últimos 14 días	Fallecidos totales	Fallecidos nuevos desde ayer	Letalidad
**España**	169.496	*+3.477*	179,26	17.489	*+517*	10,32
**Italia**	156.363	*+4.092*	97,01	19.899	*+431*	12,73
**Alemania**	120.479	*+2.821*	82,05	2.673	*+129*	2,22
**Francia**	95.403	*+1.613*	82,44	14.393	*+561*	15,09
**Reino Unido**	84.279	*+5.288*	93,52	10.612	*+737*	12,59
**Turquía**	56.956	*+4.789*	56,24	1.198	*+97*	2,10
**Bélgica**	29.647	*+1.629*	170,30	3.600	*+254*	12,14
**Países bajos**	25.587	*+1.174*	86,45	2.737	*+94*	10,70
**Suiza**	25.300	*+400*	136,10	858	*+27*	3,39
**Portugal**	16.585	*+598*	105,90	504	*+34*	3,04
**Rusia**	15.770	*+2.186*	9,89	130	*+24*	0,82
**Austria**	13.945	*+384*	57,93	350	*+13*	2,51

IA: Incidencia acumulada (casos acumulados por 100.000 habitantes)

[2] *Datos consultados: Italia (Ministerio de Sanidad), Alemania (Robert Koch Institut), Francia (Santé Publique France), Reino Unido (Public Health England), Suiza (Office Fédéral de la Santé Publique), Turquía (Ministerio de Sanidad), Bélgica (Service Public Fédéral Sante Publique,Securite de la Chaine Alimentaire et Environnement), Países Bajos (National Institute for Public Health and the Environment),Portugal (Dirección General de Sanidad), Rusia (Ministerio de Sanidad), Austria (Ministerio de sanidad).*

Figura 4. Casos nuevos confirmados de COVID-19 por fecha de notificación en Italia, España, Alemania, Francia y Reino Unido. (Datos consultados a las 7:00 horas del 13.04.2020)

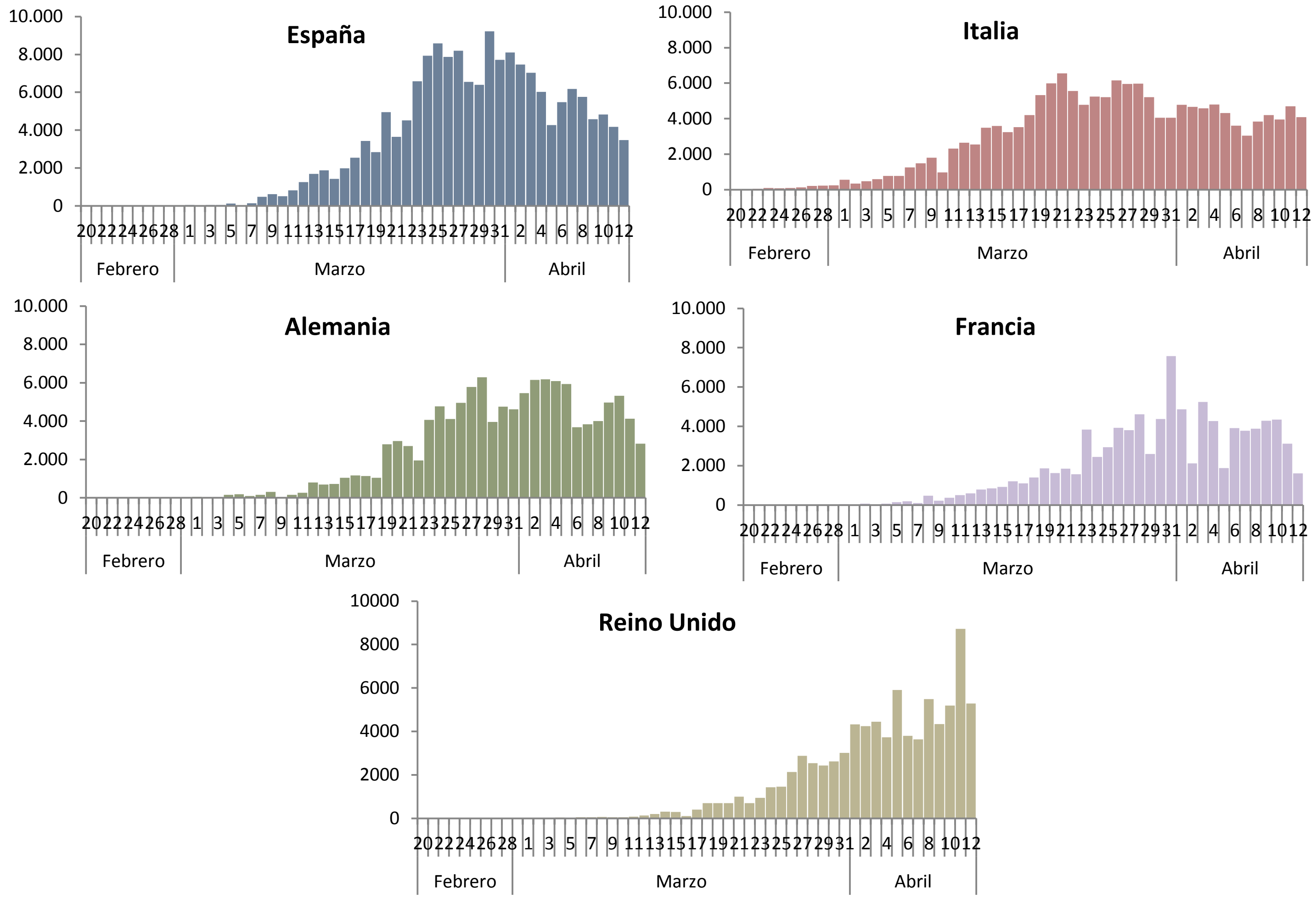

Situación a nivel global y otros países fuera de Europa:

A nivel global hasta el día de hoy se han notificado al menos 1.699.595 casos y 106.138 fallecidos (Tabla 6). Los países de fuera de Europa que han registrado más casos son Estados Unidos (525.704), China (83.587), Irán (71.686), Canadá (23.702), Brasil (22.169) y Corea del Sur (10.537) (Tabla 5, Tabla 6 y Figura 5). En Estados Unidos, los Estados que más casos han notificado[3] son Nueva York (157.073), Nueva Jersey (51.027), Michigan (21.504) y Massachusetts (18.941)

Tabla 5. Casos confirmados de COVID-19 fuera de Europa. Se incluyen los 10 países más afectados en cada continente. (Datos consultados a las 7:00 horas del 13.04.2020[4])

América	Casos	África	Casos	Asia-Oceanía	Casos
Estados Unidos	525.704	Sudáfrica	2.028	China	83.597
Canadá	23.702	Egipto	1.939	Irán	71.686
Brasil	22.169	Argelia	1.825	Corea del Sur	10.537
Ecuador	7.257	Marruecos	1.545	India	8.356
Chile	6.927	Camerún	820	Japón	7.444
Perú	6.848	Túnez	685	Australia	6.289
México	4.219	Costa de Marfil	533	Pakistán	5.038
Panamá	3.234	Níger	491	Malasia	4.530
República Dominicana	2.759	Burkina Faso	484	Filipinas	4.428
Colombia	2.709	Ghana	408	Arabia Saudí	4.033

3 CDC actualiza estos datos se actualizan de lunes a viernes
[4] *Datos consultados en las páginas web de las autoridades sanitarias de los propios países (los seis países más afectados), en la página web de la Oficina Regional para Europa de la Organización Mundial de la Salud (OMS) y del Centro Europeo de Prevención y Control de Enfermedades (ECDC).*

Tabla 6. Casos confirmados, IA de los últimos 14 días, nº de fallecidos y letalidad en los seis países más afectados fuera de Europa y a nivel global. (Datos consultados a las 7:00 horas del 13.04.2020[5])

	Casos confirmados	Casos nuevos desde ayer	IA últimos 14 días	Fallecidos totales	Fallecidos nuevos desde ayer	Letalidad
**Estados Unidos**	525.704	*+33.288*	122,81	20.486	*+1.927*	3,90
**China**	83.597	*+115*	0,08	3.351	*+2*	*4,01*
**Irán**	71.686	*+1.657*	41,12	4.474	*+117*	*6,24*
**Canadá**	23.702	*+1.158*	46,91	674	*+74*	2,84
**Brasil**	22.169	*+1.442*	8,41	1.223	*+99*	5,52
**Corea del Sur**	10.537	*+25*	1,70	217	*+3*	*2,06*
**GLOBAL**	Más de 1.699.595			Más de 106.138		

IA: Incidencia acumulada (casos acumulados por 100.000 habitantes)

[5] *Datos consultados: Estados Unidos (Centers for Disease Control and Prevention,CDC)), China (Chinese Center for Disease Control and Prevention), Irán (OMS), Canadá (Gobierno de Canadá), Brasil (Ministerio de Sanidad), Corea del Sur (Ministerio de Sanidad).*

Figura 5. Casos nuevos confirmados de COVID-19 por fecha de notificación en Estados Unidos, Irán y Corea del Sur. (Datos consultados a las 7:00 horas del 13.04.2020)

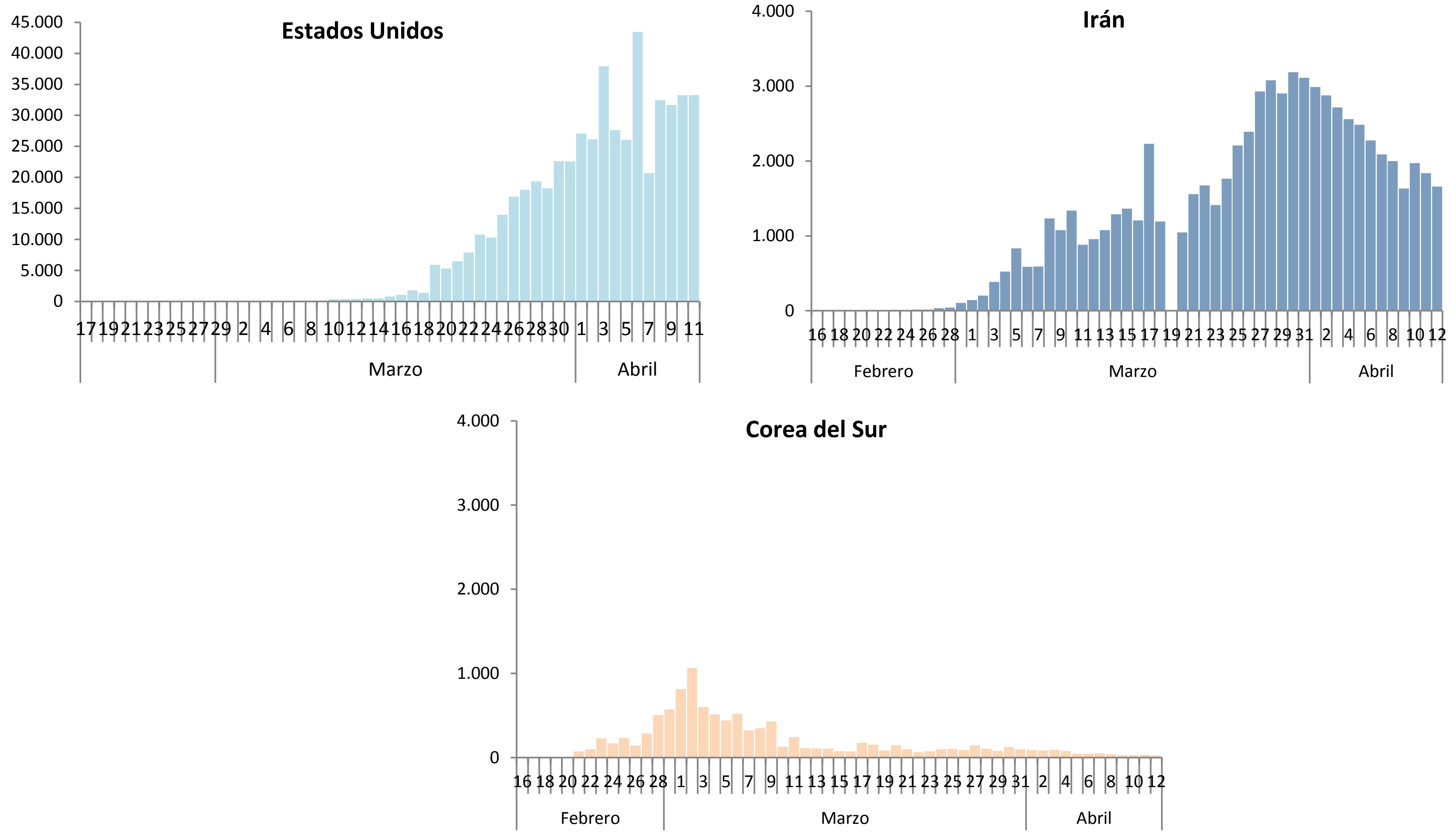

GOBIERNO DE ESPAÑA MINISTERIO DE SANIDAD

SECRETARIA GENERAL DE SANIDAD

DIRECCIÓN GENERAL DE SALUD PÚBLICA, CALIDAD E INNOVACIÓN

Centro de Coordinación de Alertas y Emergencias Sanitarias

Actualización nº 120. Enfermedad por el coronavirus (COVID-19). 29.05.2020 *(datos consolidados a las 12:00 horas del 29.05.2020)*

SITUACIÓN EN ESPAÑA

El presente informe se ha realizado, hasta el 10 de mayo de 2020, con los datos notificados diariamente de forma agregada por las comunidades autónomas. El pasado 11 de mayo de 2020 entró en vigor la nueva estrategia de diagnóstico, vigilancia y control en la fase de transición de la pandemia de COVID-19, por la que las comunidades autónomas deben notificar los casos confirmados de forma individualizada y diariamente al nivel estatal. Por lo tanto, a partir del 11 de mayo de 2020 se utiliza dicha información para la elaboración de este informe diario. Una vez combinados los datos de ambos métodos de vigilancia, en España hasta el momento se han notificado un total de 238.564 casos confirmados de COVID-19 y 27.121 fallecidos (Tabla 1, Tabla 2, Figura 1 y Figura 2). Las discrepancias que puedan aparecer respecto a los datos de casos totales notificados previamente son resultado de la validación de los mismos por las comunidades autónomas y a la transición a la nueva estrategia de vigilancia. Esta discrepancia podría persistir aún varios días.

Tabla 1. Casos de COVID-19 confirmados totales, diagnosticados el día previo y diagnosticados o con fecha de inicio de síntomas en los últimos 14 y 7 días. 28.05.2020. #

CCAA	Casos Totales*	Casos diagnosticados el día previo	Casos diagnosticados en los últimos 14 días		Casos diagnosticados en los últimos 7 días		Casos diagnosticados con fecha de inicio de síntomas en los últimos 14d.		Casos diagnosticados con fecha de inicio de síntomas en los últimos 7d.	
			Nº	IA**	Nº	IA**	Nº	IA**	Nº	IA**
**Andalucía**	12.655	7	144	1,71	47	0,56	19	0,23	3	0,04
**Aragón**	5.671	14	244	18,49	103	7,81	46	3,49	14	1,06
**Asturias**	2.401	1	17	1,66	6	0,59	7	0,68	1	0,10
**Baleares**	2.129	1	76	6,61	24	2,09	21	1,83	3	0,26
**Canarias**	2.333	5	57	2,65	22	1,02	10	0,46	1	0,05
**Cantabria**	2.298	1	39	6,71	21	3,61	11	1,89	4	0,69
**Castilla La Mancha**	17.122	3	400	19,68	166	8,17	76	3,74	20	0,98
**Castilla y León**	18.747	7	569	23,71	266	11,09	70	2,92	16	0,67
**Cataluña**	58.699	47	3.041	39,62	1.384	18,03	385	5,02	84	1,09
**Ceuta**	145	6	28	33,03	20	23,59	13	15,33	8	9,44
**C. Valenciana**	11.191	11	280	5,60	114	2,28	35	0,70	11	0,22
**Extremadura**	2.963	2	28	2,62	15	1,40	10	0,94	6	0,56
**Galicia**	9.117	1	84	3,11	28	1,04	15	0,56	3	0,11
**Madrid**	68.597	61	1.600	24,01	594	8,91	281	4,22	84	1,26
**Melilla**	121	0	2	2,31	0	0,00	0	0,00	0	0,00
**Murcia**	1.595	1	43	2,88	18	1,20	6	0,40	0	0,00
**Navarra**	5.238	15	129	19,72	68	10,39	21	3,21	7	1,07
**País Vasco**	13.494	2	146	6,61	46	2,08	27	1,22	8	0,36
**La Rioja**	4.048	2	29	9,15	9	2,84	8	2,53	3	0,95
**ESPAÑA**	**238.564**	**187**	**6.956**	**14,79**	**2.951**	**6,28**	**1.061**	**2,26**	**276**	**0,59**

**Casos totales confirmados por PCR hasta el 10 de mayo, y por PCR e IgM (sólo si sintomatología compatible) según la nueva estrategia de vigilancia desde el 11 de mayo.*

*** IA: Incidencia acumulada (casos diagnosticados/100.000 habitantes)*

#Se está realizando una validación individualizada de los casos por lo que puede haber discrepancias respecto a la notificación de días previos

Tabla 2. Casos de COVID-19 que han precisado hospitalización, ingreso en UCI y fallecidos (total y confecha de hospitalización/ingreso en UCI/fallecimiento en los últimos 7 días) por Comunidades Autónomas en España a 28.05.2020 (datos consolidados a las 12:00 horas del 29.05.2020).

CCAA	Casos que han precisado hospitalización		Casos que han ingresado en UCI		Fallecidos	
	Total*	*Con fecha de ingreso en los últimos 7 días*	Total*	*Con fecha de ingreso en UCI en los últimos 7 días*	Total**	*Con fecha de defunción en los últimos 7 días*
**Andalucía**	6.261	11	770	1	1.404	0
**Aragón**	2.643	14	267	1	826	2
**Asturias**	1.097	2	122	0	310	0
**Baleares**	1.163	1	165	0	209	0
**Canarias**	946	3	179	0	151	1
**Cantabria**	1.042	5	79	0	202	0
**Castilla La Mancha**	9.280	26	649	4	2.945	1
**Castilla y León**	8.576	30	547	0	1.922	4
**Cataluña**	29.254	68	2.982	2	5.584	8
**Ceuta**	12	1	4	0	4	0
**C. Valenciana**	5.739	10	735	1	1.334	3
**Extremadura**	1.766	9	110	0	507	3
**Galicia**	2.918	1	329	1	608	6
**Madrid**	41.993	89	3.544	2	8.691	5
**Melilla**	44	0	3	0	2	0
**Murcia**	680	0	112	0	148	1
**Navarra**	2.036	2	135	0	490	0
**País Vasco**	6.957	10	574	0	1.424	0
**La Rioja**	1.484	4	91	0	360	5
**ESPAÑA**	123.891	286	11.397	12	27.121	39

Los casos confirmados no provienen de la suma de pacientes hospitalizados, curados y fallecidos, ya que no son excluyentes. Pacientes fallecidos y curados pueden haber precisado hospitalización y por tanto computar en ambos grupos. Los pacientes que han precisado UCI también computan en los pacientes que han requerido hospitalización.

**Se está realizando una validación individualizada de los casos por lo que puede haber discrepancias respecto a la notificación agregada de días previos.*

*** Se está realizando una validación de los casos fallecidos que permitirá corregir la serie histórica que se actualizará semanalmente. Únicamente se suman al total de forma diaria los casos en los que consta como fecha de fallecimiento la del día previo a la hora de elaboración del presente informe.*

Tabla 3. Distribución de casos hospitalizados, ingresados en UCI y fallecidos por grupos de edad y sexo información disponible* 28.05.2020 (datos consolidados a las 12:00 horas del 29.05.2020).

Grupo de edad (años)	Total							
	Confirmados	Hospitalizados totales		UCI		Fallecidos		
	n	n	%	n	%	n	%	Letalidad(%)
0-9	1.123	286	0,3	39	0,5	3	0,0	0,3
10-19	2.068	286	0,3	24	0,3	5	0,0	0,2
20-29	15.272	1.492	1,6	89	1,1	24	0,1	0,2
30-39	24.902	3.902	4,2	275	3,5	65	0,3	0,3
40-49	37.970	8.805	9,5	740	9,3	218	1,1	0,6
50-59	45.750	14.360	15,4	1.565	19,7	663	3,2	1,4
60-69	36.355	17.559	18,8	2.542	32,0	1.825	8,9	5,0
70-79	34.294	21.002	22,5	2.274	28,7	4.896	23,8	14,3
80-89	40.637	19.014	20,4	332	4,2	8.463	41,1	20,8
90 y +	20.389	6.452	6,9	56	0,7	4.423	21,5	21,7
Total	258.760	93.158	100%	7.936	100%	20.585	100%	

Grupo de edad (años)	Mujeres							
	Confirmados	Hospitalizados totales		UCI		Fallecidos		
	n	n	%	n	%	n	%	Letalidad(%)
0-9	435	125	0,3	12	0,5	1	0,0	0,2
10-19	955	132	0,3	10	0,4	2	0,0	0,2
20-29	9.582	771	1,9	39	1,6	9	0,1	0,1
30-39	14.902	1.887	4,6	105	4,3	21	0,2	0,1
40-49	21.495	3.621	8,9	228	9,3	77	0,9	0,4
50-59	25.153	5.858	14,4	452	18,5	192	2,2	0,8
60-69	16.937	6.858	16,9	726	29,7	539	6,0	3,2
70-79	15.081	8.431	20,7	693	28,3	1.564	17,5	10,4
80-89	23.156	9.108	22,4	146	6,0	3.792	42,5	16,4
90 y +	14.269	3.860	9,5	35	1,4	2.723	30,5	19,1
Total	141.965	40.651	100%	2.446	100%	8.920	100%	

Grupo de edad (años)	Hombres							
	Confirmados	Hospitalizados totales		UCI		Fallecidos		
	n	n	%	n	%	n	%	Letalidad(%)
0-9	504	147	0,3	27	0,5	1	0,0	0,2
10-19	806	139	0,3	13	0,2	3	0,0	0,4
20-29	4.689	689	1,3	50	0,9	15	0,1	0,3
30-39	8.771	1.943	3,8	169	3,1	42	0,4	0,5
40-49	14.829	5.104	9,9	512	9,3	140	1,2	0,9
50-59	18.892	8.366	16,3	1.112	20,3	467	4,0	2,5
60-69	18.363	10.565	20,5	1.814	33,1	1.283	11,0	7,0
70-79	18.406	12.374	24,1	1.578	28,8	3.326	28,6	18,1
80-89	16.363	9.645	18,8	186	3,4	4.655	40,1	28,4
90 y +	5.372	2.447	4,8	21	0,4	1.681	14,5	31,3
Total	106.995	51.419	100%	5.482	100%	11.613	100%	

**Datos obtenidos del análisis sobre 248.960 casos notificados con información disponible de grupo de edad y sexo. Los datos del total pueden no coincidir con la suma de hombres y mujeres porque en algunos casos no se dispone de la información del sexo, solo de la edad. Estos datos se actualizan permanentemente, pudiendo sufrir alguna modificación. Fuente: elaboración propia.*

Figura 1. Casos diarios confirmados de COVID-19 en España a 28.05.2020 (datos consolidados a las 12:00 horas del 29.05.2020).

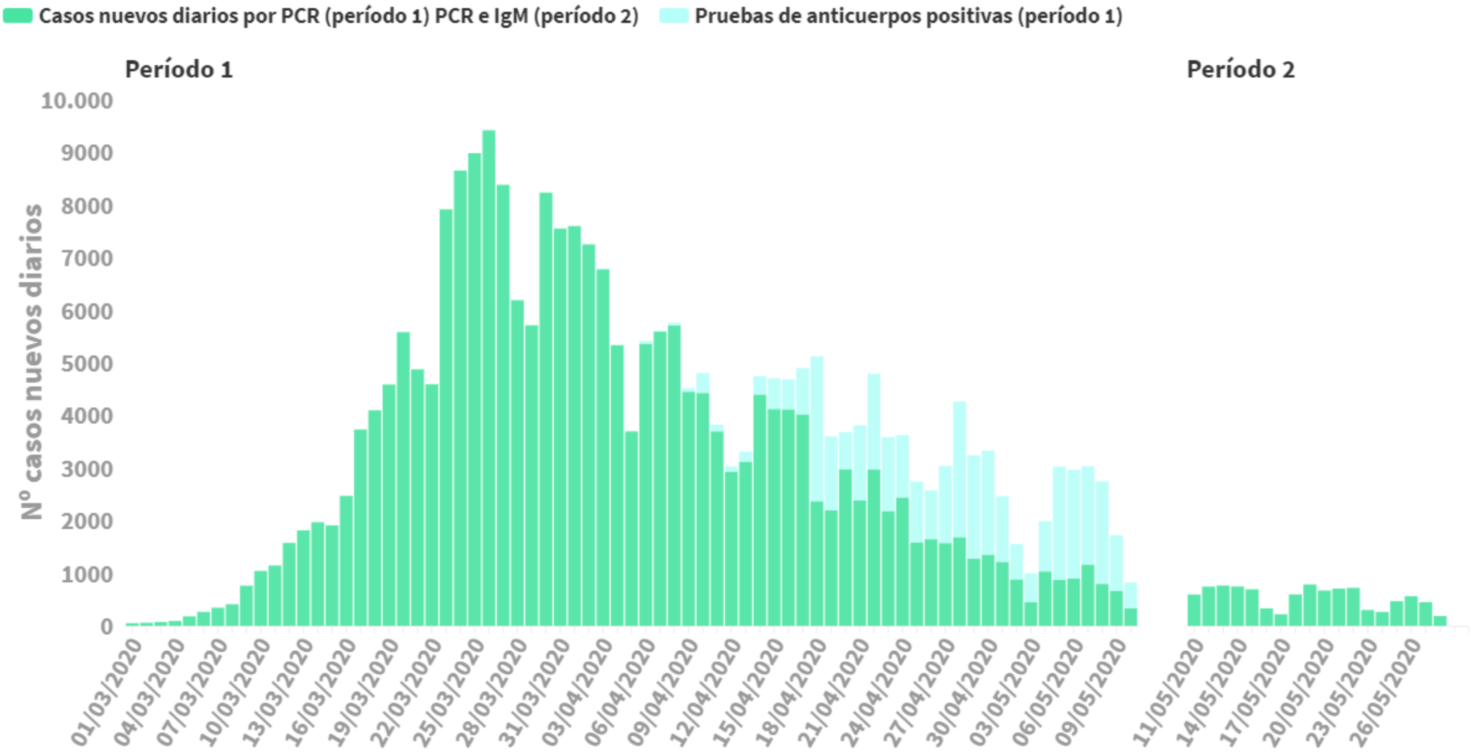

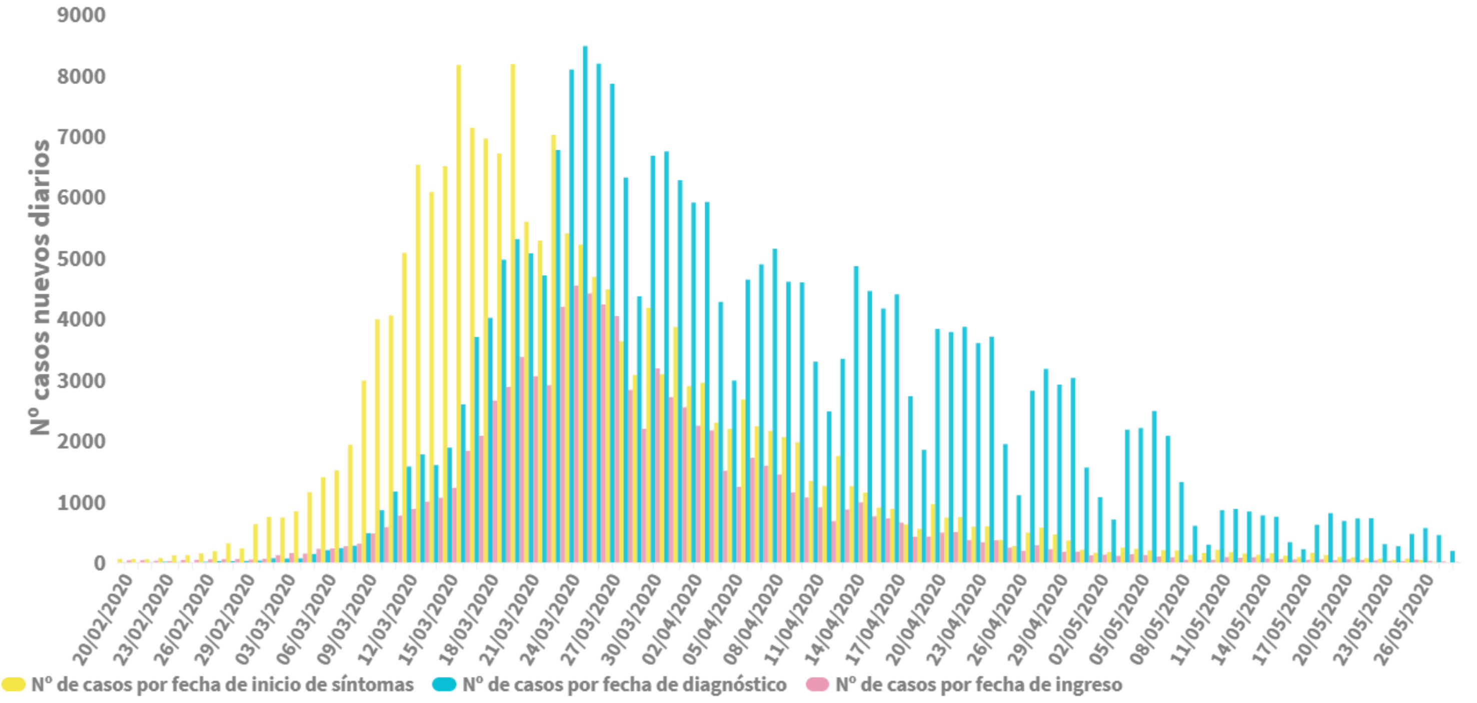

**Se dispone de fecha de inicio de síntomas en 179.347 casos, de diagnóstico en 243.782 casos y de ingreso en 87.629 casos.*

Estos datos se actualizan permanentemente, pudiendo sufrir alguna modificación.

En la primera imagen se muestran los casos nuevos según fecha de notificación durante el período 1 (del 1 de marzo al 10 de mayo de 2020) y casos por fecha de diagnóstico durante el periodo 2 (a partir del 11 de mayo) con la entrada en vigor del nuevo protocolo de vigilancia (Estrategia de Diagnóstico, Vigilancia y Control en la Fase de Transición de la Pandemia de COVID-19 Indicadores de seguimiento: https://www.mscbs.gob.es/profesionales/saludPublica/ccayes/alertasActual/nCov-China/documentos/COVID19_Estrategia_vigilancia_y_control_e_indicadores.pdf).

En la segunda imagen se reflejan los casos según la fecha de inicio de síntomas, fecha de diagnóstico y fecha de ingreso.

Figura 2. Casos diarios confirmados de COVID-19 por fecha de notificación (período 1) y fecha de diagnóstico (período 2) por Comunidades Autónomas, 28.05.2020 (datos consolidados a las 12:00 horas del 29.05.2020).

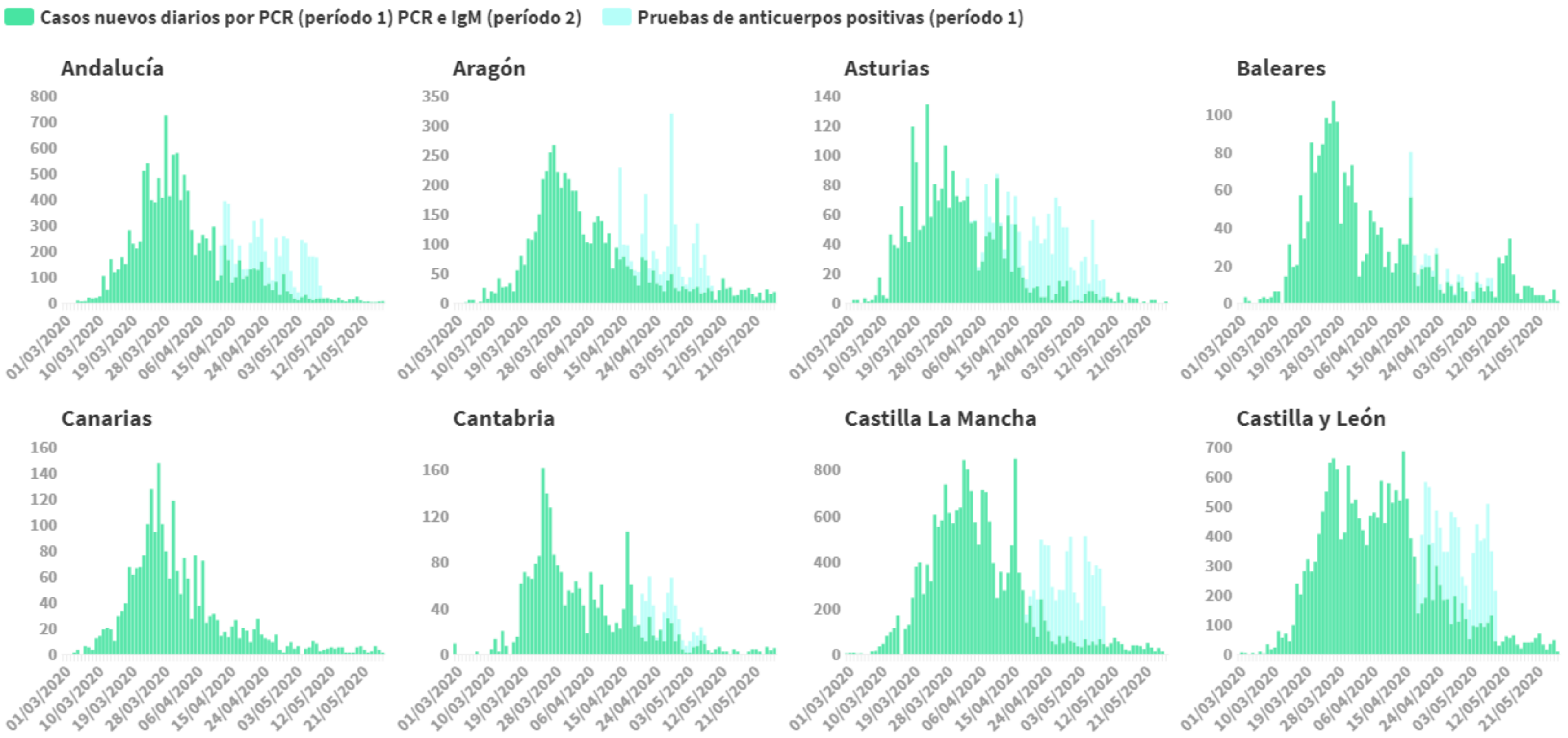

Se muestran los casos nuevos según fecha de notificación durante el período 1 (del 1 de marzo al 10 de mayo de 2020) y casos por fecha de diagnóstico durante el periodo 2 (a partir del 11 de mayo) con la entrada en vigor del nuevo protocolo de vigilancia (Estrategia de Diagnóstico, Vigilancia y Control en la Fase de Transición de la Pandemia de COVID-19 Indicadores de seguimiento: https://www.mscbs.gob.es/profesionales/saludPublica/ccayes/alertasActual/nCov-China/documentos/COVID19_Estrategia_vigilancia_y_control_e_indicadores.pdf).

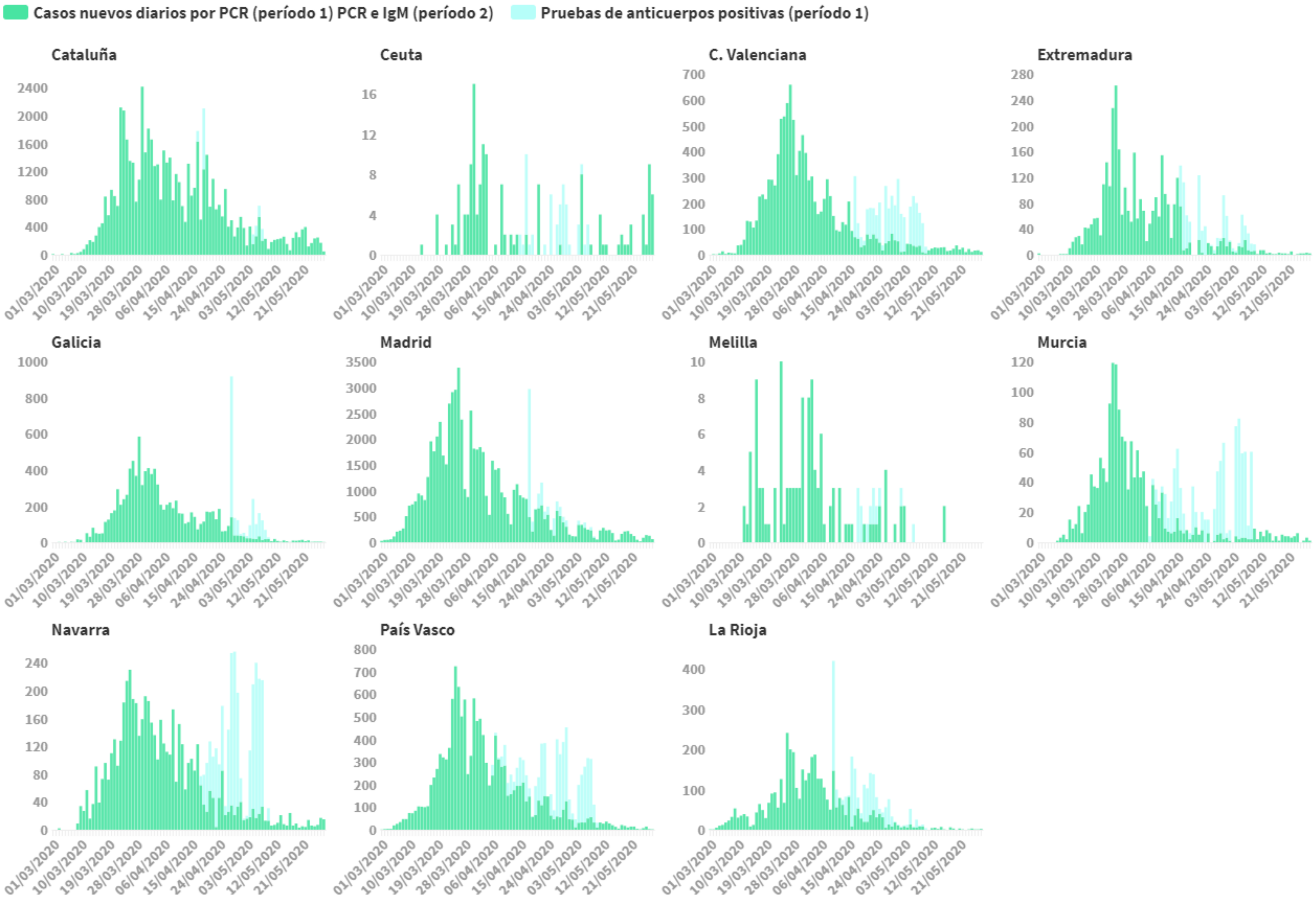

Se muestran los casos nuevos según fecha de notificación durante el período 1 (del 1 de marzo al 10 de mayo de 2020) y casos por fecha de diagnóstico durante el periodo 2 (a partir del 11 de mayo) con la entrada en vigor del nuevo protocolo de vigilancia (Estrategia de Diagnóstico, Vigilancia y Control en la Fase de Transición de la Pandemia de COVID-19 Indicadores de seguimiento: https://www.mscbs.gob.es/profesionales/saludPublica/ccayes/alertasActual/nCov-China/documentos/COVID19_Estrategia_vigilancia_y_control_e_indicadores.pdf).

PERSONAL SANITARIO

Hasta el 28.05.2020 se han notificado 51.482 casos confirmados en personal sanitario. A continuación se reflejan los datos recogidos según la nueva estrategia de vigilancia desde el 11.05.2020

Tabla 4. Casos confirmados en personal sanitario desde el 11.05.2020 por Comunidades Autónomas, a 28.05.2020 (datos consolidados a las 12:00 horas del 29.05.2020).

	Total notificados	Diagnosticados en los últimos 7 días	Diagnosticados en los últimos 14 días	Con fecha de inicio de síntomas en los últimos 7d.	Con fecha de inicio de síntomas en los últimos 14d.
**Andalucía**	20	2	11	0	2
**Aragón**	49	4	27	1	5
**Asturias**	13	3	7	1	3
**Baleares**	15	1	6	0	1
**Canarias**	14	2	6	0	1
**Cantabria**	6	0	0	0	0
**Castilla La Mancha**	152	23	95	1	7
**Castilla y León**	61	18	44	1	5
**Cataluña**	416	95	302	8	49
**Ceuta**	4	2	3	2	3
**C. Valenciana**	24	9	14	2	3
**Extremadura**	3	0	1	0	1
**Galicia**	31	8	23	0	6
**Madrid**	168	30	84	3	13
**Melilla**					
**Murcia**	7	2	3	0	0
**Navarra**	27	9	22	1	2
**País Vasco**	49	3	19	1	3
**La Rioja**	3	1	3	1	1
**ESPAÑA**	1062	212	670	22	105

Tabla 5. Ámbito de exposición en personal sanitario en función del centro de trabajo desde el 11.05.2020 a 28.05.2020 (datos consolidados a las 12:00 horas del 29.05.2020).

Ambito de exposición	Centro de trabajo			Total
	Centro Sanitario	Centro sociosanitario	*Otro centro*	
**Centro Sanitario**	208	5	5	218
**Centro Sociosanitario**	26	168	1	195
**Domicilio**	27	19	10	56
**Laboral**	62	67	32	161
**Otros**	6	8	16	30
**Desconocido**	315	54	33	402
**ESPAÑA**	644	321	97	1062

SITUACIÓN INTERNACIONAL *(datos consultados a las 12:30 horas del 29.05.2020)*

Situación en Europa: Hasta el día de hoy se han notificado al menos 2.068.332 casos confirmados (Tabla 6). Los países con más casos notificados son Rusia (387.623), Reino Unido (269.127), España (238.564), Italia (231.732) y Alemania (180.458). El país con mayor número de fallecidos es Reino Unido (37.837) seguido de Italia (33.142) y Francia (28.662). (Tabla 7 y Figura 3). En Italia, las regiones con más casos confirmados son Lombardía (88.183), seguida de Piemonte (30.445) y Emilia Romagna (27.701). En Alemania, las regiones más afectadas son Bayern (46.809), Nordrhein-Westfalen (37.803) y Baden-Württemberg (34.479). Inglaterra (151.422) y Escocia (15.288) son las partes más afectadas de Reino Unido.

Tabla 6. Casos confirmados de COVID-19 en Europa[1]

	Casos		Casos		Casos		Casos
Rusia	387.623	Polonia	22.964	Grecia	2.906	Georgia	738
Reino Unido	269.127	Ucrania	22.811	Bulgaria	2.485	San Marino	670
España	238.564	Rumanía	18.982	Bosnia y Herzegovina	2.462	Malta	616
Italia	231.732	Austria	16.564	Croacia	2.245	Montenegro	324
Alemania	180.458	Dinamarca	11.512	Macedonia del Norte	2.078	Islas Feroe	187
Turquía	160.979	Serbia	11.300	Estonia	1.851	Gibraltar	157
Francia	149.071	Chequia	9.140	Islandia	1.805	Mónaco	98
Bélgica	57.849	Noruega	8.401	Lituania	1.656	Liechtenstein	83
Países Bajos	45.950	Armenia	8.216	Eslovaquia	1.520		
Bielorrusia	39.858	República de Moldavia	7.725	Eslovenia	1.473		
Suecia	35.727	Finlandia	6.743	Albania	1.076		
Portugal	31.596	Azerbaijan	4.759	Letonia	1.061		
Suiza	30.796	Luxemburgo	4.008	Chipre	941		
Irlanda	24.841	Hungría	3.841	Andorra	763		

[1] *Datos consultados (orden alfabético): **Alemania** (Robert Koch Institut), **Austria** (Ministerio de sanidad), **Bélgica** (Service Public Fédéral Sante Publique,Securite de la Chaine Alimentaire et Environnement), **Bielorrusia** (Ministerio de Sanidad), **Francia** (Santé Publique France), **Irlanda** (Ministerio de Sanidad), **Italia** (Ministerio de Sanidad), **Países Bajos** (National Institute for Public Health and the Environment), **Polonia** (Ministerio de Sanidad), **Portugal** (Dirección General de Salud), **Reino Unido** (Public Health England), **Rumanía** (Instituto Nacional de Salud Pública), **Rusia** (Ministerio de Sanidad), **Suecia** (Public Health Agency of Sweden), **Suiza** (Office Fédéral de la Santé Publique), **Turquía** (Ministerio de Sanidad), **Ucrania** (Cabinet of Ministers of Ucraine).**Resto de países**: Página web de la Oficina Regional para Europa de la Organización Mundial de la Salud (OMS) y Centro Europeo de Prevención y Control de Enfermedades (ECDC)*

Tabla 7. Detalles de los quince países con más casos confirmados de Europa[2,3,4]

	Casos confirmados	Nuevos diarios	*Incremento diario*	Incidencia Acumulada 100.000 hab. 14 días	Fallecidos	Nuevos diarios	Letalidad
**Rusia**	387.623	*8.572*	*2,26%*	93,7	4.374	*232*	1,1%
**Reino Unido**	269.127	*1.887*	*0,71%*	54,0	37.837	*377*	14,1%
**España**	238.564	*187*		14,8	27.121		
**Italia**	231.732	*593*	*0,26%*	14,3	33.142	*70*	14,3%
**Alemania**	180.458	*741* *		9,9	8.450	*39*	4,7%
**Turquía**	160.979	*1.182*	*0,74%*	19,8	4.461	*30*	2,8%
**Francia**	149.071	*3.325* *		11,5	28.662	*66*	19,2%
**Bélgica**	57.849	*257*	*0,45%*	31,1	9.388	*24*	16,2%
**Países bajos**	45.950	*182*	*0,40%*	14,3	5.903	*32*	12,8%
**Bielorrusia**	39.858	*902*	*2,32%*	147,9	219	*5*	0,5%
**Suecia**	35.727	*639*	*1,82%*	65,3	4.266	*46*	11,9%
**Portugal**	31.596	*304*	*0,97%*	31,9	1.369	*13*	4,3%
**Suiza**	30.796			3,9	1.655		5,4%
**Irlanda**	24.841	*38*	*0,15%*	20,7	1.639	*8*	6,6%
**Polonia**	22.964	*364*	*1,61%*	14,1	1.043	*13*	4,5%
**Austria**	16.564	*23*	*0,14%*	6,2	668	*0*	4,0%

[2] *Las cifras de fallecidos por COVID-19 notificadas por cada país varían en su definición por lo que dichas cifras, y la letalidad resultante en cada país, no son directamente comparables entre sí.*
[3] *Austria ya no se encuentra entre los quince países con más casos confirmados de Europa. Se sigue incluyendo en la tabla por su relevancia en el histórico de la epidemia*
[4] *Suiza no ha actualizado datos a las 12:30 del 29/05/2020*
* *Alemania y Francia han reajustado datos por lo que sus incrementos de hoy no reflejan el incremento real de las últimas 24 horas*

Figura 3. Casos nuevos confirmados de COVID-19 por fecha de notificación en Rusia, Reino Unido, Italia, Alemania, Turquía y Francia.[5]

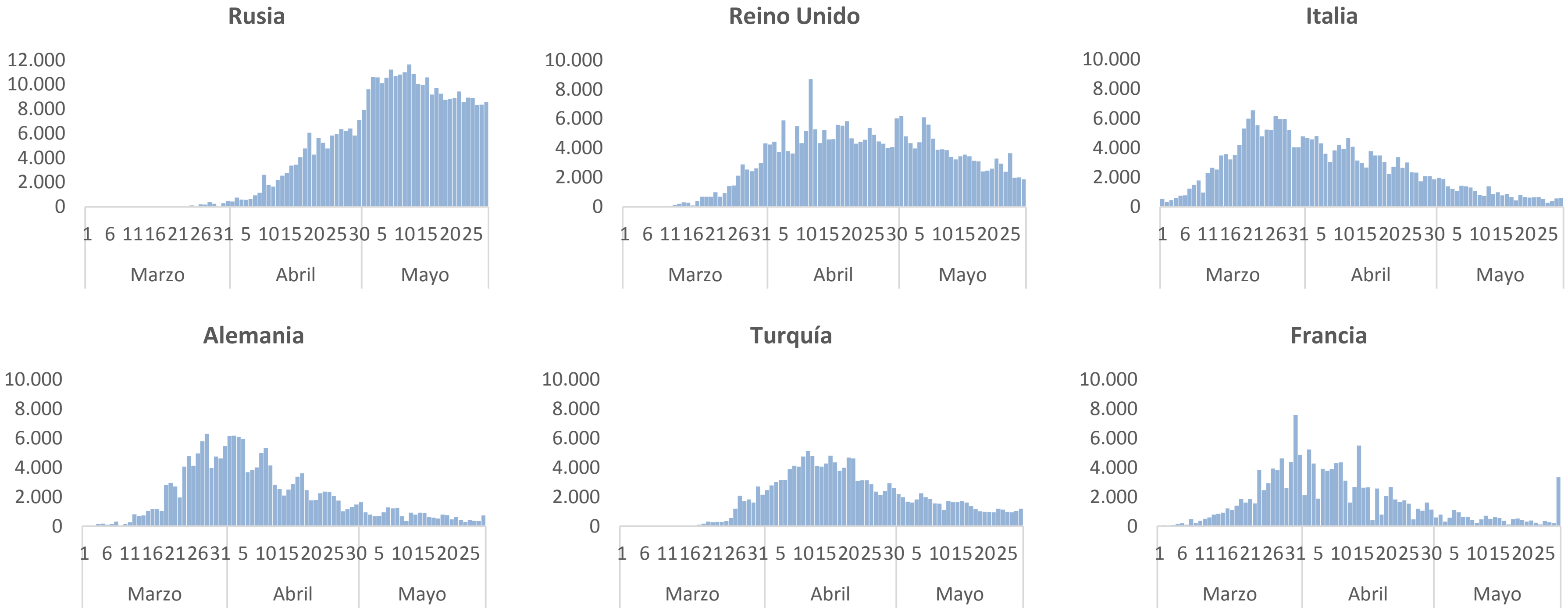

[5] *Elaboración propia usando los datos disponibles en las páginas web de las autoridades sanitarias de los propios países.*

Situación a nivel global y otros países fuera de Europa: Según OMS, a nivel global hasta el día de hoy se han notificado al menos 5.657.529 casos (104.917 casos más que ayer) y 356.254 fallecidos. Los países de fuera de Europa que han registrado más casos son Estados Unidos (1.698.523), Brasil (437.238), India (165.799) e Irán (141.591) (Tabla 8, Tabla 9 y Figura 4).

Tabla 8. Casos confirmados de COVID-19 fuera de Europa. Se incluyen los quince países con más casos confirmados en cada continente.[6]

América	Casos	África	Casos	Asia-Oceanía	Casos
Estados Unidos	1.698.523	Sudáfrica	27.403	India	165.799
Brasil	437.238	Egipto	20.793	Irán	141.591
Perú	141.779	Argelia	8.997	China	84.547
Canadá	88.512	Nigeria	8.915	Arabia Saudí	78.541
Chile	86.943	Marruecos	7.643	Pakistán	64.028
México	81.400	Ghana	7.303	Qatar	50.914
Ecuador	38.471	Camerún	5.436	Bangladesh	42.844
Colombia	25.366	Sudan	4.346	Singapur	33.249
República Dominicana	16.068	Guinea	3.553	Emiratos Árabes Unidos	32.532
Argentina	14.689	Senegal	3.348	Indonesia	24.538
Panamá	12.131	Djibouti	2.914	Kuwait	24.112
Bolivia	8.387	R. D. del Congo	2.832	Israel	16.809
Honduras	4.752	Costa de Marfil	2.641	Japón	16.683
Guatemala	4.348	Gabón	2.431	Filipinas	15.588
Puerto Rico	3.486	Somalia	1.828	Afganistán	13.036
Casos confirmados a nivel global: 5.657.529					
Fallecidos: 356.254					

[6] *Datos consultados (orden alfabético):* ***Arabia Saudí*** *(OMS),* ***Bangladesh*** *(Ministerio de Sanidad),* ***Brasil*** *(Ministerio de Sanidad),* ***Canadá*** *(Gobierno de Canadá),* ***Chile*** *(Ministerio de Sanidad),* ***China*** *(Chinese Center for Disease Control and Prevention),* ***Corea del Sur*** *(Ministerio de Sanidad),* ***Ecuador*** *(Ministerio de Sanidad),* ***Emiratos Árabes Unidos*** *(Ministerio de Sanidad),* ***Estados Unidos*** *(Centers for Disease Control and Prevention),* ***India*** *(Ministerio de Sanidad),* ***Irán*** *(OMS),* ***Israel*** *(Ministerio de Sanidad),* ***Japón*** *(Ministerio de Sanidad),* ***México*** *(Ministerio de Sanidad),* ***Pakistán*** *(Ministerio de Sanidad),* ***Perú*** *(Ministerio de Sanidad),* ***Qatar*** *(Ministerio de Sanidad).*
Resto de países*: páginas web de la Organización Mundial de la Salud (OMS) y en el Centro Europeo de Prevención y Control de Enfermedades (ECDC)*

Tabla 9. Detalles de los quince países con más casos confirmados fuera de Europa[7],[8]

	Casos confirmados	Nuevos diarios	*Incremento diario*	Incidencia Acumulada 100.000 hab. 14 días	Fallecidos	Nuevos diarios	Letalidad
**Estados Unidos**	1.698.523	*19.680*	*1,17%*	95,5	100.446	*1.415*	5,9%
**Brasil**	437.238	*25.417*	*6,17%*	111,8	26.754	*1.156*	6,1%
**India**	165.799	*7.466*	*4,72%*	6,2	4.706	*175*	2,8%
**Perú**	141.779	*5.874*	*4,32%*	191,2	4.099	*116*	2,9%
**Irán**	141.591	*2.080*	*1,49%*	33,3	7.564	*56*	5,3%
**Canadá**	88.512	*993*	*1,13%*	40,2	6.877	*112*	7,8%
**Chile**	86.943	*4.654*	*5,66%*	266,4	890	*49*	1,0%
**China**	84.547	*0*	*0,00%*	0,0	4.645	*0*	5,5%
**México**	81.400	*3.377*	*4,33%*	30,7	9.044	*447*	11,1%
**Arabia Saudí**	78.541	*1.815*	*2,37%*	96,0	425	*14*	0,5%
**Pakistán**	64.028	*2.801*	*4,57%*	12,6	1.317	*57*	2,1%
**Qatar**	50.914	*1.967*	*4,02%*	813,9	33	*3*	0,1%
**Bangladesh**	42.844	*2.523*	*6,26%*	15,5	582	*23*	1,4%
**Ecuador**	38.471	*368*	*0,97%*	46,7	3.313	*38*	8,6%
**Singapur**	33.249	*373*	*1,13%*	126,8	23	*0*	0,1%
**Japón**	16.683	*41*	*0,25%*	0,5	867	*9*	5,2%
**Corea del Sur**	11.402	*58*	*0,51%*	0,7	269	*0*	2,4%

[7] *Japón y Corea del Sur ya no se encuentran entre los quince países con más casos confirmados fuera de Europa. Se siguen incluyendo en la tabla por su relevancia en el histórico de la epidemia*
[8] *Las cifras de fallecidos por COVID-19 notificadas por cada país varían en su definición por lo que dichas cifras, y la letalidad resultante en cada país, no son directamente comparables entre sí.*

Figura 4. Casos nuevos confirmados de COVID-19 por fecha de notificación en Estados Unidos, Brasil e India.[9]

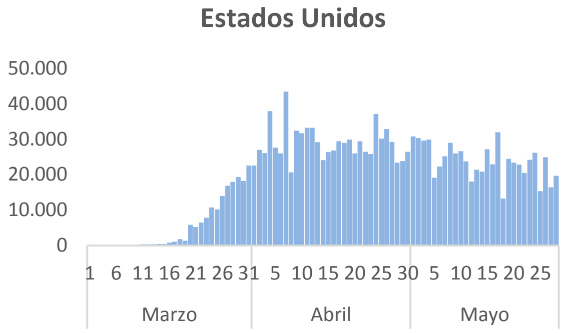

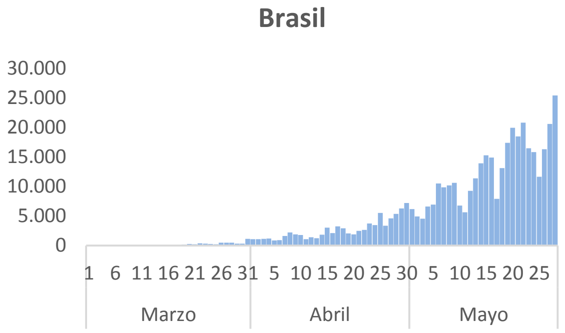

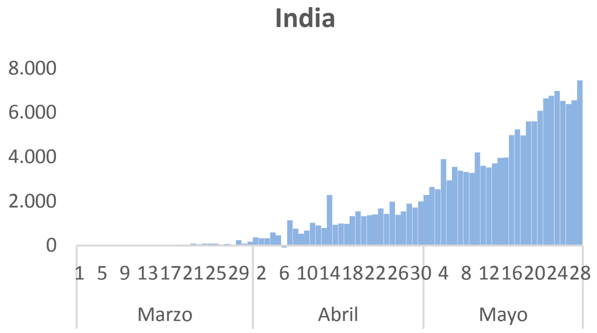

[9] *Elaboración propia usando los datos disponibles en las páginas web de las autoridades sanitarias de los propios países y OMS.*

Epidemia COVID-19

Aggiornamento nazionale
3 giugno 2020 – ore 15:00

DATA PUBBLICAZIONE: 5 GIUGNO 2020

Prodotto dall'Istituto Superiore di Sanità (ISS), Roma

A cura di: Flavia Riccardo, Xanthi Andrianou, Antonino Bella, Martina Del Manso, Alberto Mateo Urdiales, Massimo Fabiani, Stefania Bellino, Stefano Boros, Fortunato (Paolo) D'Ancona, Maria Cristina Rota, Antonietta Filia, Ornella Punzo, Andrea Siddu, Maria Fenicia Vescio, Corrado Di Benedetto, Marco Tallon, Alessandra Ciervo, Maria Rita Castrucci, Patrizio Pezzotti, Paola Stefanelli, Annalisa Pantosti per ISS,

Giorgio Guzzetta, Valentina Marziano, Piero Poletti, Filippo Trentini, Marco Ajelli, Stefano Merler per Fondazione Bruno Kessler;

e di: Antonia Petrucci (Abruzzo); Michele La Bianca (Basilicata); Anna Domenica Mignuoli (Calabria); Pietro Buono (Campania); Erika Massimiliani (Emilia-Romagna); Tolinda Gallo (Friuli Venezia Giulia); Paola Scognamiglio (Lazio); Camilla Sticchi (Liguria); Danilo Cereda (Lombardia); Lucia Di Furia (Marche); Francesco Sforza (Molise); Maria Grazia Zuccaro (P.A. Bolzano); Pier Paolo Benetollo (P.A. Trento); Daniela Tiberti (Piemonte); Maria Chironna (Puglia); Maria Antonietta Palmas (Sardegna); Salvatore Scondotto (Sicilia); Emanuela Balocchini (Toscana); Anna Tosti (Umbria); Mauro Ruffier (Valle D'Aosta); Filippo Da Re (Veneto).

EPIDEMIA COVID-19

Aggiornamento nazionale

3 giugno 2020 – ore 15:00

Nota di lettura: *Questo bollettino è prodotto dall'Istituto Superiore di Sanità (ISS) ed integra dati microbiologici ed epidemiologici forniti dalle Regioni e dal Laboratorio Nazionale di Riferimento per SARS-CoV-2 dell'ISS. I dati vengono raccolti attraverso una piattaforma web dedicata e riguardano tutti i casi di COVID-19 diagnosticati dai laboratori di riferimento regionali. I dati vengono aggiornati giornalmente da ciascuna Regione anche se alcune informazioni possono richiedere qualche giorno per il loro inserimento. Per questo motivo, potrebbe non esserci una completa concordanza con quanto riportato attraverso il flusso informativo della Protezione Civile e del Ministero della Salute che riportano dati aggregati.*

I dati raccolti sono in continua fase di consolidamento e, come prevedibile in una situazione emergenziale, alcune informazioni sono incomplete. In particolare, si segnala, soprattutto nelle Regioni in cui si sta verificando una trasmissione locale sostenuta del virus, la possibilità di un ritardo di alcuni giorni tra il momento della esecuzione del tampone per la diagnosi e la segnalazione sulla piattaforma dedicata. Pertanto, il numero casi che si osserva negli ultimi due giorni (Figura 1), deve essere al momento interpretata come provvisorio.

Il bollettino descrive, con grafici, mappe e tabelle la diffusione, nel tempo e nello spazio, dell'epidemia di COVID-19 in Italia. Fornisce, inoltre, una descrizione delle caratteristiche delle persone affette.

Prodotto dall'Istituto Superiore di Sanità (ISS), Roma, 3 giugno 2020

La situazione nazionale

- Dall'inizio dell'epidemia alle ore 15 del 3 giugno 2020, sono stati riportati al sistema di sorveglianza 234.119 casi di COVID-19 diagnosticati dai laboratori di riferimento regionale come positivi per SARS-CoV–2 (3.089 casi in più rispetto al 27 maggio 2020). Sono stati notificati 32.354 decessi (632 decessi in più rispetto al 27 maggio 2020). **Complessivamente si continua ad osservare un decremento nel numero di nuovi casi diagnosticati nell'ultima settimana rispetto alle settimane precedenti.**

- La Figura 1 mostra l'andamento del numero di casi di COVID-19 segnalati per data di prelievo/diagnosi (disponibile per 231.617/234.119 casi). La curva epidemica mostra un decremento costante anche se le diagnosi più recenti potrebbero essere sottostimate a causa di un ritardo nella notifica (particolarmente per i giorni nel box grigio).

- La data di inizio sintomi è al momento disponibile solo in 177.108 dei 234.119 casi segnalati. Questo può essere dovuto al fatto che una parte dei casi diagnosticati è asintomatico e/o dal consolidamento del dato ancora in corso. La Figura 2 mostra la distribuzione dei casi per data inizio dei sintomi.

- Il tempo mediano trascorso tra la data di insorgenza dei primi sintomi e la data di diagnosi è di 4 giorni per il periodo 20 febbraio al 10 marzo (calcolato su 13.377 casi), di 5 giorni per il periodo 11-20 marzo (37.473 casi), di 6 giorni dal 21 marzo al 30 marzo (40.956 casi), di 5 giorni dal 31 marzo al 9 maggio (32.059 casi), di 4 giorni dal 10 aprile al 19 aprile (20.835 casi), di 5 giorni dal 290 aprile al 9 maggio (24.098 casi), di nuovo di 4 giorni dal 10 al 29 maggio (7.395 casi) ed infine di 10 giorni dal 30 maggio al 3 giugno(257 casi). L'aumento nel tempo mediano registrato nell'ultimo periodo, potrebbe essere dovuto all'intensificarsi delle attività di screening con identificazione di soggetti positivi non più sintomatici con inizio sintomi riferito settimane o mesi addietro.

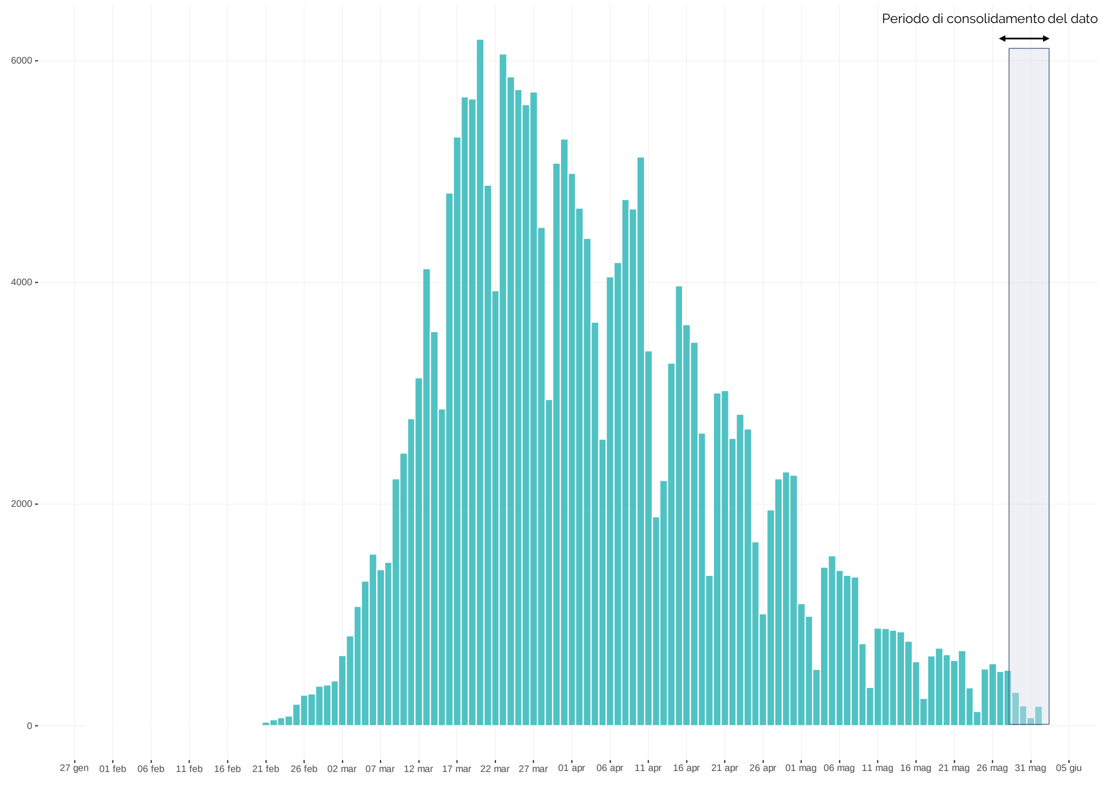

FIGURA 1 – CASI DI COVID-19 DIAGNOSTICATI DAI LABORATORI DI RIFERIMENTO REGIONALE, PER DATA PRELIEVO/DIAGNOSI (N=231.617).

Nota I dati più recenti devono essere considerati provvisori (vedere soprattutto riquadro grigio)

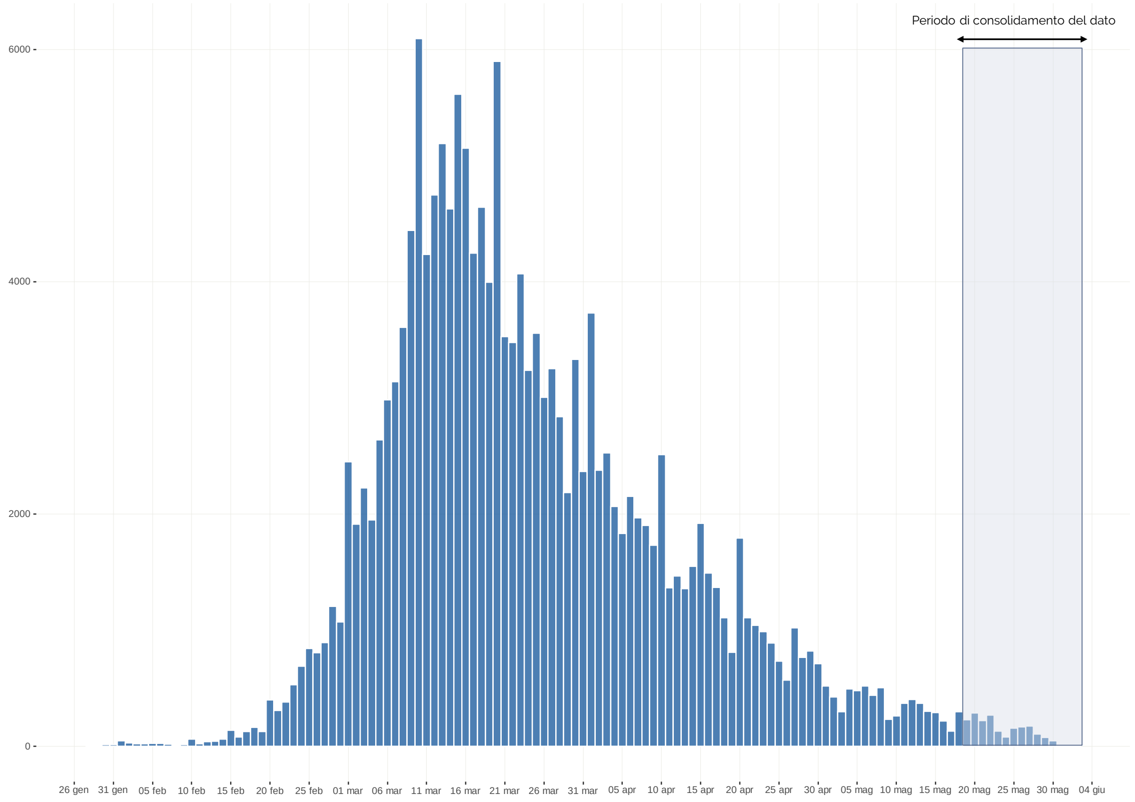

FIGURA 2 – CASI DI COVID-19 DIAGNOSTICATI DAI LABORATORI DI RIFERIMENTO REGIONALE, PER DATA INIZIO SINTOMI (N=177.108).

Nota: i dati più recenti devono essere considerati provvisori sia per il ritardo di notifica sia perché casi non ancora diagnosticati riporteranno in parte la data di inizio sintomi nei giorni del riquadro grigio.

- 107.368 casi sono di sesso maschile (45,9%). L'età mediana è di 62 anni (range 0-100).

- La Tabella 1 mostra la distribuzione dei casi e dei decessi segnalati per sesso e fasce di età decennali. L'informazione sul sesso è nota per 234.086/234.119 casi. Nelle fasce di età 0-9, 10-19, 60-69 e 70-79 anni si osserva un numero maggiore di casi di sesso maschile rispetto al numero di casi di sesso femminile. Nella fascia di età >90 anni, il numero di soggetti di sesso femminile è più del triplo di quello dei soggetti di sesso maschile, probabilmente a causa della struttura demografica della popolazione.

- Nella Tabella 1 si osserva un aumento della letalità con l'aumentare dell'età dei casi. La letalità è più elevata in soggetti di sesso maschile in tutte le fasce di età, ad eccezione della fascia 0-9 anni.

- Nel 34,9% dei casi segnalati viene riportata almeno una co-morbidità (patologie cardiovascolari, patologie respiratorie, diabete, deficit immunitari, patologie metaboliche, patologie oncologiche, obesità, patologie renali o altre patologie croniche).

- Attualmente la gravità clinica è disponibile per 31.845 casi attualmente positivi, di cui 9.102 (28,6%) asintomatici, 3.936 (12,4%) pauci-sintomatici, 1.859 (5,8%) con sintomi per cui non è specificato il livello di gravità, 11.097 (34,8%) con sintomi lievi, 5.155 (16,2%) con sintomi severi tali da richiedere ospedalizzazione, 696 (2,2%) con quadro clinico di gravità critica che richiede ricovero in Terapia Intensiva. Al 20 maggio risultano guariti 140.678 casi.

TABELLA 1 - DISTRIBUZIONE DEI CASI DIAGNOSTICATI DAI LABORATORI DI RIFERIMENTO REGIONALE (N=234.718) E DEI DECESSI SEGNALATI (N=32.354) PER FASCIA DI ETÀ E SESSO

Classe di età (anni)	Soggetti di sesso maschile					Soggetti di sesso femminile					Casi totali				
	N. casi	% casi totali	N. deceduti	% del totale deceduti	Letalità %	N. casi	% casi totali	N. deceduti	% del totale deceduti	Letalità %	N. casi	% casi per classe di età	N. deceduti	% deceduti per classe di età	Letalità %
0-9	1.047	52,7	1	20,0	0,1	938	47,3	4	80,0	0,4	1.986	0,8	5	0,0	0,3
10-19	1.799	50,4	0	0,0	0,0	1771	49,6	0	0,0	0,0	3.570	1,5	0	0,0	0,0
20-29	5.826	44,0	11	73,3	0,2	7416	56,0	4	26,7	0,1	13.250	5,7	15	0,0	0,1
30-39	8.187	44,8	41	65,1	0,5	10.094	55,2	22	34,9	0,2	18.287	7,8	63	0,2	0,3
40-49	12.722	41,8	203	72,8	1,6	17.720	58,2	76	27,2	0,4	30.444	13,0	279	0,9	0,9
50-59	19.454	46,3	869	76,7	4,5	22.580	53,7	264	23,3	1,2	42.038	18,0	1.133	3,5	2,7
60-69	18.694	59,8	2.528	76,4	13,5	12.591	40,2	779	23,6	6,2	31.286	13,4	3.307	10,2	10,6
70-79	19.184	57,3	6.071	70,0	31,6	14.289	42,7	2.606	30,0	18,2	33.474	14,3	8.677	26,8	25,9
80-89	16.590	40,6	7.431	56,2	44,8	24.303	59,4	5.802	43,8	23,9	40.901	17,5	13.233	40,9	32,4
>90	3.846	20,4	1.868	33,1	48,6	14.990	79,6	3.773	66,9	25,2	18.837	8,0	5.641	17,4	29,9
Età non nota	19	42,2	1	100,0	5,3	26	57,8	0	0,0	0,0	46	0,0	1	0,0	2,2
**Totale**	107.368		19.024		17,7	126.718		13.330		10,5	234.119		32.354		13,8

NOTA: LA TABELLA NON INCLUDE I CASI CON SESSO NON NOTO

- L'informazione sul luogo di trattamento è disponibile per 155.853 casi (66,5% del totale), di cui 13.261 (5,7%) risultano ospedalizzati. Di questi ultimi, 1.180 (8,8%) sono ricoverati in Terapia Intensiva. Attualmente, questo dato non è classificato in tutte le Regioni/PPAA in modo standardizzato, secondo le modalità previste dalla sorveglianza COVID-19, ma si sta procedendo alla raccolta di tale informazione. Pertanto, i dati sullo stato clinico e sul reparto di degenza saranno soggetti a modifiche dovute al loro progressivo consolidamento.

- La Figura 3 mostra i dati cumulativi, riportati dal Ministero della Salute e dalla Protezione Civile al 3 giugno 2020, sulla condizione di ricovero e isolamento e sugli esiti dei casi di COVID-19 diagnosticati dai laboratori di riferimento regionale. **Si sottolinea che il numero di casi riportato in Figura 3 è un numero aggregato riportato giornalmente dalle Regioni/PPAA attraverso il flusso informativo della Protezione Civile e del Ministero della Salute e, pertanto, differisce dal dato individuale (soggetto a un maggior ritardo di notifica) riportato nella piattaforma della sorveglianza Covid-19.**

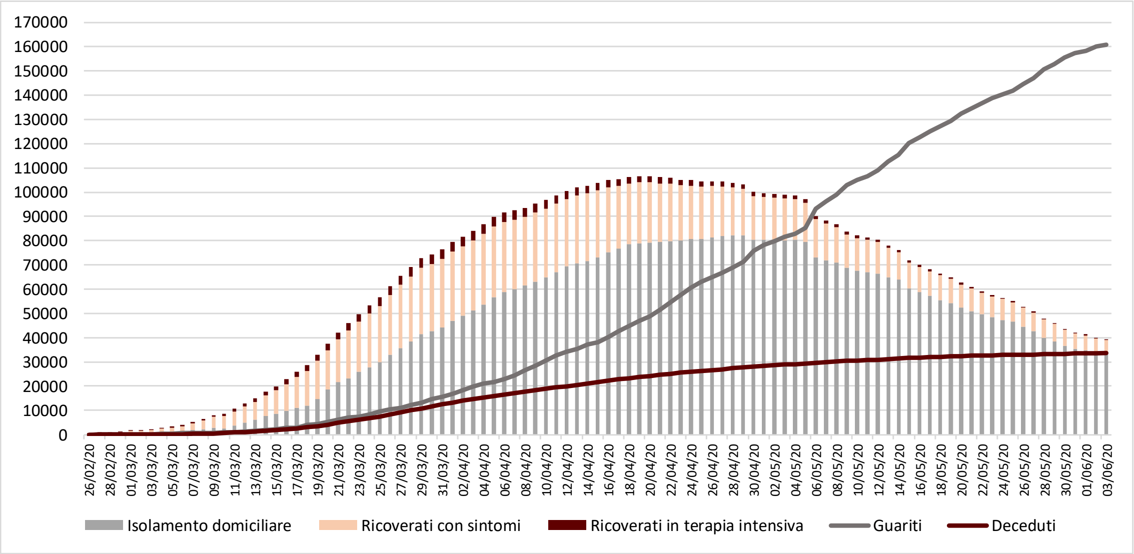

FIGURA 3 – NUMERO CUMULATIVO DI CASI DI COVID-19 DIAGNOSTICATI DAI LABORATORI DI RIFERIMENTO REGIONALE, PER STATO DI RICOVERO/ISOLAMENTO ED ESITO (N= 233.836) AL 3/06/2020

FONTE DATI MINISTERO DELLA SALUTE E PROTEZIONE CIVILE

- La Figura 4 e la Tabella 2 mostrano l'incidenza e la distribuzione dei casi segnalati per Regione/PA. I casi si concentrano soprattutto nel nord Italia, in particolare in Lombardia, Piemonte, Emilia-Romagna e Veneto. Molti casi sono riportati anche da Toscana, Liguria, Lazio, Marche, P.A. Trento, Campania, Puglia, Abruzzo, Friuli Venezia Giulia e Sicilia e in ognuna di queste regioni il numero totale di casi di COVID-19 segnalati al sistema di sorveglianza supera i 3.000. Altre 5 Regioni/PPAA hanno riportato ognuna oltre 1.000 casi di infezione. Le rimanenti 2 regioni (Molise e Basilicata) hanno riportato ognuna un numero di casi inferiore a 1.000.

- La Figura 4 mostra i dati di incidenza (per 100.000 abitanti) dei casi di COVID-19 diagnosticati dai laboratori di riferimento regionale (n=234.119) e il numero di casi diagnosticati negli ultimi 7 giorni (n=1.734), per Regione/P.A. di diagnosi. Si sottolinea che in alcune Regioni/PPAA che riportano un numero meno consistente di casi rispetto ad altre, l'incidenza cumulativa (numero di casi totali segnalati/popolazione residente) è tuttavia particolarmente elevata (PA Trento, e Valle d'Aosta), con valori simili a quelli riportati dalle Regioni Lombardia, Piemonte ed Emilia-Romagna.

- La Figura 5 confronta i dati di incidenza cumulativa per provincia di domicilio/residenza, raccolti dall'ISS con quelli raccolti dal Ministero della Salute/Protezione Civile (dati aggregati). Si può osservare che, sebbene l'incidenza stimata con i dati raccolti dal Ministero della Salute/Protezione civile sia più elevata perché meno soggetta a ritardo di notifica, le due mappe mostrano quadri simili relativamente alle aree di diffusione dell'infezione.

- La Tabella 3 riporta la distribuzione per fascia di età e sesso dei casi con un'età <18 anni. Complessivamente i casi diagnosticati in questa fascia di età sono l'1,9% del totale. Tra essi il 13,1% ha un'età inferiore ai 2 anni; il 69,4% ha una età >6 anni. La Tabella 4 riporta, per i casi per cui l'informazione è disponibile (3.529/4.564=77,3%), il dato sul luogo di trattamento. Complessivamente sono ospedalizzati circa il 2,2% dei casi <18 anni. La percentuale dei casi ospedalizzati è maggiore tra i casi con età <2 anni.

FIGURA 4 – INCIDENZA (PER 100.000 ABITANTI) DEI CASI DI COVID-19 DIAGNOSTICATI DAI LABORATORI DI RIFERIMENTO REGIONALE (N=230.811) E NUMERO DI CASI DIAGNOSTICATI NEGLI ULTIMI 7 GIORNI (N=1.734), PER REGIONE/PA DI DIAGNOSI

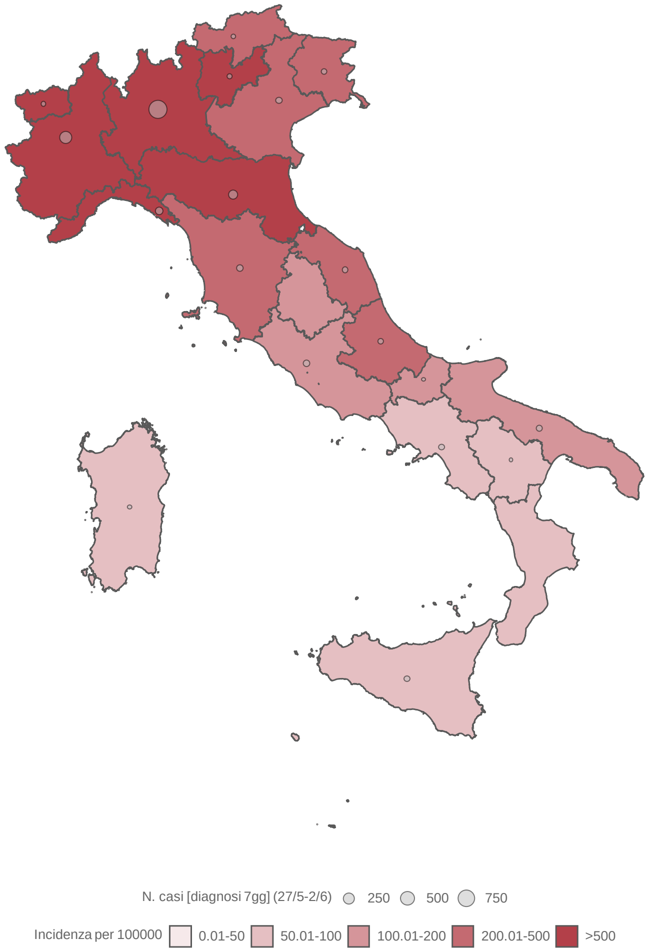

TABELLA 2- DISTRIBUZIONE DEI CASI DIAGNOSTICATI DAI LABORATORI DI RIFERIMENTO REGIONALE, PER REGIONE/PA DI DIAGNOSI (N=234.119)

Regione/PPAA	Casi	% su totale	Incidenza cumulativa per 100.000
Lombardia	89.205	38,1	886,68
Piemonte	31.140	13,3	714,81
Emilia-Romagna	27.828	11,9	624,02
Veneto	19.168	8,2	390,72
Toscana	9.907	4,2	265,63
Liguria	9.746	4,2	628,51
Lazio	7.779	3,3	132,32
Marche	6.720	2,9	440,58
PA di Trento	5.432	2,3	1.003,88
Campania	4.573	2,0	78,82
Puglia	4.499	1,9	111,66
Friuli Venezia Giulia	3.273	1,4	269,33
Abruzzo	3.255	1,4	248,17
Sicilia	3.071	1,3	61,42
PA di Bolzano	2.593	1,1	488,16
Umbria	1.431	0,6	162,24
Sardegna	1.349	0,6	82,28
Valle d'Aosta	1.193	0,5	949,34
Calabria	1.157	0,5	59,42
Molise	436	0,2	142,66
Basilicata	364	0,2	64,67

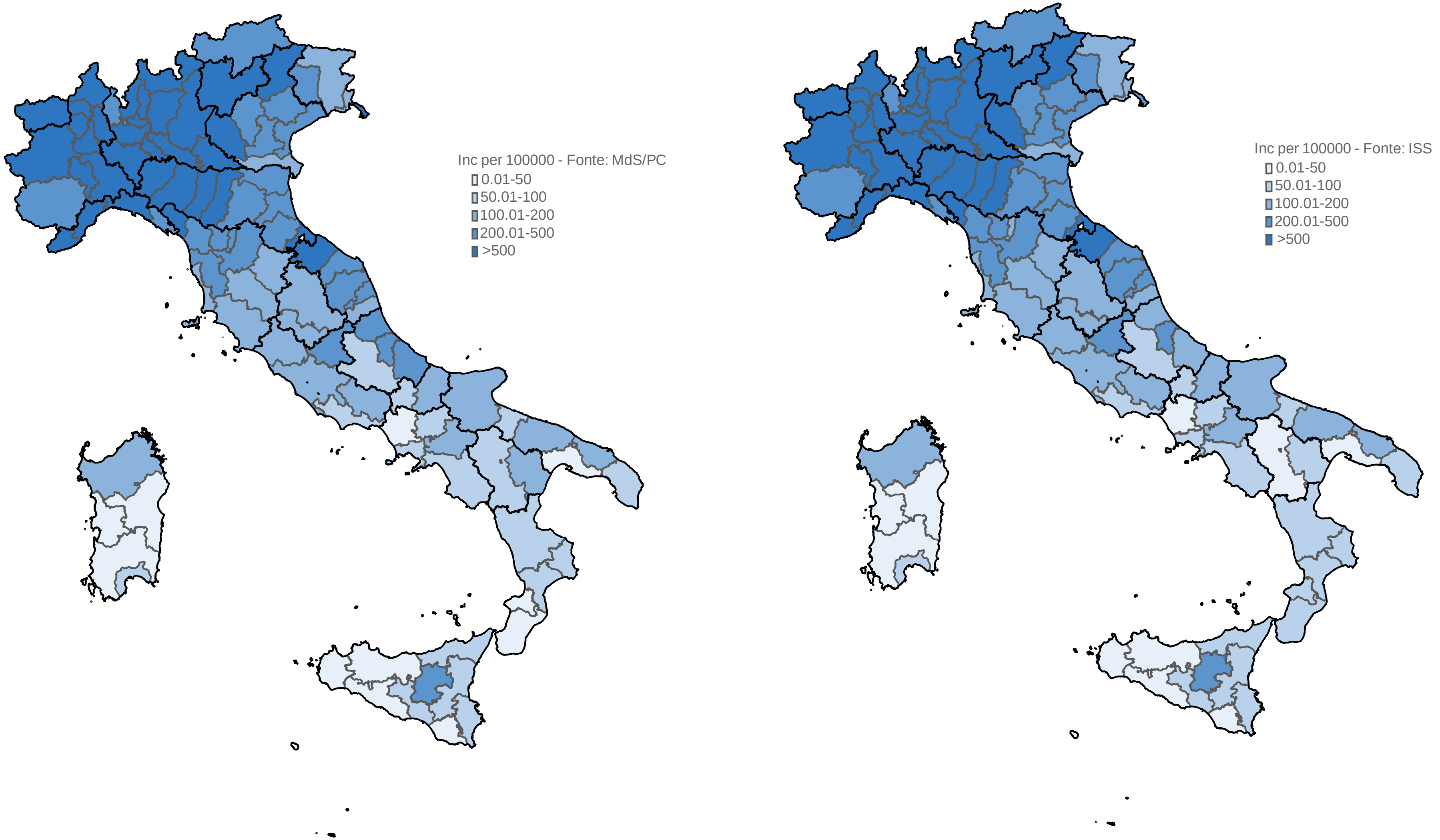

FIGURA 5 – INCIDENZA CUMULATIVA (PER 100.000 ABITANTI) DI COVID-19 PER PROVINCIA; CONFRONTO FONTE DATI ISTITUTO SUPERIORE DI SANITÀ (ISS) E MINISTERO DELLA SALUTE (MDS) E PROTEZIONE CIVILE

TABELLA 3 - DISTRIBUZIONE DEI CASI CON ETÀ <18 ANNI, PER CLASSE DI ETÀ (N=4.564)

Classe di età (anni)	N. casi	%	Femmine	Maschi	Non noto	% Femmine	% Maschi
0-1	596	13,1	272	323	1	45,7	54,3
2-6	801	17,5	375	426	NA	46,8	53,2
7-17	3.167	69,4	1.571	1.596	NA	49,6	50,4
**<18 anni**	4.564		2.218	2.345	1	48,6	51,4

TABELLA 4 - DISTRIBUZIONE DEI CASI CON ETÀ <18 ANNI PER CLASSE DI ETÀ (N=4.564) E LUOGO DI TRATTAMENTO

Classe di età (anni)	N. casi a domicilio	N. casi ospedalizzati	% ospedalizzati per classe di età	% ospedalizzati (su totale casi ospedalizzati <18 anni)
0-1	440	31	5,2	30,7
2-6	597	17	2,1	16,8
7-17	2.381	53	1,7	52,5
**<18 anni**	3.418	101	2,2	

Luogo di esposizione (analisi ristretta a casi diagnosticati dal 1 maggio)

- Un'analisi dell'informazione relativa al luogo di esposizione, disponibile per 3.349/22.631 casi, diagnosticati dal 1 maggio al 3 giugno 2020, ha evidenziato che 1.525 casi (45,5%) hanno contratto la malattia in una residenza sanitaria assistenziale o una comunità per disabili, 810 (24,2%) casi si sono contagiati in ambito familiare, mentre 218 casi (6,5%) si sono contagiati in ospedale o in ambulatorio. In Tabella 5 è riportato il luogo di esposizione per tutti i casi per cui l'informazione è disponibile. I dati, benché disponibili per un numero limitato di casi (14,8% del totale) sono in linea con quanto atteso a seguito delle misure di distanziamento sociale messe in atto a partire dal 9 marzo 2020. Una raccolta sistematica dell'informazione sul luogo di esposizione permetterebbe una valutazione più accurata dei contesti in cui sta avvenendo la trasmissione della malattia in questa fase della pandemia.

TABELLA 5 - DISTRIBUZIONE DEI LUOGHI DI ESPOSIZIONE DEI CASI DIAGNOSTICATI DAL 1/05/2020 (DATO DISPONIBILE PER 3.349/22.631 CASI).

Luogo di esposizione	Casi	
	N	%
RSA/Casa di Riposo/Comunità disabili	1.525	45,5
Ambito familiare	810	24,2
Ospedale/Ambulatorio	218	6,5
Lavoro*	97	2,9
Nave/Crociera	9	0,3
Comunità religiosa	8	0,2
Altro	682	20,4
**Totale**	**3.349**	

* Per Lavoro si intende qualunque luogo di lavoro diverso dagli altri ambiti riportati

Fattori di rischio

- Ad eccezione dei primi tre casi di COVID-19 segnalati, provenienti dalla Cina, nessun'altro caso notificato in Italia ha riportato una storia di viaggio in paesi con trasmissione sostenuta da SARS-CoV-2 durante il periodo di incubazione di 14 gg.

- Sono stati diagnosticati 28.276 casi tra operatori sanitari (età mediana 48 anni, 29,8% di sesso maschile), pari al 12% dei casi totali segnalati. La tabella 6 riporta la distribuzione dei casi segnalati per classe di età e la letalità osservata in questa popolazione.

- I dati indicano che la letalità tra gli operatori sanitari è inferiore rispetto alla letalità totale (vedi Tabella 1), verosimilmente perché gli operatori sanitari asintomatici e pauci-sintomatici sono stati maggiormente testati rispetto alla popolazione generale. Tuttavia, va anche sottolineato che l'informazione sull'esito della malattia non è nota per un numero elevato di casi.

- La Figura 6 riporta la percentuale di operatori sanitari tra i casi positivi segnalati in totale in Italia, per periodo di diagnosi (ogni 4 giorni). La curva mostra un andamento in lieve calo negli ultimi periodi di osservazione.

TABELLA 6. DISTRIBUZIONE DEI CASI, DECEDUTI E LETALITÀ IN OPERATORI SANITARI

Classe d'età (anni)	Casi		Deceduti		Letalità (%)
	N	%	N	%	
18-29	3.093	11,0	0	0,0	0%
30-39	4.947	17,6	1	1,2	0%
40-49	7.892	28,0	4	4,7	0,10%
50-59	9.202	32,7	20	23,5	0,20%
60-69	2.876	10,2	45	52,9	1,60%
70-79	160	0,6	15	17,6	9,40%
**Totale**	**28.170**		**85**		**0,30%**

NOTA: LA TABELLA NON INCLUDE I CASI CON ETÀ NON NOTA

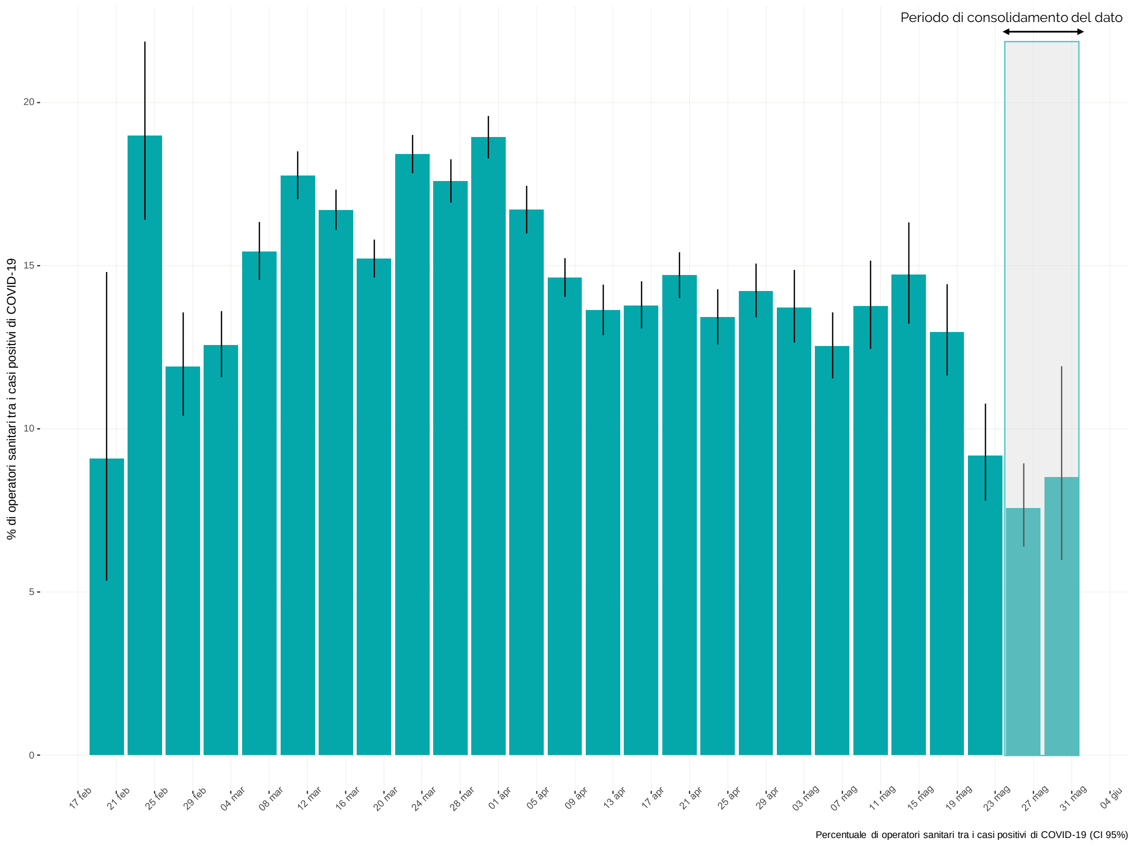

FIGURA 6 – PERCENTUALE DI OPERATORI SANITARI RIPORTATI SUL TOTALE DEI CASI PER PERIODO DI DIAGNOSI

NOTA: OGNI BARRA FA RIFERIMENTO ALL'INTERVALLO DI TEMPO TRA LA DATA INDICATA SOTTO LA BARRA E QUELLA SUCCESSIVA (ESEMPIO: 19 FEB SI RIFERISCE AL PERIODO DAL 19-22 FEB, 23 FEB SI RIFERISCE AL PERIODO DAL 23-26 FEB, ETC.)

Durante il periodo 18-31 maggio 2020, sono stati diagnosticati e segnalati complessivamente 6.350 casi, di cui 81 deceduti. Quest'ultima cifra non include le persone decedute nel periodo ma con una diagnosi avvenuta prima dell'18 maggio.

- L'età mediana dei casi è di 54 anni (range 0-100); 584 (9,1%) casi si sono verificati in operatori sanitari.

- La maggior parte dei casi è stata notificata dalla regione Lombardia seguita da Piemonte e Emilia-Romagna (Figura 7). Tuttavia, tutte le Regioni/PPAA hanno segnalato almeno un nuovo caso confermato di infezione da SARS-CoV-2 nella settimana 25-31 maggio 2020.

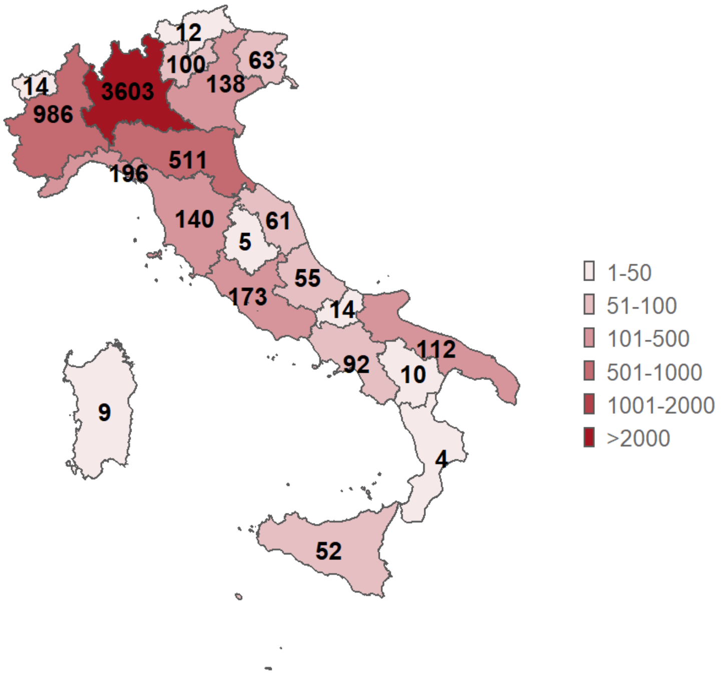

FIGURA 7 – CASI DI COVID-19 DIAGNOSTICATI PER REGIONE/PA DI NOTIFICA, ITALIA, 18-31/5/ 2020

In Figura 8 è riportata la curva epidemica per 6.350 casi diagnosticati tra il 18 e il 31 maggio e per 4.094 di questi è nota la data di inizio sintomi. La Figura evidenzia che per una piccola quota dei casi per cui la data d'inizio dei sintomi è nota, questa si può far risalire a un periodo molto lungo. Per i tempi tra esposizione al patogeno e lo sviluppo di sintomi e tra questi e la diagnosi e successiva notifica, verosimilmente molti dei casi notificati in queste settimane hanno contratto l'infezione 2-3 settimane prima, ovvero durante la prima fase di riapertura (tra il 4 e il 18 maggio 2020).

- Il lungo intervallo tra inizio sintomi e diagnosi per una parte dei casi notificati, verosimilmente riflette il recupero di campioni rimasti in lavorazione in alcuni laboratori e/o l'intensificarsi delle attività di screening con identificazione di soggetti positivi non più sintomatici con inizio sintomi riferito settimane o mesi addietro.

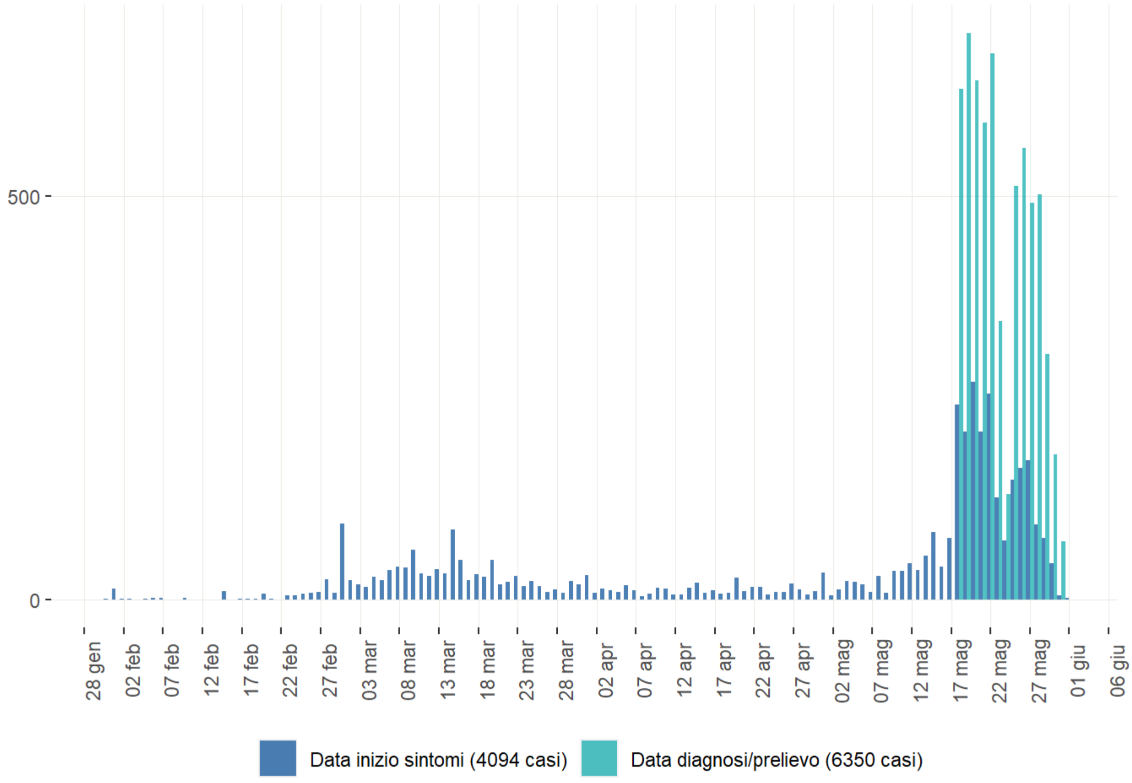

FIGURA 8 – CURVE EPIDEMICA PER DATA DI DIAGNOSI/PRELIEVO (VERDE) E DATA INIZIO SINTOMI (BLU) DEI CASI DI COVID-19 SEGNALATI AL SISTEMA DI SORVEGLIANZA INTEGRATA NAZIONALE, ITALIA, 18-31/5/2020

- Nel 56,3% dei casi le persone segnalate al sistema di sorveglianza in questo periodo hanno un'età superiore a 50 anni e nel 59,4% dei casi sono di sesso femminile (Figura 9 e Figura 10).

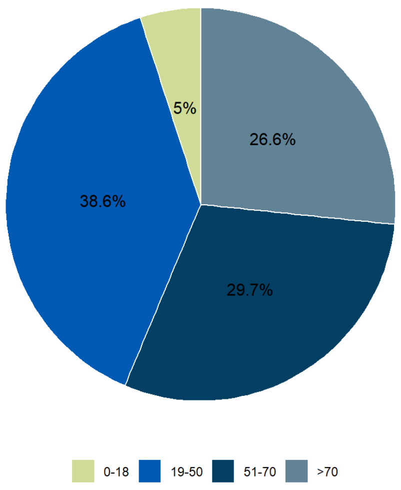

FIGURA 9 - DISTRIBUZIONE PER ETÀ DEI CASI CONFERMATI DI COVID-19, ITALIA, 18-31/5/ 2020

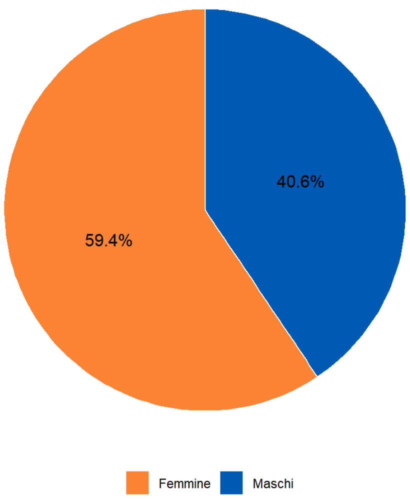

FIGURA 10 - DISTRIBUZIONE PER SESSO DEI CASI CONFERMATI DI COVID-19, ITALIA, 18-31/5/ 2020

- L'attuale andamento dell'epidemia richiama ad un atteggiamento di massima prudenza in questa fase di transizione in cui un rilassamento delle misure di distanziamento fisico potrebbe portare ad un nuovo incremento della trasmissione interumana del virus. È fondamentale mantenere comportamenti individuali corretti per limitare il rischio di un aumento del numero di casi e decessi nel breve termine.

Stime Rt - Calcolo del numero di riproduzione e interpretazione

- Il numero di riproduzione netto Rt indica il numero medio di infezioni secondarie generate da una persona infetta a una certa data, ed è una grandezza fondamentale per capire l'andamento dell'epidemia. Se Rt ha un valore inferiore alla soglia critica di 1, il numero di nuove infezioni tenderà a decrescere tanto più velocemente quanto più è lontano dall'unità. Per contro, quanto più Rt supera 1, tanto più rapidamente aumenterà il numero dei contagi. Un valore di Rt sopra la soglia, anche in presenza di un numero basso di casi, è un campanello di allarme sull'andamento epidemico. Il numero di riproduzione netto in un certo contesto geografico può essere stimato a partire dalla conoscenza della data di inizio sintomi dei casi, una volta nota la

distribuzione dell'intervallo seriale (ovvero la distanza temporale fra la comparsa dei sintomi in una persona infettata e nei casi da essa generati) [1,2,3].

- Viene riportata in questo bollettino la stima del numero di riproduzione netto Rt medio in un periodo di 14 giorni basato sulla data di inizio sintomi ($Rt^{medio14gg}$).

- **Nota bene**: Poiché la diagnosi di infezione da coronavirus SARS-CoV-2 può avvenire anche due o tre settimane dopo l'infezione a causa del tempo di incubazione della malattia (fino a 14 giorni) e dei tempi intercorsi tra l'inizio dei sintomi, la ricerca di assistenza medica e il completamento dei test di laboratorio, il valore di Rt può essere stimato correttamente solo con un ritardo di 9 giorni.

- La Figura 11 riporta la stima di $Rt^{medio14gg}$ basato su l'inizio dei sintomi per Regione/PA. **Si sottolinea che quando il numero di casi è molto piccolo, alcune Regioni/PPAA possono presentare temporaneamente stime di con valore medio Rt>1 a causa di piccoli focolai locali che incidono sul totale dei casi, senza che questo rappresenti necessariamente un elemento preoccupante.** La stima di $Rt^{medio14gg}$ é stata calcolata alla data del 3 giugno ed è riferibile alle due settimane che vanno dal 12 al 25 maggio 2020.

---

1 Guzzetta G, Riccardo F, Marziano V, Poletti P, Trentini F, Bella A, Andrianou X, et al. The impact of a nation-wide lockdown on COVID-19 transmissibility in Italy. Arxiv preprint. https://arxiv.org/ftp/arxiv/papers/2004/2004.12338.pdf

2 Cori A, Ferguson NM, Fraser C, Cauchemez S. A new framework and software to estimate time-varying reproduction numbers during epidemics. American journal of epidemiology. 2013;178(9):1505-12.

3 Riccardo F, Ajelli M, Andrianou X, Bella A, et al. Epidemiological characteristics of COVID-19 cases in Italy and estimates of the reproductive numbers one month into the epidemic. Medrxiv preprint https://doi.org/10.1101/2020.04.08.20056861

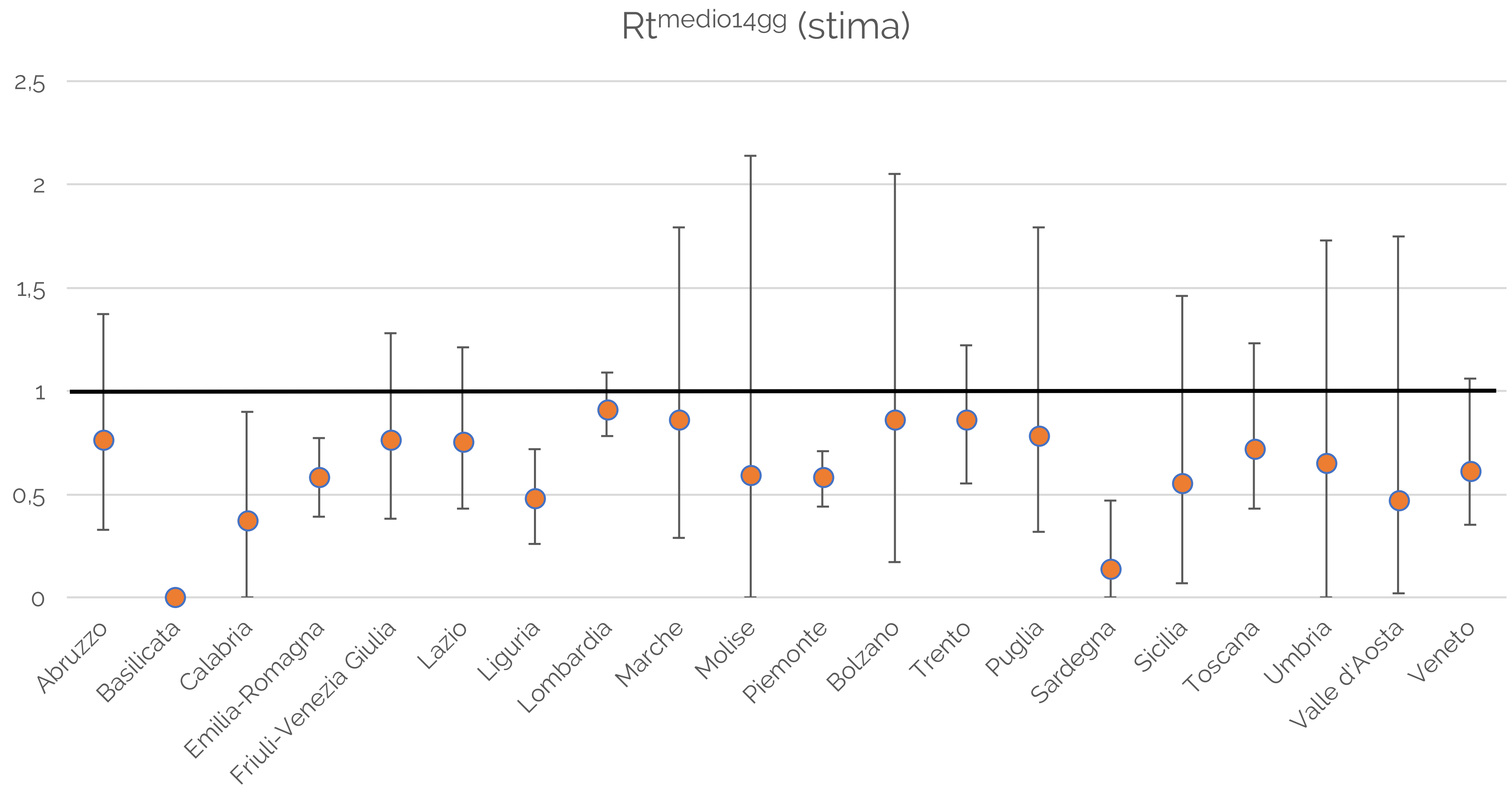

FIGURA 11. STIMA RIEPILOGATIVA DELL'RT^{MEDIO14GG} PER REGIONE BASATO SULLA DATA DI INIZIO SINTOMI DAL 12/5/2020 AL 25/5/2020, CALCOLATO AL 3/6/2020

2020

Epidemia COVID-19

Aggiornamento nazionale
29 dicembre 2020 – ore 12:00

DATA PUBBLICAZIONE: 31 DICEMBRE 2020

Prodotto dall'Istituto Superiore di Sanità (ISS), Roma

A cura di: Flavia Riccardo, Xanthi Andrianou, Antonino Bella, Martina Del Manso, Alberto Mateo Urdiales, Massimo Fabiani, Stefania Bellino, Stefano Boros, Fortunato (Paolo) D'Ancona, Maria Cristina Rota, Antonietta Filia, Ornella Punzo, Matteo Spuri, Maria Fenicia Vescio, Daniele Petrone, Corrado Di Benedetto, Marco Tallon, Alessandra Ciervo, Paola Stefanelli, Patrizio Pezzotti per ISS;
Giorgio Guzzetta, Valentina Marziano, Piero Poletti, Filippo Trentini, Marco Ajelli, Stefano Merler per Fondazione Bruno Kessler;
e di: Antonia Petrucci (Abruzzo); Michele La Bianca (Basilicata); Anna Domenica Mignuoli (Calabria); Pietro Buono (Campania); Erika Massimiliani (Emilia-Romagna); Tolinda Gallo (Friuli Venezia Giulia); Francesco Vairo (Lazio); Camilla Sticchi (Liguria); Danilo Cereda (Lombardia); Lucia Di Furia (Marche); Francesco Sforza (Molise); Annamaria Bassot (P.A. Bolzano); Pier Paolo Benetollo (P.A. Trento); Chiara Pasqualini (Piemonte); Lucia Bisceglia (Puglia); Maria Antonietta Palmas (Sardegna); Salvatore Scondotto (Sicilia); Emanuela Balocchini (Toscana); Anna Tosti (Umbria); Mauro Ruffier (Valle D'Aosta); Filippo Da Re (Veneto).

Citare il documento come segue: Task force COVID-19 del Dipartimento Malattie Infettive e Servizio di Informatica, Istituto Superiore di Sanità. Epidemia COVID-19,
Aggiornamento nazionale: 29 dicembre 2020

EPIDEMIA COVID-19

Aggiornamento nazionale

29 dicembre 2020 – *ore 12:00*

Nota di lettura: *Questo bollettino è prodotto dall'Istituto Superiore di Sanità (ISS) e riporta i dati della sorveglianza integrata dei casi di infezione da virus SARS-CoV-2 riportati sul territorio nazionale e coordinata dall'ISS ai sensi dell'Ordinanza n. 640 del 27 febbraio 2020. I dati vengono raccolti attraverso una piattaforma web dedicata e riguardano tutti i casi di infezione da virus SARS-CoV-2 confermati tramite positività al test molecolare standard. I dati vengono aggiornati giornalmente da ciascuna Regione/PA anche se alcune informazioni possono richiedere qualche giorno per il loro inserimento e/o aggiornamento. Per questo motivo, potrebbe non esserci una completa concordanza con quanto riportato attraverso il flusso informativo del Ministero della Salute che riporta dati aggregati.*
I dati raccolti sono in continua fase di consolidamento e, come prevedibile in una situazione emergenziale, alcune informazioni sono incomplete. In particolare, si segnala la possibilità di un ritardo di alcuni giorni tra il momento della esecuzione del tampone per la diagnosi e la segnalazione sulla piattaforma dedicata. Pertanto, il numero casi che si osserva nei giorni più recenti, deve essere interpretato come provvisorio.
Il bollettino descrive, con grafici, mappe e tabelle la diffusione, nel tempo e nello spazio, dell'epidemia di COVID-19 in Italia. Fornisce, inoltre, una descrizione delle caratteristiche delle persone affette.
La forte pressione sui dipartimenti di prevenzione, causata dall'intensificazione dell'epidemia e dal conseguente forte aumento dei casi, porta in alcune aree a un ritardo nella notifica e nell'aggiornamento delle informazioni sui casi individuali

Prodotto dall'Istituto Superiore di Sanità (ISS), Roma, 29 dicembre 2020

In evidenza

- L'epidemia in Italia si mantiene grave ancora a causa di un impatto elevato sui servizi assistenziali.
- Sebbene si osservi una diminuzione dell'incidenza a livello nazionale negli ultimi 14 gg (305,47 per 100.000 abitanti (14/12/2020 – 27/12/2020) vs 329,53 per 100.000 abitanti nel periodo 7/12/2020-20/12/2020, il valore è ancora lontano da livelli che permetterebbero il completo ripristino sull'intero territorio nazionale dell'identificazione dei casi e tracciamento dei loro contatti.
- Nel periodo 8 – 21 dicembre 2020, l'Rt medio calcolato sui casi sintomatici è stato pari a 0,93 (range 0,89 – 1.02) in lieve aumento da tre settimane e con un limite superiore dell'intervallo di credibilità al 95% che supera il valore 1. Continua quindi il segnale di controtendenza nell'indice di trasmissione segnalato nelle ultime tre settimane nell'intero Paese. Questo si realizza in un contesto europeo caratterizzato da un nuovo aumento nel numero di casi in molti paesi Europei e la circolazione di varianti virali con una potenziale maggiore capacità di trasmissione.
- Per dettagli sulle modalità di calcolo ed interpretazione dell'Rt riportato si rimanda all'approfondimento disponibile sul sito dell'Istituto Superiore di Sanità (https://www.iss.it/primo-piano/-/asset_publisher/o4oGR9qmvUz9/content/id/5477037)

Raccomandazioni

- Complessivamente, l'incidenza in Italia rimane ancora molto elevata e l'impatto dell'epidemia è ancora sostenuto nella maggior parte del Paese.
- Si conferma pertanto la necessità di mantenere la drastica riduzione delle interazioni fisiche tra le persone. È fondamentale che la popolazione eviti tutte le occasioni di contatto con persone al di fuori del proprio nucleo abitativo che non siano strettamente necessarie e di rimanere a casa il più possibile. Si ricorda che è obbligatorio adottare comportamenti individuali rigorosi e rispettare le misure igienico-sanitarie predisposte relative a distanziamento e uso corretto delle mascherine. Si ribadisce la necessità di rispettare le misure raccomandate dalle autorità sanitarie compresi i provvedimenti quarantenari dei contatti stretti dei casi accertati e di isolamento dei casi stessi.
- È importante continuare il rafforzamento dei servizi territoriali, attraverso un coinvolgimento straordinario di risorse professionali di supporto ed anche attraverso l'utilizzo di strumenti tecnologici come **la "app" Immuni**, nelle attività di ricerca dei contatti in modo da identificare precocemente tutte le catene di trasmissione e garantire una efficiente gestione, inclusa la quarantena dei contatti stretti e l'isolamento immediato dei casi secondari.
- **Si continua a richiamare l'importanza dell'uso appropriato degli strumenti diagnostici e di screening, nel contesto di una valutazione del rischio epidemiologico, e della corretta esecuzione delle procedure di isolamento e quarantena quando indicate.**
- **Si ribadisce la necessità di rispettare i provvedimenti quarantenari e le altre misure raccomandate dalle autorità sanitarie sia per le persone che rientrano da paesi per i quali è prevista la quarantena, e sia a seguito di richiesta dell'autorità sanitaria essendo stati individuati come contatti stretti di un caso**. Sebbene i servizi territoriali siano riusciti finora a contenere la trasmissione locale del virus, viene ripetutamente segnalato un carico di lavoro eccezionale che in molti casi compromette la tempestiva gestione dei contatti oltre che non assicurare le attività non-collegate a questa emergenza
- **Si raccomanda alla popolazione di continuare a prestare particolare attenzione al rischio di contrarre l'infezione in tutti i casi di mancato rispetto delle misure raccomandate. Si invita a rispettare tutte le norme comportamentali previste di**

prevenzione della trasmissione di SARS-CoV-2, in particolare nei confronti di fasce di popolazione più vulnerabili.

La situazione nazionale nelle ultime due settimane (14 - 27 dicembre 2020)

- Durante il periodo 14 - 27 dicembre 2020, sono stati diagnosticati e segnalati 184.032 nuovi casi, di cui 1.059 deceduti (questo numero non include le persone decedute nel periodo con una diagnosi antecedente al 14 dicembre). **Si sottolinea che a causa della forte pressione sui dipartimenti di prevenzione si sono registrati dei ritardi nella notifica e nell'aggiornamento tempestivo delle informazioni dei casi individuali, rendendo quindi il quadro più recente in parte sottostimato sia per le nuove diagnosi che per i decessi.**

- 7.376 (4%) casi si sono verificati in operatori sanitari. Anche questo numero è verosimilmente sottostimato in quanto questa informazione può richiedere tempi più lunghi per un suo consolidamento.

- La maggior parte dei casi sono stati notificati dalle regioni Veneto (N=47.538), Lombardia (N=25.388), Emilia-Romagna (N=18.442), Lazio (N=16.737), Puglia (N=12.661), Sicilia (N=10.468), Campania (N=10.402) e Piemonte (N=10.181) (**Figura 1**).

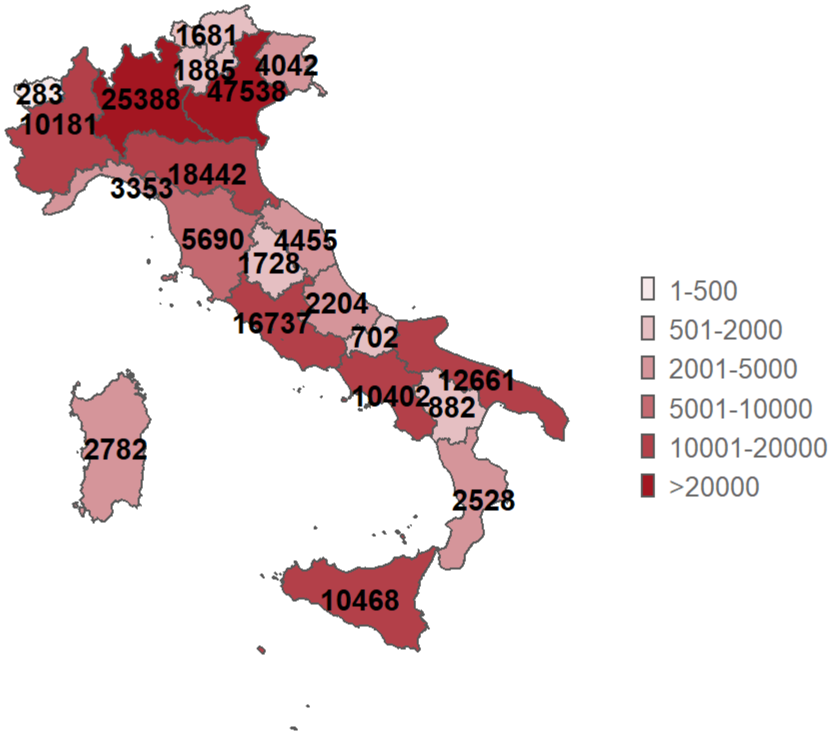

FIGURA 1 – CASI DI COVID-19 DIAGNOSTICATI IN ITALIA PER REGIONE/PA DI NOTIFICA. PERIODO: 14 - 27 DICEMBRE 2020

- In **Figura 2** è riportata la curva epidemica per data di diagnosi/prelievo dei 184.032 casi diagnosticati nel periodo 14 - 27 dicembre 2020 (in verde) e per data di inizio sintomi dei 81.699 casi per cui la data è nota e compresa negli ultimi 30 giorni (in blu).

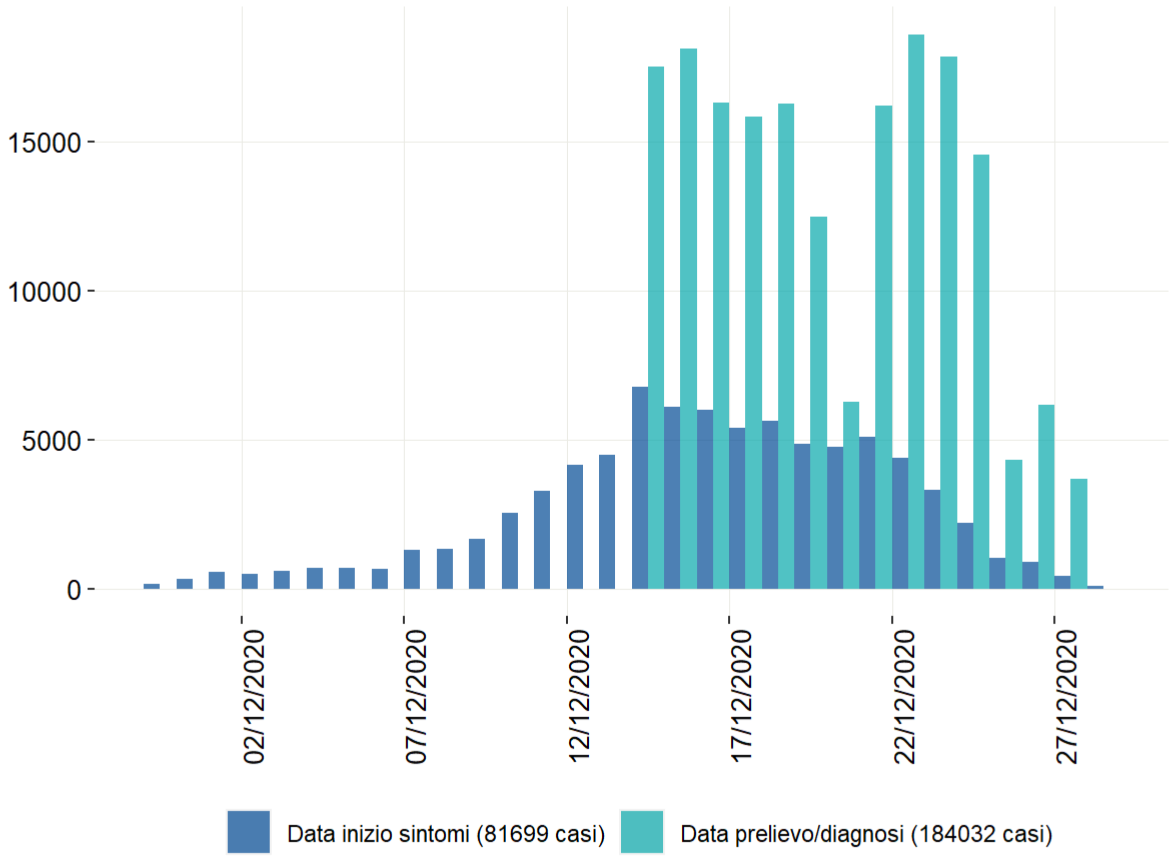

FIGURA 2 – CURVA EPIDEMICA PER DATA DI DIAGNOSI/PRELIEVO (VERDE) E DATA INIZIO SINTOMI (BLU) DEI CASI DI COVID-19 DIAGNOSTICATI IN ITALIA.
PERIODO: 14 - 27 DICEMBRE 2020

- Nel 45,6% dei casi le persone segnalate al sistema di sorveglianza nelle ultime due settimane hanno un'età superiore a 50 anni e l'11,8% ha meno di 19 anni (età mediana 48 anni (0-109 aa)); il 47,7% dei casi sono di sesso maschile (**Figura 3** e **Figura 4**).

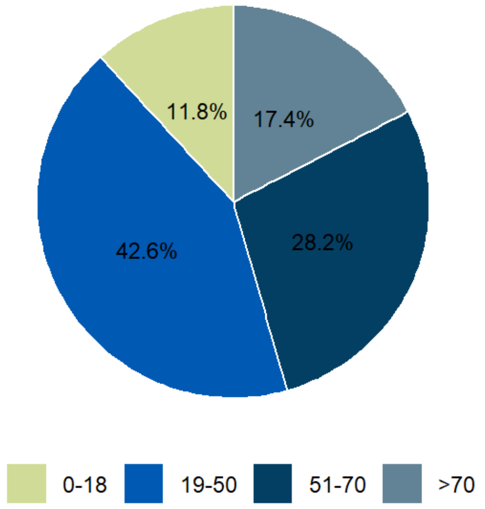

FIGURA 3 - DISTRIBUZIONE PER ETÀ DEI CASI DI COVID-19 DIAGNOSTICATI IN ITALIA
PERIODO: 14 - 27 DICEMBRE 2020

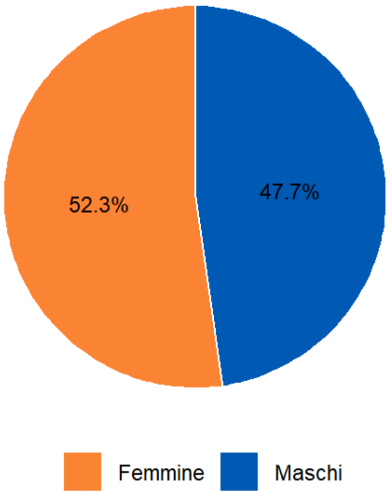

FIGURA 4 - DISTRIBUZIONE PER SESSO DEI CASI DI COVID-19 DIAGNOSTICATI IN ITALIA
PERIODO: 14 - 27 DICEMBRE 2020

- La **Figura 5** mostra la distribuzione dei nuovi casi di infezione da virus SARS-CoV-2 per comune di domicilio/residenza riportati al Sistema di Sorveglianza Integrato Nazionale COVID-19. Nella mappa sono riportati 178.787 casi rispetto ai 184.032 segnalati nel periodo 14 - 27 dicembre 2020 (3.952 casi sono stati esclusi poiché non è nota l'informazione sul domicilio/residenza e 1.293 casi hanno un domicilio/residenza che non si trova nella Regione/PA di diagnosi). I casi sono distribuiti in 6.123 comuni con un'importante diffusione su tutto il territorio nazionale.

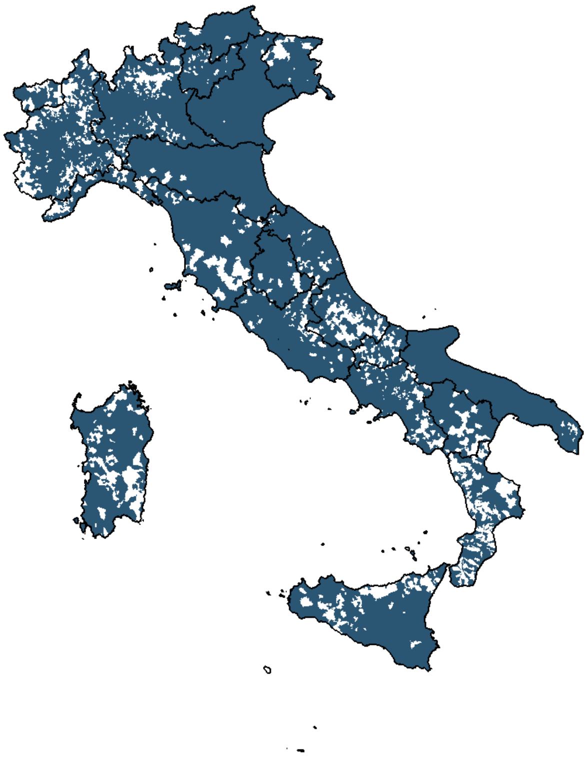

FIGURA 5 - CASI DI COVID-19 DIAGNOSTICATI IN ITALIA PER COMUNE DI DOMICILIO/RESIDENZA (COMUNI CON ALMENO UN CASO). PERIODO: 14 - 27 DICEMBRE 2020

- La **Tabella 1** e la **Tabella 2** riportano rispettivamente il motivo per cui i nuovi casi diagnosticati nel periodo di riferimento sono stati sottoposti ad accertamento diagnostico e l'origine dei casi di Covid-19 diagnosticati in Italia.

TABELLA 1 - DISTRIBUZIONE DEL MOTIVO PER CUI I CASI DI COVID-19 DIAGNOSTICATI IN ITALIA SONO STATI TESTATI – PERIODO: 14 - 27 DICEMBRE 2020

Motivo del test	Casi	
	N	%
Screening	52.942	28,8
Contact tracing	47.764	26,0
Paziente con sintomi	62.616	34,0
Non noto	20.710	11,2
**Totale**	**184.032**	

TABELLA 2 - DISTRIBUZIONE DELL'ORIGINE DEI CASI DI COVID-19 DIAGNOSTICATI IN ITALIA. PERIODO: 14 - 27 DICEMBRE 2020

Origine dei casi	Casi	
	N	%
Autoctoni	144.958	78,7
Importati dall'estero	301	0,2
Provenienti da regione diversa da quella di notifica	161	0,1
Non noto	38.612	21,0
**Totale**	**184.032**	

La situazione delle regioni nelle ultime due settimane (14 - 27 dicembre 2020)

- La **Tabella 3** riporta il numero dei casi totali dall'inizio dell'epidemia, l'incidenza cumulativa (per 100.000 abitanti), il numero di casi e l'incidenza nell'ultima settimana (21 - 27 dicembre) e negli ultimi 14 giorni (14 - 27 dicembre 2020) per Regione/PA e per l'Italia. La distribuzione dei casi nelle ultime due settimane non è uniforme nelle regioni. Il Veneto riporta la maggiore incidenza con 968,64 casi per 100.000 abitanti, mentre la Calabria che ha l'incidenza più bassa riporta un valore pari a 131,35 casi per 100.000 abitanti. Sei regioni (Emilia-Romagna, Friuli-Venezia Giulia, PA di Bolzano, PA di Trento, Puglia e Veneto) riportano un'incidenza pari o superiore al valore nazionale di incidenza che è 305,47 casi per 100.000 abitanti (**Figura 6, Tabella 3**).

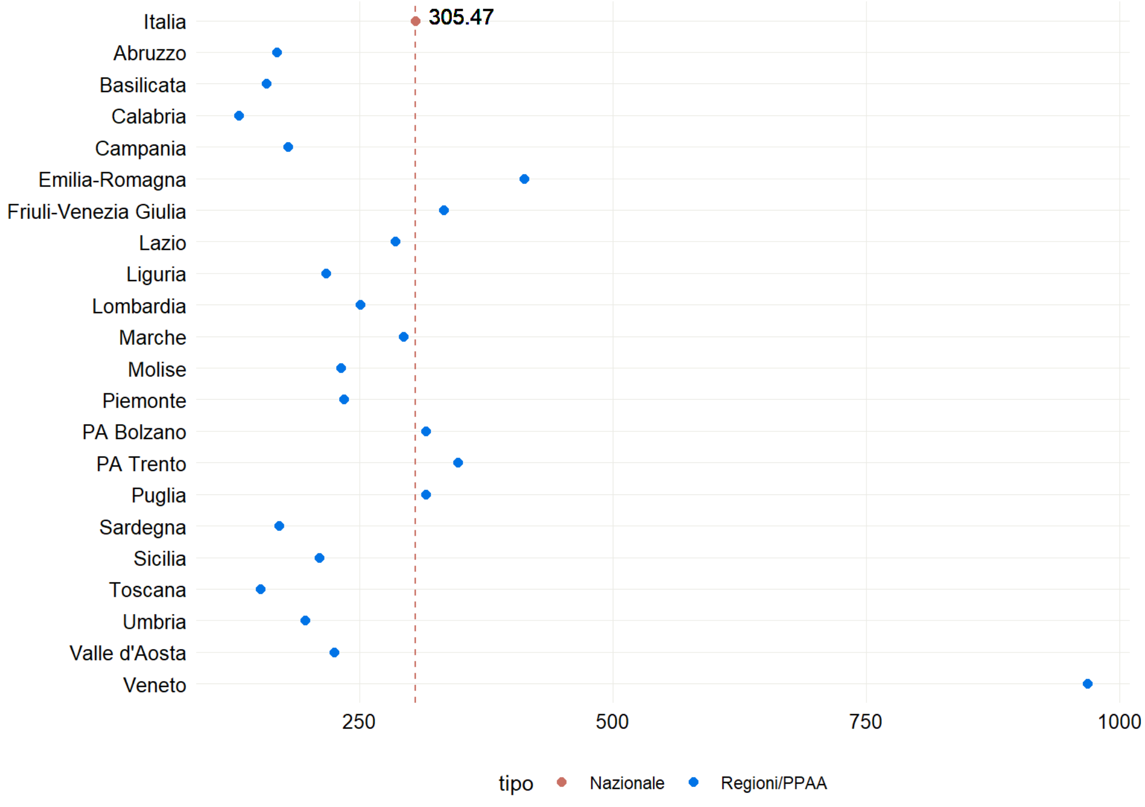

FIGURA 6 - NUMERO CASI DI COVID-19 (PER 100.000 AB) DIAGNOSTICATI IN ITALIA PER REGIONE/PA. PERIODO: 14 - 27 DICEMBRE 2020

TABELLA 3 - NUMERO ASSOLUTO E INCIDENZA (PER 100.000 AB) DEI CASI DI COVID-19 DIAGNOSTICATI IN ITALIA PER REGIONE/PA DALL'INIZIO DELL'EPIDEMIA (INCIDENZA CUMULATIVA) E NEI PERIODI 21 - 27/12 E 14 - 27/12.

REGIONE/PA	NUMERO DI CASI TOTALE	INCIDENZA CUMULATIVA (PER 100.000 AB)	N. CASI TRA IL 21 - 27/12	INCIDENZA 7GG (PER 100.000 AB)	N. CASI TRA IL 14 - 27/12	INCIDENZA 14GG (PER 100.000 AB)
Abruzzo	34.549	2.645,87	964	73,83	2.204	168,79
Basilicata	10.287	1.847,08	394	70,74	882	158,37
Calabria	23.637	1.228,09	1.290	67,02	2.528	131,35
Campania	183.734	3.175,57	4.417	76,34	10.402	179,78
Emilia-Romagna	167.077	3.740,15	8.254	184,77	18.442	412,84
Friuli-Venezia Giulia	39.931	3.296,39	1.899	156,77	4.042	333,68
Lazio	163.066	2.780,07	7.824	133,39	16.737	285,34
Liguria	59.295	3.842,52	1.419	91,96	3.353	217,29
Lombardia	472.230	4.673,71	10.680	105,70	25.388	251,27
Marche	38.603	2.542,35	1.940	127,77	4.455	293,40
Molise	6.395	2.115,69	260	86,02	702	232,25
Piemonte	195.313	4.498,87	4.215	97,09	10.181	234,51
PA Bolzano	28.315	5.321,57	709	133,25	1.681	315,93
PA Trento	21.147	3.896,35	646	119,03	1.885	347,31
Puglia	87.855	2.191,83	5.547	138,39	12.661	315,87
Sardegna	27.188	1.667,49	1.069	65,56	2.782	170,63
Sicilia	91.197	1.835,54	4.649	93,57	10.468	210,69
Toscana	118.409	3.180,70	2.444	65,65	5.690	152,84
Umbria	28.553	3.243,61	766	87,02	1.728	196,30
Valle d'Aosta	7.235	5.764,89	127	101,19	283	225,50
Veneto	245.918	5.010,86	21.802	444,24	47.538	968,64
**ITALIA**	**2.049.934**	**3.402,68**	**81.315**	**134,97**	**184.032**	**305,47**

Prodotto dall'Istituto Superiore di Sanità (ISS), Roma, 29 dicembre 2020

In **Figura 7** è riportato il confronto tra l'incidenza (per 100.000 abitanti) delle ultime due settimane **(14 - 27 dicembre 2020)** e quella osservata nelle due settimane precedenti **(30 novembre - 13 dicembre 2020)**. Il verso e il colore della freccia indicano aumenti (arancione) o diminuzione (blu).

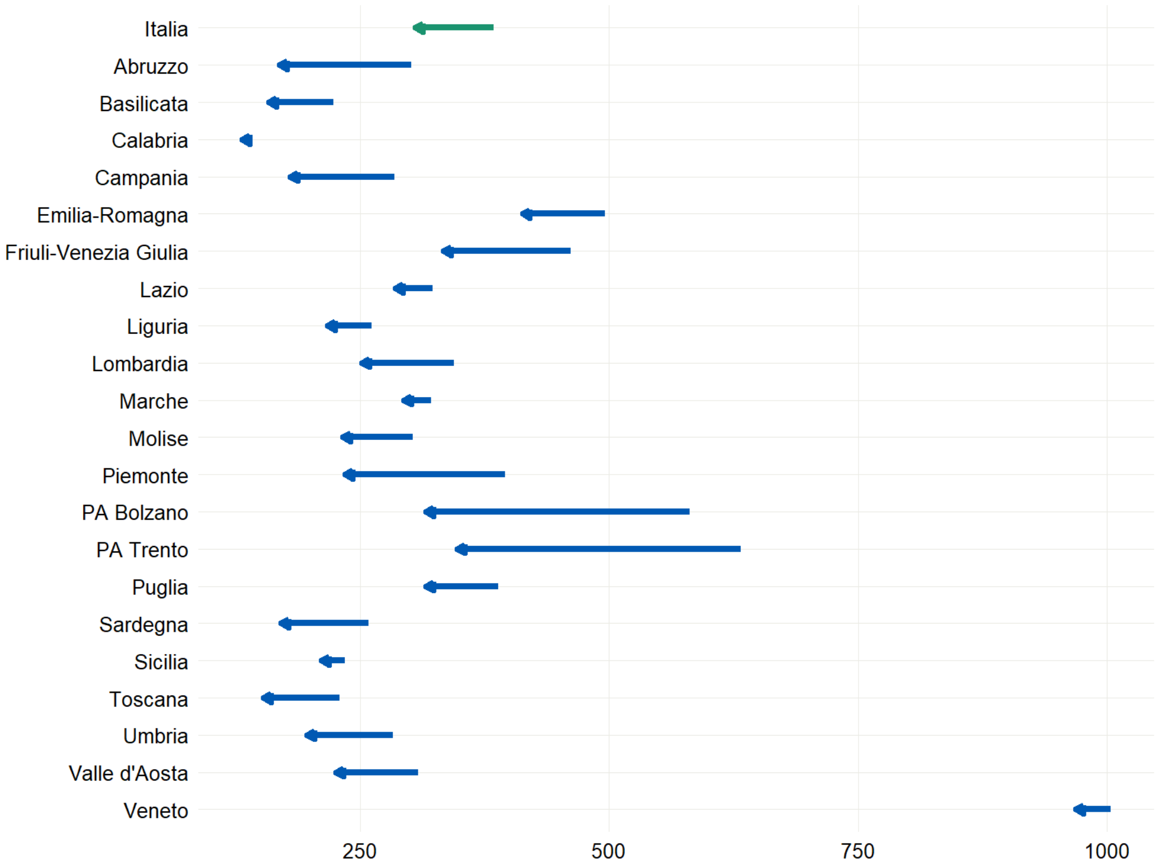

FIGURA 7 – CONFRONTO TRA IL NUMERO CASI DI COVID-19 (PER 100.000 AB) DIAGNOSTICATI IN ITALIA E PER REGIONE NEL PERIODO (14 - 27 DICEMBRE 2020) E (30 NOVEMBRE - 13 DICEMBRE 2020)

- In **Figura 8** viene riportata la stima del numero di riproduzione netto Rt medio in un periodo di 14 giorni basato sulla data di inizio sintomi (Rtmedio14gg). L'indice di trasmissione nazionale (Rtmedio14gg) calcolato al 29 dicembre 2020 sui casi sintomatici e riferito al periodo 8– 21 dicembre 2020, è pari a **0,93 (range 0,89 – 1.02)** in ulteriore aumento nelle ultime due settimane. Si riscontrano valori di RT puntuale inferiore a 1 anche nel suo intervallo di credibilità maggiore solo in 3 Regioni/PPAA, il che indica un aumento della trasmissibilità rispetto alla settimana precedente (quando erano 19 le regioni che avevano un indice di trasmissibilità puntuale inferiore a 1 anche nel suo intervallo di credibilità maggiore). **L'acquisizione dei dati epidemiologici sulle infezioni è affetta da una serie di ritardi, alcuni dei quali non comprimibili: in particolare, il tempo tra l'evento infettivo e lo sviluppo dei sintomi (tempo di incubazione), quello tra i sintomi e l'esecuzione del tampone, quello tra l'esecuzione del tampone e la conferma di positività, e quello tra la conferma di positività e l'inserimento nel sistema di sorveglianza integrata ISS. Il ritardo complessivo tra infezioni e loro rilevamento nel sistema di sorveglianza è valutato e aggiornato settimanalmente.**

Su queste valutazioni si basa la scelta della data più recente alla quale si può considerare sufficientemente stabile la stima di Rt. Per il presente bollettino, ad esempio, si considera il 21 dicembre come data ultima per valutare la stima di Rt dai casi sintomatici.

- La **Figura 9** riporta la stima del numero di riproduzione netto Rt medio nazionale a 14 giorni nel tempo in relazione alla curva epidemica.

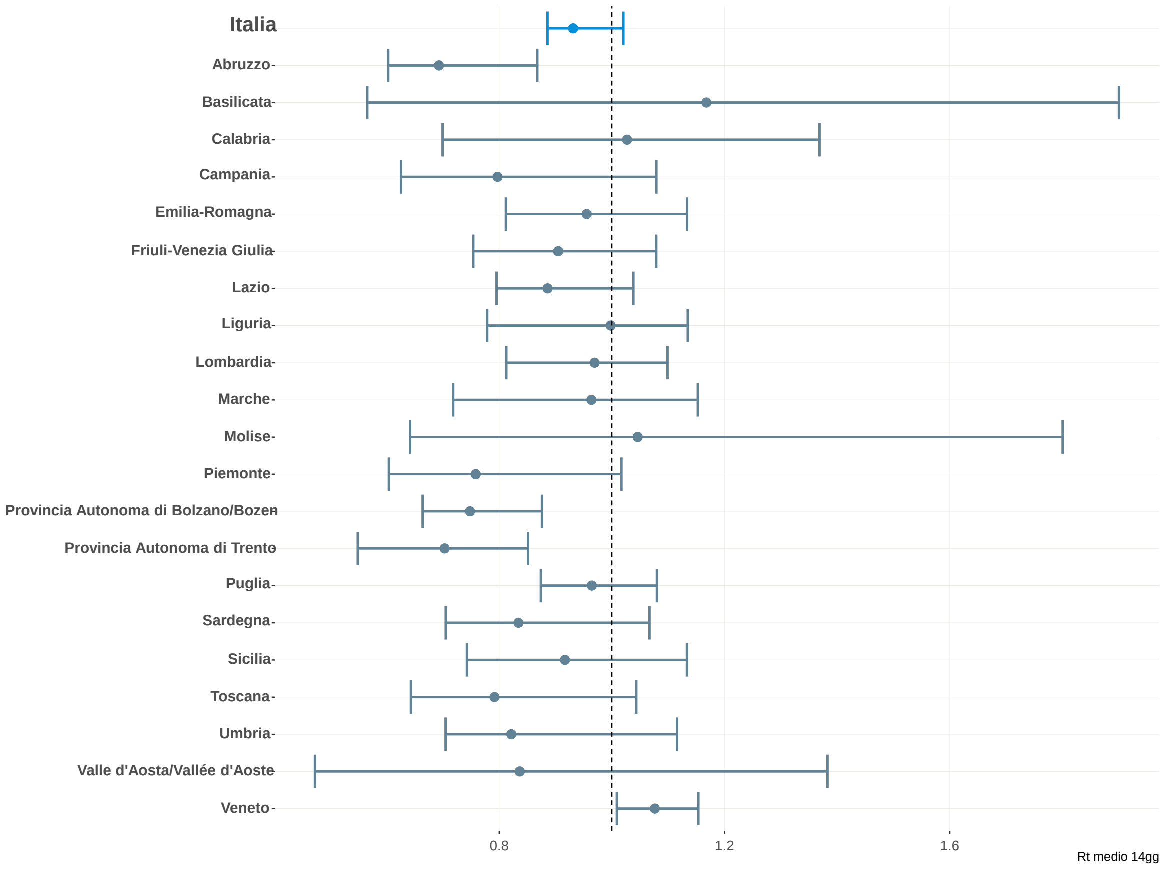

FIGURA 8 - STIMA RIEPILOGATIVA DELL'RTMEDIO14GG PER REGIONE BASATO SU INIZIO SINTOMI DALL' 8 AL 21 DICEMBRE, CALCOLATO IL 29/12/2020

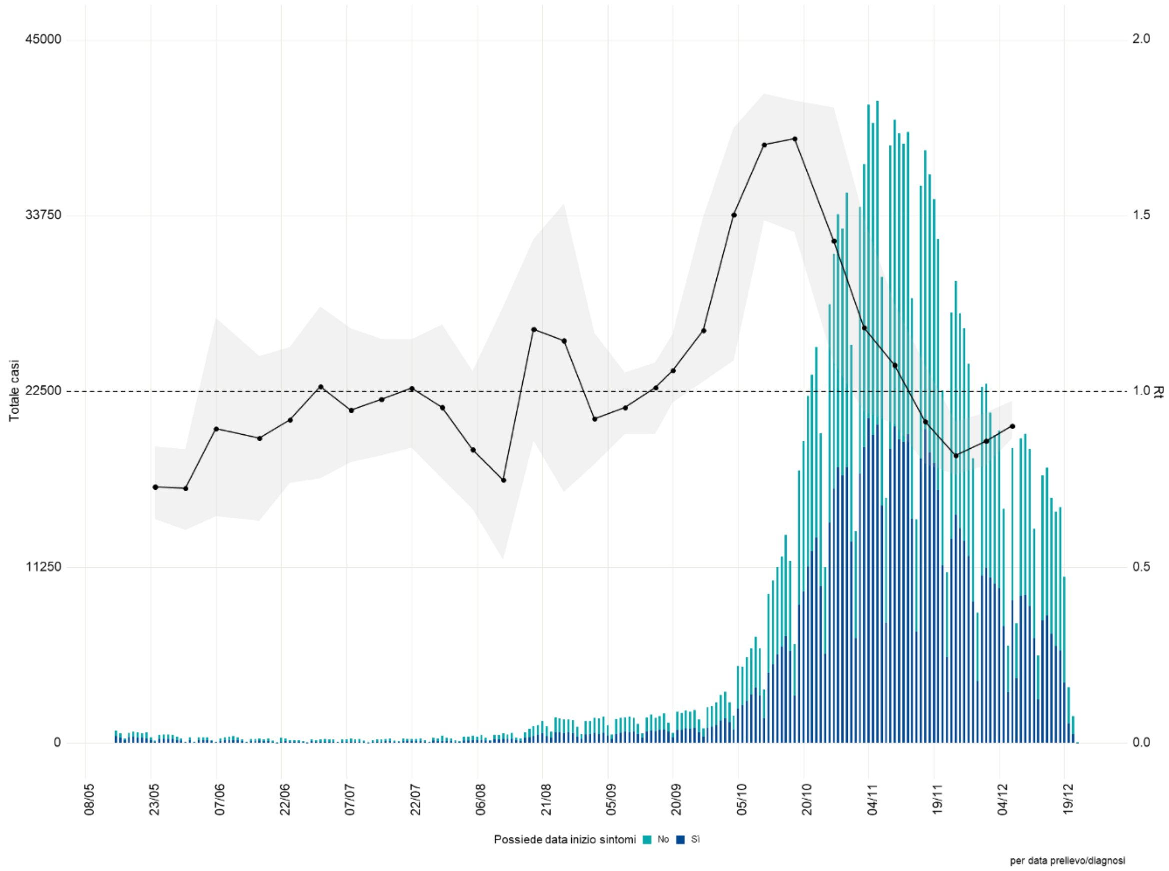

FIGURA 9 - STIME DELL'RTMEDIO14GG E NUMERO DI CASI PER DATA PRELIEVO/DIAGNOSI DISTINTI PER PRESENZA O ASSENZA DELLA DATA DI INIZIO SINTOMI.
Il punto corrispondente a ciascuna stima di Rt è collocato nel giorno centrale dell'intervallo di 14 gg a cui si riferisce

La situazione nazionale dall'inizio dell'epidemia (al 29 dicembre 2020)

- Dall'inizio dell'epidemia alle ore 12 del 29 dicembre 2020, sono stati riportati al sistema di sorveglianza 2.049.934 casi di COVID-19 diagnosticati in Italia dai laboratori di riferimento regionale come positivi per SARS-CoV–2 (86.911 casi in più rispetto al 22 dicembre 2020) e 70.799 decessi (3.259 decessi in più rispetto al 22 dicembre).

- La **Figura 10** mostra l'andamento del numero di casi di COVID-19 segnalati in Italia per data di prelievo/diagnosi (disponibile per 2.046.584/2.049.934 casi). La curva epidemica mostra che l'impatto della seconda ondata epidemica, in termini di numero complessivo di casi giornalieri notificati, è decisamente più elevato di quello della prima ondata, grazie all'aumentata capacità diagnostica. Dalla metà di novembre la curva mostra un andamento in lenta ma costante diminuzione, anche se si ricorda che il numero di casi riportati negli ultimi 7 giorni potrebbe essere sottostimato a causa di un ritardo nella notifica (box grigio).

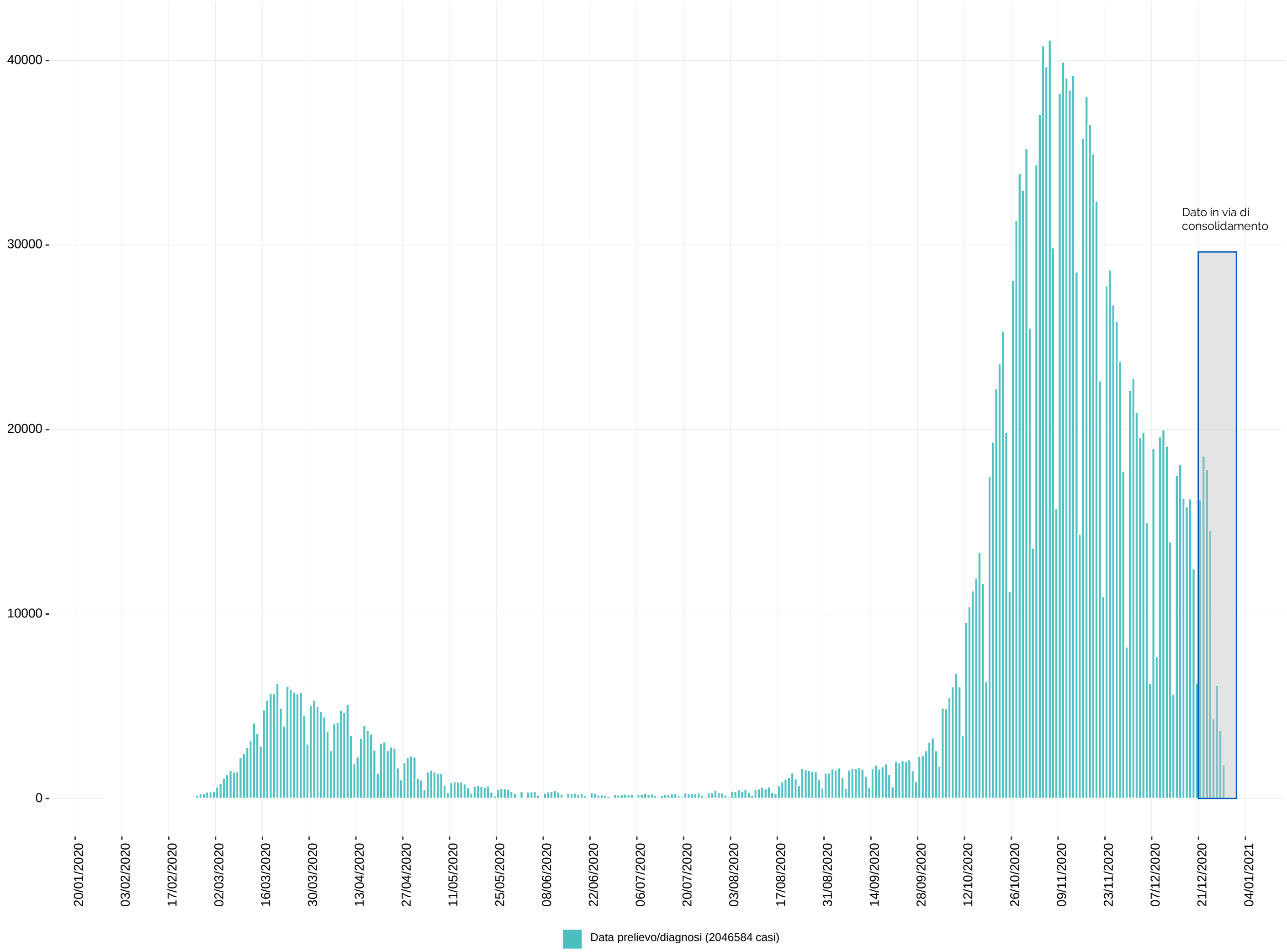

FIGURA 10 – CASI DI COVID-19 DIAGNOSTICATI IN ITALIA PER DATA PRELIEVO/DIAGNOSI (N=2.046.584).
Nota: i dati più recenti devono essere considerati provvisori (soprattutto i dati nel riquadro grigio)

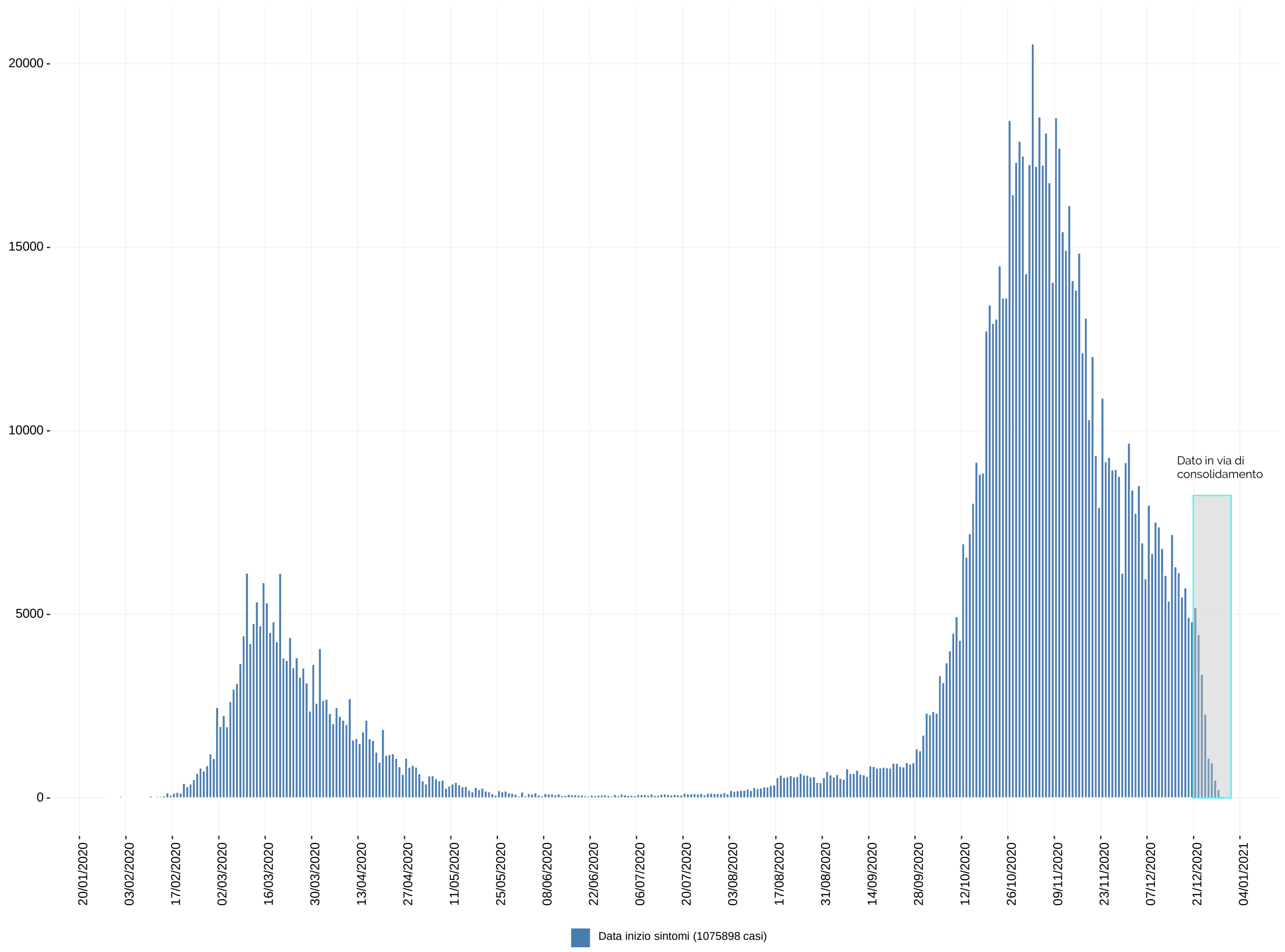

FIGURA 11 – CASI DI COVID-19 DIAGNOSTICATI IN ITALIA PER DATA INIZIO SINTOMI (N=1.075.898)

Nota: i dati più recenti devono essere considerati provvisori sia per il ritardo di notifica sia perché casi non ancora diagnosticati riporteranno in parte la data di inizio sintomi nei giorni del riquadro grigio.

- La **Figura 11** mostra la distribuzione dei casi per data inizio dei sintomi. La data di inizio sintomi è al momento disponibile per 1.075.898/2.049.934 casi segnalati. Lo scarto tra il numero di casi segnalati e quello di casi per i quali è disponibile la data di inizio dei sintomi è dovuta al fatto che una elevata percentuale dei casi diagnosticati è asintomatica e/o al consolidamento del dato ancora in corso.

- La **Tabella 4** riporta il tempo mediano trascorso tra la data di insorgenza dei primi sintomi e la data di diagnosi (dato disponibile per 1.074.904 casi) per intervalli di tempo con lo stesso tempo mediano. Dall'inizio dell'epidemia il tempo mediano che intercorre tra l'inizio dei sintomi e la diagnosi ha oscillato tra i 4 e i 5 giorni fino a metà giugno, da metà giugno a fine agosto si è ridotto a 2 giorni e da agosto in poi si registrano oscillazioni tra i 3 e i 4 giorni.

TABELLA 4 - DISTRIBUZIONE DEI CASI PER TEMPO MEDIANO INTERCORSO TRA DATA INIZIO DEI SINTOMI E PRELIEVO/DIAGNOSI (N= 1.074.904).

Periodo di prelievo/diagnosi		N. casi	Tempo mediano (gg)
Dal	al		
20/02/2020	20/03/2020	52.491	4
21/03/2020	09/04/2020	76.979	5
10/04/2020	19/04/2020	21.923	4
20/04/2020	29/05/2020	31.740	5
30/05/2020	08/06/2020	1.319	4
09/06/2020	18/06/2020	1.396	5
19/06/2020	27/08/2020	12.359	2
28/08/2020	26/09/2020	19.523	3
27/09/2020	26/10/2020	149.525	2
27/10/2020	05/11/2020	165.310	3
06/11/2020	25/11/2020	310.988	4
26/11/2020	15/12/2020	166.119	3
16/12/2020	25/12/2020	60.847	2
26/12/2020	29/12/2020	4.385	3

FIGURA 12 – INCIDENZA (PER 100.000 ABITANTI) E NUMERO DI CASI COVID-19 DIAGNOSTICATI IN ITALIA NELLA SETTIMANA 21 – 27 DICEMBRE 2020 (N=81.315), PER REGIONE/PA DI DIAGNOSI

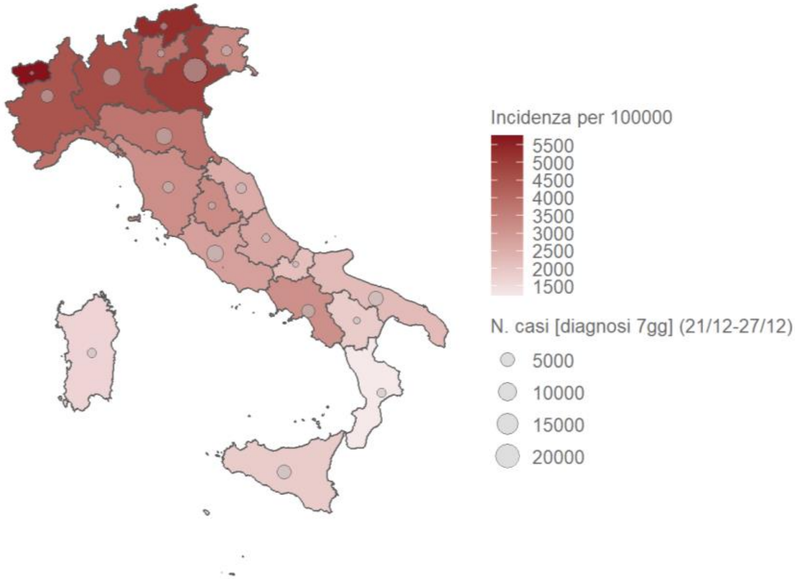

TABELLA 5 - DISTRIBUZIONE DEI CASI DI COVID-19 DIAGNOSTICATI IN ITALIA PER REGIONE/PA DI DIAGNOSI (N=2.049.934) DALL'INIZIO DELL'EPIDEMIA

Regione/PA	N. Casi	% sul totale	Incidenza cumulativa per 100.000
Valle d'Aosta	7.235	0.4%	5.764,89
PA Bolzano	28.315	1.4%	5.321,57
Veneto	245.918	12.0%	5.010,86
Lombardia	472.230	23.0%	4.673,71
Piemonte	195.313	9.5%	4.498,87
PA Trento	21.147	1.0%	3.896,35
Liguria	59.295	2.9%	3.842,52
Emilia-Romagna	167.077	8.2%	3.740,15
Friuli-Venezia Giulia	39.931	1.9%	3.296,39
Umbria	28.553	1.4%	3.243,61
Toscana	118.409	5.8%	3.180,70
Campania	183.734	9.0%	3.175,57
Lazio	163.066	8.0%	2.780,07
Abruzzo	34.549	1.7%	2.645,87
Marche	38.603	1.9%	2.542,35
Puglia	87.855	4.3%	2.191,83
Molise	6.395	0.3%	2.115,69
Basilicata	10.287	0.5%	1.847,08
Sicilia	91.197	4.4%	1.835,54
Sardegna	27.188	1.3%	1.667,49
Calabria	23.637	1.2%	1.228,09

- La **Figura 12** mostra l'incidenza (per 100.000 abitanti) dei casi confermati di COVID-19 e il numero di casi diagnosticati nella settimana dal 21 - 27 dicembre (n=81.315), per Regione/PA di diagnosi.

- La **Tabella 5** riporta in dettaglio il numero dei casi ed il tasso di incidenza cumulativa per 100.000 abitanti per Regione/PA. L'87% dei casi sono stati diagnosticati in 10 regioni: Lombardia, Veneto, Piemonte, Campania, Emilia-Romagna, Lazio, Toscana, Sicilia, Puglia e Liguria. Otto regioni (Friuli-Venezia Giulia, Marche, Abruzzo, PA Bolzano, Umbria, Sardegna, Calabria e PA Trento) hanno riportato tra 15.000 e 40.000 casi; tre regioni/PA (Basilicata, Valle d'Aosta e Molise) hanno riportato meno di 15.000 casi ciascuna. Si sottolinea che, a causa della numerosità della popolazione, la PA di Trento e la regione Valle d'Aosta pur riportando un numero meno consistente di casi presentano una incidenza cumulativa (numero di casi totali segnalati/popolazione residente) particolarmente elevata, con valori simili a quelli riportati dalla Lombardia.

- L'età mediana dei casi confermati di infezione da SARS-CoV-2 segnalati dall'inizio dell'epidemia è complessivamente pari a 48 anni (range 0-109 aa). La **Figura 13** mostra l'andamento dell'età mediana per settimana di diagnosi; si osserva, a partire dalla fine di aprile, un chiaro trend in diminuzione con l'età mediana che passa da oltre 60 anni nei primi due mesi dell'epidemia a circa 30 anni nella settimana centrale di agosto per poi risalire lentamente fino a 49 anni e poi riscendere a 48 anni nelle ultime tre settimane.

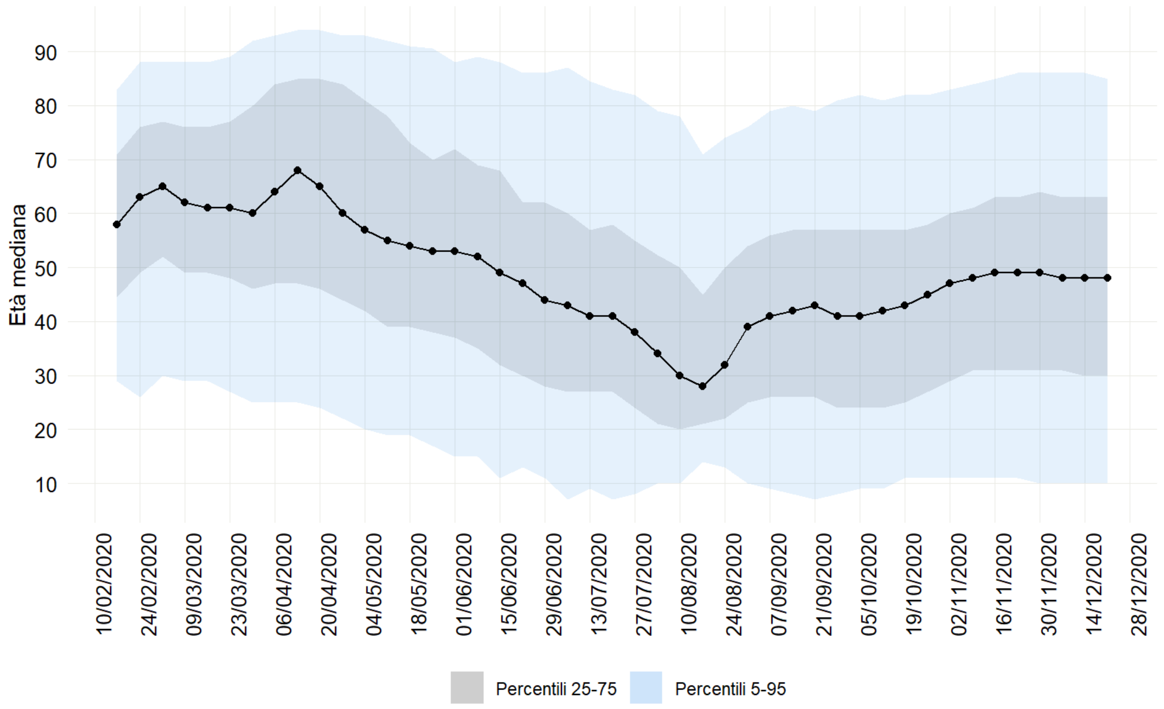

FIGURA 13 – ETÀ MEDIANA DEI CASI DI COVID-19 DIAGNOSTICATI IN ITALIA PER SETTIMANA DI DIAGNOSI

Nota: Ogni punto indica la mediana di ciascuna settimana (lunedì-domenica)

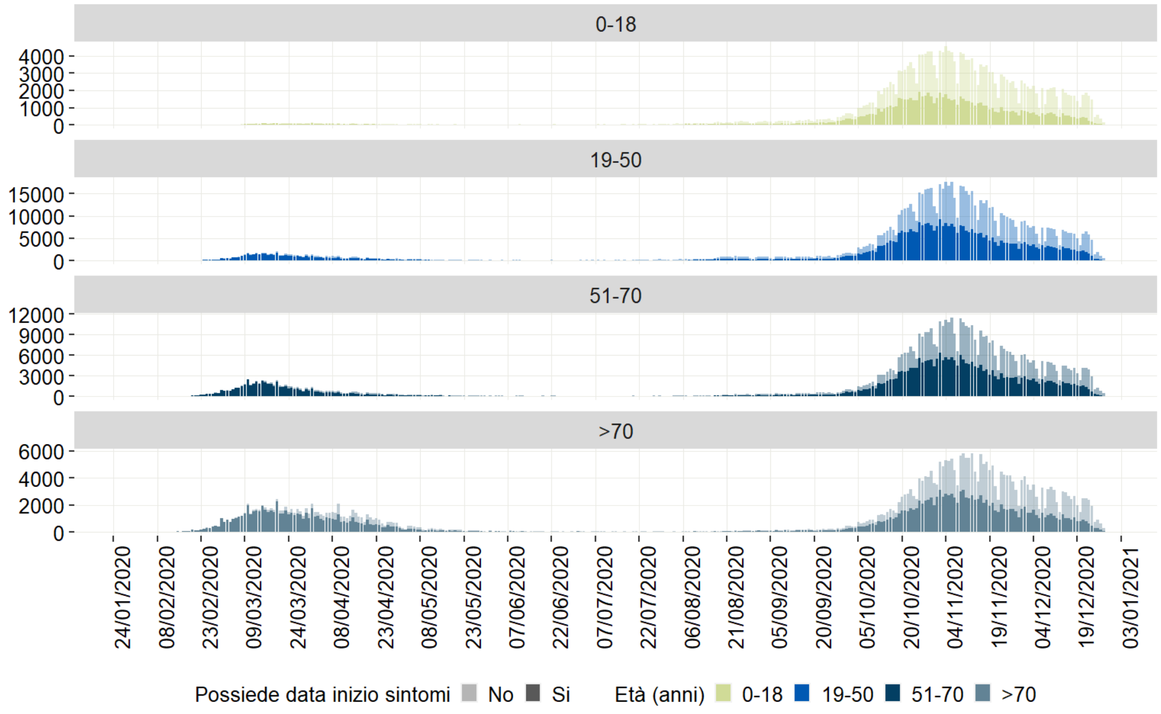

FIGURA 14 – CASI DI COVID-19 DIAGNOSTICATI IN ITALIA PER DATA INIZIO SINTOMI (O PRELIEVO/DIAGNOSI) PER CLASSE DI ETÀ

- La **Figura 14** mostra l'andamento dei casi (per data inizio sintomi o data prelievo/diagnosi se non disponibile la data inizio sintomi) per classe di età. Si osserva che a partire dall'inizio di maggio si è verificata una diminuzione importante del numero di casi in tutte le fasce di età ma con un decremento più marcato nei soggetti di età maggiore di 50 anni. Dalla metà di agosto, tuttavia, si osserva un nuovo incremento dei casi in questa fascia di età, mentre dalla fine di settembre si evidenzia un considerevole incremento dei casi in tutte le fasce di età, di cui molti asintomatici. Dalla metà di novembre si osserva un nuovo decremento in tutte le fasce di età.

- La **Figura 15** mostra la percentuale di casi per sesso nel tempo. Complessivamente si riscontra un numero di casi leggermente più elevato in persone di sesso femminile (51,5%), anche se nella fase iniziale dell'epidemia era superiore il numero di casi diagnosticato in persone di sesso maschile.

- La **Figura 16** mostra la variazione nel tempo del numero assoluto e della proporzione di casi confermati di COVID-19 per nazionalità (italiana/non italiana) e luogo (Italia/Estero) di acquisizione della malattia. La maggior parte dei casi segnalati è acquisita localmente e si osserva una diminuzione dei casi contratti all'estero, che nel periodo giugno-settembre avevano registrato un incremento. In particolare, nel mese di novembre, sono stati segnalati 2.754 (0,3%) casi di infezione da virus SARS-CoV-2 in cittadini italiani di ritorno da un viaggio all'estero, 777 (0,1%) invece il numero di casi tra i cittadini stranieri presenti in Italia con infezione contratta all'estero. Nel mese di dicembre, sono stati 397 (0,1%) i casi tra i cittadini italiani con infezione contratta fuori dal territorio nazionale e 205 (0,1%) i casi tra cittadini stranieri (dato soggetto ad

aggiornamento in base alle indagini epidemiologiche ancora in corso). Quindi, nel mese di novembre il 99,6% dei nuovi casi di infezione da SARS-CoV-2 segnalati è stato contratto in Italia e questa percentuale è salita al 99,8% nel mese di dicembre.

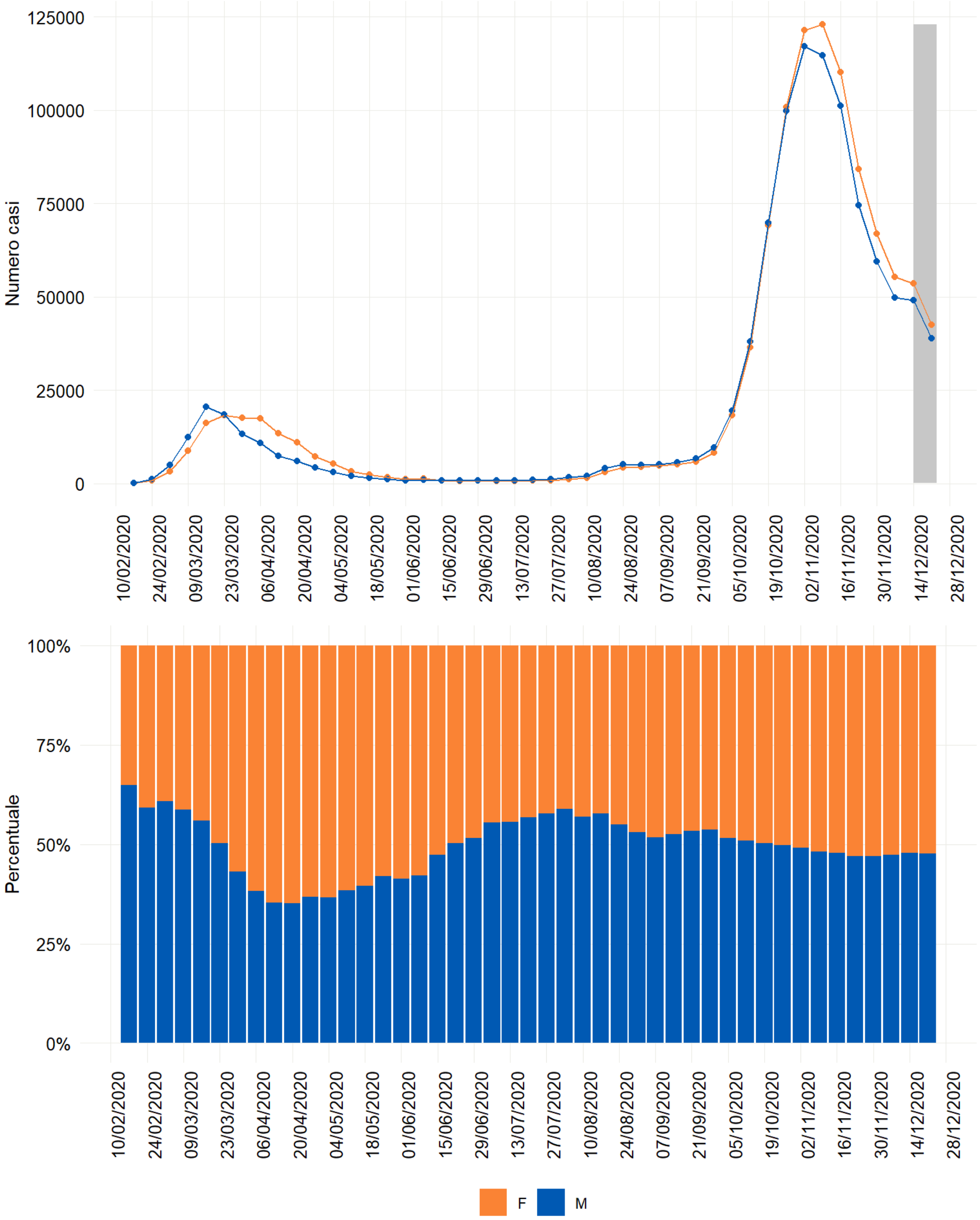

FIGURA 15 – NUMERO E PERCENTUALE DI CASI DI COVID-19 DIAGNOSTICATI IN ITALIA PER SESSO E SETTIMANA DI DIAGNOSI

Nota: Ogni punto e ogni barra indicano il numero e la percentuale di casi in ciascuna settimana (lunedì-domenica)

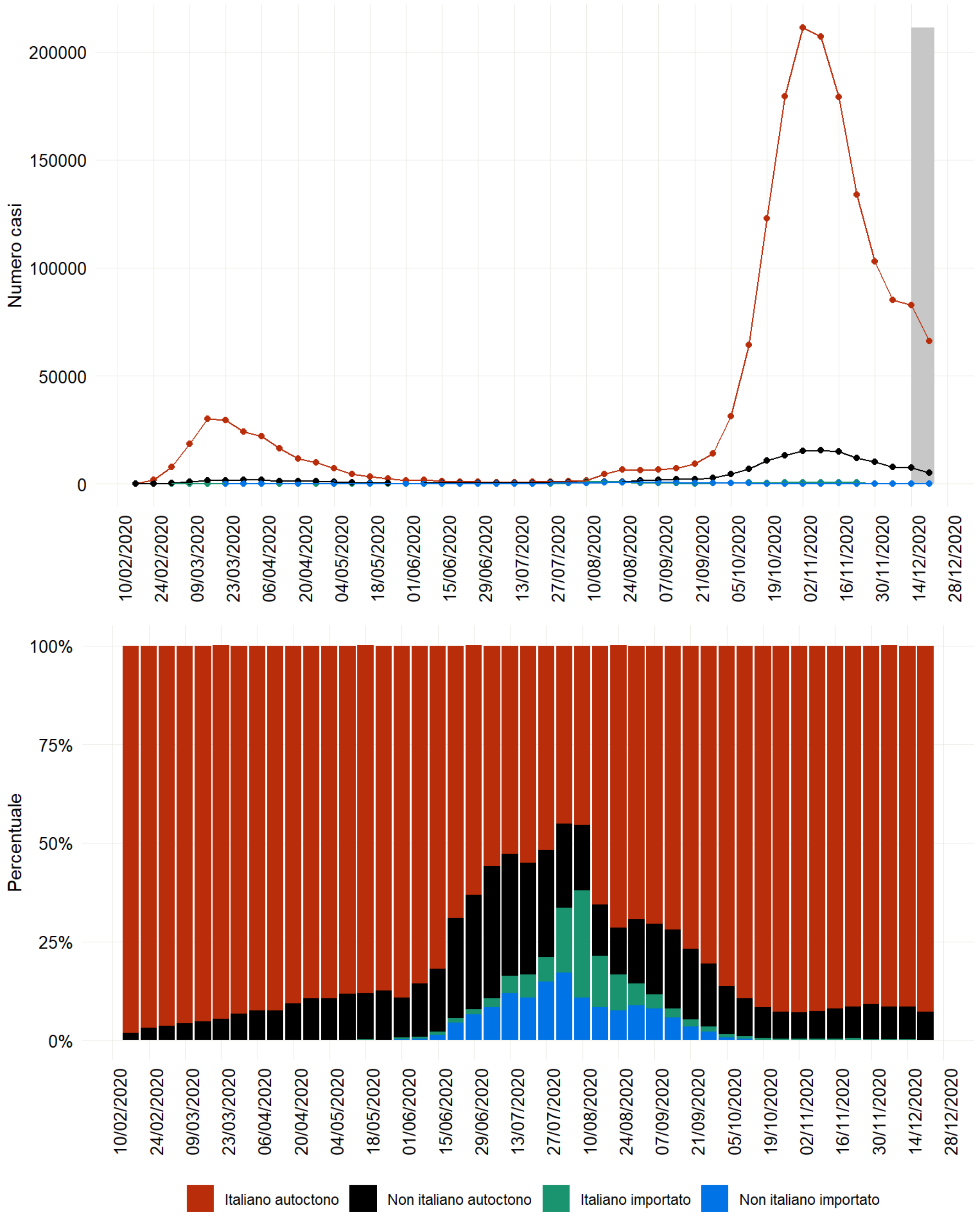

FIGURA 16 – NUMERO E PERCENTUALE DI CASI DI COVID-19 DIAGNOSTICATI IN ITALIA PER SETTIMANA DI DIAGNOSI, PER NAZIONALITA' E PER LUOGO DI ESPOSIZIONE

Nota: Ogni punto e ogni barra indicano il numero e la percentuale di casi in ciascuna settimana (lunedì-domenica)

- La **Figura 17** mostra il cambiamento nel tempo del quadro clinico riportato al momento della diagnosi dei casi confermati di COVID-19. Mentre nelle prime settimane dell'epidemia si riscontrava una maggiore percentuale di casi severi, critici e di casi già

deceduti al momento della diagnosi (diagnosticati mediante tamponi effettuali *post-mortem*), con il passare del tempo, si evidenzia, in percentuale, un netto incremento dei casi asintomatici o pauci-sintomatici e una marcata riduzione dei casi severi e dei decessi. La percentuale di casi asintomatici rispetto al totale dei casi diagnosticati è costante nelle ultime due settimane, come sono costanti le percentuali di casi con stato clinico lieve, severo o critico al momento della diagnosi.

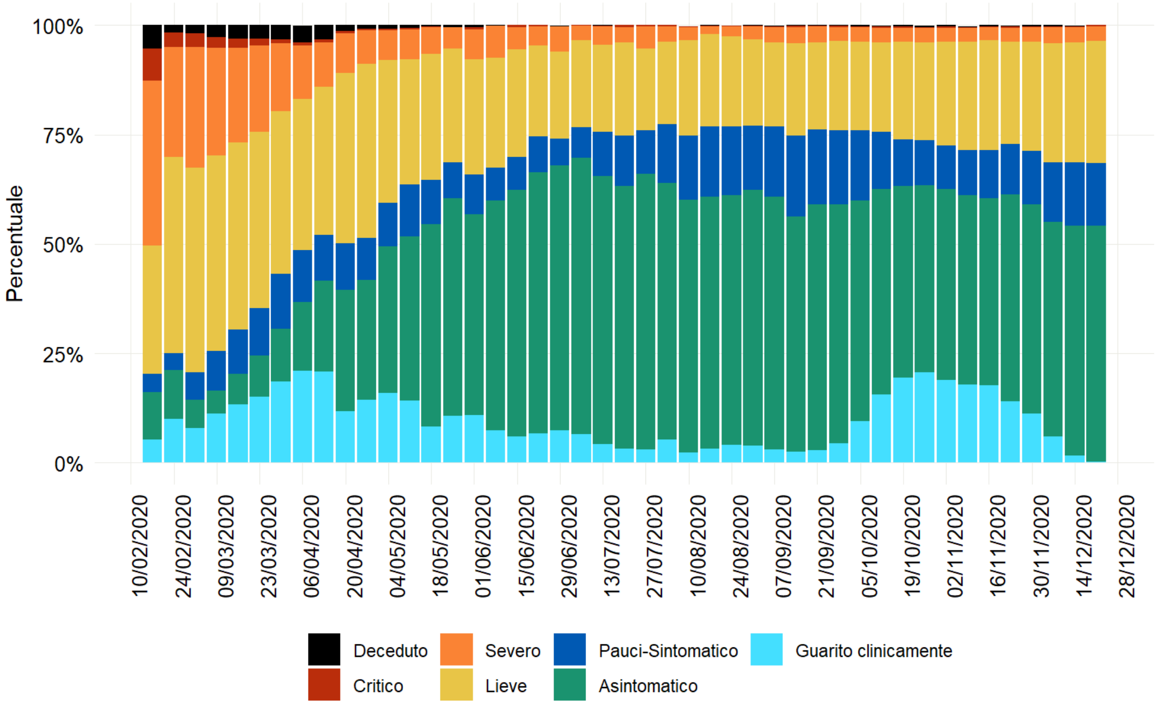

FIGURA 17 – PERCENTUALE DI CASI DI COVID-19 DIAGNOSTICATI IN ITALIA PER STATO CLINICO AL MOMENTO DELLA DIAGNOSI E SETTIMANA DI DIAGNOSI

- La **Tabella 6** mostra la distribuzione dei casi e dei decessi segnalati per sesso e fasce di età decennali. L'informazione sul sesso ed età è nota per il 99,9% dei casi segnalati (2.049.915); 1.056.165 casi sono di sesso femminile (51,5%). Nelle fasce di età 0-9, 10-19, 20-29, 60-69 e 70-79 anni si osserva un numero maggiore di casi di sesso maschile rispetto a quello di casi di sesso femminile. Inoltre, la tabella riporta il numero dei casi e la letalità per fascia di età e sesso. Si osserva un aumento della letalità con l'aumentare dell'età dei pazienti; inoltre, a partire dalla fascia di età 30-39 anni la letalità è più elevata nei soggetti di sesso maschile.

TABELLA 6 - DISTRIBUZIONE DEI CASI (N=2.049.934) E DEI DECESSI (N=70.799) PER COVID-19 DIAGNOSTICATI IN ITALIA PER FASCIA DI ETÀ E SESSO

Classe di età (anni)	Soggetti di sesso maschile					Soggetti di sesso femminile					Casi totali				
	N. casi	% casi totali	N. deceduti	% del totale deceduti	Letalità %	N. casi	% casi totali	N. deceduti	% del totale deceduti	Letalità %	N. casi	% casi per classe di età	N. deceduti	% deceduti per classe di età	Letalità %
0-9	40.723	51,8	5	55,6	0	37.941	48,2	4	44,4	0	78.664	3,8	9	0	0
10-19	88.983	52,3	6	60	0	81.065	47,7	4	40	0	170.048	8,3	10	0	0
20-29	124.114	50,6	19	54,3	0	121.340	49,4	16	45,7	0	245.458	12	35	0	0
30-39	123.311	49,1	94	62,7	0,1	127.911	50,9	56	37,3	0	251.226	12,3	150	0,2	0,1
40-49	152.263	46,6	421	69,9	0,3	174.307	53,4	181	30,1	0,1	326.571	15,9	602	0,9	0,2
50-59	178.171	48,3	1.765	74,3	1	190.462	51,7	611	25,7	0,3	368.635	18	2.376	3,4	0,6
60-69	122.158	53,3	5.066	74,3	4,1	107.041	46,7	1.752	25,7	1,6	229.200	11,2	6.818	9,6	3
70-79	89.991	52,3	12.058	68,9	13,4	82.080	47,7	5.435	31,1	6,6	172.071	8,4	17.493	24,7	10,2
80-89	60.854	40,6	16.096	55,1	26,5	89.092	59,4	13.128	44,9	14,7	149.953	7,3	29.224	41,3	19,5
≥90	13.119	22,6	4.751	33,7	36,2	44.869	77,4	9.329	66,3	20,8	57.988	2,8	14.080	19,9	24,3
Età non nota	63	52,5	2	100	3,2	57	47,5	-	0	0	120	0	2	0	1,7
**Totale**	**993.750**	**48,5**	**40.283**	**56,9**	**4,1**	**1.056.165**	**51,5**	**30.516**	**43,1**	**2,9**	**2.049.934**	**-**	**70.799**	**-**	**3,5**

NOTA: LA TABELLA NON INCLUDE I CASI PER CUI NON SONO NOTI IL SESSO E L'ETÀ (TABELLE PER SESSO) O L'ETÀ (TABELLA TOTALE)

- La **Figura 18** mostra, dall'alto verso il basso, la distribuzione dei casi per data di inizio sintomi, data di prelievo/diagnosi, data di ricovero e data di decesso. L'andamento delle curve è simile nelle quattro figure ma il raggiungimento del picco si sposta nel tempo. Infatti, nella prima ondata il picco della curva per data inizio sintomi è stato raggiunto intorno al 10 marzo, il picco per data prelievo/diagnosi e per ricovero è stato raggiunto intorno al 20 marzo, quello dei decessi è al 28 di marzo. Nella seconda ondata il picco per data di inizio sintomi è stato raggiunto, intorno al 25 ottobre mentre il picco per prelievo/diagnosi il 6 novembre.

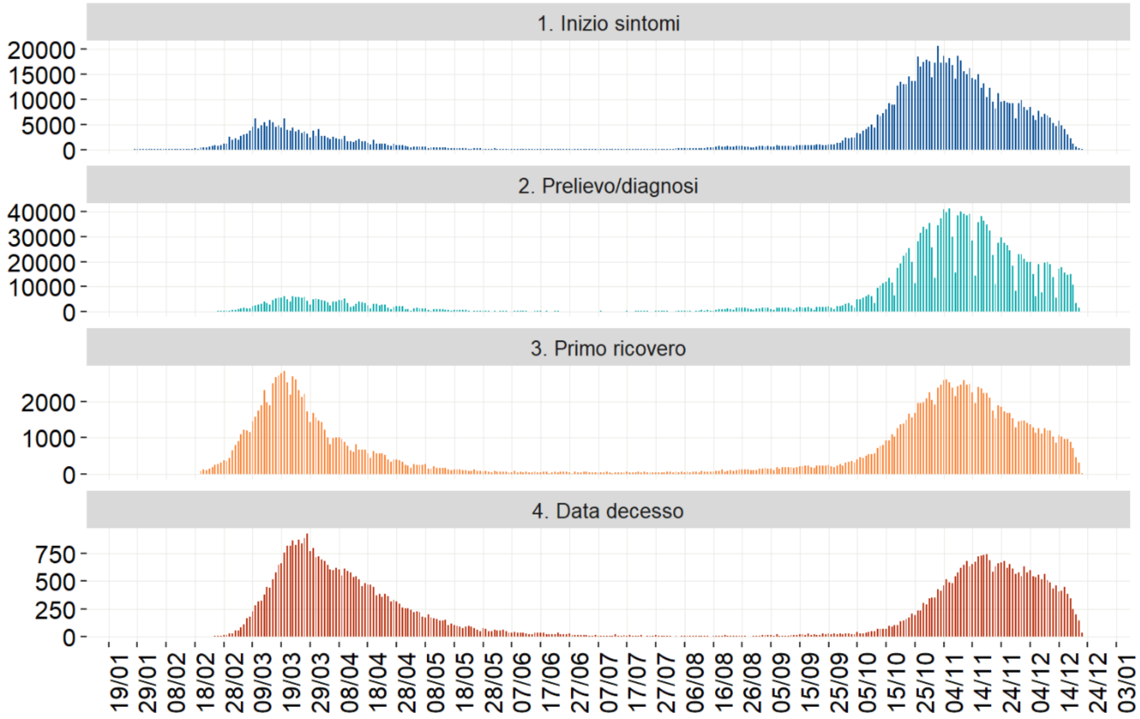

FIGURA 18 – CONFRONTO TRA I CASI DI COVID-19 DIAGNOSTICATI IN ITALIA PER DATA DI INIZIO SINTOMI, DATA DI PRELIEVO/DIAGNOSI, DATA DI RICOVERO E DATA DI DECESSO

- Al 29 dicembre 2020, risultano guariti 1.238.486 casi. Escludendo dal totale dei casi segnalati i casi guariti, quelli deceduti (70.799) e 1.640 casi persi al follow-up, l'informazione sulla gravità clinica dei pazienti affetti da COVID-19 è disponibile per 672.587/739.009 casi confermati (91,0%) riportati al sistema di sorveglianza. Tra questi, 400.138 (59,5%) risultano asintomatici, 81.559 (12,1%) sono pauci-sintomatici, 162.429 (24,1%) hanno sintomi lievi, 24.907 (3,7%) hanno sintomi severi e 3.554 (0,5%) presentano un quadro clinico critico.

- Escludendo i casi che risultano guariti, quelli deceduti e quelli persi al follow-up, l'informazione sulla collocazione del paziente è disponibile per 541.955/739.009 casi (73,0% del totale); in particolare, 512.385 (94,5%) stanno affrontando l'infezione presso il proprio domicilio/in altra struttura, 169 casi (0,1%) si trovano su una Nave Quarantena, 36 (0,1%) sono ricoverati presso il Policlinico Militare del Celio e 29.365 (5,3%) sono ospedalizzati. **Si sottolinea che i dati relativi allo stato clinico e alla collocazione del paziente sono dati soggetti a modifiche a causa dell'evoluzione dello stato clinico**

dei pazienti e al loro conseguente ricovero o dimissione. L'aggiornamento di queste variabili nel database della Sorveglianza Integrata Nazionale coordinata dall'ISS che, si ricorda, contiene dati individuali richiede tempo, e di conseguenza il dato può risultare leggermente disallineato da quello fornito dal flusso di dati aggregati coordinato dal Ministero della Salute.

- La **Figura 19** mostra l'andamento dei dati aggregati, riportati dal Ministero della Salute al 29 dicembre 2020, per condizione di ricovero, isolamento domiciliare ed esito dei casi confermati di COVID-19**.** La **Figura 20** mostra la distribuzione percentuale di casi diagnosticati quotidianamente per condizione di isolamento domiciliare e ricovero.

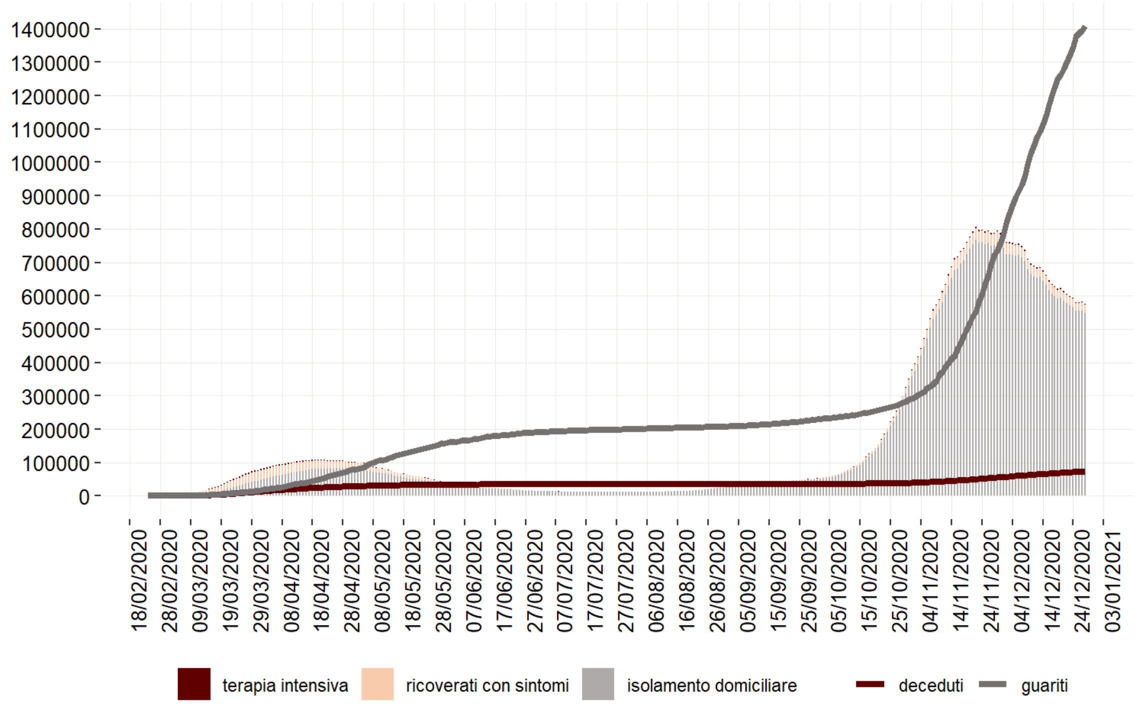

FIGURA 19 – NUMERO TOTALE DI CASI DI COVID-19 (ESCLUSI GUARITI E DECEDUTI) DIAGNOSTICATI IN ITALIA PER STATO DI RICOVERO/ISOLAMENTO E NUMERO CUMULATIVO DELL'ESITO (N=2.083.689) AL 29/12/2020 (FONTE DATI MINISTERO DELLA SALUTE E PROTEZIONE CIVILE).

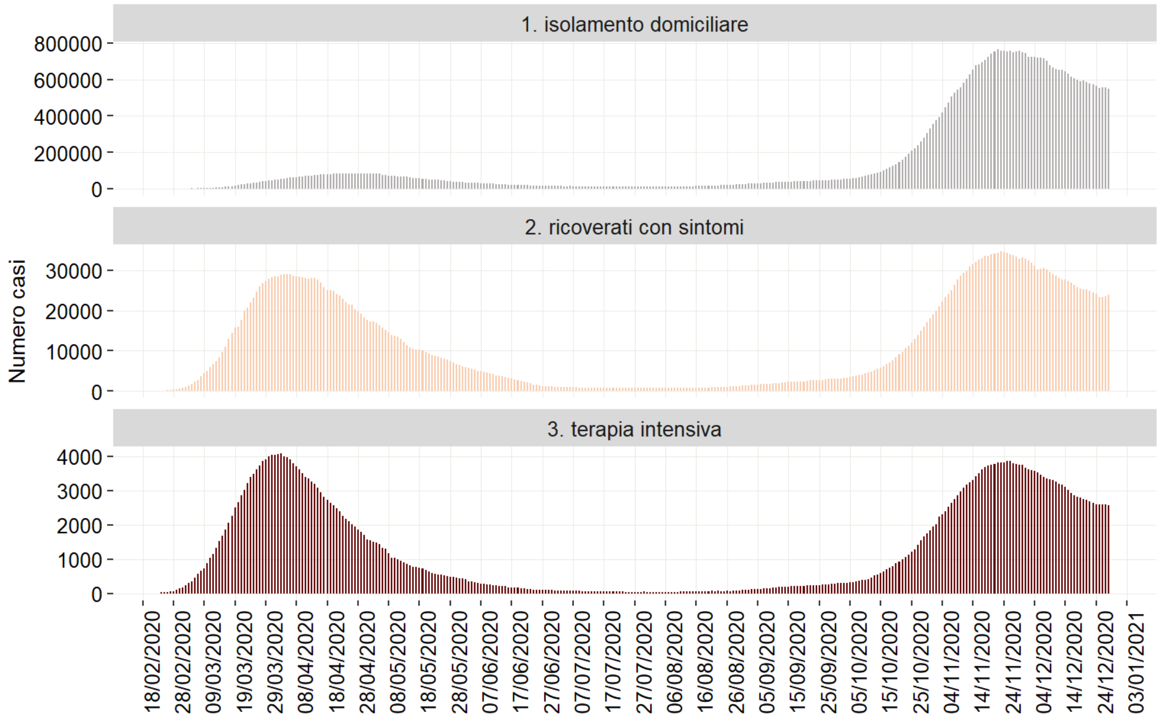

FIGURA 20 – NUMERO DI CASI DI COVID-19 DIAGNOSTICATI IN ITALIA QUOTIDIANAMENTE PER STATO DI ISOLAMENTO/RICOVERO (FONTE DATI MINISTERO DELLA SALUTE E PROTEZIONE CIVILE)

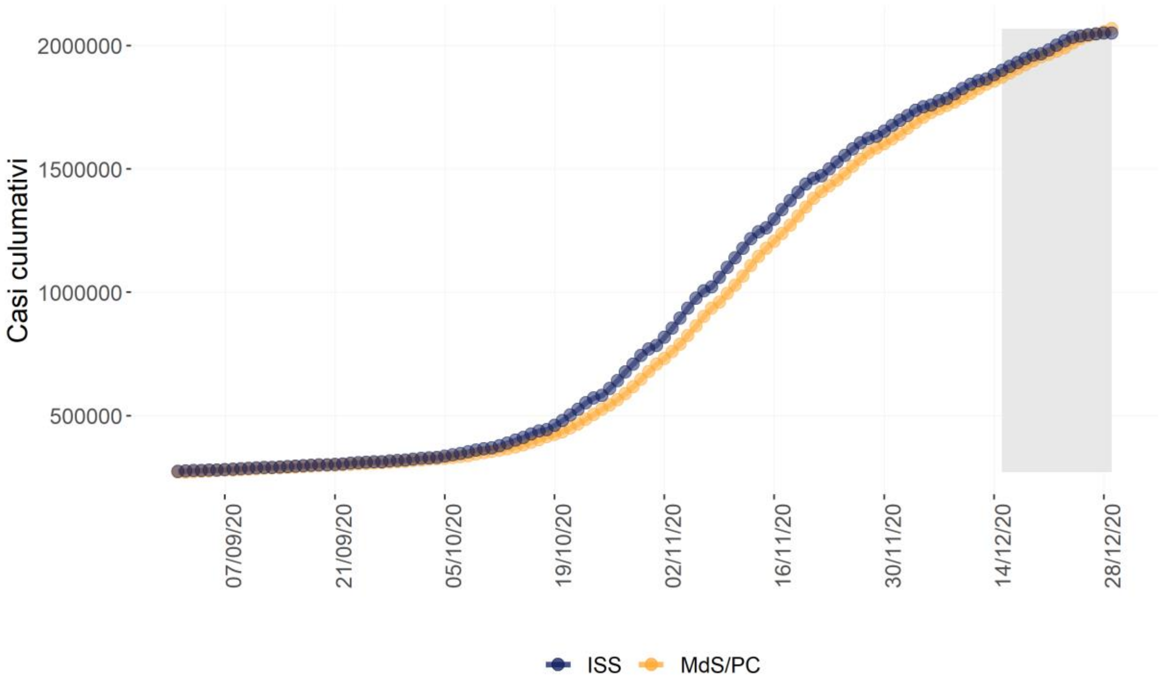

FIGURA 21 – CONFRONTO TRA I CASI NOTIFICATI QUOTIDIANAMENTE ATTRAVERSO LA PIATTAFORMA COVID-19 DELL'ISS E I CASI COMUNICATI AL MINISTERO DELLA SALUTE/PROTEZIONE CIVILE IN BASE ALLA DATA DI PRELIEVO/DIAGNOSI O ALLA DATA DI NOTIFICA. AGGIORNATO AL 29 DICEMBRE 2020

- La **Figura 21** riporta un confronto dei casi cumulativi riportati al flusso aggregato del Ministero della Salute/Protezione Civile (per data di notifica) e a quello individuale dell'ISS (per data di prelievo/diagnosi). Si evidenzia che i dati aggregati e i dati individuali sono stati allineati fino alla fine di settembre e, come atteso, i dati del flusso ISS, basati su data di prelievo/diagnosi "anticipano" la notifica del dato aggregato. Nelle ultime tre settimane i casi individuali riportati alla sorveglianza integrata dell'ISS e il numero di casi aggregati sono sostanzialmente sovrapponibili.

- Dall'inizio dell'epidemia sono stati diagnosticati 91.270 casi tra gli operatori sanitari (età mediana 47 anni) pari al 4% dei casi totali segnalati. La **Tabella 7** riporta la distribuzione dei casi segnalati per classe di età e la letalità osservata in questa popolazione.

TABELLA 7 - DISTRIBUZIONE DEI CASI (N=91.270) E DEI DECESSI (N=188) PER COVID-19 DIAGNOSTICATI NEGLI OPERATORI SANITARI IN ITALIA PER FASCIA DI ETÀ E SESSO

Classe di età (anni)	Soggetti di sesso maschile					Soggetti di sesso femminile					Casi totali				
	N. casi	% casi totali	N. deceduti	% del totale deceduti	Letalità %	N. casi	% casi totali	N. deceduti	% del totale deceduti	Letalità %	N. casi	% casi per classe di età	N. deceduti	% deceduti per classe di età	Letalità %
18-29	3.319	12,5	0	0	0%	8.085	12,5	0	0	0%	11.404	12,5	0	0	0%
30-39	5.766	21,8	1	0,7	0%	11.461	17,7	1	2	0%	17.227	18,9	2	1,1	0%
40-49	5.821	22	6	4,4	0,10%	18.906	29,2	4	7,8	0%	24.727	27,1	10	5,3	0%
50-59	7.025	26,5	26	19	0,40%	21.100	32,6	16	31,4	0,10%	28.125	30,8	42	22,3	0,10%
60-69	4.143	15,6	68	49,6	1,60%	4.818	7,4	13	25,5	0,30%	8.961	9,8	81	43,1	0,90%
70-79	282	1,1	19	13,9	6,70%	158	0,2	2	3,9	1,30%	440	0,5	21	11,2	4,80%
Età non nota	151	0,6	17	12,4	11,30%	235	0,4	15	29,4	6,40%	386	0,4	32	17	8,30%
**Totale**	**26.507**	**29**	**137**	**72,9**	**0,50%**	**64.763**	**71**	**51**	**27,1**	**0,10%**	**91.270**	**-**	**188**	**-**	**0,20%**

NOTA: LA TABELLA NON INCLUDE I CASI PER CUI NON È NOTO IL SESSO

- I dati riportati dalle Regioni/PPAA indicano che la letalità tra gli operatori sanitari è inferiore, anche a parità di classe di età (**Tabella 7**), rispetto alla letalità totale (vedi **Tabella 6**), verosimilmente perché gli operatori sanitari asintomatici e pauci-sintomatici vengono maggiormente testati rispetto alla popolazione generale,

- La **Figura 22** riporta la proporzione di casi tra operatori sanitari sul totale dei casi segnalati in Italia per periodo di diagnosi (ogni 7 giorni). Da metà novembre la percentuale di operatori sanitari ha superato il 5% del totale dei casi, anche se nelle ultime due settimane si rileva un lieve decremento che potrà essere confermato nelle prossime settimane (dato ancora in fase di consolidamento).

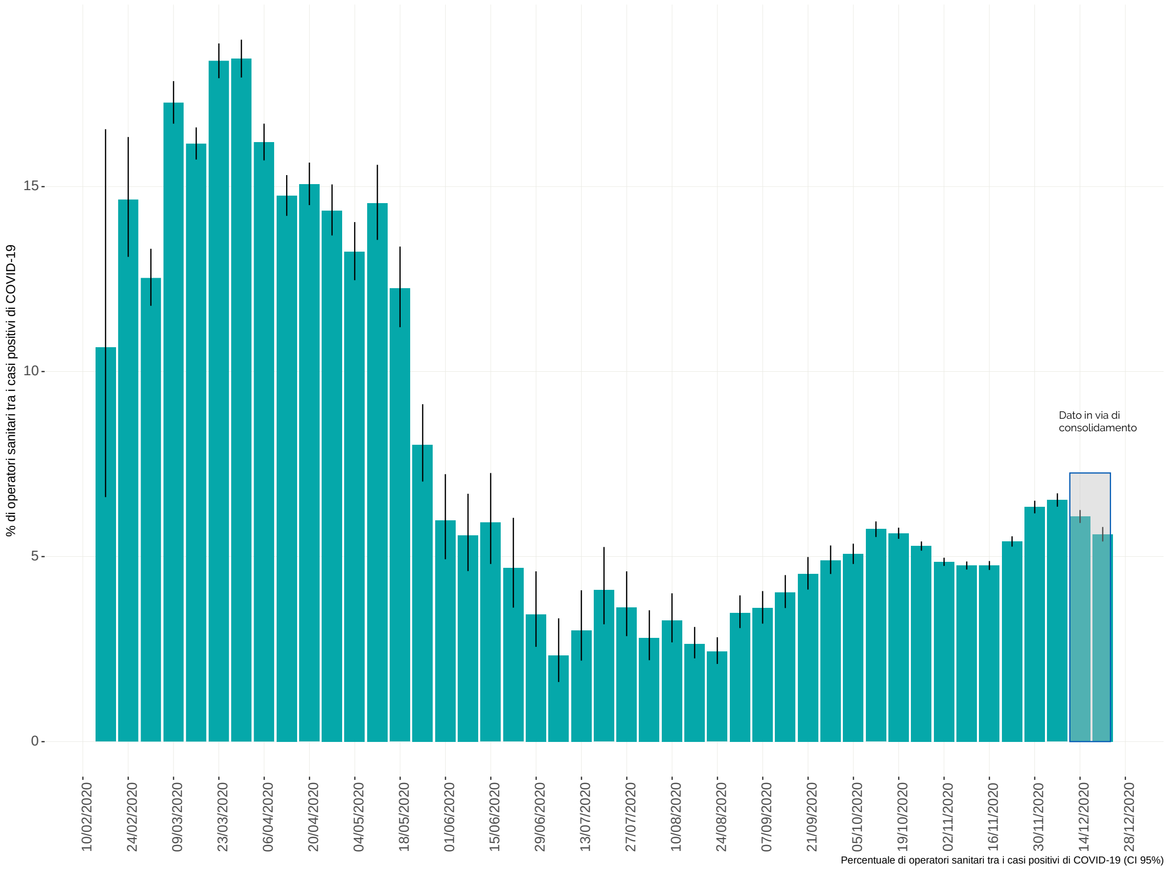

FIGURA 22 – PERCENTUALE DI OPERATORI SANITARI RIPORTATI SUL TOTALE DEI CASI DIAGNOSTICATI I ITALIA PER PERIODO DI DIAGNOSI (7 GIORNI)

NOTA: OGNI BARRA FA RIFERIMENTO ALL'INTERVALLO DI TEMPO TRA LA DATA INDICATA SOTTO LA BARRA E QUELLA SUCCESSIVA (ESEMPIO: 17 FEB SI RIFERISCE AL PERIODO DAL 19-23 FEB, 24 FEB SI RIFERISCE AL PERIODO DAL 24-30 FEB, ETC.),

Focus: Incidenza per fascia d'età nazionale e per Regione/PA

- La **Figura 23** riporta il tasso d'incidenza per fascia d'età a livello nazionale a partire dal 10 agosto 2020 (inizio della seconda ondata dell'epidemia). La fascia di età >90 anni è quella con l'incidenza maggiore, pari a 4.857 per 100.000 abitanti, mentre l'incidenza minore si rileva nella fascia 0-9 anni (1.528 per 100.000 abitanti). Il picco di incidenza viene raggiunto nella settimana del 26/10 per la fascia di età 10-19 anni e quella del 9/11 per la fascia >90 anni. Dalla settimana del 9/11 si osserva un decremento in tutte le fasce d'età.

- Le **Figure 24A e 24B** riportano il tasso d'incidenza per fascia d'età per Regione/PA nazionale a partire dal 10 agosto 2020.

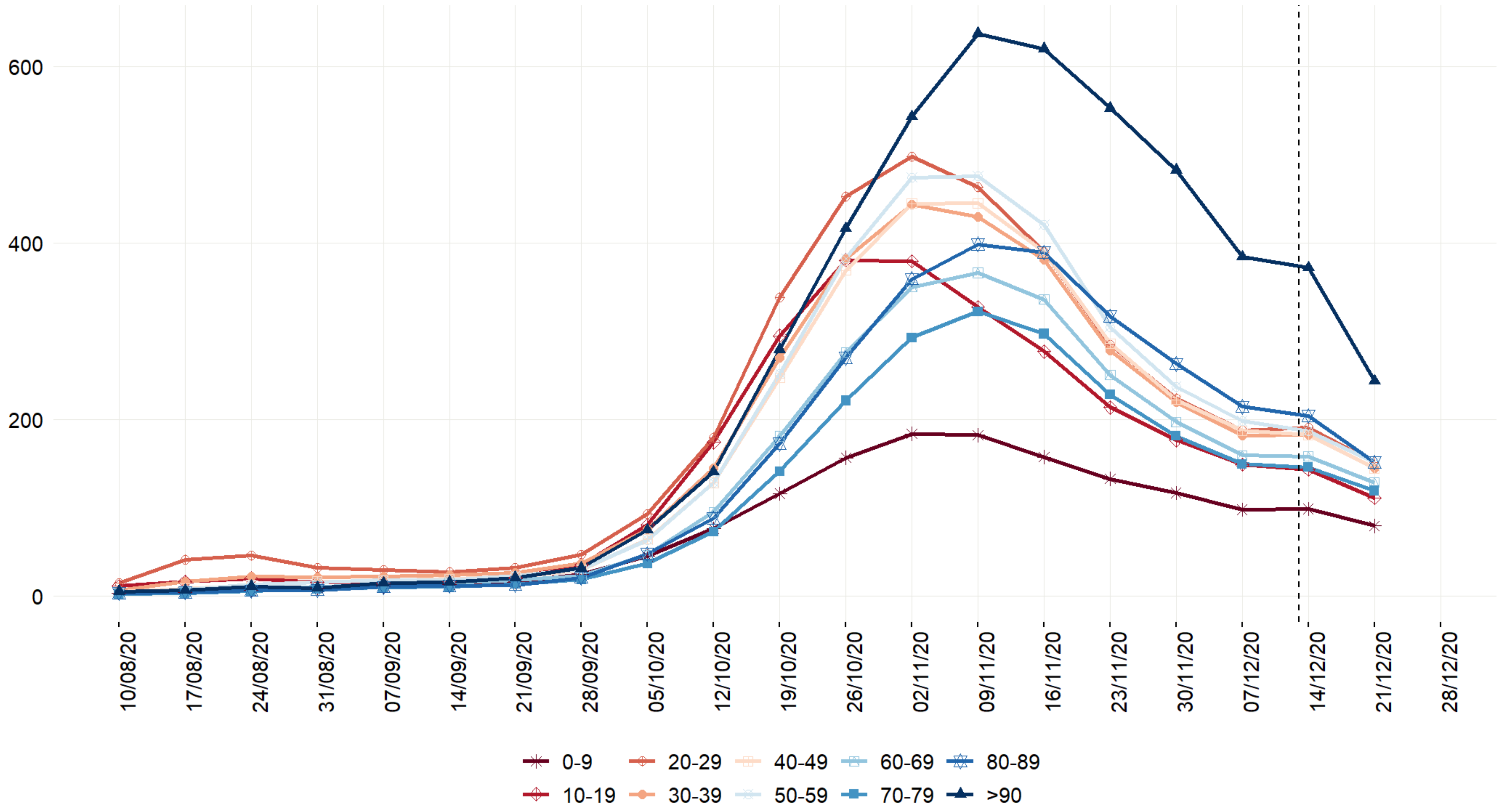

FIGURA 23 – INCIDENZA PER FASCIA D'ETÀ NAZIONALE

Nota: Oltre la linea tratteggiata il dato deve essere considerato provvisorio

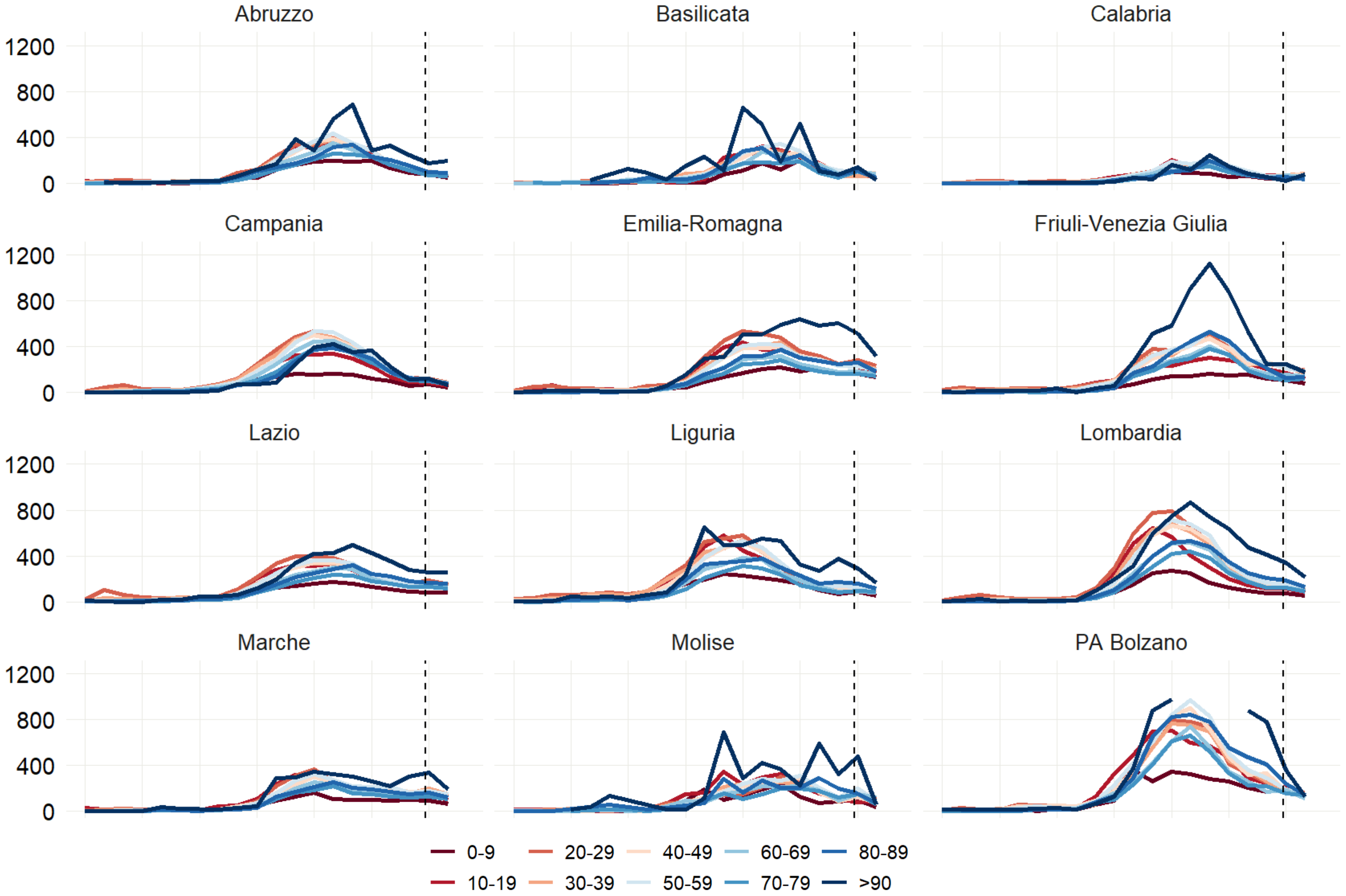

FIGURA 24A – INCIDENZA PER FASCIA D'ETÀ REGIONE/PA

Nota: Per una migliore visualizzazione l'asse delle ordinate è stato troncato a 1.250 casi (per 100.000 abitanti). Oltre la linea tratteggiata il dato deve essere considerato provvisorio

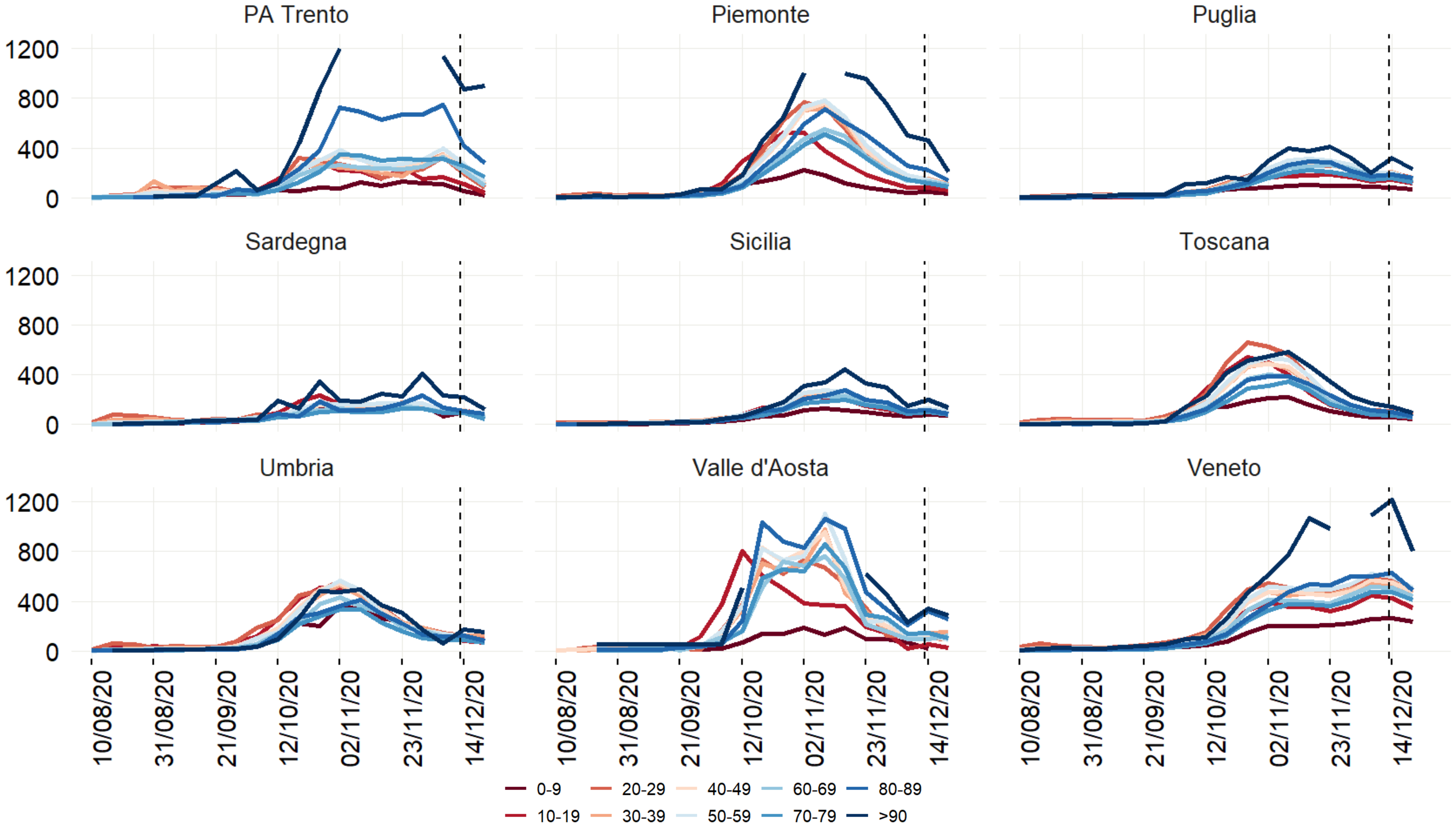

FIGURA 24B – INCIDENZA PER FASCIA D'ETÀ REGIONALE

Nota: Per una migliore visualizzazione l'asse delle ordinate è stato troncato a 1.250 casi (per 100.000 abitanti). Oltre la linea tratteggiata il dato deve essere considerato provvisorio

2021

Epidemia COVID-19

Aggiornamento nazionale
14 aprile 2021 – ore 12:00

DATA PUBBLICAZIONE: 16 APRILE 2021

Prodotto dall'Istituto Superiore di Sanità (ISS), Roma

A cura di: Flavia Riccardo, Xanthi Andrianou, Antonino Bella, Martina Del Manso, Alberto Mateo Urdiales, Massimo Fabiani, Stefania Bellino, Stefano Boros, Fortunato (Paolo) D'Ancona, Maria Cristina Rota, Antonietta Filia, Ornella Punzo, Matteo Spuri, Maria Fenicia Vescio, Daniele Petrone, Corrado Di Benedetto, Marco Tallon, Alessandra Ciervo, Paola Stefanelli, Patrizio Pezzotti per ISS;
Giorgio Guzzetta, Valentina Marziano, Piero Poletti, Filippo Trentini, Marco Ajelli, Stefano Merler per Fondazione Bruno Kessler;
e di: Antonia Petrucci (Abruzzo); Michele La Bianca (Basilicata); Anna Domenica Mignuoli (Calabria); Pietro Buono (Campania); Erika Massimiliani (Emilia-Romagna); Fabio Barbone (Friuli Venezia Giulia); Francesco Vairo (Lazio); Camilla Sticchi (Liguria); Danilo Cereda (Lombardia); Lucia Di Furia (Marche); Francesco Sforza (Molise); Annamaria Bassot (P.A. Bolzano); Pier Paolo Benetollo (P.A. Trento); Chiara Pasqualini (Piemonte); Lucia Bisceglia (Puglia); Maria Antonietta Palmas (Sardegna); Salvatore Scondotto (Sicilia); Emanuela Balocchini (Toscana); Anna Tosti (Umbria); Mauro Ruffier (Valle D'Aosta); Filippo Da Re (Veneto).

Citare il documento come segue: Task force COVID-19 del Dipartimento Malattie Infettive e Servizio di Informatica, Istituto Superiore di Sanità. Epidemia COVID-19, Aggiornamento nazionale: 14 aprile 2021

EPIDEMIA COVID-19

Aggiornamento nazionale

14 aprile 2021 – *ore 12:00*

Nota di lettura: *Questo bollettino è prodotto dall'Istituto Superiore di Sanità (ISS) e riporta i dati della sorveglianza integrata dei casi di infezione da virus SARS-CoV-2 riportati sul territorio nazionale e coordinata dall'ISS ai sensi dell'Ordinanza n. 640 del 27 febbraio 2020.*
I dati vengono raccolti attraverso una piattaforma web dedicata e riguardano tutti i casi di infezione da virus SARS-CoV-2 confermati tramite positività al test molecolare standard.
I dati vengono aggiornati giornalmente da ciascuna Regione/PA anche se alcune informazioni possono richiedere qualche giorno per il loro inserimento e/o aggiornamento. Per questo motivo, potrebbe non esserci una completa concordanza con quanto riportato attraverso il flusso informativo del Ministero della Salute che riporta dati aggregati.
I dati raccolti sono in continua fase di consolidamento e, come prevedibile in una situazione emergenziale, alcune informazioni sono incomplete. In particolare, si segnala la possibilità di un ritardo di alcuni giorni tra il momento della esecuzione del tampone per la diagnosi e la segnalazione sulla piattaforma dedicata. Pertanto, il numero casi che si osserva nei giorni più recenti, deve essere interpretato come provvisorio.
Il bollettino descrive, con grafici, mappe e tabelle la diffusione, nel tempo e nello spazio, dell'epidemia di COVID-19 in Italia. Fornisce, inoltre, una descrizione delle caratteristiche delle persone affette.
La forte pressione sui dipartimenti di prevenzione, causata dall'intensificazione dell'epidemia e dal conseguente forte aumento dei casi, porta in alcune aree a un ritardo nella notifica e nell'aggiornamento delle informazioni sui casi individuali

Prodotto dall'Istituto Superiore di Sanità (ISS), Roma, 14 aprile 2021

In evidenza

- **Questa settimana si osserva una forte diminuzione della incidenza settimanale** (160,5 per 100.000 abitanti (05/04/2021-11/04/2021) vs 210,8 per 100.000 abitanti (29/03/2021-04/04/2021)
- Nelle ultime due settimane si rileva un **lievissimo aumento della percentuale di** casi nella fascia di età 0-18 anni pari al 15,8% (nelle 2 settimane precedenti era del 15,3%), diminuisce leggermente la proporzione di casi nella fascia di età 19-50 anni (43,1% vs 43,4%) e nella fascia d'età >50 anni (41,1% vs 41,3%).
- A partire dalla seconda metà di gennaio **si osserva un trend in diminuzione del numero di casi negli operatori sanitari e nei soggetti di età >= 80 anni**, verosimilmente ascrivibile alla campagna di vaccinazione in corso.
- Nel periodo 24 marzo – 06 aprile 2021, l'Rt medio calcolato sui casi sintomatici è stato pari a **0,85 (range 0,71– 0,97),** in diminuzione rispetto alla settimana precedente e sotto l'uno anche nel limite superiore. Per dettagli sulle modalità di calcolo ed interpretazione dell'Rt riportato si rimanda all'approfondimento disponibile sul sito dell'Istituto Superiore di Sanità (https://www.iss.it/primo-piano/-/asset_publisher/o4oGR9qmvUz9/content/id/5477037).

Raccomandazioni

- Si ribadisce, anche alla luce della conferma della circolazione di alcune varianti virali a maggiore trasmissibilità di mantenere la drastica riduzione delle interazioni fisiche tra le persone e della mobilità. Analogamente a quanto avviene in altri paesi Europei, si raccomanda il rafforzamento/innalzamento delle misure su tutto il territorio nazionale.
- È fondamentale che **la popolazione eviti tutte le occasioni di contatto con persone al di fuori del proprio nucleo abitativo** che non siano strettamente necessarie e di rimanere a casa il più possibile. Si ricorda che è obbligatorio adottare comportamenti individuali rigorosi e rispettare le misure igienico-sanitarie predisposte relative a distanziamento e uso corretto delle mascherine. Si ribadisce la necessità di rispettare le misure raccomandate dalle autorità sanitarie compresi i provvedimenti quarantenari dei contatti stretti dei casi accertati e di isolamento dei casi stessi.
- È importante continuare il rafforzamento dei servizi territoriali, attraverso un coinvolgimento straordinario di risorse professionali di supporto ed anche attraverso l'utilizzo di strumenti tecnologici come **la "app" Immuni**, nelle attività di ricerca dei contatti in modo da identificare precocemente tutte le catene di trasmissione e garantire una efficiente gestione, inclusa la quarantena dei contatti stretti e l'isolamento immediato dei casi secondari.
- **Si continua a richiamare l'importanza dell'uso appropriato degli strumenti diagnostici e di screening, nel contesto di una valutazione del rischio epidemiologico, e della corretta esecuzione delle procedure di isolamento e quarantena quando indicate.**
- **Si ribadisce la necessità di rispettare i provvedimenti quarantenari e le altre misure raccomandate dalle autorità sanitarie sia per le persone che rientrano da paesi per i quali è prevista la quarantena, e sia a seguito di richiesta dell'autorità sanitaria essendo stati individuati come contatti stretti di un caso**. Sebbene i servizi territoriali siano riusciti finora a contenere la trasmissione locale del virus, viene ripetutamente segnalato un carico di lavoro eccezionale che in molti casi compromette la tempestiva gestione dei contatti oltre che non assicurare le attività non-collegate a questa emergenza
- **Si raccomanda alla popolazione di continuare a prestare particolare attenzione al rischio di contrarre l'infezione in tutti i casi di mancato rispetto delle misure raccomandate. Si invita a rispettare tutte le norme comportamentali previste di**

prevenzione della trasmissione di SARS-CoV-2, in particolare nei confronti di fasce di popolazione più vulnerabili.

La situazione nazionale nelle ultime due settimane (29 marzo - 11 aprile 2021)

- Durante il periodo 29 marzo - 11 aprile 2021 sono stati diagnosticati e segnalati 225.516 nuovi casi, di cui 1.141 deceduti (questo numero non include le persone decedute nel periodo con una diagnosi antecedente al 28 marzo). **Si sottolinea che a causa della forte pressione sui dipartimenti di prevenzione si continuano a registrare dei ritardi nella notifica e nell'aggiornamento tempestivo delle informazioni dei casi individuali, che rendono il quadro più recente in parte sottostimato sia per le nuove diagnosi che per i decessi.**

- 1.889 (1%) casi si sono verificati in operatori sanitari. Anche questo numero è verosimilmente sottostimato in quanto questa informazione può richiedere tempi più lunghi per un suo consolidamento.

- La maggior parte dei casi sono stati notificati dalle regioni Lombardia (N=38.521), Campania (N= 23.668), Piemonte (N=21.777), Puglia (N= 21.024), Lazio (N= 20.007), Emilia-Romagna (N= 17.792), Toscana (N= 16.641) e Veneto (N= 15.520).

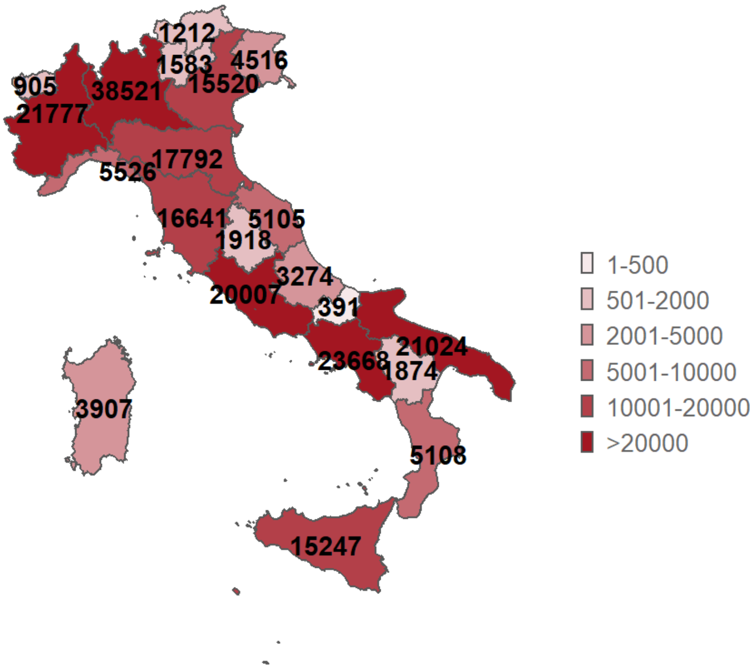

FIGURA 1 – CASI DI COVID-19 DIAGNOSTICATI IN ITALIA PER REGIONE/PA DI NOTIFICA. PERIODO: 29 MARZO - 11 APRILE 2021

- Nel 41,1% dei casi le persone segnalate al sistema di sorveglianza nelle ultime due settimane hanno un'età superiore a 50 anni e il 15,8% ha meno di 19 anni (età mediana 46 anni (0-106 aa)); il 49,6% dei casi sono di sesso maschile (**Figura 3** e **Figura 4**).

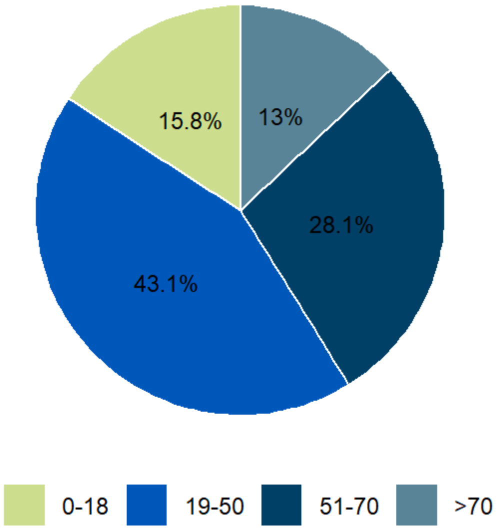

FIGURA 3 - DISTRIBUZIONE PER ETÀ DEI CASI DI COVID-19 DIAGNOSTICATI IN ITALIA PERIODO: 29 MARZO - 11 APRILE 2021

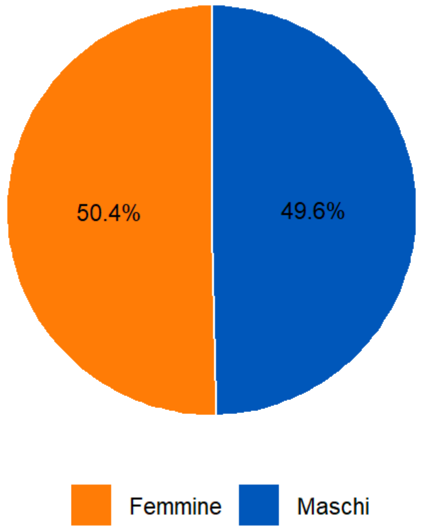

FIGURA 3 - DISTRIBUZIONE PER SESSO DEI CASI DI COVID-19 DIAGNOSTICATI IN ITALIA PERIODO: 29 MARZO - 11 APRILE 2021

- La **Figura 4** mostra la distribuzione dei nuovi casi di infezione da virus SARS-CoV-2 per comune di domicilio/residenza riportati al Sistema di Sorveglianza Integrato Nazionale COVID-19. Nella mappa sono riportati 221.919 casi rispetto ai 225.516 segnalati nel periodo 29 marzo - 11 aprile 2021 (2.157 casi sono stati esclusi poiché non è nota l'informazione sul domicilio/residenza e 1.440 casi hanno un domicilio/residenza che non si trova nella Regione/PA di diagnosi). I casi sono distribuiti in 6.500 comuni con un'importante diffusione su tutto il territorio nazionale.

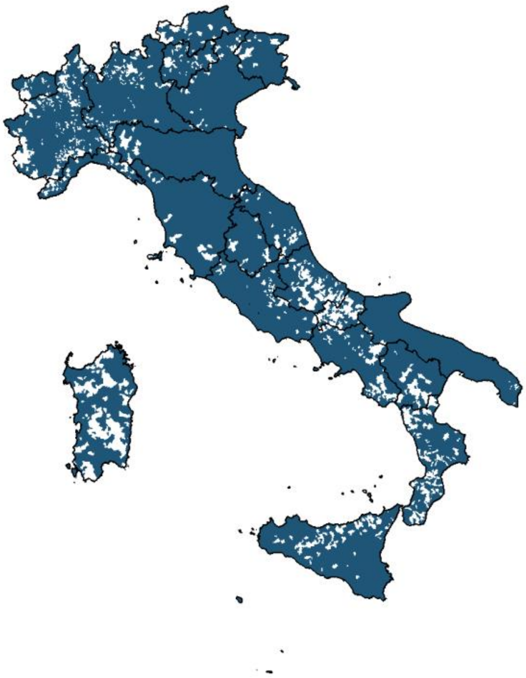

FIGURA 4 – CASI DI COVID-19 DIAGNOSTICATI IN ITALIA PER COMUNE DI DOMICILIO/RESIDENZA
(COMUNI CON ALMENO UN CASO).
PERIODO: 29 MARZO - 11 APRILE 2021

- La **Tabella 1** e la **Tabella 2** riportano rispettivamente il motivo per cui i nuovi casi diagnosticati nel periodo di riferimento sono stati sottoposti ad accertamento diagnostico e l'origine dei casi di Covid-19 diagnosticati in Italia.

TABELLA 1 – DISTRIBUZIONE DEL MOTIVO PER CUI I CASI DI COVID-19 DIAGNOSTICATI IN ITALIA SONO STATI TESTATI – PERIODO: 29 MARZO - 11 APRILE 2021

Motivo del test	Casi	
	N	%
Screening	46.589	20,7
Contact tracing	70.292	31,2
Paziente con sintomi	76.367	33,8
Non noto	32.268	14,3
**Totale**	**225.516**	

TABELLA 2 – DISTRIBUZIONE DELL'ORIGINE DEI CASI DI COVID-19 DIAGNOSTICATI IN ITALIA. PERIODO: 29 MARZO - 11 APRILE 2021

Origine dei casi	Casi	
	N	%
Autoctoni	182.320	80,8
Importati dall'estero	339	0,2
Provenienti da Regione diversa da quella di notifica	231	0,1
Non noto	42.625	18,9
**Totale**	**225.516**	

La situazione delle regioni nelle ultime due settimane (29 marzo - 11 aprile 2021)

- La **Tabella 3** riporta il numero dei casi totali dall'inizio dell'epidemia, l'incidenza cumulativa (per 100.000 abitanti), il numero di casi e l'incidenza nell'ultima settimana (5 - 11 aprile 2021) e negli ultimi 14 giorni (29 marzo - 11 aprile 2021) per Regione/PA e per l'Italia. Negli ultimi 14 giorni il valore nazionale di incidenza è 378,12 casi per 100.000 abitanti, con una diminuzione rispetto alle due settimane precedenti (450,65 casi/100.000 abitanti). La distribuzione dei casi nelle ultime due settimane non è uniforme nelle regioni: la Valle d'Aosta riporta la maggiore incidenza con 723,80 casi per 100.000 abitanti, mentre il Molise che ha l'incidenza più bassa riporta un valore pari a 130,11 casi per 100.000 abitanti. Sette Regioni/PA (Valle d'Aosta, Puglia, Piemonte, Toscana, Campania, Emilia-Romagna, Lombardia,) riportano un'incidenza pari o superiore al valore nazionale (**Figura 5, Tabella 3**).

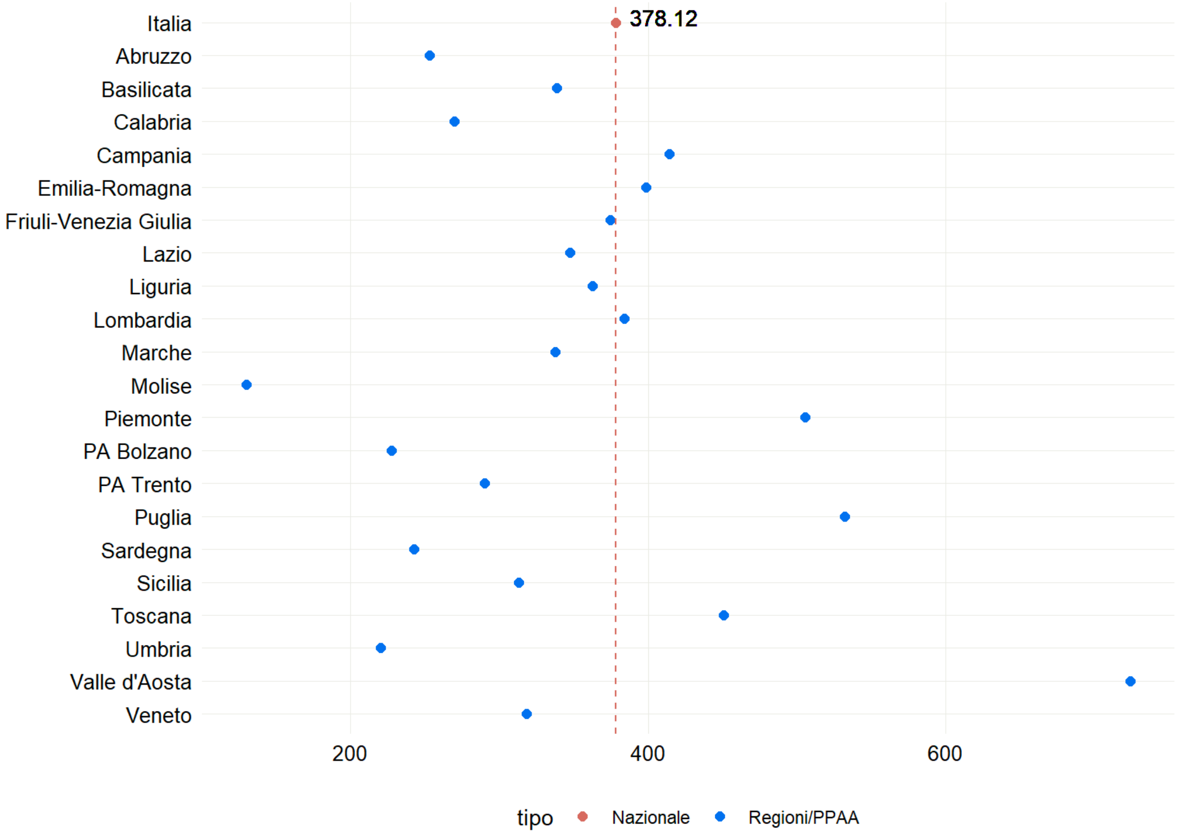

FIGURA 5 - INCIDENZA DEI CASI DI COVID-19 (PER 100.000 AB) DIAGNOSTICATI IN ITALIA PER REGIONE/PA.
PERIODO: 29 MARZO - 11 APRILE 2021

TABELLA 3 - NUMERO ASSOLUTO E INCIDENZA (PER 100.000 AB) DEI CASI DI COVID-19 DIAGNOSTICATI IN ITALIA PER REGIONE/PA DALL'INIZIO DELL'EPIDEMIA (INCIDENZA CUMULATIVA) E NEI PERIODI 5/4-11/4 E 29/3 – 11/4

REGIONE/PA	NUMERO DI CASI TOTALE	INCIDENZA CUMULATIVA (PER 100.000 AB)	N. CASI TRA IL 5/4-11/4	INCIDENZA 7GG (PER 100.000 AB)	N. CASI TRA IL 29/3 – 11/4	INCIDENZA 14GG (PER 100.000 AB)
Abruzzo	68.531	5.296,30	1.457	112,60	3.274	253,03
Basilicata	21.073	3.808,92	1.037	187,44	1.874	338,72
Calabria	51.966	2.743,56	2.447	129,19	5.108	269,68
Campania	341.933	5.986,07	10.776	188,65	23.668	414,35
Emilia-Romagna	352.902	7.905,30	7.197	161,22	17.792	398,56
Friuli-Venezia Giulia	98.453	8.162,14	1.663	137,87	4.516	374,39
Lazio	311.141	5.405,79	8.749	152,01	20.007	347,60
Liguria	94.520	6.198,74	2.335	153,13	5.526	362,40
Lombardia	770.924	7.688,02	15.129	150,87	38.521	384,15
Marche	90.738	5.998,52	2.084	137,77	5.105	337,48
Molise	12.702	4.226,73	192	63,89	391	130,11
Piemonte	329.556	7.644,15	8.700	201,80	21.777	505,12
PA Bolzano	57.963	10.882,13	521	97,81	1.212	227,54
PA Trento	43.618	7.997,07	654	119,91	1.583	290,23
Puglia	213.974	5.412,53	8.769	221,81	21.024	531,81
Sardegna	48.708	3.022,30	1.635	101,45	3.907	242,43
Sicilia	190.959	3.916,87	7.639	156,69	15.247	312,74
Toscana	211.014	5.714,58	7.115	192,69	16.641	450,66
Umbria	53.151	6.108,15	891	102,39	1.918	220,42
Valle d'Aosta	10.130	8.101,80	357	285,52	905	723,80
Veneto	398.661	8.170,73	6.387	130,90	15.520	318,09
**ITALIA**	**3.772.617**	**6.325,49**	**95.734**	**160,52**	**225.516**	**378,12**

- In **Figura 6** è riportato il confronto tra l'incidenza (per 100.000 abitanti) delle ultime due settimane (29 marzo - 11 aprile 2021) e quella osservata nelle due settimane precedenti (15 – 28 marzo 2021). Il verso e il colore della freccia indicano aumenti (arancione) o diminuzione (blu). Le Regioni Basilicata, Calabria, Sardegna, Sicilia, e Valle d'Aosta sono le uniche a <u>non</u> registrare un'inversione di tendenza rispetto alle due settimane precedenti.

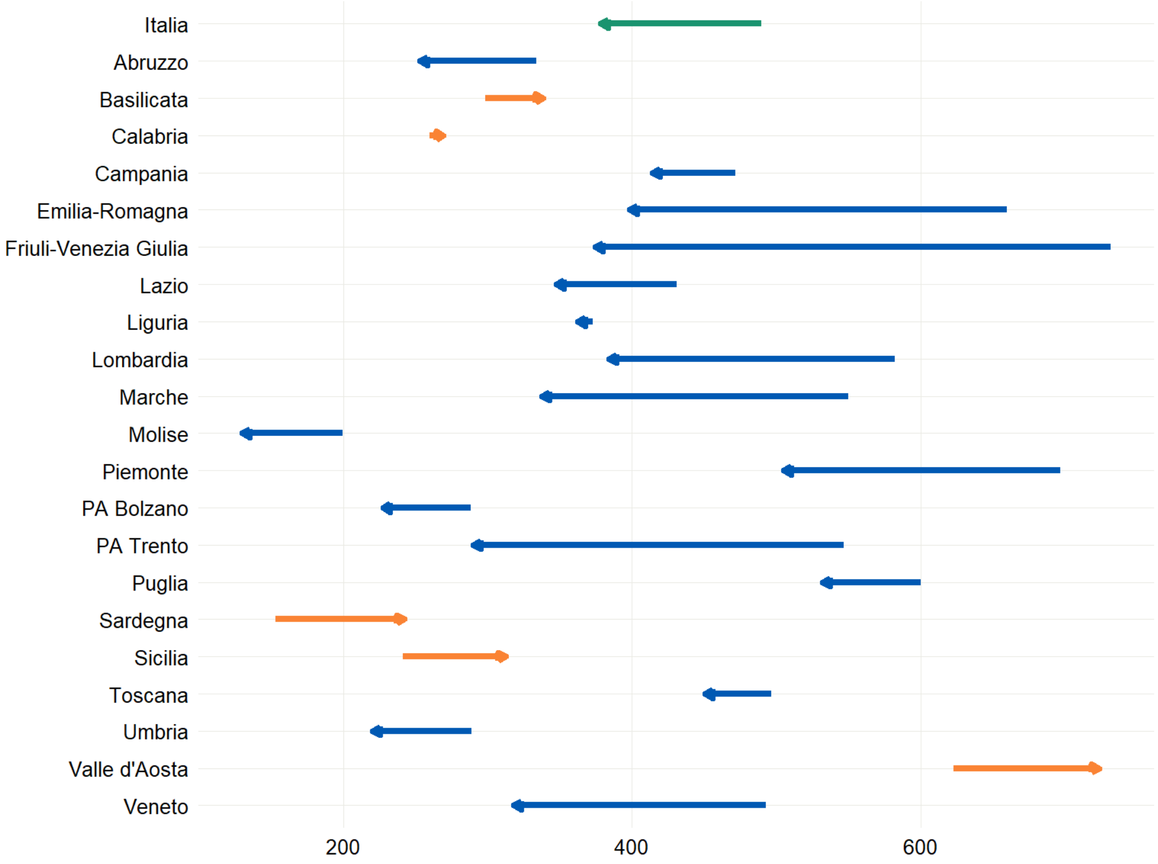

FIGURA 6 – CONFRONTO TRA IL NUMERO CASI DI COVID-19 (PER 100.000 AB) DIAGNOSTICATI IN ITALIA E PER REGIONE NEL PERIODO (29 MARZO - 11 APRILE 2021) E (15 – 28 MARZO 2021)

- In **Figura 7** viene riportata la stima del numero di riproduzione netto Rt medio in un periodo di 14 giorni basato sulla data di inizio sintomi (Rtmedio14gg). Nel periodo 24 marzo – 06 aprile 2021, l'Rt medio calcolato sui casi sintomatici è stato pari a **0,85 (range 0,71– 0,97),** in diminuzione rispetto alla settimana precedente e sotto l'uno anche nel limite superiore. **L'acquisizione dei dati epidemiologici sulle infezioni è affetta da una serie di ritardi, alcuni dei quali non comprimibili: in particolare, il tempo tra l'evento infettivo e lo sviluppo dei sintomi (tempo di incubazione), quello tra i sintomi e l'esecuzione del tampone, quello tra l'esecuzione del tampone e la conferma di positività, e quello tra la conferma di positività e l'inserimento nel sistema di sorveglianza integrata ISS. Il ritardo complessivo tra infezioni e loro rilevamento nel sistema di sorveglianza è valutato e aggiornato settimanalmente. Su queste**

valutazioni si basa la scelta della data più recente alla quale si può considerare sufficientemente stabile la stima di Rt. Per il presente bollettino, ad esempio, si considera il 6 aprile come data ultima per valutare la stima di Rt dei casi sintomatici.

- La **Figura 8** riporta la stima del numero di riproduzione netto Rt medio nazionale a 14 giorni nel tempo in relazione alla curva epidemica.

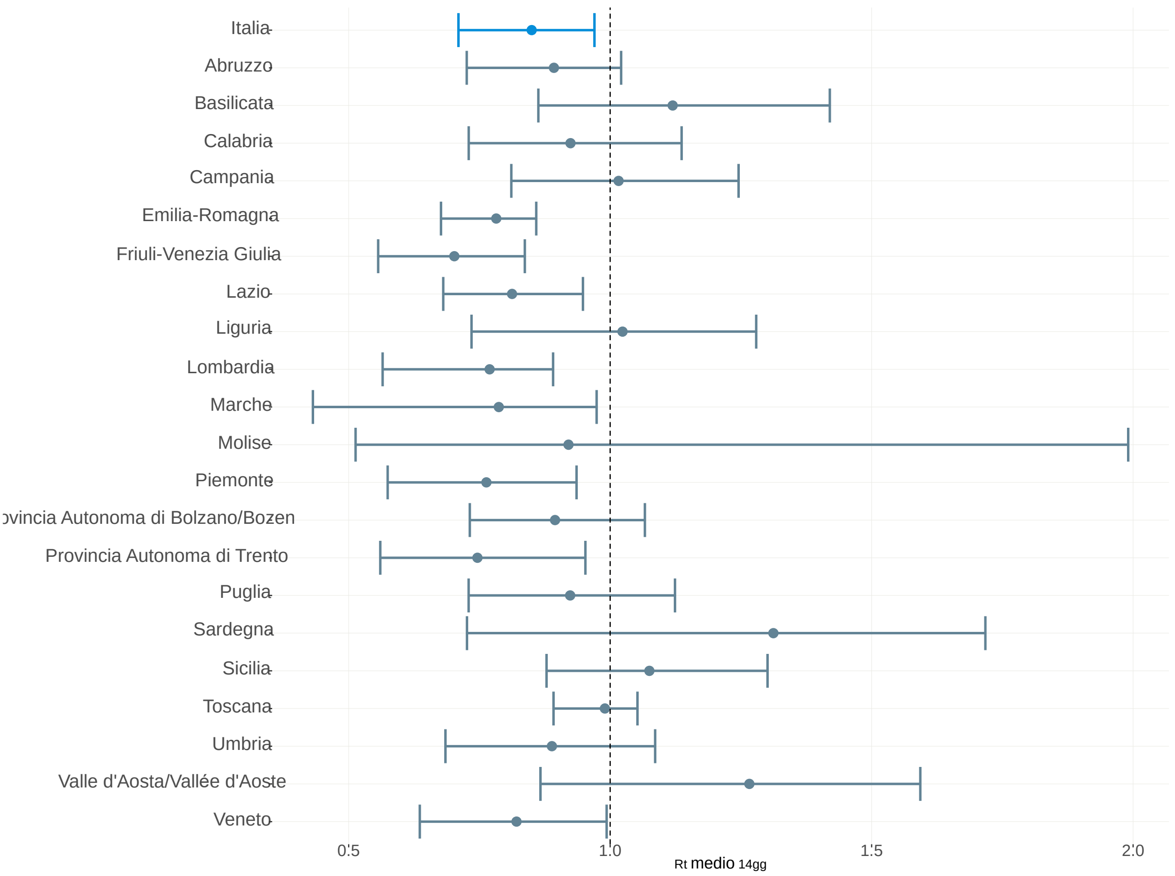

FIGURA 7 - STIMA DELL'RTMEDIO14GG PER REGIONE BASATO SU INIZIO SINTOMI 24 MARZO - 6 APRILE, CALCOLATO IL 14/04/2021

NOTA BENE: Ogni settimana vengono calcolati 3 diversi Rt, Rt puntale (basato sulle date di inizio sintomi), Rt ospedalizzazioni (basato sulle date di ricovero) e Rtmedio14gg (media degli Rt di 14 giorni). In questo report viene riportato solo l'Rtmedio14gg che risente meno di fluttuazioni di breve periodo.

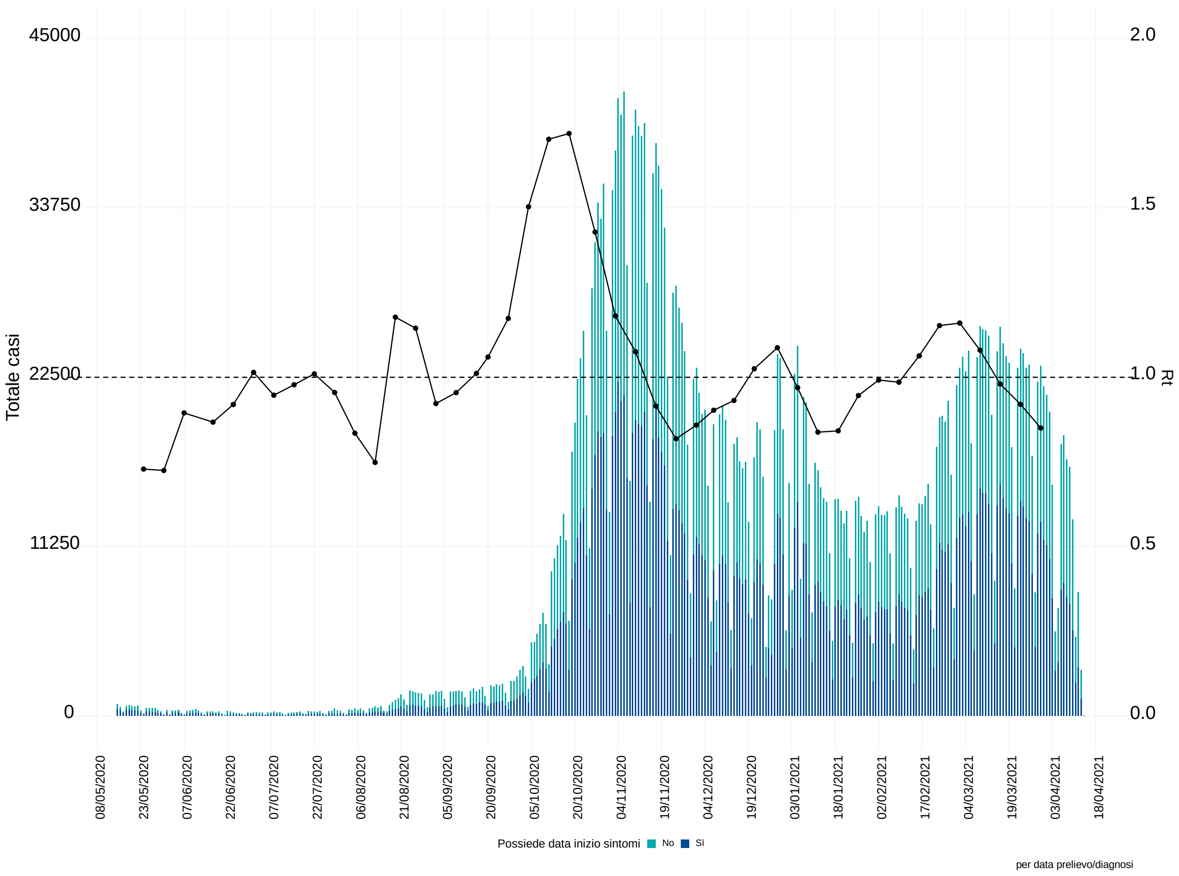

FIGURA 8 - STIME DELL'RTMEDIO14GG E NUMERO DI CASI PER DATA PRELIEVO/DIAGNOSI DISTINTI PER PRESENZA O ASSENZA DELLA DATA DI INIZIO SINTOMI.

Il punto corrispondente a ciascuna stima di Rt è collocato nel giorno centrale dell'intervallo di 14 gg a cui si riferisce

La situazione nazionale dall'inizio dell'epidemia (al 14 aprile 2021)

- Dall'inizio dell'epidemia alle ore 12 del 14 aprile 2021, sono stati riportati al sistema di sorveglianza 3.772.617 casi di COVID-19 diagnosticati in Italia dai laboratori di riferimento regionali come positivi per SARS-CoV–2 (105.041 casi in più rispetto al 7 aprile 2021) e 113.264 decessi (2.705 decessi in più rispetto al 7 aprile 2021).

  La **Figura 9** mostra l'andamento del numero di casi di COVID-19 segnalati in Italia per data di prelievo/diagnosi (disponibile per 3.769.526/3.772.617 casi segnalati). La curva epidemica mostra che l'impatto della seconda ondata epidemica, in termini di numero complessivo di casi giornalieri notificati è decisamente più elevato di quello della prima ondata, grazie all'aumentata capacità diagnostica. Dalla metà di novembre la curva ha mostrato un andamento in lenta ma costante diminuzione fino a 21 dicembre per poi risalire nelle 2 settimane successive. Nel mese di gennaio la curva si è stabilizzata con piccole variazioni giornaliere, ma dal mese di febbraio si è osservato un nuovo aumento dei casi che ha determinato una terza ondata, di minore entità rispetto alla seconda, che nella seconda metà di marzo ha iniziato la decrescita.

- Si ricorda che il numero di casi riportati negli ultimi 7 giorni potrebbe essere sottostimato a causa di un ritardo nella notifica (box grigio).

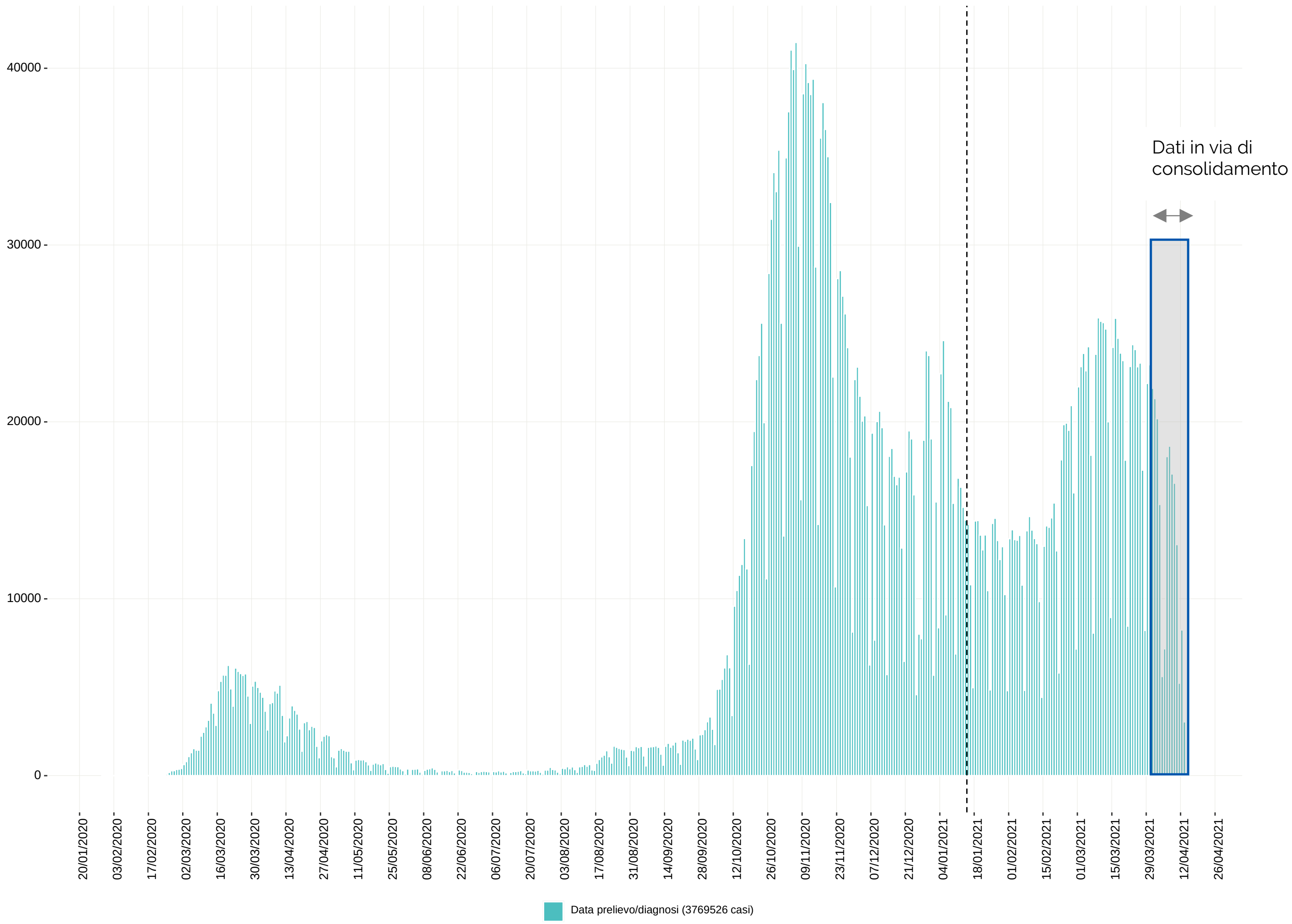

FIGURA 9 – CASI DI COVID-19 DIAGNOSTICATI IN ITALIA PER DATA PRELIEVO/DIAGNOSI (N=3.769.526).
NOTA: I DATI PIÙ RECENTI DEVONO ESSERE CONSIDERATI PROVVISORI (SOPRATTUTTO I DATI NEL RIQUADRO GRIGIO). LA LINEA TRATTEGGIATA NERA INDICA LA DATA DI ADOZIONE DELLA NUOVA DEFINIZIONE DI CASO

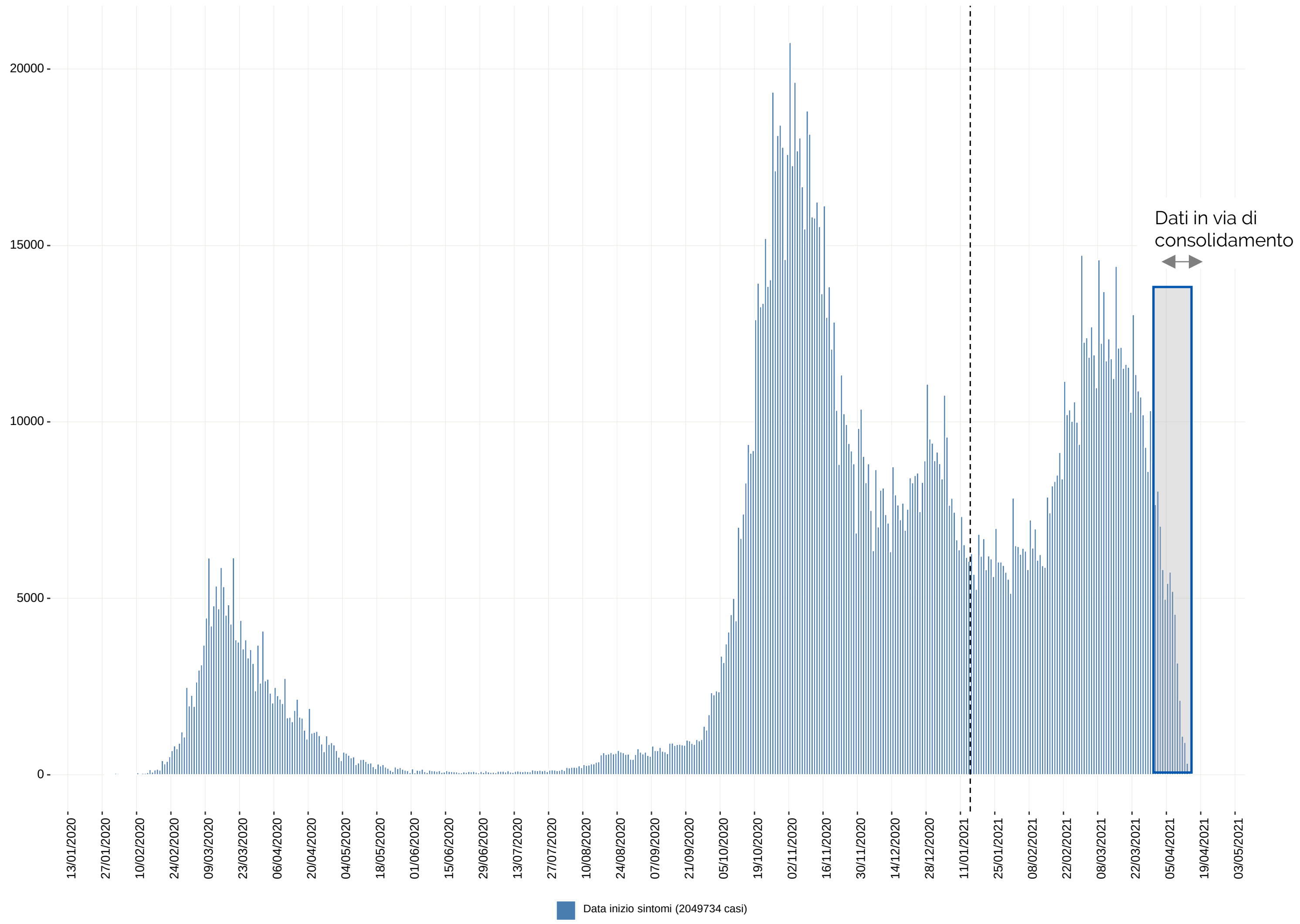

FIGURA 10 – CASI DI COVID-19 DIAGNOSTICATI IN ITALIA PER DATA INIZIO SINTOMI (N=2.049.734)

Nota: i dati più recenti devono essere considerati provvisori sia per il ritardo di notifica sia perché casi non ancora diagnosticati riporteranno in parte la data di inizio sintomi nei giorni del riquadro grigio.

LA LINEA TRATTEGGIATA NERA INDICA LA DATA DI ADOZIONE DELLA NUOVA DEFINIZIONE DI CASO

- La **Figura 10** mostra la distribuzione dei casi per data inizio dei sintomi. La data di inizio sintomi è al momento disponibile per 2.049.734/3.772.617 casi segnalati. Lo scarto tra il numero di casi segnalati e quello di casi per i quali è disponibile la data di inizio dei sintomi è dovuta al fatto che una elevata percentuale dei casi diagnosticati è asintomatica e che per una ridotta percentuale di casi il consolidamento del dato è ancora in corso.

FIGURA 11 – INCIDENZA (PER 100.000 ABITANTI) E NUMERO DI CASI DI COVID-19 DIAGNOSTICATI IN ITALIA NELLA SETTIMANA 5/4-11/4 (N=95.734), PER REGIONE/PA DI DIAGNOSI

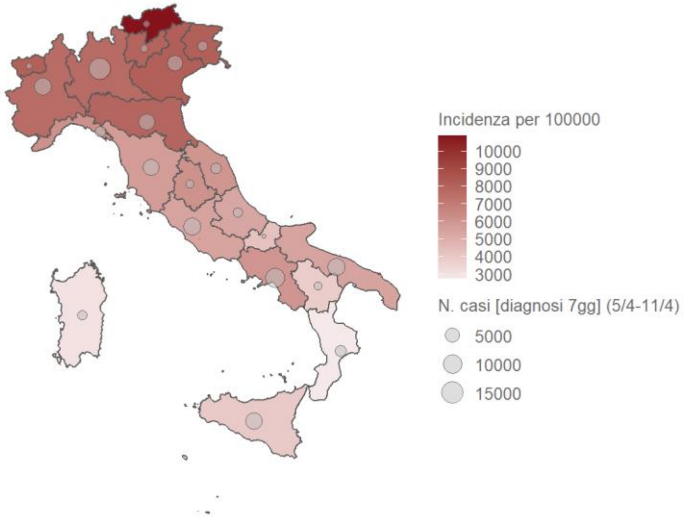

TABELLA 4 - DISTRIBUZIONE DEI CASI DI COVID-19 DIAGNOSTICATI IN ITALIA E INCIDENZA CUMULATIVA PER REGIONE/PA DI DIAGNOSI (N=3.772.617) DALL'INIZIO DELL'EPIDEMIA

Regione/PA	N. Casi	% sul totale	Incidenza cumulativa per 100.000
PA Bolzano	57.963	1,5%	10.882,13
Veneto	398.661	10,6%	8.170,73
Friuli-Venezia Giulia	98.453	2,6%	8.162,14
Valle d'Aosta	10.130	0,3%	8.101,80
PA Trento	43.618	1,2%	7.997,07
Emilia-Romagna	352.902	9,4%	7.905,30
Lombardia	770.924	20,4%	7.688,02
Piemonte	329.556	8,7%	7.644,15
Liguria	94.520	2,5%	6.198,74
Umbria	53.151	1,4%	6.108,15
Marche	90.738	2,4%	5.998,52
Campania	341.933	9,1%	5.986,07
Toscana	211.014	5,6%	5.714,58
Puglia	213.974	5,7%	5.412,53
Lazio	311.141	8,2%	5.405,79
Abruzzo	68.531	1,8%	5.296,30
Molise	12.702	0,3%	4.226,73
Sicilia	190.959	5,1%	3.916,87
Basilicata	21.073	0,6%	3.808,92
Sardegna	48.708	1,3%	3.022,30
Calabria	51.966	1,4%	2.743,56

- La **Figura 11** mostra l'incidenza (per 100.000 abitanti) dei casi confermati di COVID-19 e il numero di casi diagnosticati nella settimana 5/4-11/4 (N=95.734), per Regione/PA di diagnosi.

- La **Tabella 4** riporta in dettaglio il numero dei casi e il tasso di incidenza cumulativa per 100.000 abitanti per Regione/PA. L'83% dei casi è stato diagnosticato in nove regioni: Lombardia, Veneto, Emilia-Romagna, Campania, Piemonte, Lazio, Puglia, Toscana e Sicilia. Nove regioni (Friuli-Venezia Giulia, Liguria, Marche, Abruzzo, PA Bolzano, Umbria, Sardegna, Calabria e PA Trento) hanno riportato tra 40.000 e 100.000 casi; tre regioni/PA (Basilicata, Valle d'Aosta e Molise) hanno riportato meno di 22.000 casi ciascuna. Si sottolinea che, a causa della bassa numerosità della popolazione, la PA di Trento, la PA Bolzano e la regione Valle d'Aosta pur riportando un numero meno consistente di casi presentano una incidenza cumulativa (numero di casi totali segnalati/popolazione residente) particolarmente elevata, con valori simili a quelli riportati dalla Lombardia e dal Veneto.

- L'età mediana dei casi confermati di infezione da SARS-CoV-2 segnalati dall'inizio dell'epidemia è pari a 47 anni (range 0-109 aa). La **Figura 12** mostra l'andamento dell'età mediana per settimana di diagnosi; a partire dalla fine di aprile si osserva un chiaro trend in diminuzione con l'età mediana che passa da oltre 60 anni nei primi due mesi dell'epidemia a circa 30 anni nella settimana centrale di agosto, per poi risalire fino a 49 anni e scendere di nuovo a 45 anni nell'ultima settimana. La **Figura 13** mostra l'età mediana dei casi di Covid-19 al primo ricovero, la **Figura 14** mostra l'età mediana dei casi all'ingresso in terapia intensiva e la **Figura 15** mostra l'età dei casi al momento del decesso.

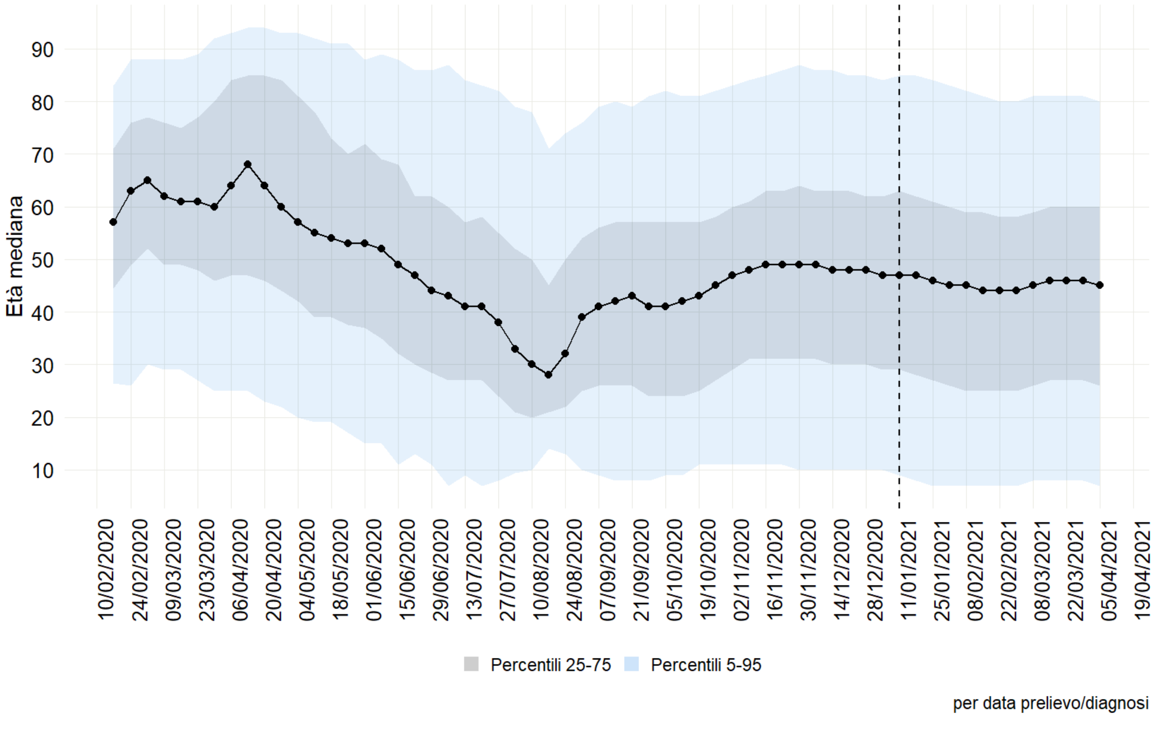

FIGURA 12 – ETÀ MEDIANA DEI CASI DI COVID-19 DIAGNOSTICATI IN ITALIA PER SETTIMANA DI DIAGNOSI

Nota: Ogni punto indica la mediana di ciascuna settimana (lunedì-domenica).

LA LINEA TRATTEGGIATA NERA INDICA LA DATA DI ADOZIONE DELLA NUOVA DEFINIZIONE DI CASO

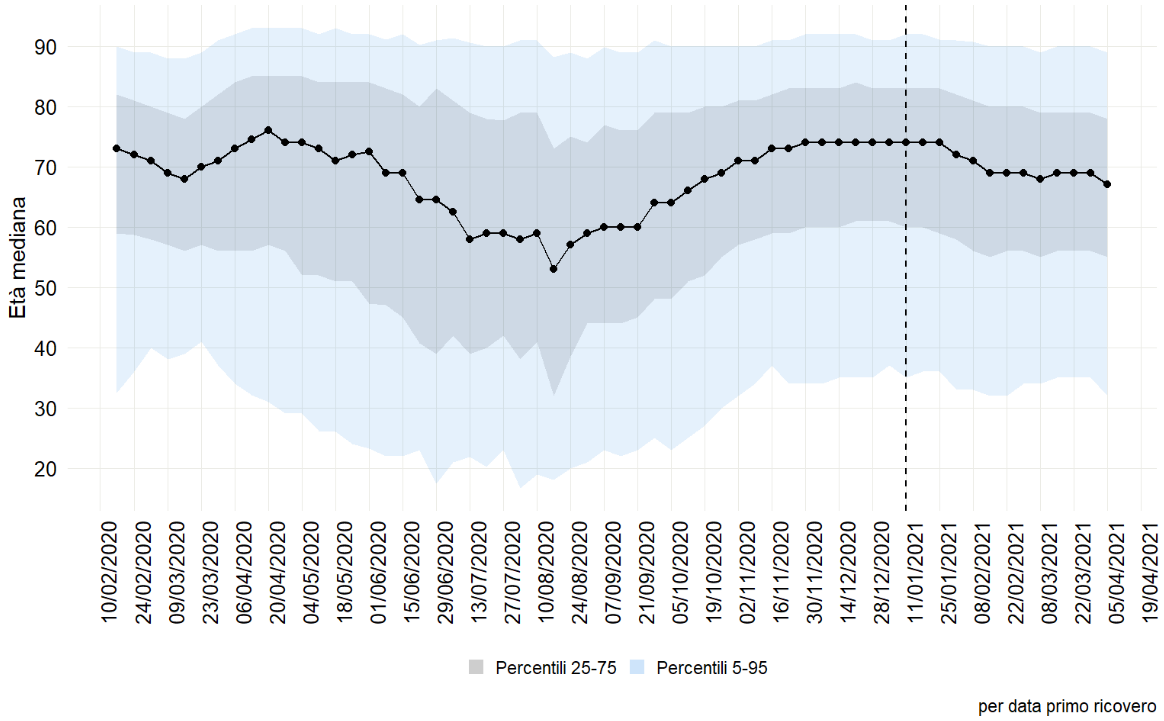

FIGURA 13 – ETÀ MEDIANA DEI CASI DI COVID-19 AL PRIMO RICOVERO IN ITALIA PER SETTIMANA DI DIAGNOSI

Nota: Ogni punto indica la mediana di ciascuna settimana (lunedì-domenica).
LA LINEA TRATTEGGIATA NERA INDICA LA DATA DI ADOZIONE DELLA NUOVA DEFINIZIONE DI CASO

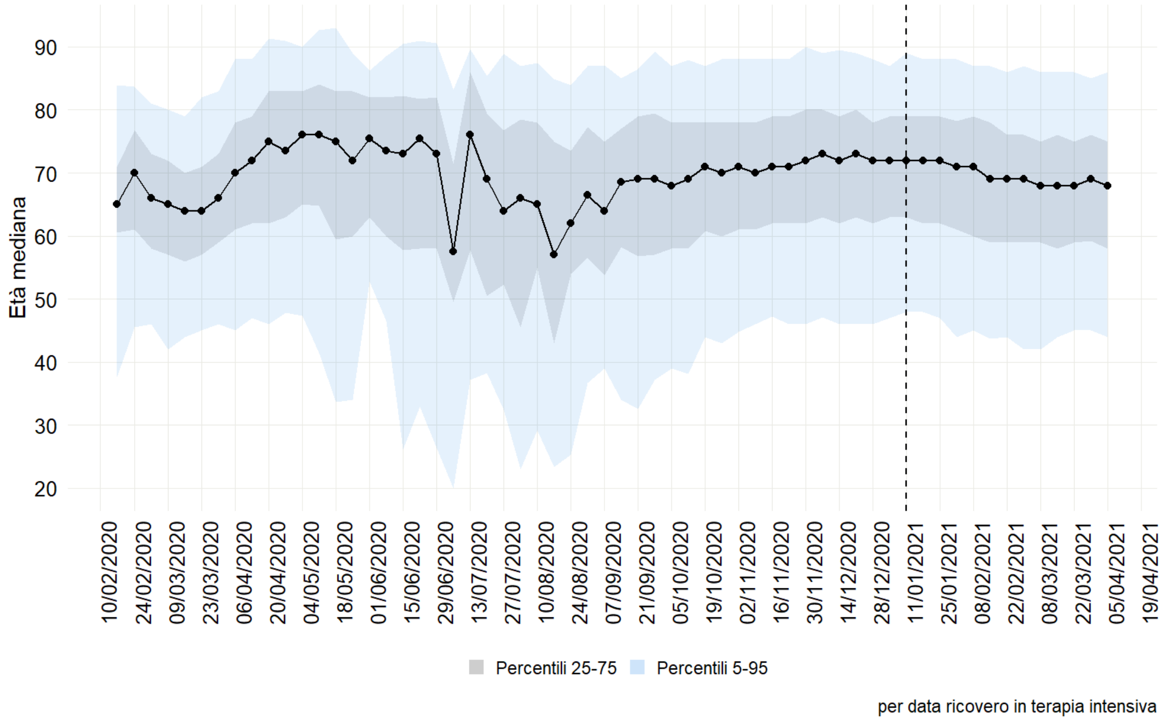

FIGURA 14 – ETÀ MEDIANA DEI CASI DI COVID-19 ALL'INGRESSO IN TERAPIA INTENSIVA IN ITALIA PER SETTIMANA DI DIAGNOSI

Nota: Ogni punto indica la mediana di ciascuna settimana (lunedì-domenica).
LA LINEA TRATTEGGIATA NERA INDICA LA DATA DI ADOZIONE DELLA NUOVA DEFINIZIONE DI CASO

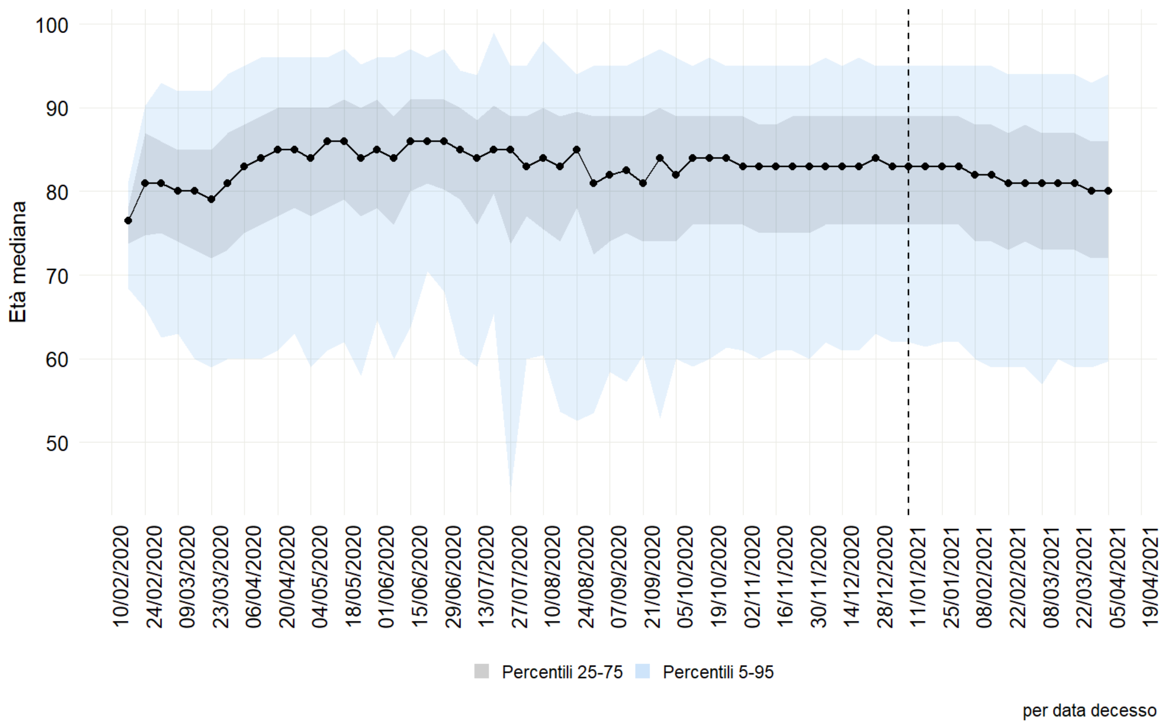

FIGURA 15 – ETÀ MEDIANA DEI CASI DI COVID-19 <u>AL DECESSO</u> IN ITALIA PER SETTIMANA DI DIAGNOSI

Nota: Ogni punto indica la mediana di ciascuna settimana (lunedì-domenica).

LA LINEA TRATTEGGIATA NERA INDICA LA DATA DI ADOZIONE DELLA NUOVA DEFINIZIONE DI CASO

- L'età mediana dei casi confermati di infezione da SARS-CoV-2 al primo ricovero nelle ultime 4 settimane è pari a 67 anni; l'età mediana all'ingresso in terapia intensiva è stabile a 68 anni; l'età mediana al decesso è pari a 80 anni.

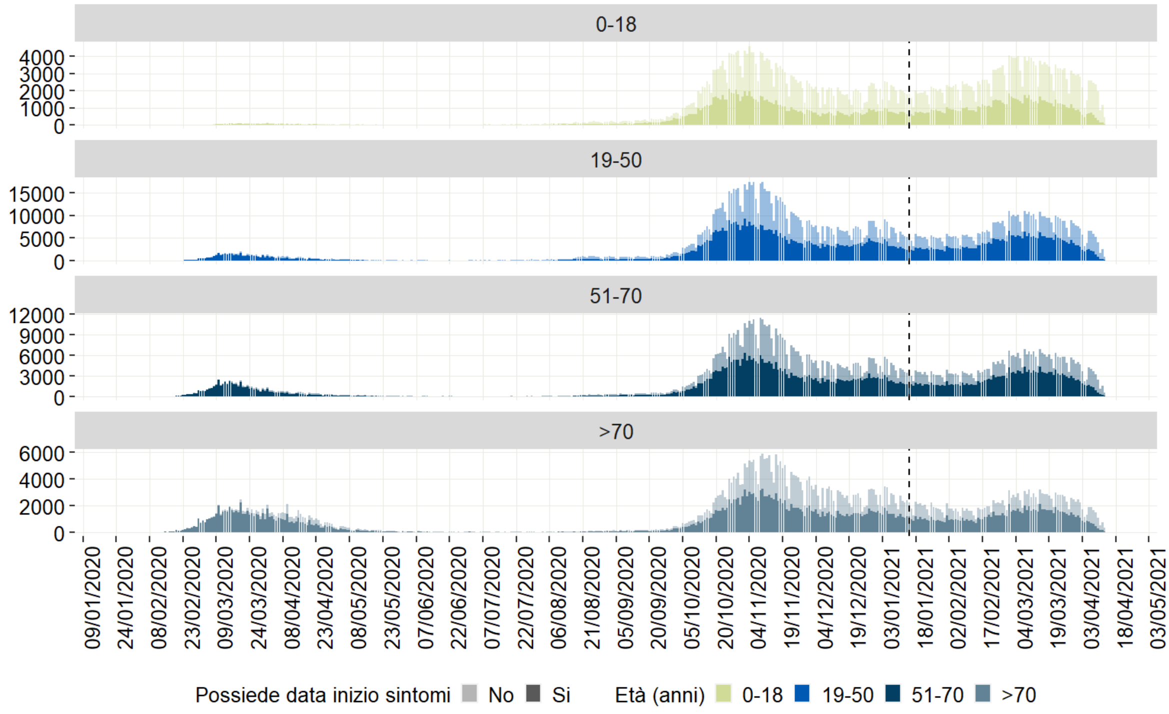

FIGURA 16 – CASI DI COVID-19 DIAGNOSTICATI IN ITALIA PER DATA INIZIO SINTOMI (O PRELIEVO/DIAGNOSI) PER CLASSE DI ETÀ

LA LINEA TRATTEGGIATA NERA INDICA LA DATA DI ADOZIONE DELLA NUOVA DEFINIZIONE DI CASO

- La **Figura 16** mostra l'andamento dei casi (per data inizio sintomi o data prelievo/diagnosi se non disponibile la data inizio sintomi) per classe di età. Dopo il picco della seconda ondata, verificatosi intorno alla metà di novembre, si è osservato un decremento in tutte le fasce di età e un successivo picco di minore entità nella terza decade di dicembre, seguito da un decremento nel mese di gennaio e poi da un nuovo incremento in tutte le fasce d'età. Dalla seconda metà di marzo si sta osservando un nuovo lento decremento della curva.

- La **Figura 17** mostra la percentuale di casi per sesso nel tempo. Complessivamente la percentuale tra i maschi e le femmine è molto simile (51,4% per le femmine), anche se nella fase iniziale dell'epidemia il numero di casi diagnosticato in persone di sesso maschile era leggermente superiore.

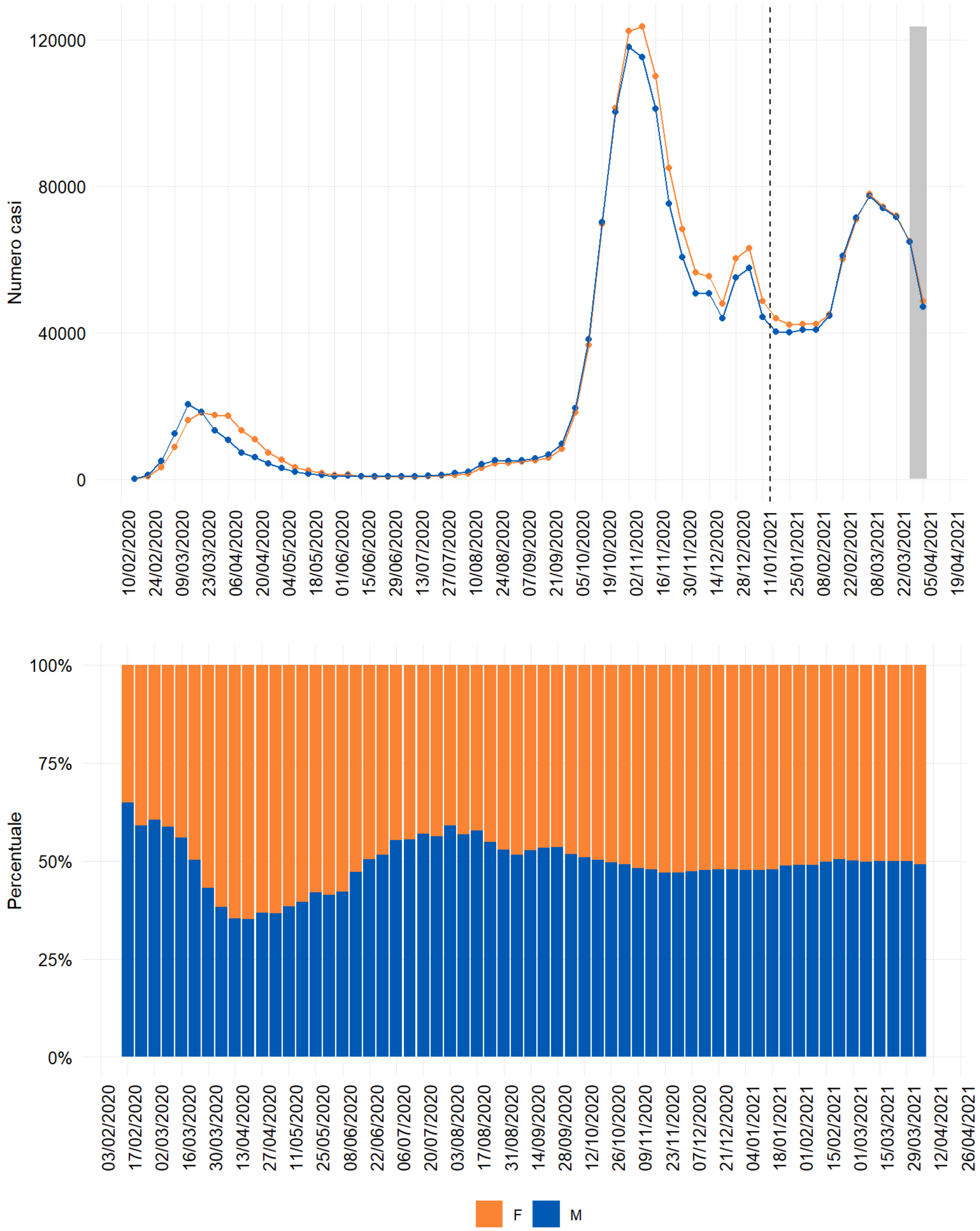

FIGURA 17 – NUMERO E PERCENTUALE DI CASI DI COVID-19 DIAGNOSTICATI IN ITALIA PER SESSO E SETTIMANA DI DIAGNOSI

Nota: Ogni punto e ogni barra indicano il numero e la percentuale di casi in ciascuna settimana (lunedi-domenica).

LA LINEA TRATTEGGIATA NERA INDICA LA DATA DI ADOZIONE DELLA NUOVA DEFINIZIONE DI CASO

- La **Figura 18** mostra il cambiamento nel tempo del quadro clinico riportato al momento della diagnosi dei casi confermati di COVID-19. Mentre nelle prime settimane dell'epidemia si riscontrava una maggiore percentuale di casi severi,

critici e di casi già deceduti al momento della diagnosi (diagnosticati mediante tamponi effettuali *post-mortem*), con il passare del tempo, si evidenzia, in percentuale, un netto incremento dei casi asintomatici o pauci-sintomatici e una marcata riduzione dei casi severi e dei decessi specialmente nelle fasce d'età 0-19, 20-59 e 60-69.

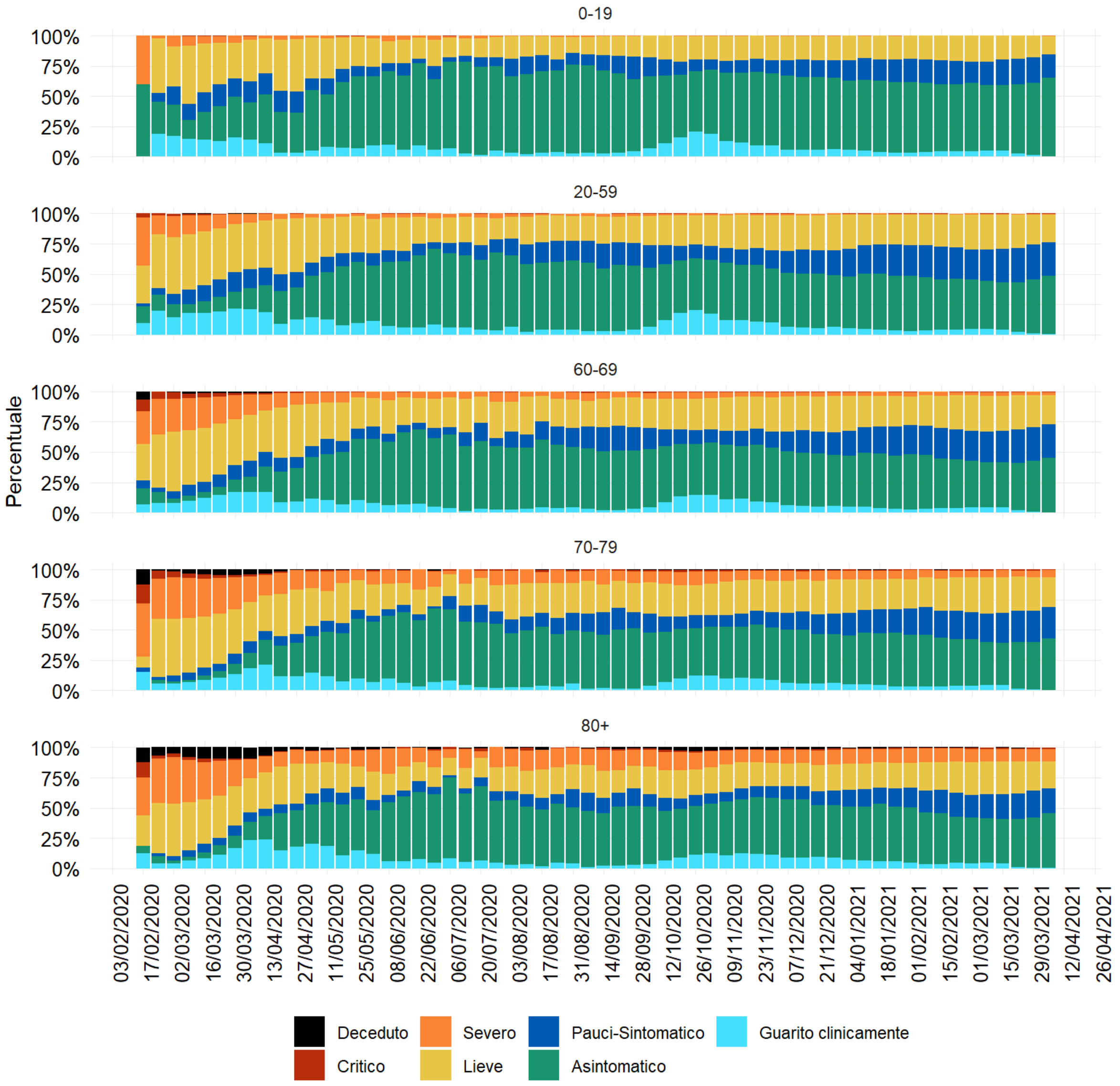

FIGURA 18 – PERCENTUALE DI CASI DI COVID-19 DIAGNOSTICATI IN ITALIA PER STATO CLINICO AL MOMENTO DELLA DIAGNOSI, PER CLASSE D'ETÀ E SETTIMANA DI DIAGNOSI

- La **Tabella 5** mostra la distribuzione dei casi e dei decessi segnalati per sesso e fasce di età decennali. L'informazione sul sesso ed età è nota per il 99,9% dei casi segnalati; 1.931.154 casi sono di sesso femminile (51,2%). Nelle fasce di età 0-9, 10-19, 20-29, 50-59, 60-69 e 70-79 anni si osserva un numero maggiore di casi di sesso maschile rispetto a quello di casi di sesso femminile. Inoltre, la tabella riporta il numero dei casi e la letalità per fascia di età e sesso. Si osserva un aumento della letalità con l'aumentare dell'età dei pazienti; inoltre, a partire dalla fascia di età 30-39 anni la letalità è più elevata nei soggetti di sesso maschile.

TABELLA 5 - DISTRIBUZIONE DEI CASI (N=3.772.617) E DEI DECESSI (N=113.264) PER COVID-19 DIAGNOSTICATI IN ITALIA PER FASCIA DI ETÀ E SESSO

Classe di età (anni)	Soggetti di sesso maschile					Soggetti di sesso femminile					Casi totali				
	N. casi	% casi totali	N. deceduti	% del totale deceduti	Letalità %	N. casi	% casi totali	N. deceduti	% del totale deceduti	Letalità %	N. casi	% casi per classe di età	N. deceduti	% deceduti per classe di età	Letalità %
0-9	97.371	51,8	4	40,0	0	90.464	48,2	6	60,0	0	187.835	5,0	10	0	0
10-19	181.695	52,1	7	58,3	0	167.253	47,9	5	41,7	0	348.948	9,2	12	0	0
20-29	226.352	50,7	33	60,0	0	220.121	49,3	22	40,0	0	446.477	11,8	55	0	0
30-39	228.142	48,9	133	63,3	0,1	238.619	51,1	77	36,7	0	466.765	12,4	210	0,2	0
40-49	287.344	47,5	675	70,2	0,2	317.221	52,5	287	29,8	0,1	604.566	16,0	962	0,8	0,2
50-59	324.493	48,9	2.731	72,5	0,8	338.450	51,1	1.037	27,5	0,3	662.945	17,6	3.768	3,3	0,6
60-69	219.247	52,1	8.060	73,2	3,7	201.297	47,9	2.956	26,8	1,5	420.545	11,1	11.016	9,7	2,6
70-79	158.006	51,0	18.921	67,9	12,0	151.982	49,0	8.942	32,1	5,9	309.988	8,2	27.863	24,6	9,0
80-89	98.569	41,0	25.446	54,4	25,8	141.995	59,0	21.297	45,6	15,0	240.571	6,4	46.743	41,3	19,4
≥90	20.170	24,1	7.735	34,2	38,3	63.696	75,9	14.883	65,8	23,4	83.866	2,2	22.618	20,0	27,0
Età non nota	55	49,5	6	85,7	10,9	56	50,5	1	14,3	1,8	111	-	7	-	6,3
**Totale**	**1.841.444**	**48,8**	**63.751**	**56,3**	**3,5**	**1.931.154**	**51,2**	**49.513**	**43,7**	**2,6**	**3.772.617**	**-**	**113.264**	**-**	**3,0**

NOTA: LA TABELLA NON INCLUDE I CASI PER CUI NON SONO NOTI IL SESSO E L'ETÀ (TABELLE PER SESSO) O L'ETÀ (TABELLA TOTALE)

- La **Tabella 6** riporta la distribuzione dei casi e dei decessi segnalati per classe di età tra la popolazione 0-19 anni.

TABELLA 6 - DISTRIBUZIONE DEI CASI (N=534.320) E DEI DECESSI (N=22) DIAGNOSTICATI NELLA POPOLAZIONE 0-19 ANNI PER FASCIA DI ETÀ IN ITALIA

Classe di età (anni)	N. casi	N. deceduti
<3	40.391	4
3-5	50.808	3
6-10	125.577	3
11-13	98.955	5
14-19	218.589	7
**Totale**	**534.320**	**22**

- La **Figura 19** mostra, dall'alto verso il basso, la distribuzione dei casi per data di inizio sintomi, data di prelievo/diagnosi, data di ricovero e data di decesso. L'andamento delle curve è simile nelle quattro figure ma il raggiungimento del picco si sposta nel tempo.

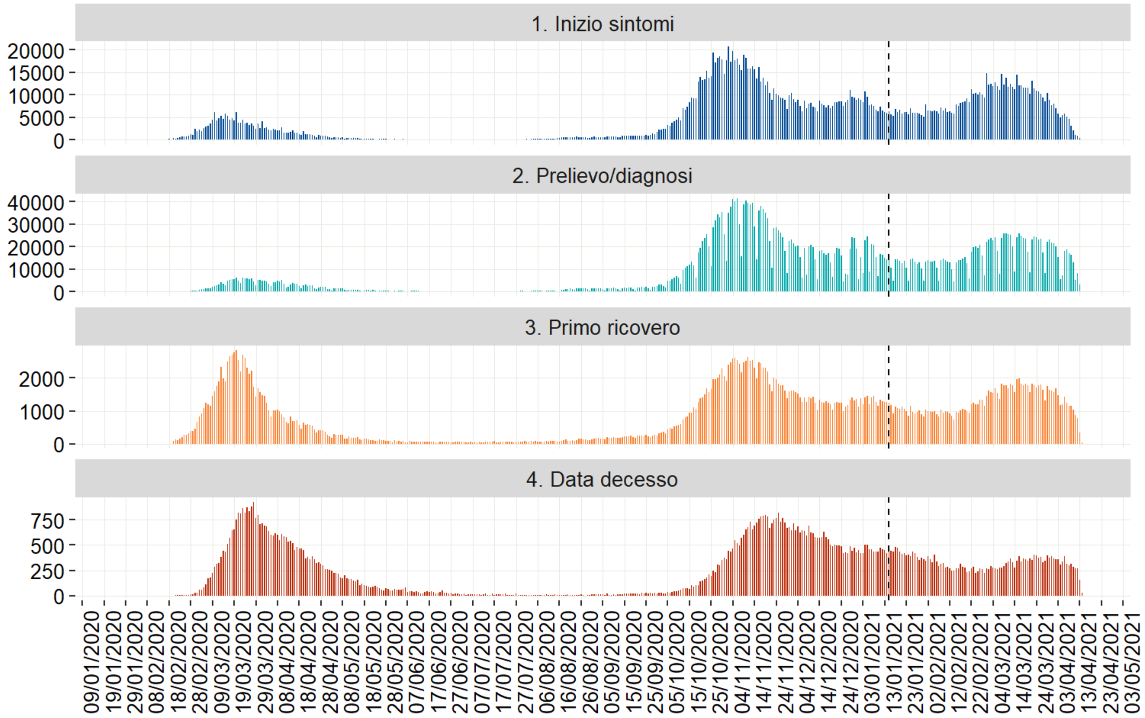

FIGURA 19 – CONFRONTO TRA I CASI DI COVID-19 DIAGNOSTICATI IN ITALIA PER DATA DI INIZIO SINTOMI, DATA DI PRELIEVO/DIAGNOSI, DATA DI RICOVERO E DATA DI DECESSO
LA LINEA TRATTEGGIATA NERA INDICA LA DATA DI ADOZIONE DELLA NUOVA DEFINIZIONE DI CASO

- Al 14 aprile 2021, risultano guariti 2.886.069 casi. Escludendo dal totale dei casi segnalati (3.772.617) i casi guariti, i casi deceduti per Covid-19 e gli altri decessi non legati a COVID-19 (113.264 e 573 rispettivamente) e 4.135 casi persi al follow-up, l'informazione sulla gravità clinica dei pazienti affetti da COVID-19 è disponibile per 725.960/768.576 casi confermati (94,1%). Tra questi, 413.626 (57,0%) risultano asintomatici, 123.633 (17,0%) sono

pauci-sintomatici, 151.607 (20,9%) hanno sintomi lievi, 31.759 (4,4%) hanno sintomi severi e 5.335 (0,7%) presentano un quadro clinico critico.

- Escludendo i casi che risultano guariti, deceduti e persi al follow-up, l'informazione sulla collocazione del paziente è disponibile per 614.691/768.576 casi (79,7% del totale); in particolare, 581.808 (94,6%) stanno affrontando l'infezione presso il proprio domicilio o in altra struttura, 296 casi (<0,1%) si trovano su una Nave Quarantena, 29 (<0,1%) sono ricoverati presso il Policlinico Militare del Celio e 32.558 (5,3%) sono ospedalizzati. **Si sottolinea che i dati relativi allo stato clinico e alla collocazione del paziente sono dati soggetti a modifiche a causa dell'evoluzione dello stato clinico dei pazienti e al loro conseguente ricovero o dimissione. L'aggiornamento di queste variabili nel database della Sorveglianza Integrata Nazionale coordinata dall'ISS che, si ricorda, contiene dati individuali richiede tempo, e di conseguenza il dato può risultare leggermente disallineato da quello fornito dal flusso di dati aggregati coordinato dal Ministero della Salute.**

- La **Figura 20** mostra l'andamento dei dati aggregati, riportati dal Ministero della Salute al 14 aprile 2021, per condizione di ricovero, isolamento domiciliare ed esito dei casi confermati di COVID-19**.** La **Figura 21** mostra la distribuzione di casi diagnosticati quotidianamente per condizione di isolamento domiciliare e ricovero.

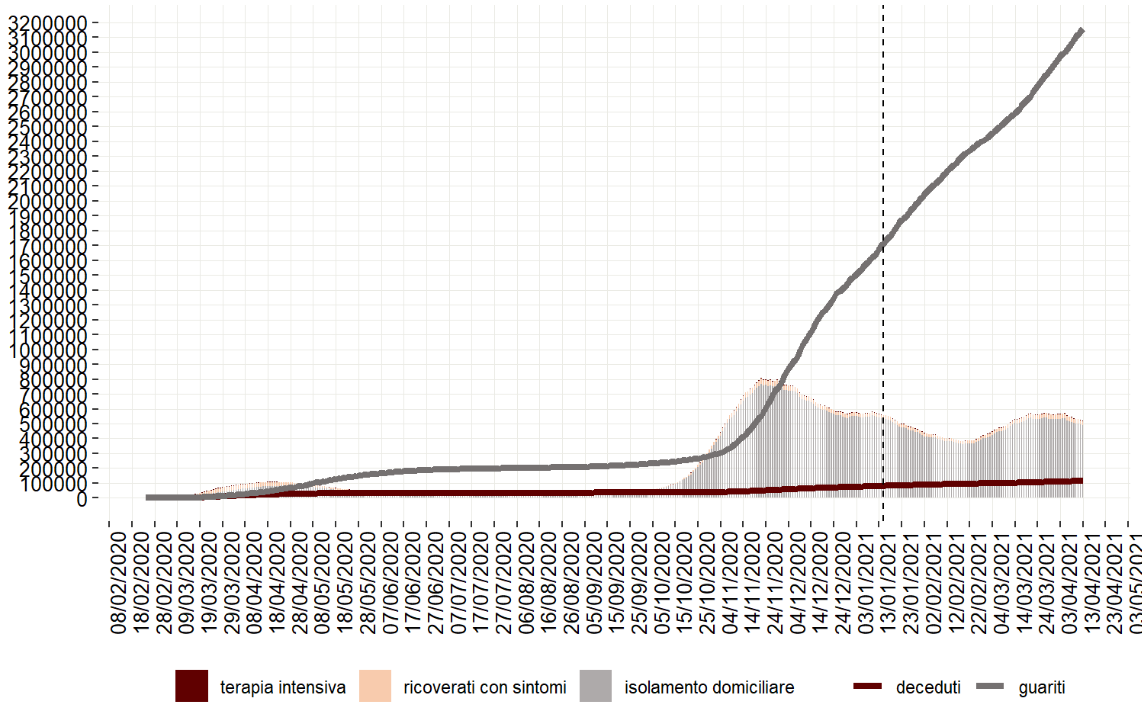

FIGURA 20 – NUMERO TOTALE DI CASI DI COVID-19 (ESCLUSI GUARITI E DECEDUTI) DIAGNOSTICATI IN ITALIA PER STATO DI RICOVERO/ISOLAMENTO E NUMERO CUMULATIVO DELL'ESITO (N=3.809.193) AL 14/4/2021 (FONTE DATI MINISTERO DELLA SALUTE E PROTEZIONE CIVILE).

LA LINEA TRATTEGGIATA NERA INDICA LA DATA DI ADOZIONE DELLA NUOVA DEFINIZIONE DI CASO

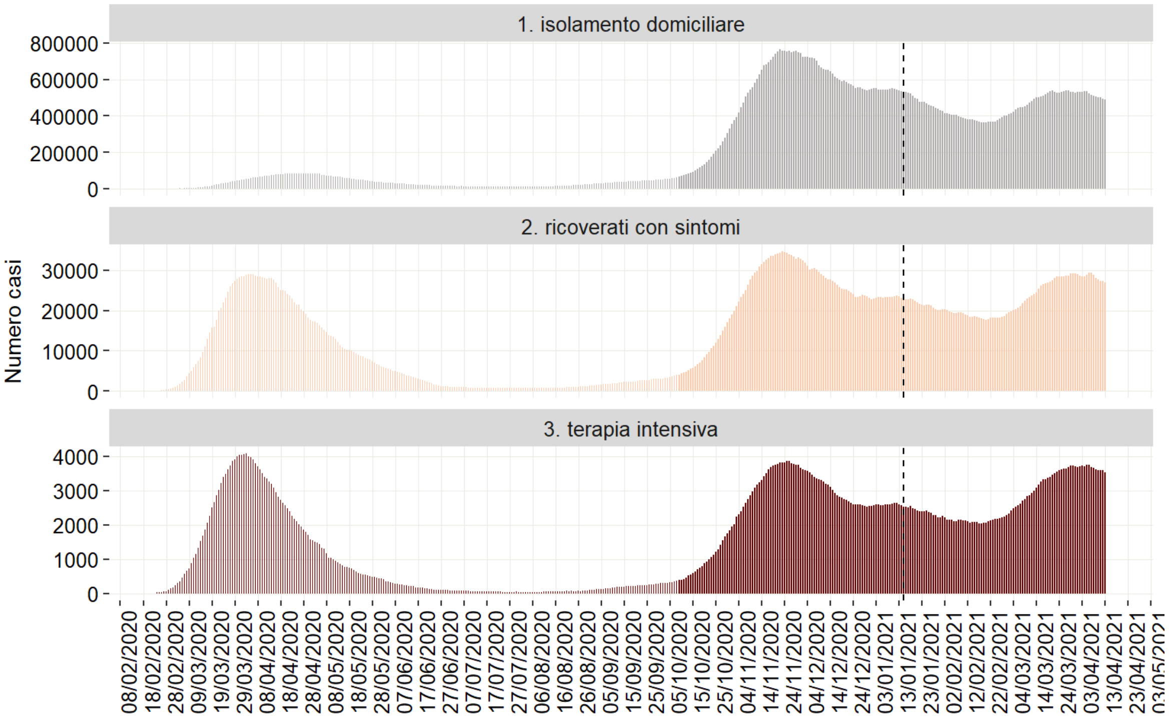

FIGURA 21 – NUMERO DI CASI DI COVID-19 DIAGNOSTICATI IN ITALIA QUOTIDIANAMENTE PER STATO DI ISOLAMENTO/RICOVERO (FONTE DATI MINISTERO DELLA SALUTE E PROTEZIONE CIVILE)

LA LINEA TRATTEGGIATA NERA INDICA LA DATA DI ADOZIONE DELLA NUOVA DEFINIZIONE DI CASO

- Dall'inizio dell'epidemia sono stati diagnosticati 130.849 casi tra gli operatori sanitari (età mediana 47 anni) pari al 3% dei casi totali segnalati. La **Tabella 7** riporta la distribuzione dei casi segnalati per classe di età e sesso e la letalità osservata in questa popolazione.

- I dati riportati dalle Regioni/PPAA indicano che la letalità tra gli operatori sanitari è inferiore, anche a parità di classe di età (**Tabella 7**), alla letalità totale (vedi **Tabella 5**), verosimilmente perché gli operatori sanitari asintomatici e pauci-sintomatici vengono maggiormente testati rispetto alla popolazione generale.

- La **Figura 22** riporta la proporzione di casi tra operatori sanitari sul totale dei casi segnalati in Italia per periodo di diagnosi (ogni 7 giorni). La proporzione è stata calcolata solo sui casi per i quali l'informazione è nota. A metà novembre la percentuale dei casi tra gli operatori sanitari ha superato il 5% del totale, ma dalla metà di gennaio si osserva un trend in diminuzione verosimilmente attribuibile al completamento del ciclo vaccinale in una buona percentuale di soggetti appartenenti a questa categoria.

TABELLA 7 - DISTRIBUZIONE DEI CASI (N=130.849) E DEI DECESSI (N=315) PER COVID-19 DIAGNOSTICATI NEGLI OPERATORI SANITARI IN ITALIA PER FASCIA DI ETÀ E SESSO

Classe di età (anni)	Soggetti di sesso maschile					Soggetti di sesso femminile					Casi totali				
	N. casi	% casi totali	N. deceduti	% del totale deceduti	Letalità %	N. casi	% casi totali	N. deceduti	% del totale deceduti	Letalità %	N. casi	% casi per classe di età	N. deceduti	% deceduti per classe di età	Letalità %
18-29	4.864	12,5	0	0	0%	11.711	12,7	0	0	0%	16.575	12,7	0	0	0%
30-39	8326	21,4	1	0,5	0%	16.876	18,4	2	2,1	0%	25.202	19,3	3	1	0%
40-49	8473	21,8	8	3,7	0,10%	26.524	28,9	8	8,3	0%	34.997	26,7	16	5,1	0%
50-59	10246	26,3	39	17,8	0,40%	29.286	31,9	28	29,2	0,10%	39.532	30,2	67	21,3	0,20%
60-69	6367	16,4	111	50,7	1,70%	7.035	7,7	23	24	0,30%	13.402	10,2	134	42,5	1%
70-79	439	1,1	34	15,5	7,70%	219	0,2	9	9,4	4,10%	658	0,5	43	13,7	6,50%
Età non nota	215	0,6	26	11,9	12,10%	268	0,3	26	27,1	9,70%	483	0,4	52	16,5	10,80%
**Totale**	**38.930**	**29,8**	**219**	**69,5**	**0,60%**	**91.919**	**70,2**	**96**	**30,5**	**0,10%**	**130.849**	**-**	**315**	**-**	**0,20%**

NOTA: LA TABELLA NON INCLUDE I CASI PER CUI NON È NOTO IL SESSO

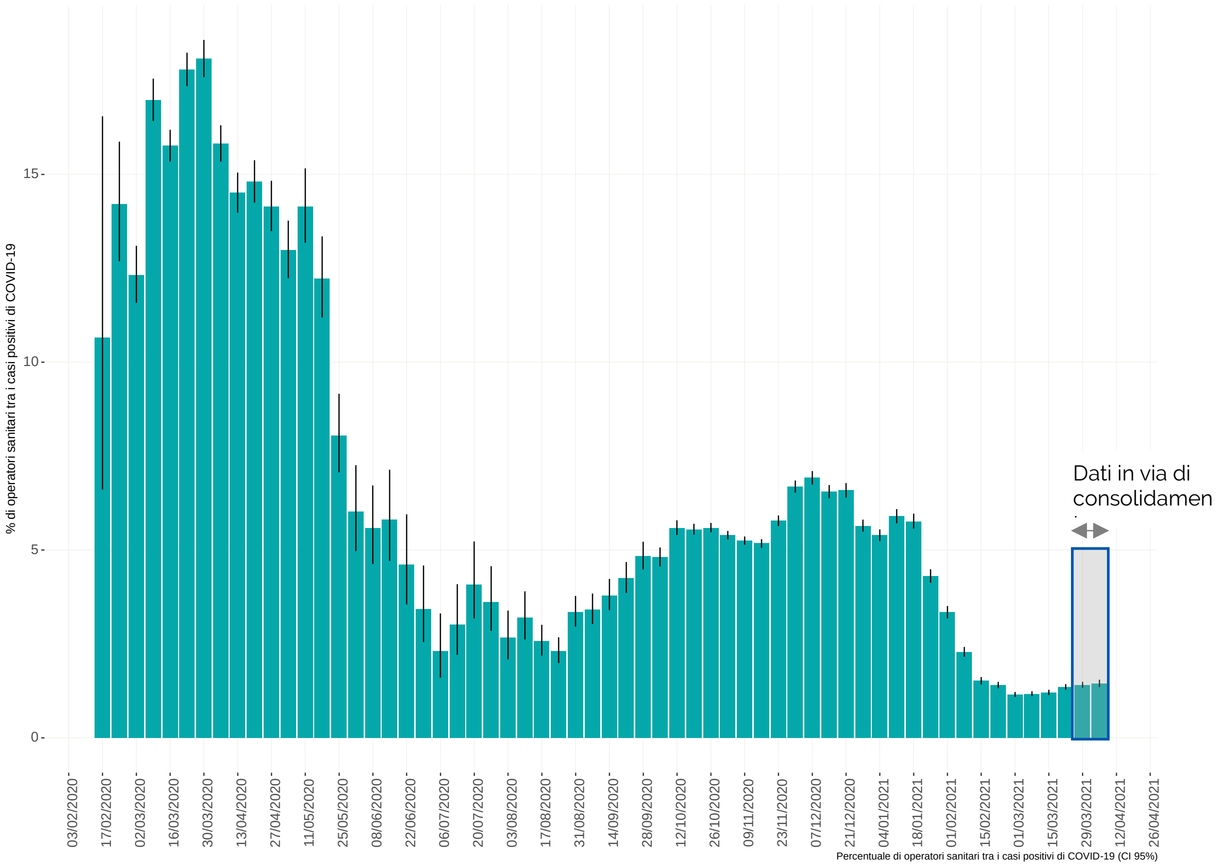

FIGURA 22 – PERCENTUALE DI OPERATORI SANITARI RIPORTATI SUL TOTALE DEI CASI DIAGNOSTICATI IN ITALIA PER PERIODO DI DIAGNOSI (7 GIORNI)

NOTA: OGNI BARRA FA RIFERIMENTO ALL'INTERVALLO DI TEMPO TRA LA DATA INDICATA SOTTO LA BARRA E QUELLA SUCCESSIVA (ESEMPIO: 17 FEB SI RIFERISCE AL PERIODO DAL 19-23 FEB, 24 FEB SI RIFERISCE AL PERIODO DAL 24-30 FEB, ETC.)

Focus: Incidenza per fascia d'età nazionale e per Regione/PA

- La **Figura 23** riporta il tasso d'incidenza per fascia d'età a livello nazionale a partire dal 10 agosto 2020 (inizio della seconda ondata dell'epidemia). La fascia di età >90 anni è quella con l'incidenza maggiore, pari a 8.138,66 per 100.000 abitanti, mentre l'incidenza minore si rileva nella fascia 0-9 anni (3.763,68 per 100.000 abitanti). Il picco di incidenza viene raggiunto nella settimana del 8/3/2021 per la fascia d'età 0-9, del 26/10 per la fascia di età 10-19 anni, del 2/11 per le fasce d'età, 30-39, 40-49 e 50-59 e nella settimana del 9/11 per tutte le fasce di età oltre i 60 anni. Dalla settimana del 9/11 si osserva un decremento fino al 21 dicembre, seguito da un successivo incremento in tutte le fasce d'età fino alla seconda settimana di marzo quando inizia un nuovo decremento.

- La **Figura 24** riporta il tasso d'incidenza nazionale per fascia d'età per la popolazione in età scolare (0-19 anni) a partire dal 24 agosto 2020. All'inizio di gennaio si è osservato un incremento dell'incidenza nella popolazione di età 0-19 anni durato fino all'inizio di marzo quando l'incidenza inizia a diminuire in tutte le fasce d'età.

Prodotto dall'Istituto Superiore di Sanità (ISS), Roma, 14 aprile 2021

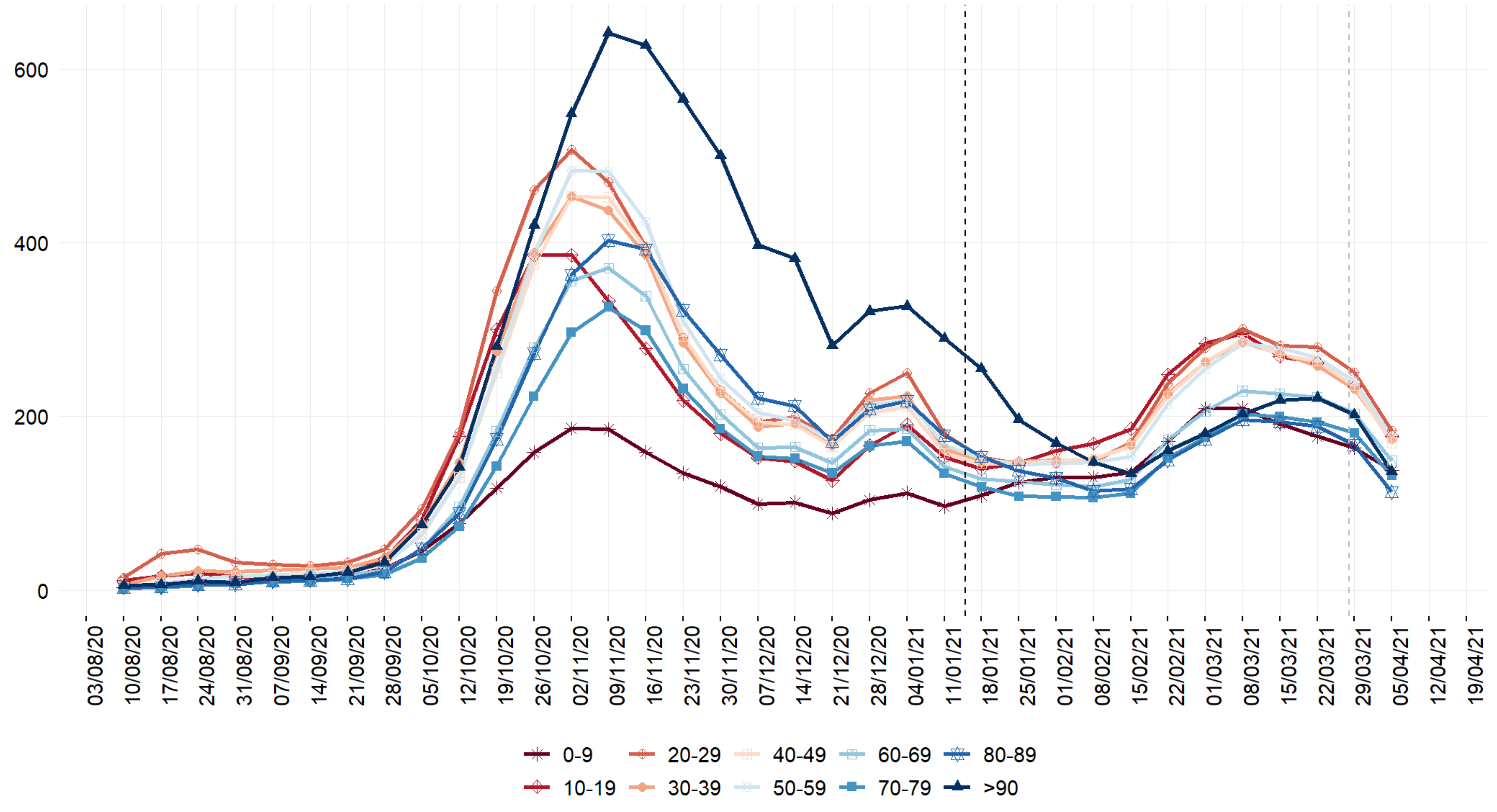

FIGURA 23 – INCIDENZA NAZIONALE PER FASCIA D'ETÀ

Nota: Oltre la linea tratteggiata grigia il dato deve essere considerato provvisorio.

LA LINEA TRATTEGGIATA NERA INDICA LA DATA DI ADOZIONE DELLA NUOVA DEFINIZIONE DI CASO

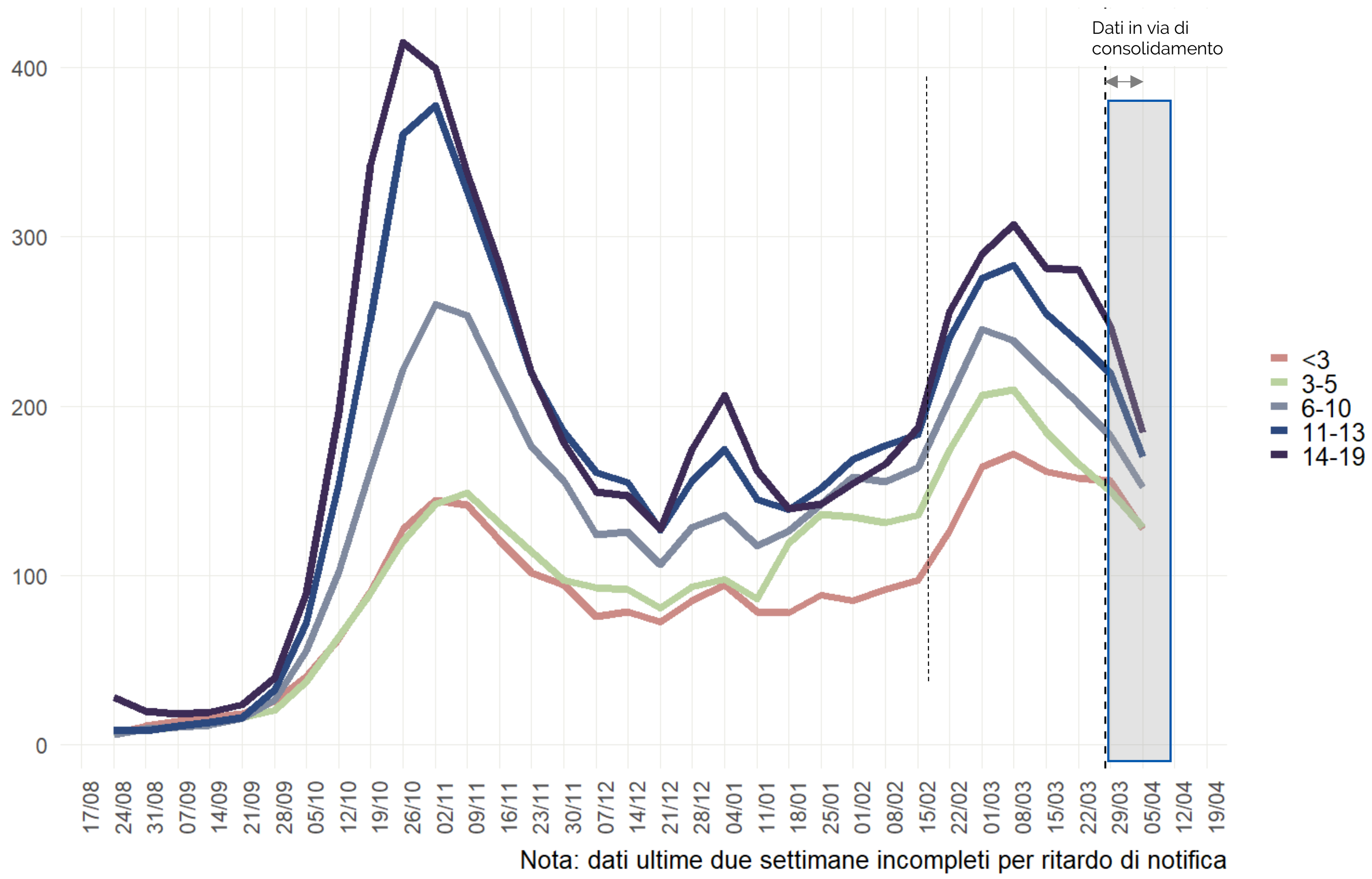

FIGURA 24 – INCIDENZA PER FASCIA D'ETÀ – POPOLAZIONE 0-19 ANNI

All'interno dell'area grigia il dato deve essere considerato provvisorio **LA LINEA TRATTEGGIATA NERA INDICA LA DATA DI ADOZIONE DELLA NUOVA DEFINIZIONE DI CASO**

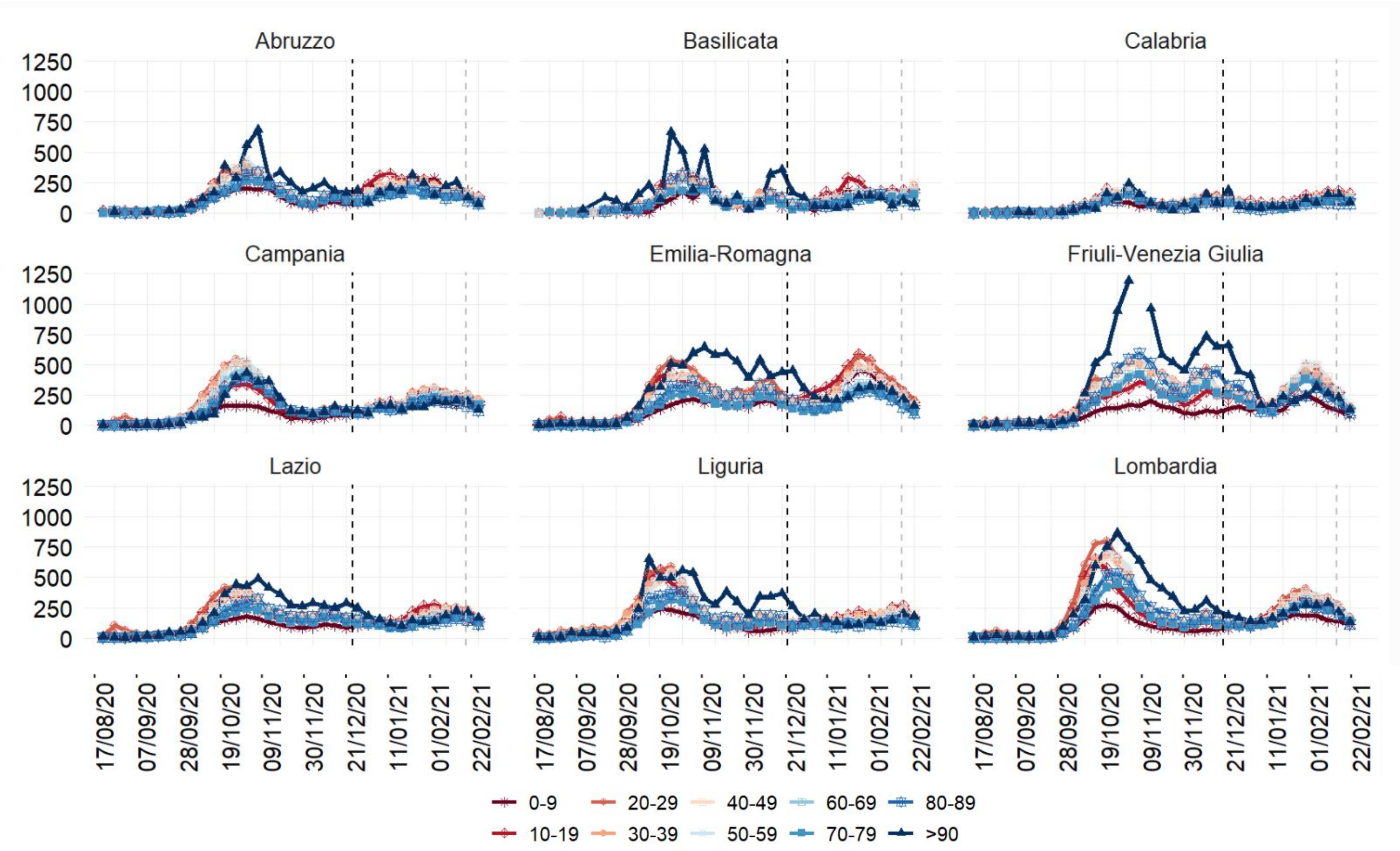

FIGURA 25A – INCIDENZA PER FASCIA D'ETÀ PER REGIONE/PA

Nota: Per una migliore visualizzazione l'asse delle ordinate è stato troncato a 1.250 casi (per 100.000 abitanti). Oltre la linea tratteggiata grigia il dato deve essere considerato provvisorio.

LA LINEA TRATTEGGIATA NERA INDICA LA DATA DI ADOZIONE DELLA NUOVA DEFINIZIONE DI CASO

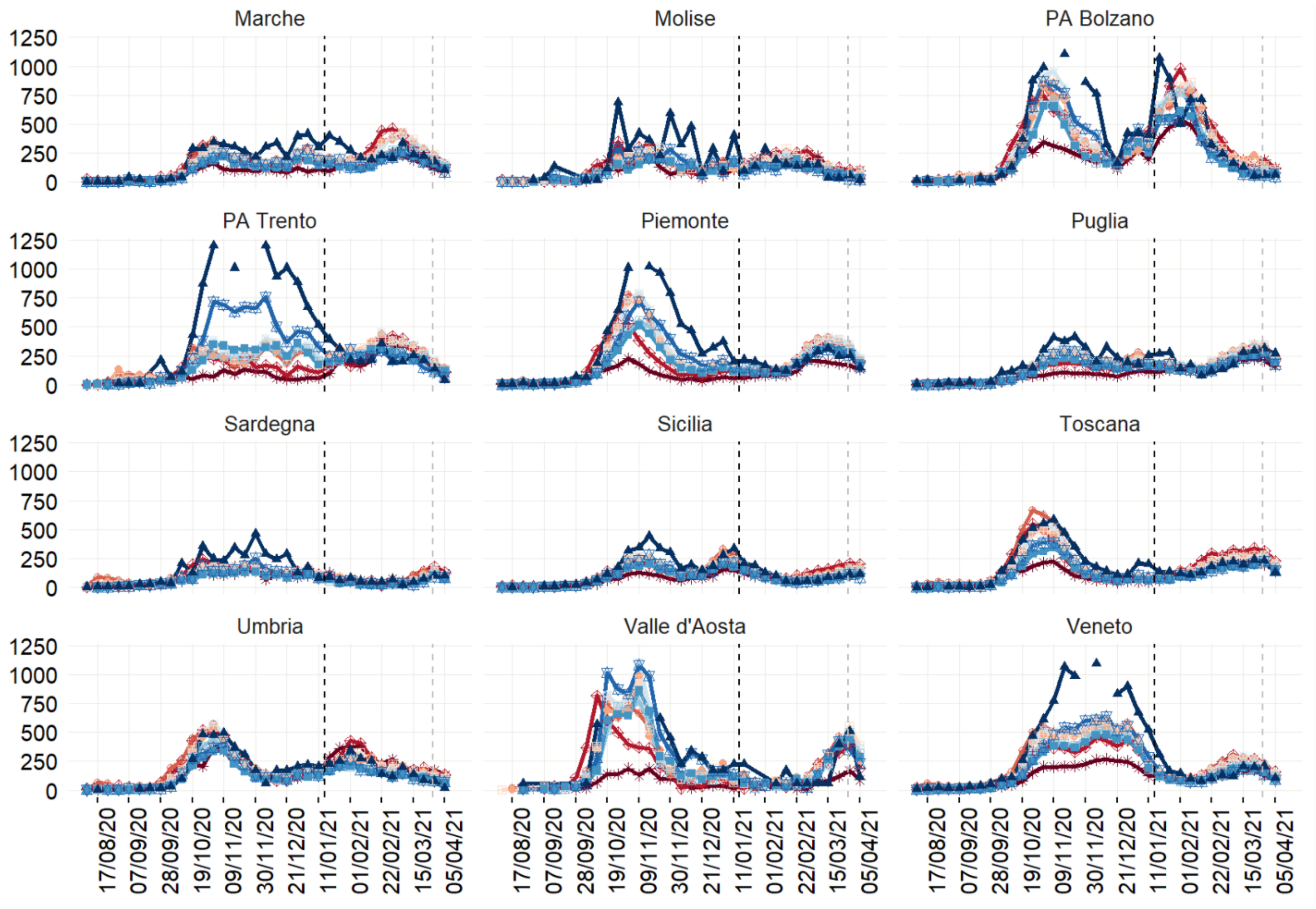

FIGURA 25B – INCIDENZA PER FASCIA D'ETÀ PER REGIONE/PA

Nota: Per una migliore visualizzazione l'asse delle ordinate è stato troncato a 1.250 casi (per 100.000 abitanti). Oltre la linea tratteggiata il dato deve essere considerato provvisorio. **LA LINEA TRATTEGGIATA NERA INDICA LA DATA DI ADOZIONE DELLA NUOVA DEFINIZIONE DI CASO**

- La campagna vaccinale iniziata il 27 dicembre 2020 prevedeva di vaccinare, nella prima fase, tutti gli operatori sanitari e sociosanitari, gli ospiti delle strutture residenziali e le persone con età >= 80 anni. Al 14 aprile 2021, sono state somministrate 13.943.068 delle 17.121.360 dosi di vaccino finora consegnate (9.801.798 prime dosi e 4.141.270 seconde dosi) (**Figura 26**) (https://github.com/italia/covid19-opendata-vaccini)

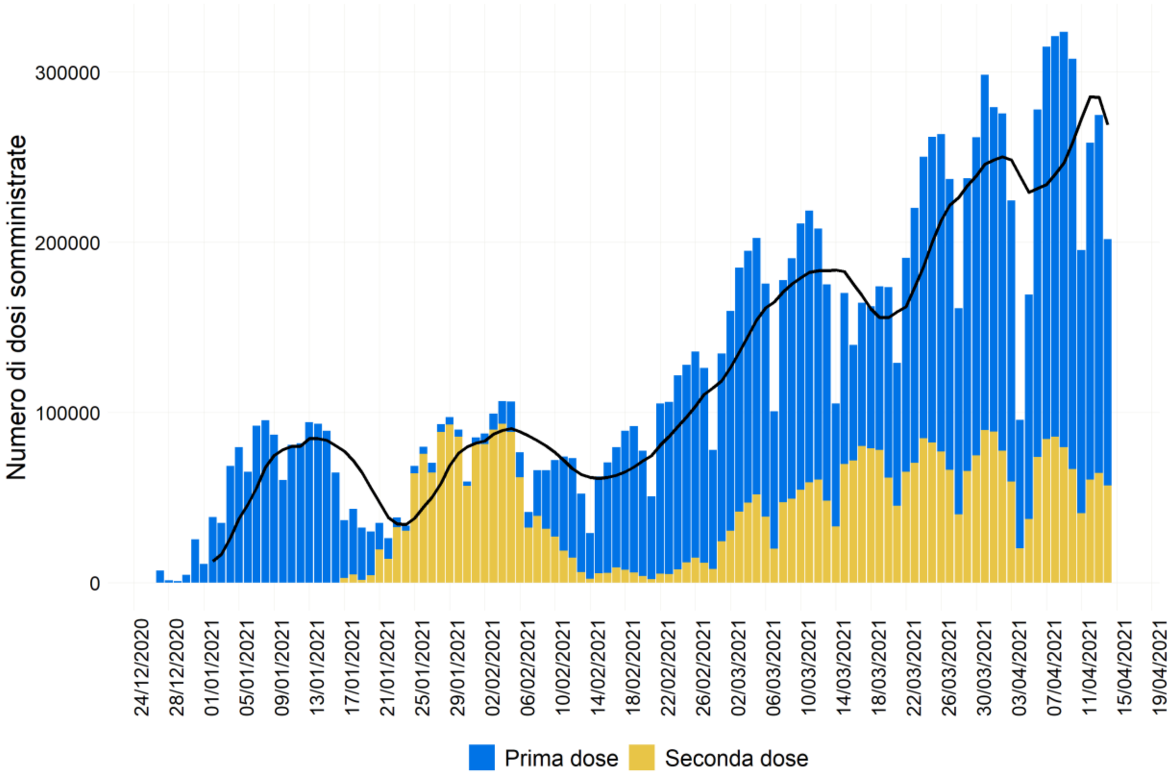

FIGURA 26 - NUMERO DI PRIME E SECONDE DOSI DI VACCINO SOMMINISTRATE GIORNALMENTE DAL 27/12/2020 AL 14/04/2021.

- Al 14 aprile, sono state somministrate 3.178.204 dosi a operatori sanitari e sociosanitari, 528.428 dosi a personale non sanitario, 4.835.406 dosi a persone di età >= 80 anni, 602.471 dosi a ospiti di strutture residenziali, 235.970 a personale delle forze armate, 1.124.516 a personale scolastico e 3.438.073 ad altri gruppi, che include i gruppi di età tra 60 e 79 anni (Figura 28). Analizzando i dati per fascia di età, il gruppo che in proporzione ha ricevuto il numero maggiore di dosi è la fascia >= 90 anni (il 76,5% circa ha ricevuto almeno una dose), seguito dalla fascia 80-89 anni (il 77,2% circa ha ricevuto almeno una dose).

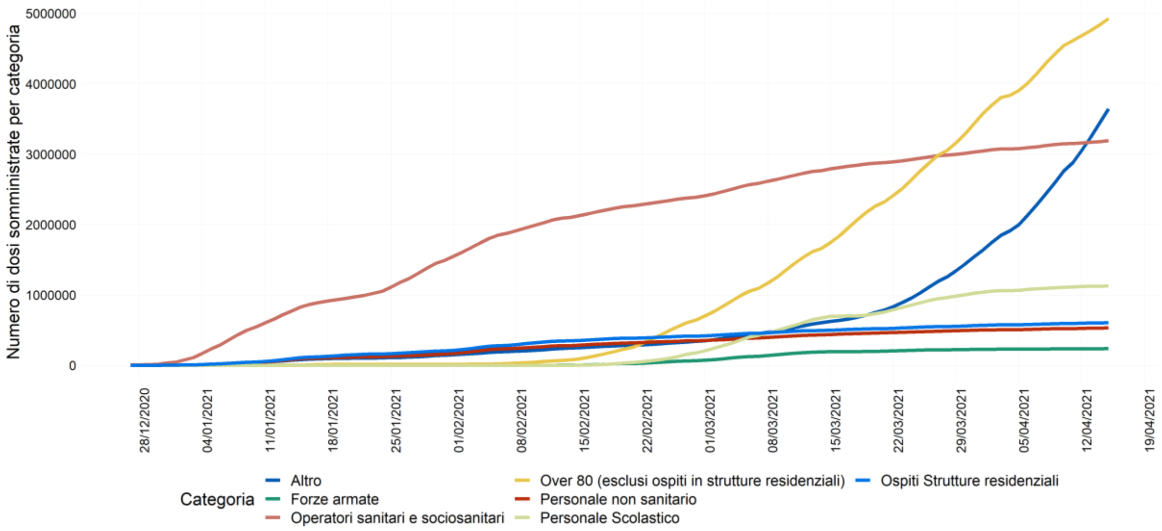

FIGURA 27 - NUMERO DI DOSI DI VACCINO SOMMINISTRATE PER CATEGORIA DI RISCHIO

- Le curve epidemiche dei casi riportati come operatori sanitari e quella dei casi non riportati come operatori sanitari hanno avuto un andamento molto simile fino alla seconda metà di gennaio, quando hanno iniziato a divergere, mostrando un trend visibilmente in calo per gli operatori sanitari a fronte di un trend stazionario, con tendenza a un evidente aumento dall'8 febbraio, nella popolazione generale. (**Figura 28**).

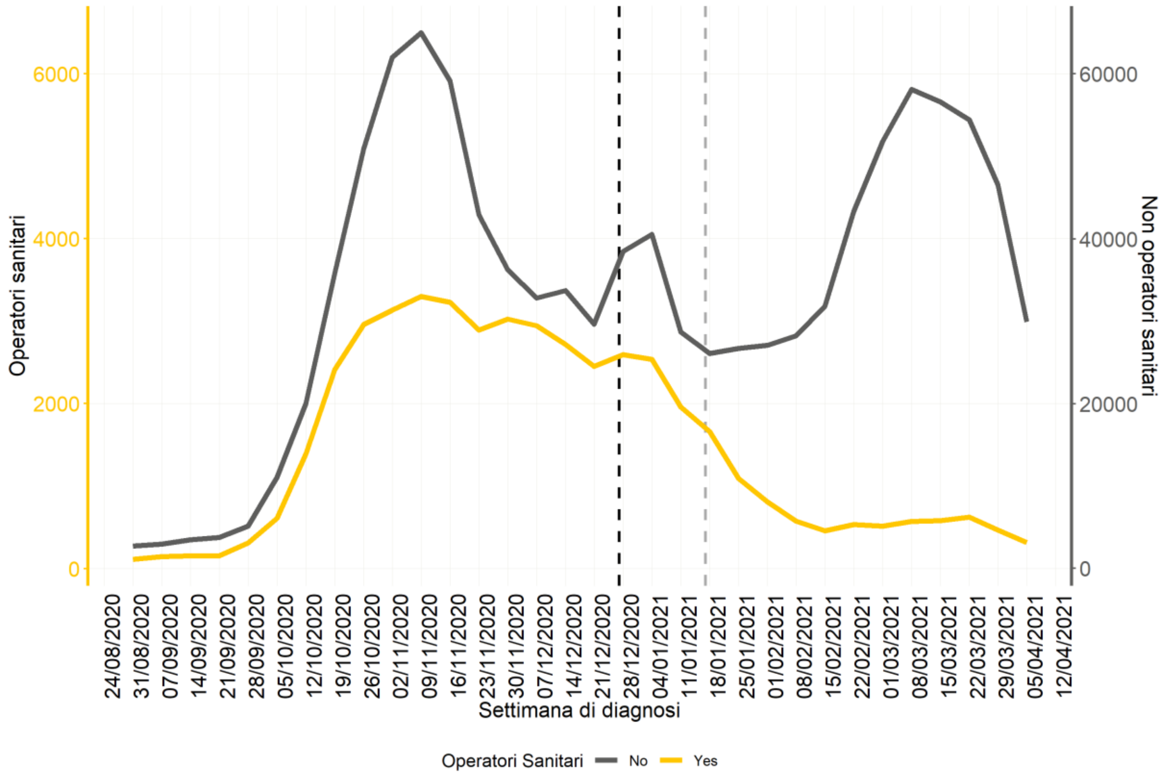

FIGURA 28 - ANDAMENTO DEL NUMERO ASSOLUTO DI CASI NEGLI OPERATORI SANITARI SINTOMATICI E NEL RESTO DELLA POPOLAZIONE.

LA LINEA NERA INDICA L'INIZIO DELLA CAMPAGNA VACCINALE, LINEA GRIGIA INDICA L'INIZIO DELLA SOMMINISTRAZIONE DELLA SECONDA DOSE

- Analizzando il numero di casi di infezione da SARS-CoV-2 nella popolazione suddivisa per fascia di età 60-69 anni, 70-79 anni e >=80 anni si osserva un andamento molto simile nelle due fasce ma con un'incidenza più elevata nella fascia di età >= 80 anni fino all'inizio di febbraio, quando, in concomitanza con la vaccinazione degli ultraottantenni, la curva mostra una inversione con una maggiore incidenza nei soggetti di età 60-69 anni.(**Figura 29**).

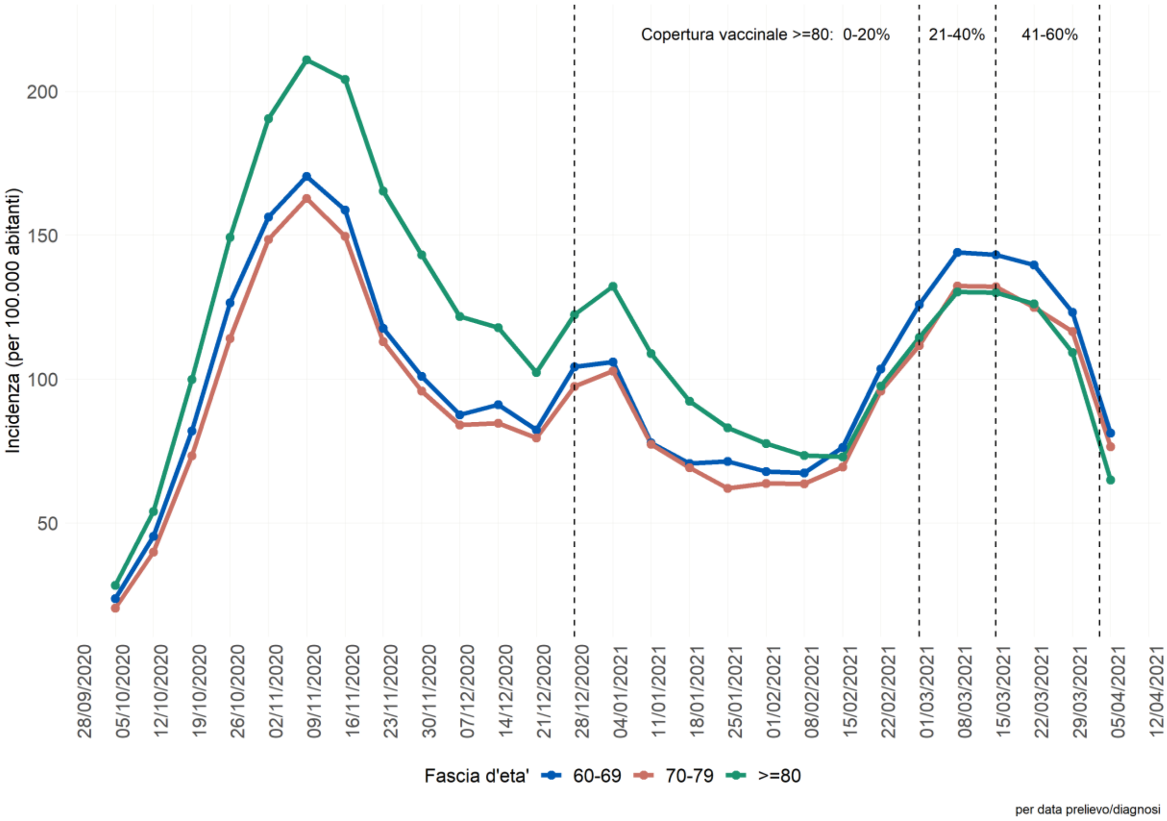

FIGURA 29 - ANDAMENTO DEL NUMERO DI CASI SEGNALATI NELLA POPOLAZIONE DI ETÀ 60-69 ANNI VS 70-79 ANNI E >=80 ANNI

LA LINEA NERA INDICA L'INIZIO DELLA CAMPAGNA VACCINALE, LINEA GRIGIA INDICA L'INIZIO DELLA SOMMINISTRAZIONE DELLA SECONDA DOSE

- In conclusione, le differenze nei trend osservati nel numero di casi tra gli operatori sanitari e nelle persone >=80 anni, sono attribuibili alla campagna di vaccinazione, sebbene la presente analisi sia puramente descrittiva e siano necessarie valutazioni più approfondite.
